# Semisynthesis, Biological Evaluation and Molecular Docking Studies of Barbatic Acid Derivatives as Novel Diuretic Candidates

**DOI:** 10.3390/molecules28104010

**Published:** 2023-05-10

**Authors:** Xiang Yu, Yi Sui, Yinkai Xi, Yan Zhang, Guoyong Luo, Yi Long, Wude Yang

**Affiliations:** 1 College of Pharmacy, Guizhou University of Traditional Chinese Medicine, Guiyang 550025, China; yuxiangjx@126.com (X.Y.);; 2 Guizhou Joint Laboratory for International Cooperation in Ethnic Medicine, Guizhou University of Traditional Chinese Medicine, Guiyang 550025, China

**Keywords:** barbatic acid, structural modification, diuretic activity, litholytic activity, molecular docking

## Abstract

Barbatic acid, a compound isolated from lichen, has demonstrated a variety of biological activities. In this study, a series of esters based on barbatic acid (**6a**–**q′**) were designed, synthesized, and evaluated for their diuretic and litholytic activity at a concentration of 100 μmol/L in vitro. All target compounds were characterized using ^1^H NMR, ^13^C NMR, and HRMS, and the spatial structure of compound **6w** was confirmed using X-ray crystallography. The biological results showed that some derivatives, including **6c**, **6b′**, and **6f′**, exhibited potent diuretic activity, and **6j** and **6m** displayed promising litholytic activity. Molecular docking studies further suggested that **6b′** had an optimal binding affinity to WNK1 kinases related to diuresis, while **6j** could bind to the bicarbonate transporter CaSR through a variety of forces. These findings indicate that some barbatic acid derivatives could be further developed into novel diuretic agents.

## 1. Introduction

Nephrolithiasis, also known as kidney stone disease or urolithiasis, is the formation of sediment when urine in the renal calyx and pelvis becomes oversaturated with minerals, which then precipitate and either float freely or attach to the kidneys to form stones [1,2]. Without prompt treatment, kidney stones will lead to ureteral obstruction, blood in urine, frequent urinary tract infection, vomiting or painful urination, and eventually, permanent renal function damage [3,4]. Over the past few decades, the global prevalence of nephrolithiasis has significantly increased, making it a significant public health problem due to its high incidence among working-age adults [5,6,7]. Treatment of kidney stones mainly involves promoting urination or surgically removing them. Thiazide diuretics are effective at reducing the risk of recurrent kidney stones in patients with a history of kidney stones or hypercalciuria [8].

**Figure 1:**
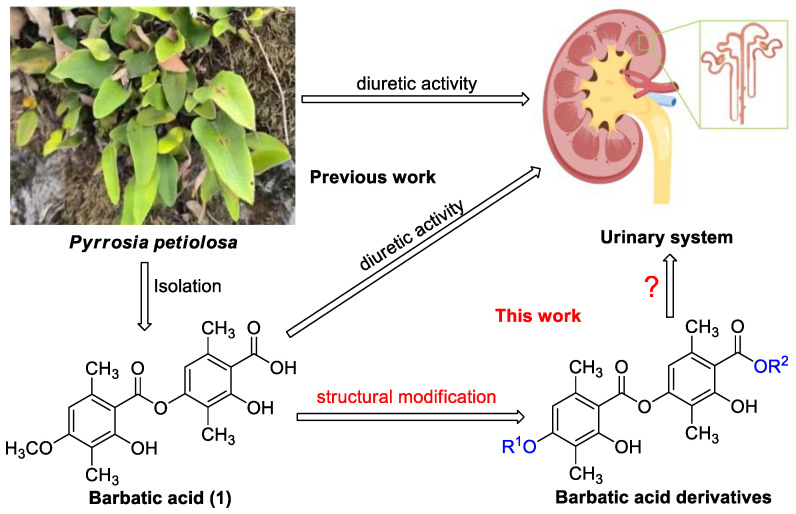
Design of barbatic acid derivatives as novel diuretic agents.

Natural products have a long history of use as drugs to treat various diseases for humankind [9,10]. The discovery and development of novel diuretic agents from natural products or their derivatives represents an attractive approach. Barbatic acid (**1**, Figure 1) is one of the depsides widely discovered in lichen [11], and was determined to have a variety of biological activities, including anticancer and schistosomicidal, inhibiting the growth of plants [12,13,14,15,16]. We first isolated barbatic acid from *Pyrrosia petiolosa*, a medicinal plant with significant diuretic effects, and found that it had a diuretic effect on mice [17]. However, little attention has been paid to barbatic acid and its derivatives as novel diuretic drugs.

The traditional methods for screening diuretic drugs are mostly in vivo experiments with mice, which have a long experimental cycle and are not suitable for screening massive compounds. As we know, the generation and excretion of urine involve two processes: glomerular filtration and tubular reabsorption. Reabsorption refers to the process in which the renal tubular epithelial cells transport water and certain solutes from the tubular fluid (urine) partially or entirely back into the bloodstream. Then, 99% of the water in the original urine filtered by the glomerulus is reabsorbed, and the transport of Na^+^ and Cl^−^ plays an important role in this process [18,19]. Thiazide and furosemide diuretics exert their diuretic effects by inhibiting the transport of Na^+^ and Cl^−^, which affects the tubular reabsorption of the original urine [20,21]. Based on this principle, we established a new type of cell screening model for diuretic active drugs. Madin–Darby canine kidney (MDCK) cells were inoculated into a Transwell chamber to simulate the renal tubules. This cell model could transport and absorb external NaCl, simulating the reabsorption phenomenon. After the cells were treated with drugs, their transport ability was inhibited, thereby exhibiting diuretic activity. The effectiveness of this model was confirmed by the validation of hydrochlorothiazide, which is a commercially available diuretic drug and could inhibit the transport of Na^+^ and Cl^−^ in renal tubules [22].

Encouraged by these results, we designed and synthesized a series of barbatic acid derivatives and tested their diuretic and litholytic effects. Their potential mechanisms of action were also explored by molecular docking methods.

## 2. Results and Discussion

### 2.1. Chemistry

**Scheme 1:**
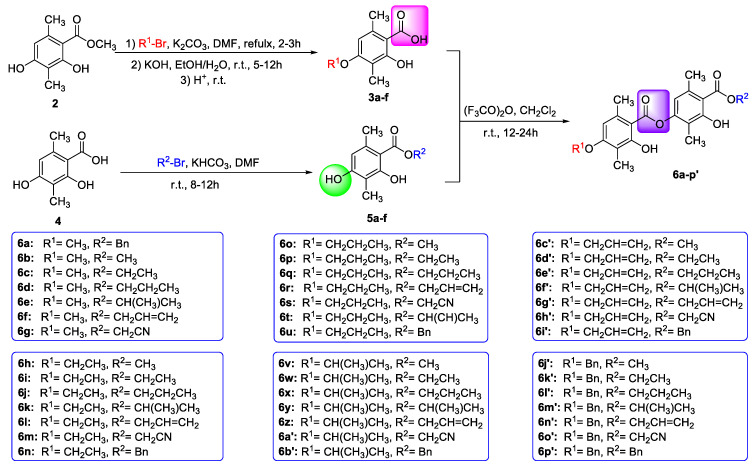
General synthetic procedure of barbatic acid derivatives (**6a**–**6p′**).

**Figure 2:**
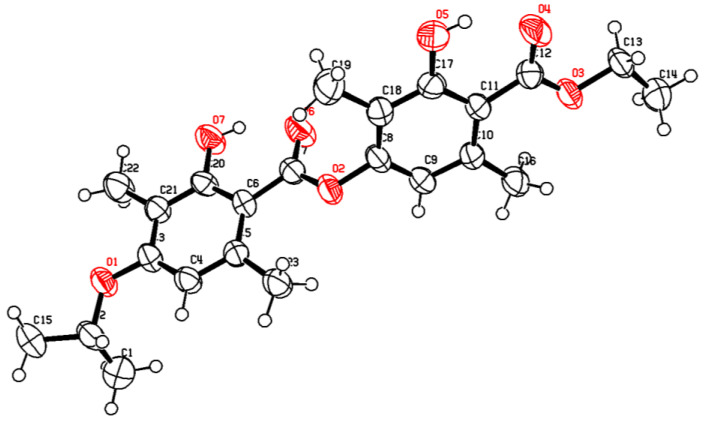
X-ray crystallographic structure of **6w**.

The synthesis of the ester derivatives based on barbatic acid was performed as illustrated in Scheme 1 via our previously reported method [23]. The *p*-OH of the benzene ring of **2** was selectively substituted by the hydrocarbon group via the Williamson reaction in the presence of K_2_CO_3_ in dimethyl formamide, followed by hydrolysis to obtain intermediates **3a**–**f** in 54–95% yields. On the other hand, the compounds **5a**–**f** were prepared in 49–90% yields by esterification of 2,4-dihydroxy-3,6-dimethylbenzoic acid (**4**) with different substituted brominated hydrocarbons. The final compounds (**6a**–**p′**) were formed in 27–50% yields via an esterification reaction between intermediates **3a**–**f** and **5a**–**f** in the presence of trifluoroacetic anhydride. The yields of the target product were relatively low, which might be due to more substituent groups on the benzene ring, causing steric hindrance and affecting the reaction. All target compounds were characterized by ^1^H NMR, ^13^C NMR, and HRMS (see Supplementary Materials). The stereochemistry of **6w** was further confirmed by the X-ray crystallographic analysis (Figure 2), and the crystallographic data were deposited at the Cambridge Crystallographic Data Centre (CCDC) with the no. 2253777.

### 2.2. In Vitro Cytotoxicity

First, we used the MTT method to perform cytotoxicity of barbatic acid derivatives (**1**, **6a**–**6p′**) on MDCK cells at the concentration of 100 μmol/L, and the results are shown in Table 1. According to the experimental results, compounds **1**, **6a**–**6p′** showed no or little toxicity to MDCK cells and could be used for subsequent activity testing.

### 2.3. In Vitro Diuretic Activity

Generally, the transport of Na^+^ and Cl^−^ plays an important role in glomerular filtration and tubular reabsorption. In this study, a Transwell chamber seeded with MDCK cells was used to simulate the renal tubules and investigate the inhibitory effect of barbatic acid derivatives (**1**, **6a**–**6p′**) on NaCl transport in the renal tubules at 100 μmol/L. As shown in Table 2, the results revealed that some compounds exhibited excellent inhibitory activity on Na^+^ transport, especially **6a**, **6c**, **6w**, **6b′**, and **6f′**, with inhibition rates of 33.51%, 47.18%, 33.11%, 33.08%, and 32.83%, respectively, significantly higher than the raw material, barbatic acid (**1**), and the positive control, hydrochlorothiazide. On the other hand, none of the compounds had a strong inhibitory effect on Cl^−^ transport, and some even showed a strong promoting effect. For example, compounds **6t** (−44.09%), **6w** (−80.44%), and **6y** (−43.32%) significantly promoted Cl^−^ transport. The structure–activity relationships (SARs) indicated that the introduction of ethyl and benzyl to the R^2^ of barbatic acid could yield more active compounds for inhibiting Na^+^ transport compared to those of ethyl, propyl, isopropyl, allyl, and cyanomethyl groups. For example, inhibition rates of transport of **6a** and **6c** were over 30%, and had better inhibitory ability than barbatic acid. In contrast, the inhibition rates of compounds **6b** and **6d**–**g** were all less than 10% or negative. When ethyl, propyl, and benzyl were substituted on R^1^, the introduction of any group could not enhance the inhibitory activity of barbatic acid, such as **6h**–**u** and **6j′**–**6p′**.

**Figure 3:**
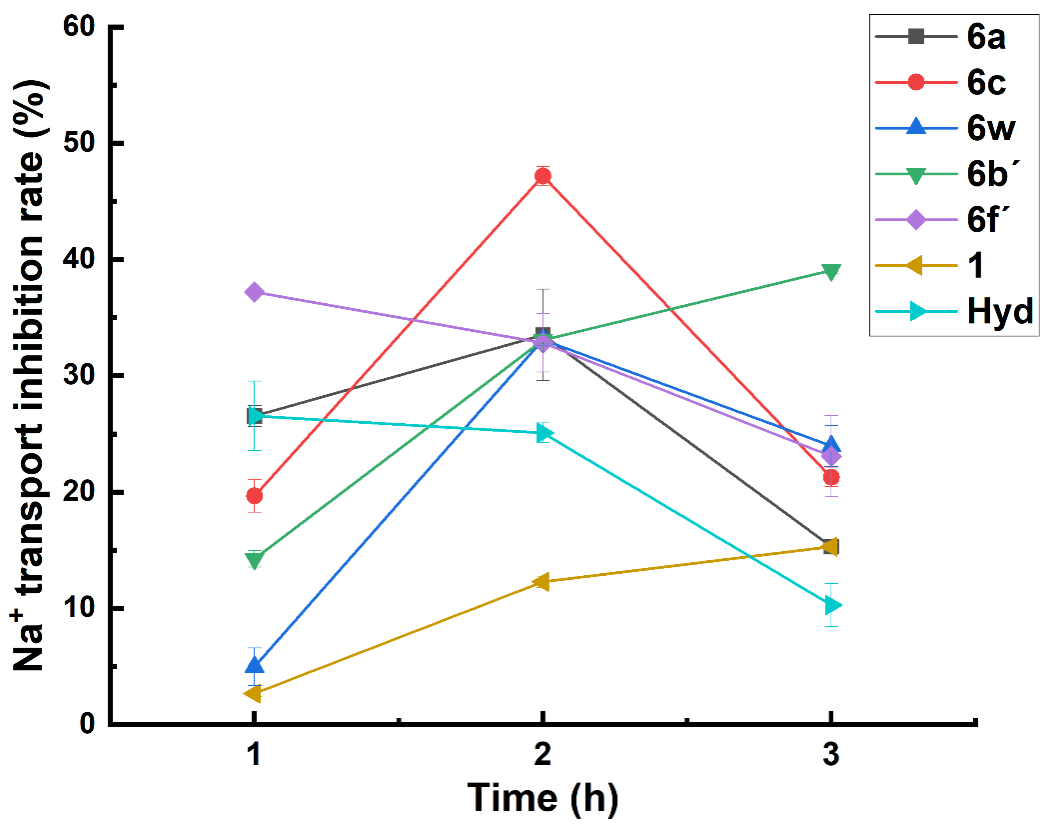
Na^+^ transport inhibition activity of barbatic acid derivatives at 1, 2, and 3 h.

In order to explore the relationship between the Na^+^ transport inhibition activity of potential compounds and time, the inhibitory activities of compounds **6a**, **6c**, **6w**, **6b′**, and **6f′** with inhibition rates higher than 30% were further evaluated at 1, 2, and 3 h. It could be seen from the data in Figure 3 that the transport inhibition rates of compounds **6c**, **6b′**, and **6f′** remained above 20%, with good inhibition steadiness. Therefore, the preliminary test results demonstrated that **6c**, **6b′**, and **6f′** have the potential for application in diuretic activity.

### 2.4. In Vitro Litholytic Activity

The formation process of kidney stones is caused by certain factors that result in an increase in the concentration of crystal substances in the urine or a decrease in solubility, leading to a state of supersaturation. Crystals precipitate, grow, and accumulate locally, ultimately forming stones. CaC_2_O_4_ is the most common component of kidney stones [24,25]. Therefore, one of the effective methods for preventing kidney stones is to prevent the reabsorption of Ca^2+^ and C_2_O_4_^2−^ and promote their excretion from the body in a timely manner.

The transwell chamber system with MDCK cells was simulated as renal tubules to test the inhibitory effect of barbatic acid derivatives (**1**, **6a**–**6p′**) on the transport of Ca^2+^ and C_2_O_4_^2−^ in renal tubules at 100 μmol/L. As represented in Table 3, compounds **6i**, **6j**, and **6m** exhibited significant inhibitory effects on C_2_O_4_^2−^ transport, with inhibitory rates of 54.05%, 42.26%, and 60.95%, respectively, much higher than that of the raw material barbatic acid (**1**, 2.97%). On the other hand, all compounds had little inhibitory effect on Ca^2+^ transport. The structure–activity relationships (SARs) were also observed. The results suggested that the introduction of an additional ethyl group to the R^1^ position and an ethyl, propyl, or cyanomethyl group to the R^2^ position of barbatic acid at the same time could markedly increase its C^2^O_4_^2−^ transport inhibition activity. On the other hand, the introduction of the benzyl group to the R^1^ or R^2^ of barbatic acid could not lead to more promising compounds, such as **6a**, **6n**, **6u**, **6b′**, and **6i′**–**p′**.

**Figure 4:**
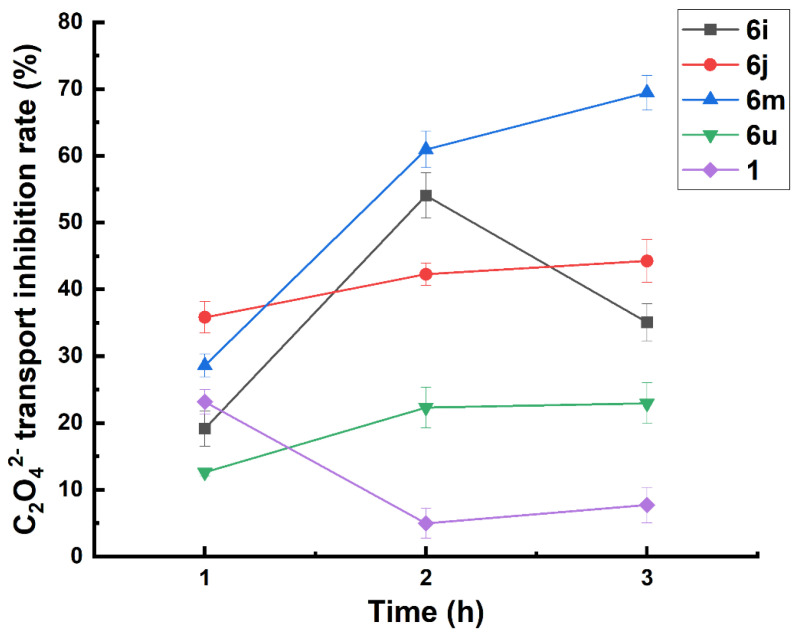
C^2^O_4_^2−^ transport inhibition activity of barbatic acid derivatives at 1, 2, and 3 h.

As shown in Figure 4, the C^2^O_4_^2−^ transport inhibition activities of **6i**, **6j**, **6m**, and **6u** were also observed at different time periods, and **6j** and **6m** expressed very strong and continual inhibitory activities against C^2^O_4_^2−^ transport. Thus, it could be seen that **6j** and **6m** have the potential for application in litholytic activity.

### 2.5. Molecular Docking 

To explore the possible inhibition mechanism of the potent compound, molecular modeling studies were also performed on compounds **6b′** and **6j** in the active site of WNK1 kinase domain and calcium-sensing receptor (CaSR), respectively. WNK kinases play key roles in blood pressure regulation and electrolyte and body fluid homeostasis. WNK463 increases urine flow rate and urinary sodium excretion [26]. CaSR is a cell surface sensor for Ca^2+^ and primarily regulates calcium homeostasis in humans. Cinacalcet is a calcimimetic-positive allosteric modulator that clinically targets CaSR [27]. WNK463 and cinacalcet were first re-docked into the active site to validate the docking reliability. The results showed that the binding modes of co-crystallized and re-docked WNK463 and cinacalcet were almost the same in the active site of the WNK1 kinase domain and CaSR, except for the trifluoromethyl substituent of the cinacalcet (Figure S1). The flexible linker of a three-carbon chain led to the structural change of the trifluoromethyl substituent in the ligand preparation. After validating the docking reliability, the established binding modes were then employed to evaluate the inhibition activity of the potent compound. 

**Figure 5:**
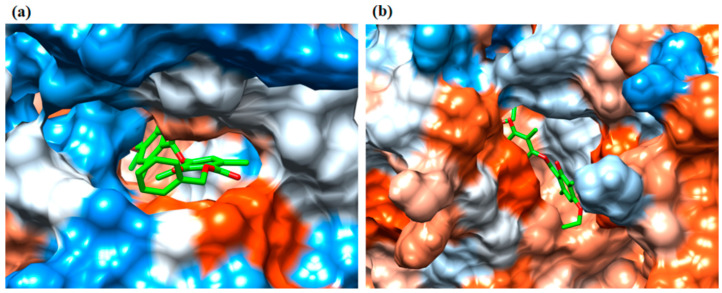
The molecular surface of the active site of WNK1 kinase domain (**a**) and CaSR (**b**) color-coded by amino acid hydrophobicity. The surface ranges from orange to red for the most hydrophobic to blue for the most polar residues, with white in between. Compounds **6b′** and **6j** in the docked complex are shown in green.

**Figure 6:**
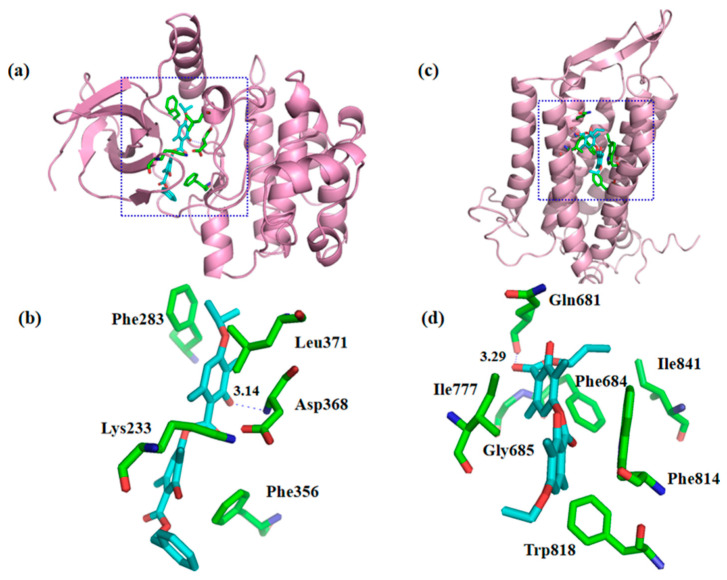
Interaction modes of compounds **6b′** with WNK1 kinase domain (**a**,**b**) and compound **6j** with CaSR (**c**,**d**).

In this study, the binding energy of compound **6b′** and WNK1 was −9.81 kcal/mol. The binding energy of compound 6j and CaSR was −7.89 kcal/mol. Therefore, the results indicated that compounds **6b′** and **6j** had better binding activity with core targets. On the other hand, compound **6b′** was wholly buried into the binding pocket (Figure 5), and primarily interacted with the α-C helix and the hinge portion of the WNK1 kinase domain, which is similar to WNK463 (Figure S1 in the Supplementary Materials). Yamada et al. found that the exquisite selectivity of WNK463 for the WNK kinase family was highly related to the interactions between the hinge portion and the a-C helix [26]. Specifically, compound **6b′** mainly formed polar interactions with Lys233 (Figure 6) and a hydrogen bond with Asp368, which were key for Na^+^ and Cl^−^ transport. In addition, the hydrophobic interactions with Phe283, Leu371, and Phe356 were important for the binding of compound **6b′** and the WNK1 kinase domain (Figure 6a,b). Compared with barbatic acid, the introductions of isopropyl and benzyl groups increased the hydrophobic interactions between compound **6b′** and the WNK1 kinase domain. Phenyl, methyl, ethyl, and propyl groups of compound **6j** primarily interacted with the hydrophobic binding site of CaSR (Figure 5), which consisted of Phe684, Gly685, Ile777, Phe814, Trp818, and Ile841, located on TM3, TM5, TM5, and TM7. (Figure 6c,d). Additionally, the carbonyl group of compound **6j** and Gln681 of CaSR formed an important hydrogen bond. The study by Skiniotis et al. indicated that naphthylethylamine and naphthyl groups of cinacalcet formed polar interactions with Gln681 and hydrophobic interactions with Phe684, Ile777, and Trp818, respectively. These interactions are key for cinacalcet and CaSR [27], which were also formed between compound **6j** and CaSR. Moreover, the hydrophobic interactions between compound **6j** and CaSR are more favorable. Compared with barbatic acid, the introductions of ethyl and propyl groups increased hydrophobic interactions between compound **6j** and CaSR. The important hydrophobic and hydrogen bond interactions between compound **6j** and CaSR were favorable for Ca^2+^ transmembrane transport. Therefore, the potential of diuresis and relieving stranguria of barbatic acid and their derivatives are contributed by inhibiting the activities of the WNK1 kinase domain and CaSR, transporting Na^+^, and Ca^2+^. Their binding driving force was primarily polar, hydrophobic, and hydrogen bond interactions.

## 3. Material and Methods

### 3.1. Chemistry

All reagents and solvents were of reagent grade or purified according to standard methods before use. Analytical thin-layer chromatography (TLC) was performed with silica gel plates using silica gel 60 GF_254_ (Qingdao Haiyang Chemical Co., Ltd., Qingdao, China). Melting points were determined on an XT-4 digital melting point apparatus (Beijing Tech Instrument Co., Ltd., Beijing, China) and were uncorrected. Proton nuclear magnetic resonance spectra (^1^H NMR) were recorded on a Bruker Avance DMX 400 MHz instrument (Bruker, Bremerhaven, Germany) in CDCl_3_ or DMSO-*d*_6_ using TMS (tetramethylsilane) as the internal standard. Mass spectrometry (MS) was carried out with a Waters XEVO TQ-D instrument (Waters, Milford, MA, USA). High-resolution mass spectrometry (HRMS) was carried out with a Xevo G2-SQTOF instrument (Waters, Milford, MA, USA).

#### 3.1.1. Synthesis of Intermediates **3a**–**f**

A mixture of methyl atratate (**2**, 300 mg, 1.5 mmol), potassium carbonate (310.9 mg, 2.25 mmol), and brominated hydrocarbon (2 mmol) in DMF (5 mL) was refluxed for 2–3 h until the reaction was completed according to TLC analysis. After being cooled to room temperature, the mixture was poured into 1 mol/L hydrochloric acid (30 mL) and extracted with ethyl acetate (30 mL × 3) and washed with water (10 mL × 2). The combined organic layer was dried over anhydrous Na_2_SO_4_, filtered, and evaporated. The crude products were purified by flash chromatography on silica gel (petroleum ether: ethyl acetate = 3:1). Then, the crude products were added to the mixed solution (ethyl alcohol: water = 25:1) with KOH (673.3 mg, 12 mmol) and stirred at room temperature for 5–12 h. After completion of the reaction, the pH was adjusted to 1–2 with 1M hydrochloric acid, and then filtrated to give soil product **3a**–**f** by recrystallization in petroleum ether.

Data for *2-Hydroxy-4-methoxy-3,6-dimethylbenzoic acid* (**3a**): Yield: 97%, white solid, mp 200–202 °C; ^1^H NMR (400 MHz, DMSO-*d*_6_): *δ* 6.46 (s, 1H), 3.83 (s, 3H), 2.51 (s, 3H), 1.96 (s, 3H); ^13^C NMR (100 MHz, DMSO-*d*_6_): *δ* 174.2, 161.9, 161.0, 140.5, 109.5, 106.1, 105.6, 55.8, 24.2, 8.1; MS *m/z*: 197.10 ([M + H]^+^, 100).

Data for *4-(benzyloxy)-2-hydroxy-3,6-dimethylbenzoic acid* (**3b**): Yield: 89%, white solid, mp 200–203 °C; ^1^H NMR (400 MHz, DMSO-*d*_6_); *δ* 7.48–7.30 (m, 5H), 6.57 (s, 1H), 5.18 (s, 2H), 2.48 (s, 3H), 2.01 (s, 3H). ^13^C NMR (100 MHz, CDCl_3_): *δ* 179.1, 167.0, 165.1, 145.4, 142.2, 133.7 × 2, 133.1, 132.6 × 2, 115.0, 112.4, 110.9, 74.5, 29.2, 13.3; MS (ESI) *m/z*: 273.10 ([M + H]^+^, 100).

Data for *4-ethoxy-2-hydroxy-3,6-dimethylbenzoic acid* (**3c**): Yield: 81%, white solid, mp 222–224 °C; ^1^H NMR (400 MHz, DMSO-*d*_6_): *δ* 6.43 (s, 1H), 4.09 (s, 2H), 2.49 (s, 3H), 1.96 (s, 3H), 1.34 (s, 3H); ^13^C NMR (100 MHz, DMSO-*d*_6_): *δ* 174.4, 162.2, 160.6, 140.6, 109.8, 107.1, 105.6, 63.8, 24.4, 15.1, 8.3; MS (ESI) *m/z*: 211.10 ([M + H]^+^, 100).

Data for *2-hydroxy-3,6-dimethyl-4-propoxybenzoic acid* (**3d**): Yield: 90%, white solid, mp 189–191 °C; ^1^H NMR (400 MHz, DMSO-*d*_6_): *δ* 6.43 (s, 1H), 3.98 (t, *J* = 6.4 Hz, 2H), 2.48 (s, 3H), 1.96 (s, 3H), 1.79–1.68 (m, 2H), 0.99 (t, *J* = 7.4 Hz, 3H); ^13^C NMR (100 MHz, DMSO-*d*_6_): *δ* 174.4, 162.2, 160.7, 140.6, 109.8, 107.1, 105.7, 69.5, 24.4, 22.5, 10.8, 8.3; MS (ESI) *m/z*: 247.10 ([M + Na]^+^, 100).

Data for *2-hydroxy-4-isopropoxy-3,6-dimethylbenzoic acid* (**3e**): Yield: 87%, white solid, mp 167–169 °C; ^1^H NMR (400 MHz, DMSO-*d*_6_): *δ* 6.46 (s, 1H), 4.70 (p, *J* = 6.0 Hz, 1H), 2.49 (s, 3H), 1.94 (s, 3H), 1.28 (s, 3H), 1.27 (s, 3H); ^13^C NMR (100 MHz, DMSO-*d*_6_): *δ* 174.3, 162.5, 159.8, 140.4, 110.8, 108.3, 105.4, 70.1, 24.4, 22.5 × 2, 8.5; MS (ESI) *m/z*: 247.10 ([M + Na]^+^, 100).

Data for *4-(allyloxy)-2-hydroxy-3,6-dimethylbenzoic acid* (**3f**): Yield: 54%, white solid, mp 188–191 °C; ^1^H NMR (400 MHz, DMSO-*d*_6_): *δ* 6.45 (s, 1H), 6.05–6.00 (m, 1H), 5.40–5.35 (m, 1H), 5.26–5.22 (m, 1H), 4.62 (dt, *J* = 5.0, 1.7 Hz, 2H), 2.48 (s, 3H), 1.98 (s, 3H); ^13^C NMR (100 MHz, DMSO-*d*_6_) *δ* 174.3, 162.2, 160.1, 140.5, 133.9, 117.6, 110.0, 107.4, 105.9, 68.6, 24.4, 8.3; MS (ESI) *m/z*: 223.10 ([M + H]^+^, 100).

#### 3.1.2. Synthesis of Intermediates **5a**–**g**

Compound **4** (500 mg, 2.74 mmol) was added into 5 mL DMF, followed by the addition of KHCO_3_ (411.45 mg, 4.11 mmol) and brominated hydrocarbon (3.3 mmol). The reaction mixture was stirred at room temperature for 8–12 h and detected by TLC. After completion of the reaction, the mixture solvent was extracted with ethyl acetate (30 mL × 3) and washed with water (10 mL × 2). The combined organic layer was dried over anhydrous Na_2_SO_4_, filtered, and evaporated. The given residue was purified by flash chromatography on silica gel (petroleum ether to ethyl acetate = 5:1) to give the target product **5a**–**f**.

Data for *Benzyl 2,4-dihydroxy-3,6-dimethylbenzoate* (**5a**): Yield: 90%, white solid, mp 110–112 °C; ^1^H NMR (400 MHz, CDCl_3_): *δ* 12.05 (s, 1H), 7.39 (brs, 5H), 6.19 (s, 1H), 5.38 (s, 2H), 2.45 (s, 3H), 2.11 (s, 3H); ^13^C NMR (100 MHz, CDCl_3_): *δ* 172.4, 163.7, 158.6, 140.7, 135.9, 129.1 × 2, 128.9 × 2, 111.1 × 2, 109.1, 105.6, 67.5, 24.9, 8.1; MS *m/z*: 295.10 ([M + Na]^+^, 100).

Data for *ethyl 2,4-dihydroxy-3,6-dimethylbenzoate* (**5b**): Yield: 62%, white solid, mp 134–136 °C; ^1^H NMR (400 MHz, CDCl_3_): *δ* 12.12 (s, 1H), 6.20 (s, 1H), 4.39 (q, *J* = 5.0 Hz, 2H), 2.47 (s, 3H), 2.10 (s, 3H), 1.41 (t, *J* = 7.4 Hz, 3H); ^13^C NMR (100 MHz, CDCl_3_): *δ* 172.3, 163.2, 158.1, 140.3, 110.7, 108.7, 105.4, 61.4, 24.3, 14.3, 7.7; MS (ESI) *m/z*: 211.10 ([M + H]^+^, 100).

Data for *propyl 2,4-dihydroxy-3,6-dimethylbenzoate* (**5c**): Yield: 51%, white solid, mp 141–143 °C; ^1^H NMR (400 MHz, DMSO-*d*_6_): *δ* 11.83 (s, 1H), 10.12 (s, 1H), 6.29 (s, 1H), 4.24 (t, *J* = 6.4 Hz, 2H), 2.40 (s, 3H), 1.94 (s, 3H), 1.73 (h, *J* = 7.0 Hz, 2H), 0.98 (t, *J* = 7.4 Hz, 3H); ^13^C NMR (100 MHz, DMSO-*d*_6_): *δ* 172.1, 162.5, 160.5, 139.3, 111.0, 108.6, 104.2, 67.1, 24.1, 21.9, 11.0, 8.4; MS (ESI) *m/z*: 247.10 ([M + Na]^+^, 100).

Data for *isopropyl 2,4-dihydroxy-3,6-dimethylbenzoate* (**5d**): Yield: 49%, white solid, mp 141–143 °C; ^1^H NMR (400 MHz, CDCl_3_): *δ* 12.18 (s, 1H), 6.20 (s, 1H), 5.29 (p, *J* = 6.2 Hz, 1H), 2.47 (s, 3H), 2.10 (s, 3H), *δ* 1.39 (d, *J* = 6.3 Hz, 6H); ^13^C NMR (100 MHz, CDCl_3_): *δ* 171.6, 163.1, 157.8, 140.1, 110.5, 108.5, 105.6, 69.2, 24.3, 22.0 × 2, 7.6; MS (ESI) *m/z*: 247.10 ([M + Na]^+^, 100).

Data for *allyl 2,4-dihydroxy-3,6-dimethylbenzoate* (**5e**): Yield: 79%, white solid, mp 219–222 °C; ^1^H NMR (400 MHz, CDCl_3_): *δ* 12.03 (s, 1H), 6.20 (s, 1H), 6.16–5.89 (m, 1H), 5.46–5.26 (m, 2H), 4.84 (dt, *J* = 5.8, 1.4 Hz, 2H), 2.48 (s, 3H), 2.10 (s, 3H); ^13^C NMR (100 MHz, CDCl_3_): *δ* 172.0, 163.3, 158.2, 140.4, 131.9, 118.9, 110.7, 108.7, 105.2, 66.0, 24.4, 7.8; MS (ESI) *m/z*: 245.10 ([M + Na]^+^, 100).

Data for *cyanomethyl 2,4-dihydroxy-3,6-dimethylbenzoate* (**5f**): Yield: 83%, white solid, mp 205–207 °C; ^1^H NMR (400 MHz, DMSO-*d*_6_): *δ* 10.80 (s, 1H), 10.25 (s, 1H), 6.33 (s, 1H), 5.19 (s, 2H), 2.36 (s, 3H), 1.96 (s, 3H); ^13^C NMR (100 MHz, DMSO-*d*_6_): *δ* 169.3, 160.9, 160.5, 138.6, 116.0, 110.7, 108.5, 103.9, 49.4, 22.9, 8.0; MS (ESI) *m/z*: 244.10 ([M + Na]^+^, 100).

#### 3.1.3. General Synthetic Procedure of Barbatic Acid Derivatives (**6a**–**6p′**)

Compounds **3a**–**f** (1 mmol) and **2**, **5a**–**f** (1 mmol) were added into 20 mL methylene chloride, followed by the addition of 0.31 mL trifluoroacetic anhydride. The reaction mixture was stirred at room temperature for 12–24 h and detected by TLC. When the reaction was over, the solvent was removed by decompression, and then the residue was purified by flash chromatography on silica gel (petroleum ether: ethyl acetate = 25:1) to give the target product **6a**–**6p′**.

Data for *Benzyl 2-hydroxy-4-((2-hydroxy-4-methoxy-3,6-dimethylbenzoyl)oxy)-3,6-dimethylbenzoate* (**6a**): Yield: 50%, white solid, mp 113–115 °C; ^1^H NMR (400 MHz, CDCl_3_): *δ* 11.92 (s, 1H), 11.51 (s, 1H), 7.48–7.34 (m, 5H), 6.51 (s, 1H), 6.38 (s, 1H), 5.43 (s, 2H), 3.90 (s, 3H), 2.69 (s, 3H), 2.52 (s, 3H), 2.10 (s, 3H), 2.09 (s, 3H); ^13^C NMR (100 MHz, CDCl_3_): *δ* 172.0, 170.6, 163.5 × 2, 162.7, 153.0, 141.2, 140.3, 135.5, 129.2 × 2, 129.1, 128.9 × 2, 117.5, 116.9, 111.8, 110.4, 106.9, 104.8, 67.9, 56.0, 25.5, 24.8, 9.8, 8.3; HRMS *m/z* calcd. for C_26_H_26_O_7_Na ([M + Na]^+^) 473.1570, found 473.1569 (Figures S2–S4).

Data for *3-hydroxy-4-(methoxycarbonyl)-2,5-dimethylphenyl 2-hydroxy-4-methoxy-3,6-dimethylbenzoate* (**6b**): Yield: 45%, white solid, mp 177–180 °C; ^1^H NMR (400 MHz, CDCl_3_): *δ* 11.93 (s, 1H), 11.51 (s, 1H), 6.52 (s, 1H), 6.38 (s, 1H), 3.98 (s, 3H), 3.90 (s, 3H), 2.69 (s, 3H), 2.54 (s, 3H), 2.10 (s, 3H), 2.09 (s, 3H); ^13^C NMR (100 MHz, CDCl_3_): *δ* 172.4, 170.3, 163.1, 162.9, 162.3, 152.6, 140.8, 139.8, 117.0, 116.5, 111.4, 110.0, 106.5, 104.4, 55.7, 52.3, 25.2, 24.1, 9.4, 7.9; HRMS *m/z* calcd. for C_20_H_22_O_7_Na ([M + Na]^+^) 397.1257, found 397.1252 (Figures S5–S7).

Data for *4-(ethoxycarbonyl)-3-hydroxy-2,5-dimethylphenyl 2-hydroxy-4-methoxy-3,6-dimethylbenzoate* (**6c**): Yield: 30%, white solid, mp 175–178 °C; ^1^H NMR (400 MHz, CDCl_3_): *δ* 12.00 (s, 1H), 11.52 (s, 1H), 6.51 (s, 1H), 6.38 (s, 1H), 4.45 (q, *J* = 7.1 Hz, 2H), 3.90 (s, 3H), 2.69 (s, 3H), 2.55 (s, 3H), 2.10 (s, 3H), 2.09 (s, 3H), 1.43 (t, *J* = 7.1 Hz, 3H); ^13^C NMR (100 MHz, CDCl_3_): *δ* 171.8, 170.2, 162.9, 162.8, 162.2, 152.4, 140.7, 139.7, 139.6, 116.9, 116.3, 111.3, 110.1, 106.4, 104.3, 61.7, 55.5, 25.1, 24.1, 14.2, 9.3, 7.8; HRMS *m/z* calcd. for C_21_H_24_O_7_Na ([M + Na]^+^) 411.1414, found 411.1410 (Figures S8–S10).

Data for *3-hydroxy-2,5-dimethyl-4-(propoxycarbonyl)phenyl 2-hydroxy-4-methoxy-3,6-dimethylbenzoate* (**6d**): Yield: 32%, white solid, mp 146–149 °C; ^1^H NMR (400 MHz, CDCl_3_): *δ* 12.04 (s, 1H), 11.52 (s, 1H), 6.52 (s, 1H), 6.38 (s, 1H), 4.36 (t, *J* = 6.6 Hz, 2H), 3.90 (s, 3H), 2.69 (s, 3H), 2.56 (s, 3H), 2.10 (s, 3H), 2.09 (s, 3H), 1.83 (h, *J* = 7.3 Hz, 2H), 1.05 (t, *J* = 7.4 Hz, 3H); ^13^C NMR (100 MHz, CDCl_3_): *δ* 172.0, 170.2, 163.0, f162.9, 162.2, 152.4, 140.7, 139.6, 116.9, 116.3, 111.3, 110.1, 106.4 × 2, 104.3, 67.5, 55.5, 25.1, 24.1, 21.9, 10.7, 9.3, 7.8; HRMS *m/z* calcd. for C_22_H_26_O_7_Na ([M + Na]^+^) 425.1570, found 425.1564 (Figures S11–S13).

Data for *3-hydroxy-4-(isopropoxycarbonyl)-2,5-dimethylphenyl 2-hydroxy-4-methoxy-3,6-dimethylbenzoate* (**6e**): Yield: 48%, white solid, mp 135–138 °C; ^1^H NMR (400 MHz, CDCl_3_): *δ* 12.05 (s, 1H), 11.53 (s, 1H), 6.50 (s, 1H), 6.38 (s, 1H), 5.34 (p, *J* = 6.3 Hz, 1H), 3.90 (s, 3H), 2.69 (s, 3H), 2.55 (s, 3H), 2.10 (s, 3H), 2.08 (s, 3H), 1.41 (d, *J* = 6.3 Hz, 6H); ^13^C NMR (100 MHz, CDCl_3_): *δ* 171.3, 170.2, 162.9, 162.8, 162.2, 152.3, 140.7, 139.6, 116.9, 116.2, 111.3, 110.4, 106.4, 104.3, 69.8, 55.5, 25.1, 24.2, 21.9 × 2, 9.3, 7.8; HRMS *m/z* calcd. for C_22_H_26_O_7_Na ([M + Na]^+^) 425.1570, found 425.1566 (Figures S14–S16).

Data for *allyl 2-hydroxy-4-(2-hydroxy-4-methoxy-3,6-dimethylbenzoyloxy)-3,6-dimethylbenzoate* (**6f**): Yield: 48%, white solid, mp 155–157 °C; ^1^H NMR (400 MHz, CDCl_3_): *δ* 11.91 (s, 1H), 11.51 (s, 1H), 6.52 (s, 1H), 6.37 (s, 1H), 6.04 (ddt, *J* = 17.3, 10.4, 5.8 Hz, 1H), 5.46–5.28 (m, 2H), 4.89 (dt, *J* = 5.9, 1.4 Hz, 2H), 3.90 (s, 3H), 2.68 (s, 3H), 2.56 (s, 3H), 2.10 (s, 3H), 2.09 (s, 3H); ^13^C NMR (100 MHz, CDCl_3_): *δ* 171.9, 170.6, 163.4 × 2, 162.7, 153.0, 141.2, 140.2, 131.9, 119.7, 117.4, 116.8, 111.7, 110.3, 106.8, 104.7, 66.7, 56.0, 25.5, 24.6, 9.7, 8.2; HRMS *m/z* calcd. for C_22_H_24_O_7_Na ([M + Na]^+^) 423.1414, found 423.1411 (Figures S17–S19).

Data for *allyl 4-((cyanomethoxy)carbonyl)-3-hydroxy-2,5-dimethylphenyl 2-hydroxy-4-methoxy-3,6-dimethylbenzoate* (**6g**): Yield: 45%, white solid, mp 184–187 °C; ^1^H NMR (400 MHz, DMSO-*d*_6_): *δ* 10.74 (s, 1H), 9.97 (s, 1H), 6.75 (s, 1H), 6.61 (s, 1H), 5.22 (s, 2H), 3.87 (s, 3H), 2.57 (s, 3H), 2.30 (s, 3H), 2.01 (d, *J* = 1.5 Hz, 6H). ^13^C NMR (100 MHz, DMSO-*d*_6_): *δ* 169.2, 167.5, 161.5, 159.9, 156.0, 151.5, 139.4, 136.0, 116.9, 116.8, 116.3, 116.2, 110.5, 107.5, 106.8, 56.2, 50.1, f23.5, 20.3, 9.9, 8.5; HRMS *m/z* calcd. for C_21_H_21_NO_7_Na ([M + Na]^+^) 422.1210, found 422.1195 (Figures S20–S22).

Data for *3-hydroxy-4-(methoxycarbonyl)-2,5-dimethylphenyl 4-ethoxy-2-hydroxy-3,6-dimethylbenzoate* (**6h**): Yield: 36%, white solid, mp 152–155 °C; ^1^H NMR (400 MHz, DMSO-*d*_6_): *δ* 10.77 (s, 1H), 10.53 (s, 1H), 6.72 (s, 1H), 6.59 (s, 1H), 4.14 (q, *J* = 6.8 Hz, 2H), 3.88 (s, 3H), 2.55 (s, 3H), 2.35 (s, 3H), 2.01 (s, 3H), 2.00 (s, 3H), 1.36 (t, *J* = 6.8 Hz, 3H). ^13^C NMR (100 MHz, CDCl_3_): *δ* 172.1, 170.0, 162.9, 162.6, 161.6, 152.4, 140.4, 139.4, 116.7, 116.2, 111.2, 109.7, 107.1, 103.9, 63.6, 52.0, 24.9, 23.8, 14.6, 9.1, 7.7; HRMS *m/z* calcd. for C_21_H_24_O_7_Na ([M + Na]^+^) 411.1414, found 411.1406 (Figures S23–S25).

Data for *4-(ethoxycarbonyl)-3-hydroxy-2,5-dimethylphenyl 4-ethoxy-2-hydroxy-3,6-dimethylbenzoate* (**6i**): Yield: 40%, white solid, mp 187–190 °C; ^1^H NMR (400 MHz, CDCl_3_): *δ* 12.00 (s, 1H), 11.52 (s, 1H), 6.51 (s, 1H), 6.35 (s, 1H), 4.45 (q, *J* = 7.1 Hz, 2H), 4.12 (q, *J* = 7.0 Hz, 2H), 2.67 (s, 3H), 2.55 (s, 3H), 2.10 (s, 3H), 2.09 (s, 3H); 1.49–1.45 (m, 3H), 1.45–1.41 (m, 3H); ^13^C NMR (100 MHz, CDCl_3_): *δ* 171.8, 170.2, 163.1, 162.8, 161.7, 152.4, 140.6, 139.7, 116.9, 116.3, 111.4, 110.0, 107.3, 104.1, 63.8, 61.7, 25.0, 24.1, 14.8, 14.2, 9.3, 7.8; HRMS *m/z* calcd. for C_22_H_26_O_7_Na ([M + Na]^+^) 425.1570, found 425.1568 (Figures S26–S28).

Data for *3-hydroxy-2,5-dimethyl-4-(propoxycarbonyl)phenyl 4-ethoxy-2-hydroxy-3,6-dimethylbenzoate* (**6j**): Yield: 30%, white solid, mp 154–157 °C; ^1^H NMR (400 MHz, CDCl_3_): *δ* 12.04 (s, 1H), 11.52 (s, 1H), 6.51 (s, 1H), 6.36 (s, 1H), 4.36 (t, *J* = 6.6 Hz, 2H), 4.12 (q, *J* = 7.0 Hz, 2H), 2.67 (s, 3H), 2.56 (s, 3H), 2.10 (s, 3H), 2.09 (s, 3H), 1.83 (h, *J* = 7.2 Hz, 2H), 1.46 (t, *J* = 7.0 Hz, 3H), 1.05 (t, *J* = 7.4 Hz, 3H); ^13^C NMR (100 MHz, CDCl_3_): *δ* 172.0, 170.2, 163.1, 162.9, 161.7, 152.4, 140.6, 139.6, 116.9, 116.3, 111.4, 110.1, 107.3, 104.1, 67.5, 63.8, 25.0, 24.1, 21.9, 14.8, 10.7, 9.3, 7.8; HRMS *m/z* calcd. for C_23_H_28_O_7_Na ([M + Na]^+^) 439.1727, found 439.1721 (Figures S29–S31).

Data for *3-hydroxy-4-(isopropoxycarbonyl)-2,5-dimethylphenyl 4-ethoxy-2-hydroxy-3,6-dimethylbenzoate* (**6k**): Yield: 37%, white solid, mp 125–128 °C; ^1^H NMR (400 MHz, CDCl_3_): *δ* 12.05 (s, 1H), 11.53 (s, 1H), 6.50 (s, 1H), 6.36 (s, 1H), 5.38–5.29 (m, 1H), 4.12 (q, *J* = 7.0 Hz, 2H), 2.67 (s, 3H), 2.54 (s, 3H), 2.10 (s, 3H), 2.08 (s, 3H), 1.46 (t, *J* = 7.0 Hz, 3H), 1.41 (d, *J* = 6.3 Hz, 6H); ^13^C NMR (100 MHz, CDCl_3_): *δ* 171.3, 170.2, 163.0, 162.8, 161.7, 152.3, 140.5, 139.6, 116.9, 116.3, 111.4, 110.4, 107.2, 104.1, 77.3, 69.8, 63.8, 25.0, 24.2, 21.9, 14.8, 9.3, 7.8; HRMS *m/z* calcd. for C_23_H_28_O_7_Na ([M + Na]^+^) 439.1727, found 439.1722 (Figures S32–S34).

Data for *3-hydroxy-4-(isopropoxycarbonyl)-2,5-dimethylphenyl 4-ethoxy-2-hydroxy-3,6-dimethylbenzoate* (**6l**): Yield: 39%, white solid, mp 132–135 °C; ^1^H NMR (400 MHz, DMSO-*d*_6_): *δ* 10.76 (s, 1H), 10.49 (s, 1H), 6.73 (s, 1H), 6.59 (s, 1H), 6.10–6.00 (m, 1H), 5.46–5.40 (m, 1H), 5.30 (dd, *J* = 6.8, 2.0 Hz, 1H), 4.85 (d, *J* = 5.6 Hz, 2H), 4.13 (q, *J* = 6.8 Hz, 2H), 2.55 (s, 3H), 2.35 (s, 3H), 2.01 (s, 3H), 2.00 (s, 3H), 1.38 (t, *J* = 6.8 Hz, 3H); ^13^C NMR (100 MHz, DMSO-*d*_6_): *δ* 169.2, 160.9, 160.1, 157.6, 151.5, 139.4, 136.8, 132.7, 119.0, 118.5, 116.6, 116.3, 116.0, 110.6, 107.6, 107.2, 66.1, 64.1, 23.5, 21.4, 15.1, 9.8, 8.5; HRMS *m/z* calcd. for C_23_H_26_O_7_Na ([M + Na]^+^) 437.1570, found 437.1573 (Figures S35–S37).

Data for *4-((cyanomethoxy)carbonyl)-3-hydroxy-2,5-dimethylphenyl 4-ethoxy-2-hydroxy-3,6-dimethylbenzoate* (**6m**): Yield: 30%, white solid, mp 160–163 °C; ^1^H NMR (400 MHz, CDCl_3_): *δ* 11.44 (s, 1H), 11.29 (s, 1H), 6.57 (s, 1H), 6.36 (s, 1H), 5.02 (s, 2H), 4.13 (q, *J* = 7.0 Hz, 2H), 2.67 (s, 3H), 2.57 (s, 3H), 2.10 (s, 6H), 1.46 (t, *J* = 7.0 Hz, 3H); ^13^C NMR (100 MHz, CDCl_3_): *δ* 170.2, 170.0, 163.5, 163.1, 161.9, 153.6, 140.6, 139.8, 117.6, 117.1, 113.9, 111.4, 108.1, 107.3, 103.9, 63.8, 48.9, 25.1, 24.2, 14.8, 9.3, 7.9; HRMS *m/z* calcd. for C_22_H_23_NO_7_Na ([M + Na]^+^) 436.1366, found 436.1358 (Figures S38–S40).

Data for *benzyl 4-(4-ethoxy-2-hydroxy-3,6-dimethylbenzoyloxy)-2-hydroxy-3,6-dimethylbenzoate* (**6n**): Yield: 36%, white solid, mp 129–131 °C; ^1^H NMR (400 MHz, CDCl_3_): *δ* 11.93 (s, 1H), 11.52 (s, 1H), 7.47–7.33 (m, 5H), 6.51 (s, 1H), 6.36 (s, 1H), 5.43 (s, 2H), 4.12 (q, *J* = 6.8 Hz, 2H), 2.67 (s, 3H), 2.53 (s, 3H), 2.11 (s, 3H), 2.10 (s, 3H), 1.46 (t, *J* = 6.8 Hz, 3H); ^13^C NMR (100 MHz, CDCl_3_): *δ* 171.6, 170.2, 163.1, 163.0, 161.8, 152.6, 140.6, 139.8, 135.1, 128.7 × 2, 128.6, 128.5 × 2, 117.0, 116.4, 111.3, 109.8, 107.3, 104.1, 67.5, 63.8, 25.1, 24.4, 14.8, 9.3, 7.9; HRMS *m/z* calcd. for C_27_H_28_O_7_Na ([M + Na]^+^) 487.1727, found 487.1729 (Figures S41–S43).

Data for *3-hydroxy-4-(methoxycarbonyl)-2,5-dimethylphenyl 2-hydroxy-3,6-dimethyl-4-propoxybenzoate* (**6o**): Yield: 33%, white solid, mp 110–113 °C; ^1^H NMR (400 MHz, CDCl_3_): *δ* 11.91 (s, 1H), 11.50 (s, 1H), 6.51 (s, 1H), 6.34 (s, 1H), 4.00 (t, *J* = 6.4 Hz, 2H), 3.96 (s, 3H), 2.65 (s, 3H), 2.52 (s, 3H), 2.10 (s, 3H), 2.07 (s, 3H), 1.85 (p, *J* = 6.9 Hz, 2H), 1.06 (t, *J* = 7.4 Hz, 3H); ^13^C NMR (100 MHz, CDCl_3_): δ 172.3, 170.2, 163.0, 162.8, 161.9, 152.5, 140.6, 139.6, 116.9, 116.4, 111.4, 109.9, 107.3, 104.0, 69.7, 52.2, 25.0, 24.0, 22.6, 10.5, 9.3, 7.8; HRMS *m/z* calcd. for C_22_H_26_O_7_Na ([M + Na]^+^) 425.1570, found 425.1564 (Figures S44–S46).

Data for *4-(ethoxycarbonyl)-3-hydroxy-2,5-dimethylphenyl 2-hydroxy-3,6-dimethyl-4-propoxybenzoate* (**6p**): Yield: 44%, white solid, mp 160–163 °C; ^1^H NMR (400 MHz, CDCl_3_): *δ* 12.00 (s, 1H), 11.52 (s, 1H), 6.51 (s, 1H), 6.36 (s, 1H), 4.45 (q, *J* = 7.1 Hz, 2H), 4.01 (t, *J* = 6.4 Hz, 2H), 2.67 (s, 3H), 2.55 (s, 3H), 2.11 (s, 3H), 2.08 (s, 3H), 1.85 (h, *J* = 7.3 Hz, 2H), 1.43 (t, *J* = 7.1 Hz, 3H), 1.07 (t, *J* = 7.4 Hz, 3H); ^13^C NMR (100 MHz, CDCl_3_): *δ* 171.8, 170.2, 163.0, 162.8, 161.9, 152.4, 140.6, 139.7, 116.9, 116.3, 111.4, 110.0, 107.3, 104.1, 69.7, 61.7, 25.0, 24.1, 22.6, 14.2, 10.5, 9.3, 7.8; HRMS *m/z* calcd. for C_23_H_28_O_7_Na ([M + Na]^+^) 439.1727, found 439.1722 (Figures S47–S49).

Data for *3-hydroxy-2,5-dimethyl-4-(propoxycarbonyl)phenyl 2-hydroxy-3,6-dimethyl-4-propoxybenzoate* (**6q**): Yield: 31%, white solid, mp 126–130 °C; ^1^H NMR (400 MHz, CDCl_3_): *δ* 12.04 (s, 1H), 11.52 (s, 1H), 6.52 (s, 1H), 6.36 (s, 1H), 4.36 (t, *J* = 6.6 Hz, 2H), 4.01 (t, *J* = 6.4 Hz, 2H), 2.67 (s, 3H_3_), 2.56 (s, 3H), 2.11 (s, 3H), 2.09 (s, 3H), 1.90–1.77 (m, 4H), 1.06 (q, *J* = 7.9 Hz, 6H); ^13^C NMR (100 MHz, CDCl_3_): *δ* 172.0, 170.2, 163.0, 162.9, 161.9, 152.4, 140.6, 139.6, 116.9, 116.3, 111.4, 110.1, 107.3, 104.1, 69.7, 67.5, 25.0, 24.1, 22.6, 21.9, 10.7, 10.5, 9.3, 7.8; HRMS *m/z* calcd. for C_24_H_30_O_7_Na ([M + Na]^+^) 453.1883, found 453.1877 (Figures S50–S52).

Data for *allyl 2-hydroxy-4-(2-hydroxy-3,6-dimethyl-4-propoxybenzoyloxy)-3,6-dimethylbenzoate* (**6r**): Yield: 33%, white solid, mp 98–111 °C; ^1^H NMR (400 MHz, CDCl_3_): *δ* 11.90 (s, 1H), 11.51 (s, 1H), 6.52 (s, 1H), 6.36 (s, 1H), 6.10–5.98 (m, 1H), 5.42 (dd, *J* = 17.2, 1.3 Hz, 1H), 5.33 (dd, *J* = 10.4, 1.1 Hz, 1H), 4.89 (d, *J* = 5.8 Hz, 2H), 4.02 (t, *J* = 6.4 Hz, 2H), 2.67 (s, 3H), 2.56 (s, 3H), 2.11 (s, 3H), 2.09 (s, 3H), 1.85 (h, *J* = 7.3 Hz, 2H), 1.07 (t, *J* = 7.4 Hz, 3H); ^13^C NMR (100 MHz, CDCl_3_): *δ* 171.5, 170.2, 163.0, 162.9, 161.9, 152.6, 140.6, 139.7, 131.4, 119.2, 117.0, 116.4, 111.4, 109.9, 107.3, 104.0, 69.7, 66.3, 25.0, 24.2, 22.6, 10.5, 9.3, 7.8; HRMS *m/z* calcd. for C_24_H_28_O_7_Na ([M + Na]^+^) 451.1727, found 451.1726 (Figures S53–S55).

Data for *4-((cyanomethoxy)carbonyl)-3-hydroxy-2,5-dimethylphenyl 2-hydroxy-3,6-dimethyl-4-propoxybenzoate* (**6s**): Yield: 28%, white solid, mp 135–138 °C; ^1^H NMR (400 MHz, CDCl_3_): *δ* 11.44 (s, 1H), 11.29 (s, 1H), 6.57 (s, 1H), 6.36 (s, 1H), 5.02 (s, 2H), 4.02 (t, *J* = 6.4 Hz, 2H), 2.67 (s, 3H), 2.57 (s, 3H), 2.11 (s, 3H), 2.10 (s, 3H), 1.86 (q, *J* = 6.7 Hz, 2H), 1.08 (t, *J* = 7.4 Hz, 3H); ^13^C NMR (100 MHz, CDCl_3_): *δ* 170.2, 170.0, 163.4, 163.1, 162.0, 153.7, 140.6, 139.8, 117.6, 117.1, 113.8, 111.5, 108.2, 107.4, 103.9, 69.7, 48.9, 25.0, 24.2, 22.6, 10.5, 9.3, 7.8; HRMS *m/z* calcd. for C_23_H_25_NO_7_Na ([M + Na]^+^) 450.1523, found 450.1512 (Figures S56–S58).

Data for *3-hydroxy-4-(isopropoxycarbonyl)-2,5-dimethylphenyl 2-hydroxy-3,6-dimethyl-4-propoxybenzoate* (**6t**): Yield: 37%, white solid, mp 112–115 °C; ^1^H NMR (400 MHz, CDCl_3_): *δ* 12.05 (s, 1H), 11.53 (s, 1H), 6.50 (s, 1H), 6.36 (s, 1H), 5.38–5.29 (m, 1H), 4.01 (t, *J* = 6.4 Hz, 2H), 2.67 (s, 3H), 2.54 (s, 3H), 2.11 (s, 3H), 2.08 (s, 3H), 1.89–1.80 (m, 2H), 1.42 (s, 3H), 1.41 (d, *J* = 6.3 Hz, 6H), 1.07 (t, *J* = 7.2 Hz, 3H); ^13^C NMR (100 MHz, CDCl_3_): *δ* 171.3, 170.3, 163.0, 162.8, 161.9, 152.3, 140.6, 139.6, 116.9, 116.3, 111.4, 110.4, 107.3, 104.1, 69.8, 69.7, 25.0, 24.2, 22.6, 21.9 × 2, 10.5, 9.3, 7.8; HRMS *m/z* calcd. for C_24_H_30_O_7_Na ([M + Na]^+^) 453.1883, found 453.1883 (Figures S59–S61).

Data for *benzyl 2-hydroxy-4-(2-hydroxy-3,6-dimethyl-4-propoxybenzoyloxy)-3,6-dimethylbenzoate* (**6u**): Yield: 30%, white solid, mp 111–114 °C; ^1^H NMR (400 MHz, CDCl_3_): *δ* 11.92 (s, 1H), 11.51 (s, 1H), 7.51–7.29 (m, 5H), 6.51 (s, 1H), 6.36 (s, 1H), 5.43 (s, 2H), 4.02 (t, *J* = 6.4 Hz, 2H), 2.67 (s, 3H), 2.52 (s, 3H), 2.11 (s, 3H), 2.09 (s, 3H), 1.86 (q, *J* = 6.5 Hz, 2H), 1.07 (t, *J* = 7.4 Hz, 3H); ^13^C NMR (100 MHz, CDCl_3_): *δ* 171.6, 170.2, 163.0, 163.0, 161.9, 152.6, 140.6, 139.7, 135.1, 128.7 × 2, 128.6, 128.4 × 2, 117.0, 116.4, 111.4, 109.9, 107.3, 104.1, 69.7, 67.4, 25.0, 24.3, 22.6, 10.5, 9.3, 7.8; HRMS *m/z* calcd. for C_28_H_30_O_7_Na ([M + Na]^+^) 501.1883, found 501.1880 (Figures S62–S64).

Data for *3-hydroxy-4-(methoxycarbonyl)-2,5-dimethylphenyl 2-hydroxy-4-isopropoxy-3,6-dimethylbenzoate* (**6v**): Yield: 36%, white solid, mp 60–63 °C; ^1^H NMR (400 MHz, CDCl_3_): *δ* 11.92 (s, 1H), 11.53 (s, 1H), 6.51 (s, 1H), 6.37 (s, 1H), 4.67 (p, *J* = 6.1 Hz, 1H), 3.97 (s, 3H), 2.66 (s, 3H), 2.53 (s, 3H), 2.09 (s, 6H), 1.38 (d, *J* = 6.0 Hz, 6H); ^13^C NMR (100 MHz, CDCl_3_): *δ* 172.3, 170.2, 163.4, 162.8, 161.0, 152.6, 140.3, 139.6, 116.9, 116.4, 112.3, 109.9, 108.6, 103.8, 70.3, 52.2, 25.1, 24.0, 22.2 × 2, 9.3, 8.0; HRMS *m/z* calcd. for C_22_H_26_O_7_Na ([M + Na]^+^) 425.1570, found 425.1568 (Figures S65–S67).

Data for *4-(ethoxycarbonyl)-3-hydroxy-2,5-dimethylphenyl 2-hydroxy-4-isopropoxy-3,6-dimethylbenzoate* (**6w**): Yield: 44%, white solid, mp 62–65 °C; ^1^H NMR (400 MHz, CDCl_3_): *δ* 12.00 (s, 1H), 11.54 (s, 1H), 6.51 (s, 1H), 6.37 (s, 1H), 4.67 (p, *J* = 6.1 Hz, 1H), 4.45 (q, *J* = 7.1 Hz, 2H), 2.66 (s, 3H), 2.55 (s, 3H), 2.09 (s, 6H), 1.43 (t, *J* = 7.1 Hz, 3H), 1.38 (d, *J* = 6.0 Hz, 6H); ^13^C NMR (100 MHz, CDCl_3_): *δ* 171.8, 170.2, 163.4, 162.8, 161.0, 152.4, 140.3, 139.7, 116.9, 116.3, 112.3, 110.0, 108.6, 103.8, 70.3, 61.7, 25.1, 24.1, 22.2 × 2, 14.2, 9.3, 8.0; HRMS *m/z* calcd. for C_23_H_28_O_7_Na ([M + Na]^+^) 439.1727, found 439.1721 (Figures S68–S70).

Data for *3-hydroxy-2,5-dimethyl-4-(propoxycarbonyl)phenyl 2-hydroxy-4-isopropoxy-3,6-dimethylbenzoate* (**6x**): Yield: 36%, white solid, mp 117–120 °C; ^1^H NMR (400 MHz, CDCl_3_): *δ* 12.04 (s, 1H), 11.54 (s, 1H), 6.51 (s, 1H), 6.37 (s, 1H), 4.67 (p, *J* = 6.1 Hz, 1H), 4.36 (t, *J* = 6.6 Hz, 2H), 2.66 (s, 3H), 2.56 (s, 3H), 2.09 (s, 6H), 1.83 (h, *J* = 7.1 Hz, 2H), 1.38 (d, *J* = 6.0 Hz, 6H), 1.05 (t, *J* = 7.4 Hz, 3H); ^13^C NMR (100 MHz, CDCl_3_): *δ* 172.0, 170.2, 163.4, 162.9, 161.0, 152.4, 140.3, 139.6, 116.9, 116.3, 112.3, 110.1, 108.6, 103.8, 70.3, 67.5, 25.1, 24.1, 22.2 × 2, 21.9, 10.7, 9.3, 8.0; HRMS *m/z* calcd. for C_24_H_30_O_7_Na ([M + Na]^+^) 453.1883, found 453.1877 (Figures S71–S73).

Data for *3-hydroxy-4-(isopropoxycarbonyl)-2,5-dimethylphenyl 2-hydroxy-4-isopropoxy-3,6-dimethylbenzoate* (**6y**): Yield: 35%, white solid, mp 140–143 °C; ^1^H NMR (400 MHz, CDCl_3_): *δ* 12.05 (s, 1H), 11.55 (s, 1H), 6.50 (s, 1H), 6.37 (s, 1H), 5.34 (p, *J* = 6.3 Hz, 1H), 4.67 (p, *J* = 6.1 Hz, 1H), 2.66 (s, 3H), 2.54 (s, 3H), 2.09 (s, 6H), 1.41 (d, *J* = 6.3 Hz, 6H), 1.38 (d, *J* = 6.0 Hz, 6H); ^13^C NMR (100 MHz, CDCl_3_): *δ* 171.3, 170.2, 163.4, 162.8, 161.0, 152.3, 140.3, 139.6, 116.9, 116.3, 112.3, 110.3, 108.5, 103.8, 70.3, 69.8, 25.0, 24.2, 22.2 × 2, 21.9 × 2, 9.3, 8.0; HRMS *m/z* calcd. for C_24_H_30_O_7_Na ([M + Na]^+^) 453.1883, found 453.1879 (Figures S74–S76).

Data for *allyl 2-hydroxy-4-(2-hydroxy-4-isopropoxy-3,6-dimethylbenzoyloxy)-3,6-dimethylbenzoate* (**6z**): Yield: 34%, white solid, mp 111–114 °C; ^1^H NMR (400 MHz, CDCl_3_): *δ* 11.90 (s, 1H), 11.53 (s, 1H), 6.52 (s, 1H), 6.37 (s, 1H), 6.09–5.99 (m, 1H), 5.46–5.37 (m, 1H), 5.36–5.30 (m, 1H), 4.89 (d, *J* = 5.8 Hz, 2H), 4.67 (p, *J* = 6.0 Hz, 1H), 2.66 (s, 3H), 2.56 (s, 3H), 2.09 (s, 6H), 1.38 (d, *J* = 6.0 Hz, 6H); ^13^C NMR (100 MHz, CDCl_3_): *δ* 171.5, 170.2, 163.4, 162.9, 161.0, 152.6, 140.3, 139.7, 131.4, 119.2, 117.0, 116.4, 112.3, 109.9, 108.6, 103.8, 70.3, 66.3, 25.1, 24.2, 22.2, 9.3, 8.0; HRMS *m/z* calcd. for C_24_H_28_O_7_Na ([M + Na]^+^) 451.1727, found 451.1725 (Figures S77–S79).

Data for *4-((cyanomethoxy)carbonyl)-3-hydroxy-2,5-dimethylphenyl 2-hydroxy-4-isopropoxy-3,6-dimethylbenzoate* (**6a′**): Yield: 36%, white solid, mp 155–158 °C; ^1^H NMR (400 MHz, CDCl_3_): *δ* 11.46 (s, 1H), 11.28 (s, 1H), 6.57 (s, 1H), 6.37 (s, 1H), 5.01 (s, 2H), 4.67 (p, *J* = 6.0 Hz, 1H), 2.66 (s, 3H), 2.57 (s, 3H), 2.10 (s, 3H), 2.09 (s, 3H), 1.38 (d, *J* = 6.0 Hz, 6H); ^13^C NMR (100 MHz, CDCl_3_): *δ* 170.2, 170.0, 163.4 × 2, 161.1, 153.7, 140.3, 139.8, 117.6, 117.2, 113.8, 112.4, 108.6, 108.1, 103.6, 70.4, 48.9, 25.1, 24.2, 22.2 × 2, 9.3, 8.0; HRMS *m/z* calcd. for C_23_H_25_NO_7_Na ([M + Na]^+^) 450.1523, found 450.1509 (Figures S80–S82).

Data for *benzyl 2-hydroxy-4-(2-hydroxy-4-isopropoxy-3,6-dimethylbenzoyloxy)-3,6-dimethylbenzoate* (**6b′**): Yield: 40%, white solid, mp 83–86 °C; ^1^H NMR (400 MHz, CDCl_3_): *δ* 11.91 (s, 1H), 11.53 (s, 1H), 7.47–7.33 (m, 5H), 6.50 (s, 1H), 6.36 (s, 1H), 5.42 (s, 2H), 4.66 (p, *J* = 6.1 Hz, 1H), 2.65 (s, 3H), 2.51 (s, 3H), 2.08 (s, 6H), 1.37 (d, *J* = 6.0 Hz, 6H); ^13^C NMR (100 MHz, CDCl_3_): *δ* 171.6, 170.2, 163.4, 163.0, 161.0, 152.6, 140.3, 139.7, 135.1, 128.7 × 2, 128.6, 128.4 × 2, 117.0, 116.4, 112.3, 109.8, 108.6, 103.8, 70.3, 67.5, 25.1, 24.3, 22.2 × 2, 9.3, 8.0; HRMS *m/z* calcd. for C_28_H_30_O_7_Na ([M + Na]^+^) 501.1883, found 501.1883 (Figures S83–S85).

Data for *benzyl 3-hydroxy-4-(methoxycarbonyl)-2,5-dimethylphenyl 4-(allyloxy)-2-hydroxy-3,6-dimethylbenzoate* (**6c′**): Yield: 27%, white solid, mp 138–141 °C; ^1^H NMR (400 MHz, DMSO-*d*_6_): *δ* 10.75 (s, 1H), 10.55 (s, 1H), 6.72 (s, 1H), 6.60 (s, 1H), 6.12–6.03 (m, 1H), 5.46–5.40 (m, 1H), 5.32–5.26 (m, 1H), 4.68 (d, *J* = 5.0 Hz, 2H), 3.89 (s, 3H), 2.55 (s, 3H), 2.35 (s, 3H), 2.04 (s, 3H), 2.01 (s, 3H); ^13^C NMR (100 MHz, DMSO-*d*_6_): *δ* 170.1, 169.1, 160.4, 160.0, 157.8, 151.5, 139.2, 137.0, 133.8, 117.7, 116.6, 116.3, 115.7, 110.8, 107.8, 107.6, 68.9, 52.8, 23.5, 21.5, 9.8, 8.6; HRMS *m/z* calcd. for C_22_H_24_O_7_Na ([M + Na]^+^) 423.1414, found 423.1407 (Figures S86–S88).

Data for *4-(ethoxycarbonyl)-3-hydroxy-2,5-dimethylphenyl 4-(allyloxy)-2-hydroxy-3,6-dimethylbenzoate* (**6d′**): Yield: 32%, white solid, mp 116–120 °C; ^1^H NMR (400 MHz, DMSO-*d*_6_): *δ* 10.73 (s, 1H), 10.68 (s, 1H), 6.71 (s, 1H), 6.60 (s, 1H), 6.12–6.02 (m, 1H), 5.42 (d, *J* = 17.2 Hz, 1H), 5.29 (d, *J* = 11.9 Hz, 1H), 4.67 (d, *J* = 4.9 Hz, 2H), 4.36 (q, *J* = 7.1 Hz, 2H), 2.54 (s, 3H), 2.37 (s, 3H), 2.04 (s, 3H), 2.00 (s, 3H), 1.34 (t, *J* = 7.1 Hz, 3H); ^13^C NMR (100 MHz, CDCl_3_): *δ* 171.8, 170.2, 163.1, 162.8, 161.2, 152.4, 140.6, 139.7, 132.8, 117.5, 116.9, 116.3, 111.6, 110.1, 107.5, 104.4, 68.7, 61.7, 25.1, 24.1, 14.2, 9.3, 7.9; HRMS *m/z* calcd. for C_23_H_26_O_7_Na ([M + Na]^+^) 437.1570, found 437.1572 (Figures S89–S91).

Data for *3-hydroxy-2,5-dimethyl-4-(propoxycarbonyl)phenyl 4-(allyloxy)-2-hydroxy-3,6-dimethylbenzoate* (**6e′**): Yield: 33%, white solid, mp 122–125 °C; ^1^H NMR (400 MHz, CDCl_3_): *δ* 11.90 (s, 1H), 11.51 (s, 1H), 6.52 (s, 1H), 6.36 (s, 1H), 6.11–5.98 (m, 1H), 5.43 (d, *J* = 17.2 Hz, 1H), 5.33 (d, *J* = 10.4 Hz, 1H), 4.89 (d, *J* = 7.0 Hz, 2H), 4.01 (t, *J* = 6.4 Hz, 2H), 2.67 (s, 3H), 2.56 (s, 3H), 2.11 (s, 3H), 2.09 (s, 3H), 1.84 (p, *J* = 6.9 Hz, 2H), 1.07 (t, *J* = 7.4 Hz, 3H); ^13^C NMR (100 MHz, CDCl_3_): *δ* 171.5, 170.2, 163.0, 162.9, 161.9, 152.6, 140.6, 139.7, 131.4, 119.2, 117.0, 116.4, 111.4, 109.9, 107.3, 104.0, 69.7, 66.3, 25.0, 24.2, 22.6, 10.5, 9.3, 7.8; HRMS *m/z* calcd. for C_24_H_28_O_7_Na ([M + Na]^+^) 451.1727, found 451.1721 (Figures S92–S94).

Data for *3-hydroxy-2,5-dimethyl-4-(propoxycarbonyl)phenyl 4-(allyloxy)-2-hydroxy-3,6-dimethylbenzoate* (**6f′**): Yield: 37%, white solid, mp 124–127 °C; ^1^H NMR (400 MHz, CDCl_3_): *δ* 12.05 (s, 1H), 11.54 (s, 1H), 6.50 (s, 1H), 6.36 (s, 1H), 6.12–6.02 (m, 1H), 5.49–5.41 (m, 1H), 5.37–5.29 (m, 2H), 4.63 (t, *J* = 5.0, 2H), 2.67 (s, 3H), 2.54 (s, 3H), 2.14 (s, 3H), 2.08 (s, 3H), 1.41 (d, *J* = 6.3 Hz, 6H); ^13^C NMR (100 MHz, CDCl_3_): *δ* 171.3, 170.2, 163.1, 162.8, 161.2, 152.3, 140.5, 139.6, 132.8, 117.4, 116.9, 116.2, 111.6, 110.4, 107.5, 104.4, 69.8, 68.7, 25.0, 24.2, 21.9 × 2, 9.3, 7.9; HRMS *m/z* calcd. for C_24_H_28_O_7_Na ([M + Na]^+^) 451.1727, found 451.1721 (Figures S95–S97).

Data for *allyl 4-(4-(allyloxy)-2-hydroxy-3,6-dimethylbenzoyloxy)-2-hydroxy-3,6-dimethylbenzoate* (**6g′**): Yield: 37%, white solid, mp 110–113 °C; ^1^H NMR (400 MHz, DMSO-*d*_6_): *δ* 10.76 (s, 1H), 10.54 (s, 1H), 6.73 (s, 1H), 6.60 (s, 1H), 6.12–6.00 (m, 2H), 5.43 (d, *J* = 18.8 Hz, 2H), 5.32–5.27 (m, 2H), 4.85 (d, *J* = 5.5 Hz, 2H), 4.68 (d, *J* = 4.9 Hz, 2H), 2.55 (s, 3H), 2.36 (s, 3H), 2.04 (s, 3H), 2.01 (s, 3H); ^13^C NMR (100 MHz, DMSO-*d*_6_): *δ* 169.3, 169.1, 160.4, 160.0, 157.7, 151.5, 139.2, 136.8, 133.8, 132.7, 119.0, 117.7, 116.6, 116.3, 115.9, 110.8, 107.8, 107.6, 68.9, 66.1, 23.5, 21.5, 9.8, 8.6; HRMS *m/z* calcd. for C_24_H_26_O_7_Na ([M + Na]^+^) 449.1570, found 449.1561 (Figures S98–S100).

Data for *allyl 4-((cyanomethoxy)carbonyl)-3-hydroxy-2,5-dimethylphenyl 4-(allyloxy)-2-hydroxy-3,6-dimethylbenzoate* (**6h′**): Yield: 31%, white solid, mp 124–127 °C; ^1^H NMR (400 MHz, CDCl_3_): *δ* 11.45 (s, 1H), 11.29 (s, 1H), 6.57 (s, 1H), 6.36 (s, 1H), 6.12–6.08 (m, 1H), 5.49–5.36 (m, 1H), 5.35–5.28 (m, 1H), 5.02 (s, 2H), 4.64 (d, *J* = 5.0 Hz, 2H), 2.66 (s, 3H), 2.57 (s, 3H), 2.14 (s, 3H), 2.10 (s, 3H); ^13^C NMR (100 MHz, CDCl_3_): *δ* 170.2, 170.0, 163.5, 163.2, 161.4, 153.6, 140.6, 139.8, 132.7, 117.6, 117.5, 117.1, 113.8, 111.7, 108.2, 107.6, 104.2, 68.8, 48.9, 25.1, 24.2, 9.3, 7.9; HRMS *m/z* calcd. for C_23_H_23_NO_7_Na ([M + Na]^+^) 448.1366, found 448.1354 (Figures S101–S103).

Data for *benzyl 4-(4-(allyloxy)-2-hydroxy-3,6-dimethylbenzoyloxy)-2-hydroxy-3,6-dimethylbenzoate* (**6i′**): Yield: 44%, white solid, mp 75–78 °C; ^1^H NMR (400 MHz, CDCl_3_): *δ* 11.91 (s, 1H), 11.51 (s, 1H), 7.46–7.33 (m, 5H), 6.50 (s, 1H), 6.35 (s, 1H), 6.11–6.02 (m, 1H,), 5.47 (q, *J* = 1.6 Hz, 1H), 5.43 (s, 2H), 5.32 (dq, *J* = 10.5, 1.5 Hz, 1H), 4.62 (s, 2H), 2.66 (s, 3H), 2.52 (s, 3H), 2.13 (s, 3H), 2.08 (s, 3H); ^13^C NMR (100 MHz, CDCl_3_): *δ* 171.6, 170.1, 163.1, 163.0, 161.3, 152.6, 140.5, 139.7, 135.0, 132.8, 128.7 × 2, 128.6, 128.4 × 2, 117.5, 117.0, 116.4, 111.6, 109.9, 107.5, 104.4, 68.7, 67.5, 25.0, 24.3, 9.3, 7.9; HRMS *m/z* calcd. for C_28_H_28_NO_7_Na ([M + Na]^+^) 499.1727, found 499.1652 (Figures S104–S106).

Data for *3-hydroxy-4-(methoxycarbonyl)-2,5-dimethylphenyl 4-(benzyloxy)-2-hydroxy-3,6-dimethylbenzoate* (**6j′**): Yield: 35%, white solid, mp 131–134 °C; ^1^H NMR (400 MHz, CDCl_3_): *δ* 11.93 (s, 1H), 11.54 (s, 1H), 7.48–7.33 (m, 5H), 6.52 (s, 1H), 6.45 (s, 1H), 5.18 (s, 2H), 3.98 (s, 3H), 2.67 (s, 3H), 2.54 (s, 3H), 2.18 (s, 3H), 2.09 (s, 3H); ^13^C NMR (100 MHz, CDCl_3_): *δ* 172.7, 170.6, 163.6, 163.3, 161.8, 152.9, 141.1, 140.1, 137.1, 129.1 × 2, 128.4, 127.5 × 2, 117.3, 116.8, 112.2, 110.4, 108.1, 105.0, 70.4, 52.7, 25.5, 24.4, 9.7, 8.5; HRMS *m/z* calcd. for C_26_H_26_O_7_Na ([M + Na]^+^) 473.1570, found 473.1569 (Figures S107–S109).

Data for *4-(ethoxycarbonyl)-3-hydroxy-2,5-dimethylphenyl 4-(benzyloxy)-2-hydroxy-3,6-dimethylbenzoate* (**6k′**): Yield: 40%, white solid, mp 123–126 °C; ^1^H NMR (400 MHz, CDCl_3_): *δ* 12.00 (s, 1H), 11.54 (s, 1H), 7.47–7.32 (m, 5H), 6.51 (s, 1H), 6.44 (s, 1H), 5.17 (s, 2H), 4.45 (q, *J* = 7.1 Hz, 2H), 2.67 (s, 3H), 2.55 (s, 3H), 2.17 (s, 3H), 2.08 (s, 3H), 1.43 (t, *J* = 7.1 Hz, 3H); ^13^C NMR (100 MHz, CDCl_3_): *δ* 171.8, 170.2, 163.1, 162.8, 161.4, 152.4, 140.6, 139.7, 136.6, 128.6 × 2, 128.0, 127.0 × 2, 116.9, 116.3, 111.8, 110.1, 107.6, 104.5, 69.9, 61.7, 25.1, 24.1, 14.2, 9.3, 8.1; HRMS *m/z* calcd. for C_27_H_28_O_7_Na ([M + Na]^+^) 487.1727, found 487.1721 (Figures S110–S112).

Data for *3-hydroxy-2,5-dimethyl-4-(propoxycarbonyl)phenyl 4-(benzyloxy)-2-hydroxy-3,6-dimethylbenzoate* (**6l′**): Yield: 27%, white solid, mp 115–118 °C; ^1^H NMR (400 MHz, CDCl_3_): *δ* 12.04 (s, 1H), 11.54 (s, 1H), 7.47–7.32 (m, 5H), 6.51 (s, 1Hr), 6.44 (s, 1H), 5.17 (s, 2H), 4.36 (t, *J* = 6.6 Hz, 2H), 2.67 (s, 3H), 2.56 (s, 3H), 2.17 (s, 3H), 2.09 (s, 3H), 1.88–1.77 (m, 2H), 1.05 (t, *J* = 7.4 Hz, 3H); ^13^C NMR (100 MHz, CDCl_3_): *δ* 172.0, 170.2, 163.1, 162.9, 161.4, 152.4, 140.6, 139.6, 136.6, 128.6 × 2, 128.0, 127.0 × 2, 116.9, 116.3, 111.8, 110.1, 107.6, 104.5, 69.9, 67.5, 25.1, 24.1, 21.9, 10.7, 9.3, 8.1; HRMS *m/z* calcd. for C_28_H_30_O_7_Na ([M + Na]^+^) 501.1883, found 501.1881 (Figures S113–S115).

Data for *3-hydroxy-4-(isopropoxycarbonyl)-2,5-dimethylphenyl 4-(benzyloxy)-2-hydroxy-3,6-dimethylbenzoate* (**6m′**): Yield: 33%, white solid, mp 135–138 °C; ^1^H NMR (400 MHz, CDCl_3_): *δ* 12.05 (s, 1H), 11.55 (s, 1H), 7.47–7.32 (m, 5H), 6.50 (s, 1H), 6.44 (s, 1H), 5.33 (h, *J* = 6.2 Hz, 1H), 5.17 (s, 2H), 2.67 (s, 3H), 2.54 (s, 3H), 2.17 (s, 3H), 2.08 (s, 3H), 1.42 (s, 3H), 1.41 (s, 3H); ^13^C NMR (100 MHz, CDCl_3_): *δ* 171.3, 170.2, 163.1, 162.8, 161.4, 152.3, 140.6, 139.6, 136.6, 128.6 × 2, 128.0, 127.0 × 2, 116.9, 116.2, 111.8, 110.4, 107.6, 104.6, 69.9, 69.8, 25.1, 24.2, 21.9 × 2, 9.3, 8.1; HRMS *m/z* calcd. for C_28_H_30_O_7_Na ([M + Na]^+^) 501.1883, found 501.1882 (Figures S116–S118).

Data for *allyl 4-(4-(benzyloxy)-2-hydroxy-3,6-dimethylbenzoyloxy)-2-hydroxy-3,6-dimethylbenzoate* (**6n′**): Yield: 36%, white solid, mp 127–130 °C; ^1^H NMR (400 MHz, DMSO-*d*_6_): *δ* 9.89 (s, 1H), 9.67 (s, 1H), 6.64–6.47 (m, 5H), 5.86 (d, *J* = 5.0 Hz, 2H), 5.20 (dq, *J* = 16.4, 1H), 4.58 (d, *J* = 18.3 Hz, 1H), 4.45 (d, *J* = 10.5 Hz, 1H), 4.37 (s, 2H), 3.99 (d, *J* = 5.6 Hz, 2H), 1.69 (s, 3H), 1.50 (s, 3H), 1.21 (s, 3H), 1.16 (s, 3H, CH_3_); ^13^C NMR (100 MHz, DMSO-*d*_6_): *δ* 169.3, 169.1, 160.6, 159.9, 157.8, 151.5, 139.1, 137.3, 136.9, 132.7, 128.9 × 2, 128.3, 127.8 × 2, 119.0, 116.6, 116.3, 115.9, 111.0, 108.0, 107.8, 66.9, 66.2, 23.4, 21.5, 9.8, 8.7; HRMS *m/z* calcd. for C_28_H_28_O_7_Na ([M + Na]^+^) 499.1727, found 499.1727 (Figures S119–S121).

Data for *4-((cyanomethoxy)carbonyl)-3-hydroxy-2,5-dimethylphenyl 4-(benzyloxy)-2-hydroxy-3,6-dimethylbenzoate* (**6o′**): Yield: 28%, white solid, mp 151–155 °C; ^1^H NMR (400 MHz, DMSO-*d*_6_): *δ* 10.74 (s, 1H), 9.96 (s, 1H), 7.51–7.32 (m, 5H), 6.75 (s, 1H), 6.72 (s, 1H), 5.23 (s, 2H), 5.22 (s, 2H), 2.55 (s, 3H), 2.30 (s, 3H), 2.07 (s, 3H), 2.02 (s, 3H); ^13^C NMR (100 MHz, DMSO-*d*_6_): *δ* 169.1, 167.5, 160.6, 159.9, 156.0, 151.5, 139.1, 137.3, 136.0, 128.9 × 2, 128.3, 127.8 × 2, 116.9, 116.8, 116.3, 116.2, 111.0, 108.0, 107.8, 69.9, 50.1, 23.4, 20.3, 9.9, 8.7; HRMS *m/z* calcd. for C_27_H_25_NO_7_Na ([M + Na]^+^) 498.1523, found 498.1516 (Figures S122–S124).

Data for *benzyl 4-(4-(benzyloxy)-2-hydroxy-3,6-dimethylbenzoyloxy)-2-hydroxy-3,6-dimethylbenzoate* (**6p′**): Yield: 27%, white solid, mp 153–157 °C; ^1^H NMR (400 MHz, CDCl_3_): *δ* 11.92 (s, 1H), 11.53 (s, 1H), 7.47–7.30 (m, 10H), 6.50 (s, 1H), 6.44 (s, 1H), 5.42 (s, 2H), 5.16 (s, 2H), 2.66 (s, 3H), 2.51 (s, 3H), 2.17 (s, 3H), 2.08 (s, 3H); ^13^C NMR (100 MHz, CDCl_3_): *δ* 170.3, 168.8, 161.8, 161.7, 160.1, 151.3, 139.3, 138.5, 135.3, 133.7, 127.4 × 2, 127.3 × 2, 127.3, 127.1 × 2, 126.7, 125.7 × 2, 115.7, 115.1, 110.5, 108.6, 106.3, 103.2, 68.6, 66.2, 23.8, 23.0, 8.0, 6.8; HRMS *m/z* calcd. for C_32_H_30_O_7_Na ([M + Na]^+^) 549.1883, found 549.1883 (Figures S125–S127).

### 3.2. In Vitro Cytotoxicity Assay

The MTT assay was used to evaluate the cytotoxicity of the target compounds on DMCK cells. Logarithmic-growth-phase DMCK cells were seeded in a 96-well plate (10^4^ cells/well) and incubated in a cell culture incubator for 24 h. The culture medium was then removed and replaced with a drug-containing medium for the treatment groups (**1**, **6a**–**6p′**, 100 μmol/L), normal saline for the control group, or hydrochlorothiazide (100 μmol/L) for the positive control group. Each well was treated with 100 µL of the respective solution and incubated for an additional 24 h. The culture medium was then removed and replaced with a DMEM solution containing 20 µL of MTT (5 mg/mL). After incubating for 4 h, the liquid in each well was removed and replaced with 150 µL of DMSO, which was shaken for 10 min to ensure thorough mixing. The absorbance at 490 nm was measured to determine the optical density (OD) of each well. Cell growth inhibition rate (%) = (OD_Blank_ − OD_Experimental_)/OD_Blank_ × 100%.

### 3.3. In Vitro Diuretic Activity Assay

Log-phase MDCK cells were seeded in the upper chambers of Transwell plates (4 × 10^4^ cells/well), and 800 µL of complete culture medium was added to the lower chambers. After 24 h of incubation at 37 °C, the electrical resistance of the upper chamber cells was measured one by one (R = (R_cell_ − R_blank_) × 0.04π). When the electrical resistance of the upper chamber cells reached ≥300 Ω cm^2^, the upper chamber medium was removed and replaced with the drug solution. The blank group (given normal saline), the experimental group (**1**, **6a**–**6p′**, 100 μmol/L), and the hydrochlorothiazide group (100 μmol/L) were set, and each well was treated with 100 µL of the corresponding solution. The cells were then further incubated for 24 h. After that, the upper and lower chamber fluids were removed, and 200 µL of NaCl solution (15 mg/mL) was added to the upper chamber, while 800 µL of DMEM was added to the lower chamber for continued incubation. At 0.5, 1, 2, and 3 h, 50 µL of the lower chamber fluid was taken and the OD values were measured using Na^+^ and Cl^−^ detection kits. The transport inhibition rate (%) was calculated as follows: transport inhibition rate (%) = (OD_blank_ − OD_experimental_)/OD_blank_ × 100%.

### 3.4. In Vitro Litholytic Activity Assay

Logarithmic-phase DMCK cells were inoculated in the upper chamber of a Transwell plate (4 × 10^4^ cells/well). Then, 800 µL of complete culture medium was added to the lower chamber, and the cells were incubated for 24 h until they adhered to the wall. The resistance value of the upper chamber cells was measured one by one at 37 °C (R = (R_cell_ − R_blank_) × 0.04π). When the resistance value of the upper chamber cells was ≥300 Ω cm^2^, the upper chamber liquid was discarded for drug administration. A control group (given physiological saline) and an experimental group (**1**, **6a**–**6p′**, 100 μmol/L) were set up, and 100 µL of each drug was administered per well, followed by continued incubation for 24 h. Then, the upper and lower chamber liquids were discarded, and 200 µL of CaC_2_O_4_ (30 mmol/L) solution was added to the upper chamber, and 800 µL of PBS solution was added to the lower chamber for continued incubation. At 0.5, 1, 2, and 3 h, 50 µL of the lower chamber liquid was taken and then tested using the Ca^2+^ and C_2_O_4_^2−^ detection kits and the following detection methods. The transport inhibition rate (%) = (OD_blank_ − OD_experimental_)/OD_blank_ × 100%.

### 3.5. Molecular Docking Study

The crystal structure of the WNK1 kinase domain in complex with WNK463 (PDB ID 5DRB) [26] and the calcium-sensing receptor (CaSR) in complex with cinacalcet (PDB ID 7m3f) [27] were selected to perform the molecular docking studies. All the structures were prepared using Schrodinger’s LigPrep program [28]. The receptor grids of the WNK1 kinase domain and CaSR were defined by WNK463 and cinacalcet, respectively. The center and size of the receptor grid of the WNK1 kinase domain are 7.18, 1.84, 20.60, and 20 Å, respectively. The center and size of the receptor grid of CaSR are 211.00, 204.91, 230.56, and 13.17 Å, respectively. All the crystal waters were kept to generate grids. The protein was assigned protonation states and added hydrogen atoms using the Protein Preparation Wizard in the Schrodinger Suite. Based on WNK463 and cinacalcet, grids were then generated using the Receptor Grid Generation module [29]. The docking of all the complexes was carried out using Glide.

## 4. Conclusions

In summary, we conceived and synthesized forty-two barbatic acid derivatives and evaluated their diuretic and litholytic activity in vitro. The results indicated that compounds **6c**, **6b′**, and **6f′** exhibited more potent diuretic activity, while **6j** and **6m** had better litholytic activity. This demonstrated that the introduction of appropriate substituent groups to the R^2^ of barbatic acid was found to increase the activity of the compounds. Mechanistic studies further revealed that **6b′** possessed an optimal binding affinity to WNK1 kinases, and **6j** could bind to the bicarbonate transporter CaSR. These compounds have the potential to be used as diuretic agents for the treatment of nephrolithiasis in the future.

## Figures and Tables

**Table 1:** Cytotoxicity of compounds **1**, **6a**–**6p′** on MDCK Cells at 100 μmol/L.

Compound	Growth Inhibition Rate (%) ^a^	Compound	Growth Inhibition Rate (%) ^a^
**6a**	/ ^b^	**6w**	0.7 ± 0.30
**6b**	/	**6x**	/
**6c**	/	**6y**	/
**6d**	3.04 ± 0.30	**6z**	2.47 ± 0.50
**6e**	/	**6a′**	/
**6f**	/	**6b′**	/
**6g**	0.12 ± 0.10	**6c′**	/
**6h**	/	**6d′**	1.42 ± 0.00
**6i**	/	**6e′**	/
**6j**	/	**6f′**	/
**6k**	/	**6g′**	/
**6l**	1.43 ± 0.40	**6h′**	/
**6m**	/	**6i′**	2.43 ± 0.30
**6n**	0.36 ± 0.80	**6j′**	/
**6o**	/	**6k′**	1.45 ± 0.10
**6p**	/	**6l′**	2.11 ± 0.90
**6q**	/	**6m′**	/
**6r**	/	**6n′**	/
**6s**	/	**6o′**	/
**6t**	1.42 ± 0.00	**6p′**	/
**6u**	/	**1** ^c^	/
**6v**	/	**Hyd** ^d^	/

^a^ Values are the mean ± SD of three replicates. ^b^ No inhibition action. ^c^ **1** represents barbatic acid. ^d^ **Hyd** represents hydrochlorothiazide.

**Table 2:** Na^+^ and Cl^−^ transport inhibition activity of compounds **1**, **6a**–**6p′** at 100 μmol/L.

Compound	R^1^	R^2^	Transport Inhibition Rate (%) ^a^	
			Na^+^	Cl^−^
**6a**	CH_3_	Bn	33.51 ± 5.95	8.76 ± 0.25
**6b**	CH_3_	CH_3_	−9.31 ± 7.15	5.71 ± 0.00
**6c**	CH_3_	CH_2_CH_3_	47.18 ± 0.80	−7.56 ± 0.35
**6d**	CH_3_	CH_2_CH_2_CH_3_	8.04 ± 0.40	−9.21 ± 0.45
**6e**	CH_3_	CH(CH_3_)CH_3_	−0.88 ± 4.35	−3.13 ± 0.65
**6f**	CH_3_	CH_2_CH=CH_2_	−0.50 ± 0.10	−0.61 ± 0.60
**6g**	CH_3_	CH_2_CN	12.10 ± 1.30	−27.71 ± 1.00
**6h**	CH_2_CH_3_	CH_3_	−7.67 ± 1.00	2.19 ± 0.55
**6i**	CH_2_CH_3_	CH_2_CH_3_	5.20 ± 3.60	−2.13 ± 1.90
**6j**	CH_2_CH_3_	CH_2_CH_2_CH_3_	5.20 ± 3.60	5.75 ± 1.10
**6k**	CH_2_CH_3_	CH(CH_3_)CH_3_	−62.50 ± 1.95	−7.10 ± 1.00
**6l**	CH_2_CH_3_	CH_2_CH=CH_2_	−7.24 ± 0.75	−2.47 ± 0.25
**6m**	CH_2_CH_3_	CH_2_CN	−2.99 ± 6.00	−29.31 ± 1.75
**6n**	CH_2_CH_3_	Bn	−3.98 ± 0.15	−4.47 ± 0.20
**6o**	CH_2_CH_2_CH_3_	CH_3_	−2.68 ± 0.45	−2.80 ± 1.20
**6p**	CH_2_CH_2_CH_3_	CH_2_CH_3_	−12.24 ± 0.45	−18.38 ± 0.15
**6q**	CH_2_CH_2_CH_3_	CH_2_CH_2_CH_3_	9.39 ± 0.25	11.63 ± 2.20
**6r**	CH_2_CH_2_CH_3_	CH_2_CH=CH_2_	1.14 ± 1.25	−10.30 ± 1.40
**6s**	CH_2_CH_2_CH_3_	CH_2_CN	−27.85 ± 0.50	−6.21 ± 0.30
**6t**	CH_2_CH_2_CH_3_	CH(CH_3_)CH_3_	−37.63 ± 0.55	−44.09 ± 3.50
**6u**	CH_2_CH_2_CH_3_	Bn	6.31 ± 2.35	−15.89 ± 0.55
**6v**	CH(CH_3_)CH_3_	CH_3_	−9.43 ± 0.75	−13.55 ± 0.10
**6w**	CH(CH_3_)CH_3_	CH_2_CH_3_	33.11 ± 0.55	−80.44 ± 1.20
**6x**	CH(CH_3_)CH_3_	CH_2_CH_2_CH_3_	−1.11 ± 1.75	−1.92 ± 0.25
**6y**	CH(CH_3_)CH_3_	CH(CH_3_)CH_3_	29.35 ± 1.85	−43.32 ± 3.25
**6z**	CH(CH_3_)CH_3_	CH_2_CH=CH_2_	−22.66 ± 1.05	−32.59 ± 3.10
**6a′**	CH(CH_3_)CH_3_	CH_2_CN	−15.79 ± 4.85	−10.00 ± 1.95
**6b′**	CH(CH_3_)CH_3_	Bn	33.08 ± 0.35	15.93 ± 0.25
**6c′**	CH_2_CH=CH_2_	CH_3_	15.21 ± 1.25	−18.16 ± 1.45
**6d′**	CH_2_CH=CH_2_	CH_2_CH_3_	−7.24 ± 0.75	−5.06 ± 0.35
**6e′**	CH_2_CH=CH_2_	CH_2_CH_2_CH_3_	2.79 ± 0.25	−12.90 ± 0.85
**6f′**	CH_2_CH=CH_2_	CH(CH_3_)CH_3_	32.83 ± 2.50	1.88 ± 4.25
**6g′**	CH_2_CH=CH_2_	CH_2_CH=CH_2_	5.29 ± 0.15	18.58 ± 2.20
**6h′**	CH_2_CH=CH_2_	CH_2_CN	−14.38 ± 2.40	0.62 ± 0.70
**6i′**	CH_2_CH=CH_2_	Bn	−12.89 ± 1.60	−8.43 ± 2.85
**6j′**	Bn	CH_3_	−5.13 ± 0.25	0.62 ± 0.80
**6k′**	Bn	CH_2_CH_3_	14.10 ± 3.20	−19.60 ± 2.40
**6l′**	Bn	CH_2_CH_2_CH_3_	−18.25 ± 0.20	−2.92 ± 2.15
**6m′**	Bn	CH(CH_3_)CH_3_	−0.36 ± 0.40	−4.93 ± 2.95
**6n′**	Bn	CH_2_CH=CH_2_	−1.85 ± 0.80	−12.97 ± 0.15
**6o′**	Bn	CH_2_CN	−3.28 ± 0.75	−24.03 ± 1.50
**6p′**	Bn	Bn	16.30 ± 0.10	−5.43 ± 0.85
**1** ^b^	CH_3_	H	12.27 ± 0.50	2.34 ± 1.20
**Hyd** ^c^			25.09 ± 0.85	19.74 ± 1.00

^a^ Values are the mean ± SD of three replicates. ^b^ **1** represents barbatic acid. ^c^ **Hyd** (hydrochlorothiazide) was used as the positive control.

**Table 3:** Ca^2+^ and C_2_O_4_^2−^ transport inhibition activity of compounds **1**, **6a**–**6p′** at 100 μmol/L.

Compound	R^1^	R^2^	Transport Inhibition Rate (%) ^a^	
			Ca^2+^	C_2_O_4_^2−^
**6a**	CH_3_	Bn	−7.98 ± 0.17	−16.65 ± 0.37
**6b**	CH_3_	CH_3_	−5.14 ± 0.31	−4.25 ± 1.08
**6c**	CH_3_	CH_2_CH_3_	−2.30 ± 0.34	1.01 ± 2.03
**6d**	CH_3_	CH_2_CH_2_CH_3_	−9.93 ± 0.19	−6.69 ± 8.67
**6e**	CH_3_	CH(CH_3_)CH_3_	−3.66 ± 0.27	−2.05 ± 3.00
**6f**	CH_3_	CH_2_CH=CH_2_	−3.66 ± 0.25	6.89 ± 1.50
**6g**	CH_3_	CH_2_CN	−8.61 ± 0.19	−12.63 ± 2.88
**6h**	CH_2_CH_3_	CH_3_	−3.85 ± 0.19	−4.23 ± 4.77
**6i**	CH_2_CH_3_	CH_2_CH_3_	1.71 ± 0.13	54.05 ± 10.39
**6j**	CH_2_CH_3_	CH_2_CH_2_CH_3_	4.45 ± 0.29	42.26 ± 1.66
**6k**	CH_2_CH_3_	CH(CH_3_)CH_3_	1.20 ± 0.13	−5.12 ± 1.75
**6l**	CH_2_CH_3_	CH_2_CH=CH_2_	2.23 ± 0.10	8.10 ± 2.68
**6m**	CH_2_CH_3_	CH_2_CN	2.57 ± 0.10	60.95 ± 2.75
**6n**	CH_2_CH_3_	Bn	−2.13 ± 0.30	0.11 ± 0.70
**6o**	CH_2_CH_2_CH_3_	CH_3_	4.43 ± 0.13	5.60 ± 3.85
**6p**	CH_2_CH_2_CH_3_	CH_2_CH_3_	4.79 ± 0.46	4.29 ± 0.24
**6q**	CH_2_CH_2_CH_3_	CH_2_CH_2_CH_3_	4.61 ± 0.32	−25.98 ± 4.17
**6r**	CH_2_CH_2_CH_3_	CH_2_CH=CH_2_	5.85 ± 0.36	9.67 ± 4.60
**6s**	CH_2_CH_2_CH_3_	CH_2_CN	−1.74 ± 0.12	15.68 ± 1.13
**6t**	CH_2_CH_2_CH_3_	CH(CH_3_)CH_3_	−1.74 ± 0.29	11.94 ± 5.83
**6u**	CH_2_CH_2_CH_3_	Bn	0.77 ± 0.09	22.32 ± 5.04
**6v**	CH(CH_3_)CH_3_	CH_3_	0.19 ± 0.29	−17.94 ± 1.35
**6w**	CH(CH_3_)CH_3_	CH_2_CH_3_	1.55 ± 0.11	18.97 ± 2.64
**6x**	CH(CH_3_)CH_3_	CH_2_CH_2_CH_3_	2.74 ± 0.22	−5.42 ± 2.83
**6y**	CH(CH_3_)CH_3_	CH(CH_3_)CH_3_	3.47 ± 0.30	−26.32 ± 5.41
**6z**	CH(CH_3_)CH_3_	CH_2_CH=CH_2_	−2.56 ± 0.17	0.23 ± 2.16
**6a′**	CH(CH_3_)CH_3_	CH_2_CN	−0.91 ± 0.11	−13.20 ± 1.67
**6b′**	CH(CH_3_)CH_3_	Bn	0.73 ± 0.08	−20.59 ± 1.31
**6c′**	CH_2_CH=CH_2_	CH_3_	1.12 ± 0.12	10.04 ± 0.98
**6d′**	CH_2_CH=CH_2_	CH_2_CH_3_	2.04 ± 0.03	0.52 ± 1.86
**6e′**	CH_2_CH=CH_2_	CH_2_CH_2_CH_3_	2.04 ± 0.08	5.77 ± 3.15
**6f′**	CH_2_CH=CH_2_	CH(CH_3_)CH_3_	1.12 ± 0.08	15.51 ± 3.62
**6g′**	CH_2_CH=CH_2_	CH_2_CH=CH_2_	2.79 ± 0.13	−11.31 ± 1.90
**6h′**	CH_2_CH=CH_2_	CH_2_CN	−0.38 ± 0.16	10.69 ± 1.41
**6i′**	CH_2_CH=CH_2_	Bn	2.68 ± 0.11	11.41 ± 1.13
**6j′**	Bn	CH_3_	0.00 ± 0.09	−1.79 ± 3.79
**6k′**	Bn	CH_2_CH_3_	1.34 ± 0.10	10.26 ± 2.11
**6l′**	Bn	CH_2_CH_2_CH_3_	−2.68 ± 0.39	16.50 ± 4.38
**6m′**	Bn	CH(CH_3_)CH_3_	−2.06 ± 0.09	2.28 ± 1.34
**6n′**	Bn	CH_2_CH=CH_2_	0.10 ± 0.30	−29.42 ± 3.89
**6o′**	Bn	CH_2_CN	−1.82 ± 0.23	2.03 ± 1.07
**6p′**	Bn	Bn	−3.34 ± 0.26	7.16 ± 1.56
**1** ^b^	CH_3_	H	8.80 ± 0.18	2.97 ± 2.21

^a^ Values are the mean ± SD of three replicates. ^b^ **1** represents barbatic acid.

## Data Availability

Data are included within the manuscript or the Supplementary Materials.

## Supplementary Materials

The following supporting information can be downloaded at: https://www.mdpi.com/article/10.3390/molecules28104010/s1, Figure S1: The binding mode of co-crystallized (green) and re-docked (cyan) WNK463 and cinacalcet in the WNK1 kinase domain and CaSR, respectively; Figures S2–S127: ^1^H NMR, ^13^C NMR and HRMS spectrum of the compounds **6a**–**p’**.
