# Supplementary material for: Semisynthesis, Biological Evaluation and Molecular Docking Studies of Barbatic Acid Derivatives as Novel Diuretic Candidates
Source: Molecules. 2023 May 10;28(10):4010. doi: 10.3390/molecules28104010 (PMC10222548; doi:10.3390/molecules28104010)
Supplement: Supplementary file 1 [file molecules-28-04010-s001.zip › molecules-2355306-supplementary.pdf]

Supporting Information

Semisynthesis, Biological Evaluation and Molecular Docking Studies of Barbatic Acid Derivatives as Novel Diuretic Candidates

Xiang Yu [1,2,†], Yi Sui [1,†], Yinkai Xi [1,†], Yan Zhang [1], Guoyong Luo [1], Yi Long [1] and Wude Yang [1,2,*]

1 College of Pharmacy, Guizhou University of Traditional Chinese Medicine, Guiyang 550025, China; yuxiangjx@126.com (X.Y.); suiyi041@gzy.edu.cn (Y.S.); xyk_xyt@163.com (Y.X.); zhangyan0003@gzy.edu.cn (Y.Z.); ft420038851@163.com (G.L.); longyi621@gzy.edu.cn (Y.L.)

2 Guizhou Joint Laboratory for International Cooperation in Ethnic Medicine, Guizhou University of Traditional Chinese Medicine, Guiyang 550025, China

* Correspondence: ywd_680708@sina.com

† These authors contributed equally to this work.

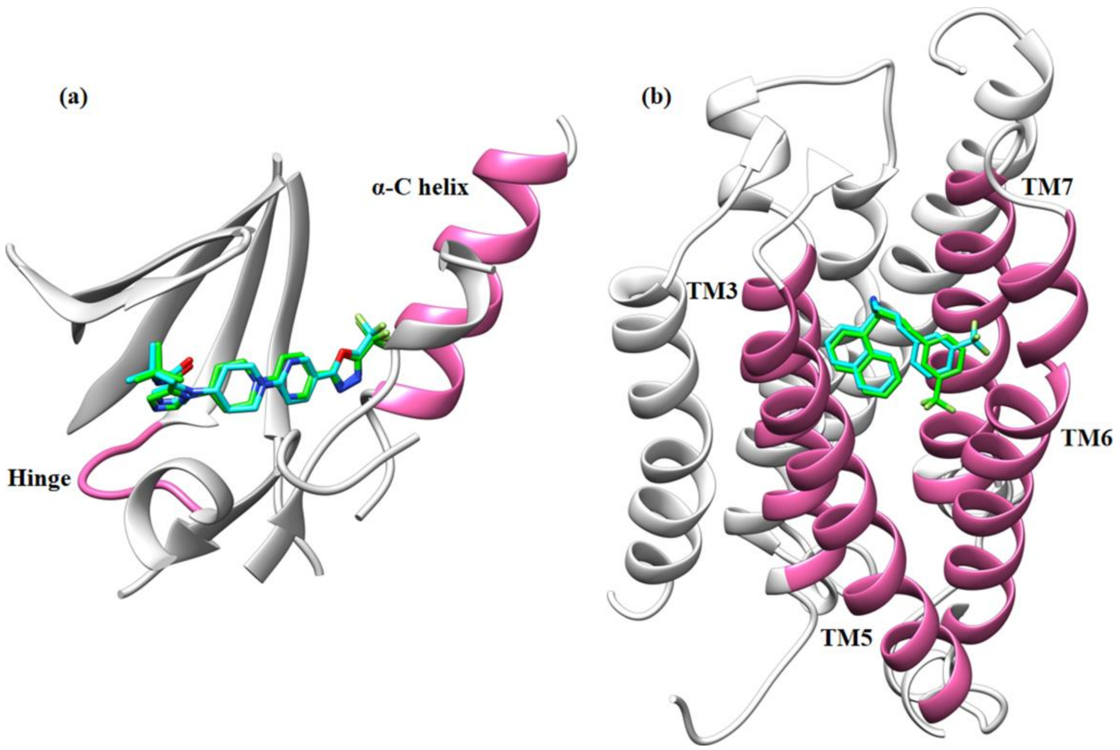

Figure S1. The binding mode of co-crystallized (green) and re-docked (cyan) WNK463 and cinacalcet in the WNK1 kinase domain and CaSR, respectively.

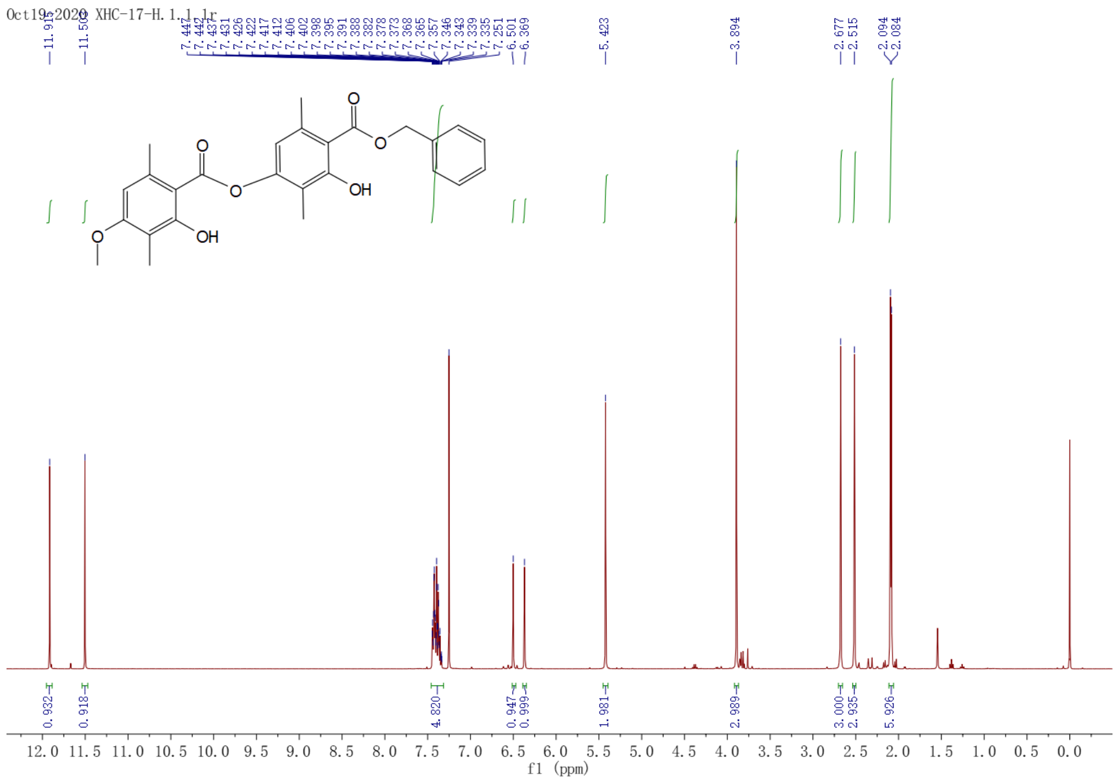

Figure S2. ^{1}H NMR spectrum of compound **6a**

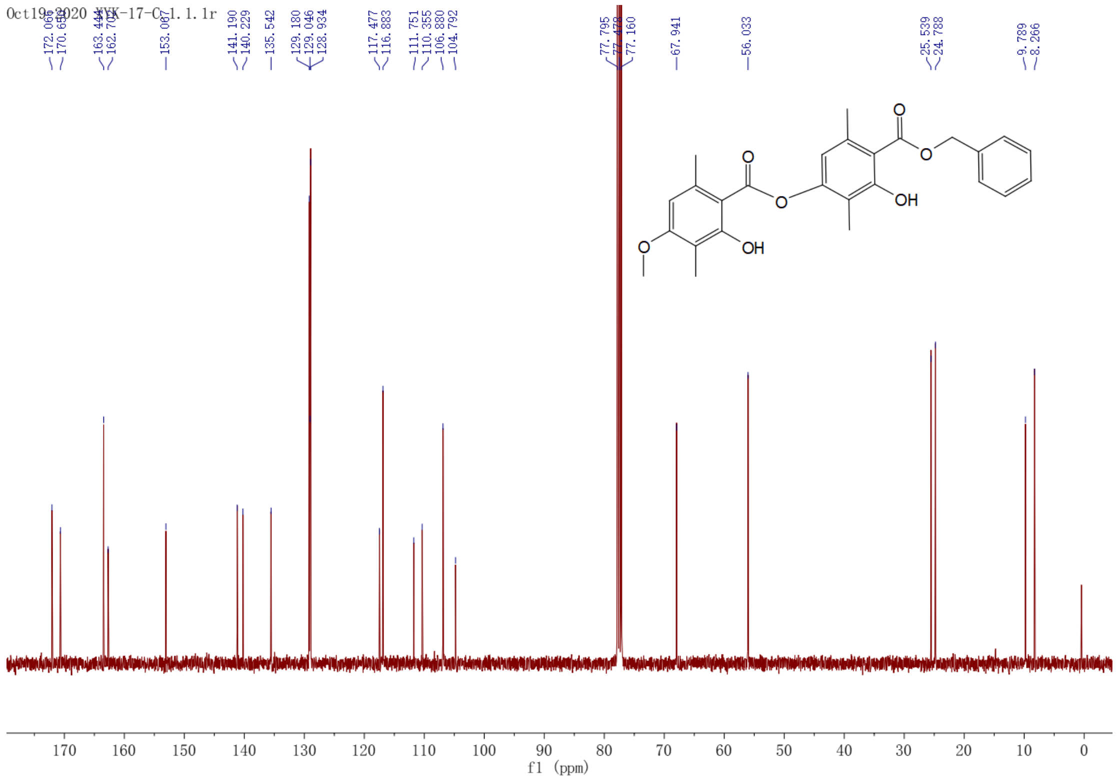

Figure S3. ^{13}C NMR spectrum of compound **6a**

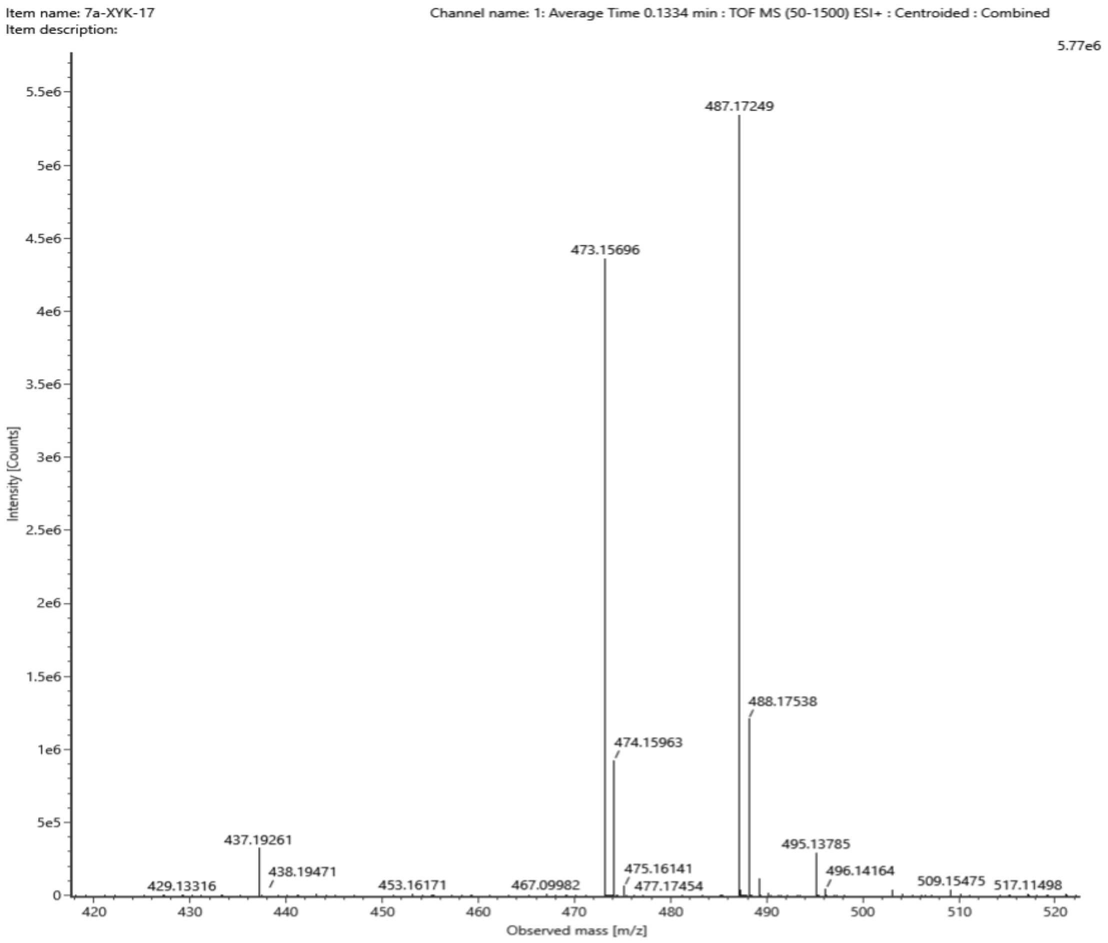

Figure S4. HRMS spectrum of compound **6a**

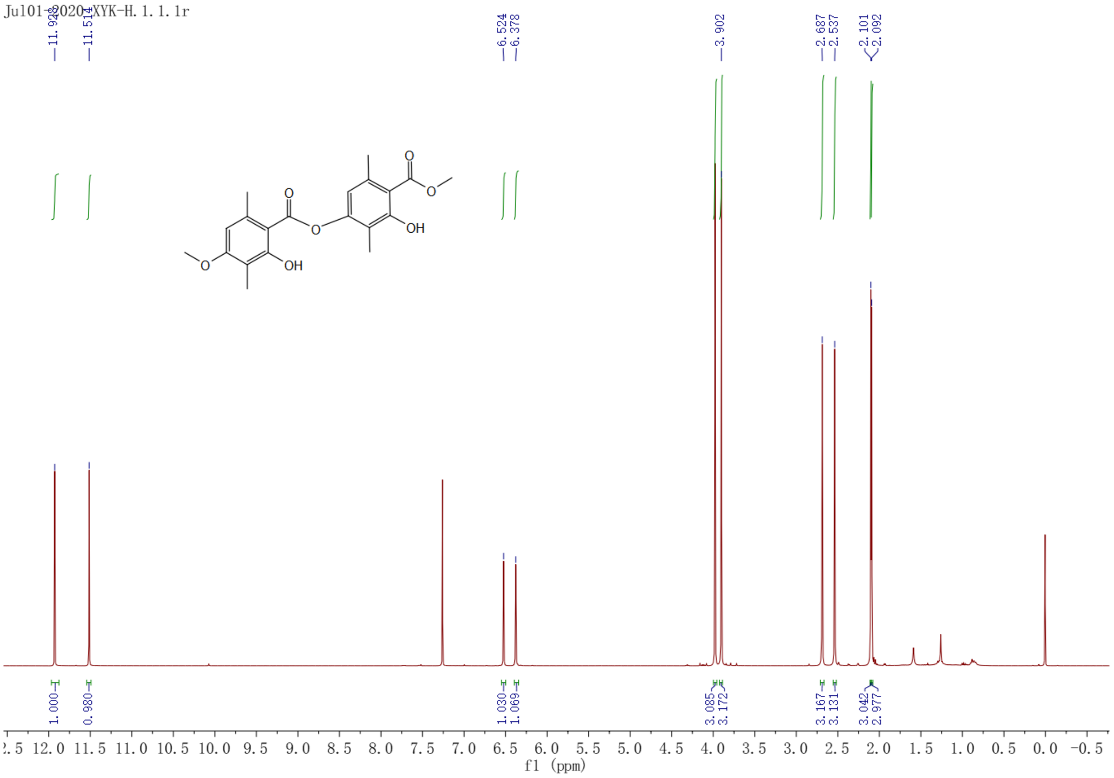

Figure S5. ^{1}H NMR spectrum of compound **6b**

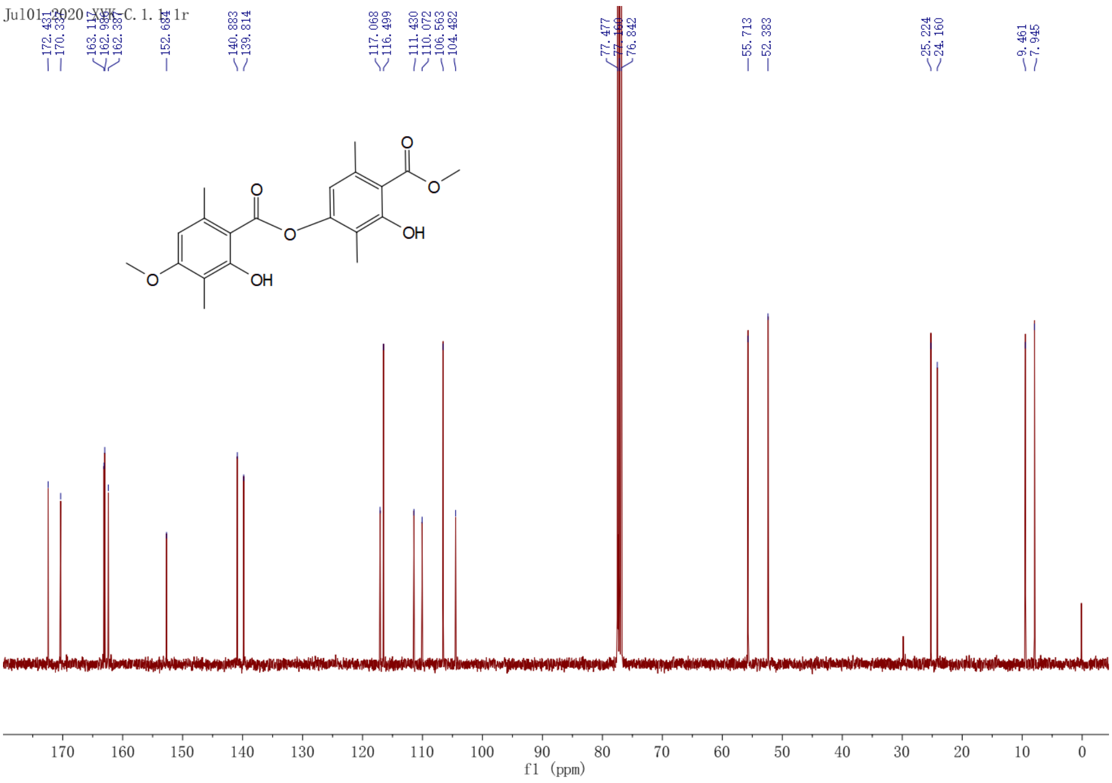

Figure S6. ^{13}C NMR spectrum of compound **6b**

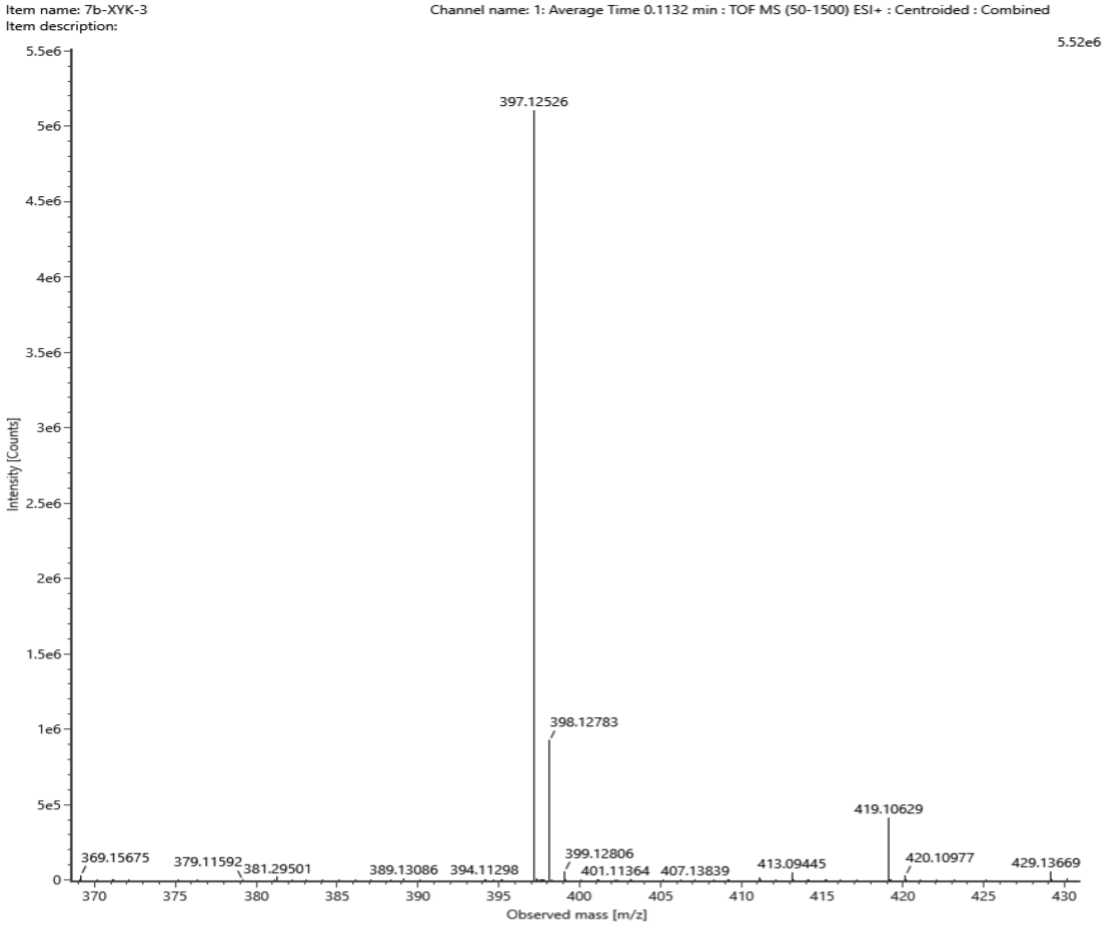

Figure S7. HRMS spectrum of compound **6b**

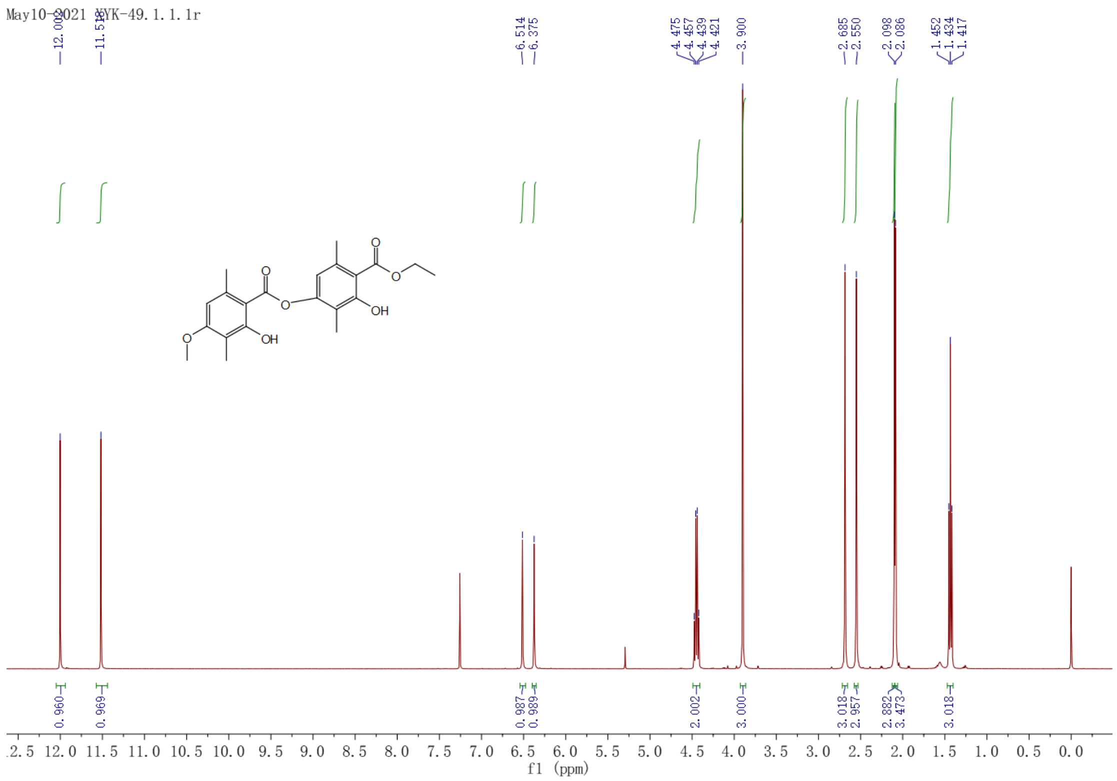

Figure S8. ^{1}H NMR spectrum of compound **6c**

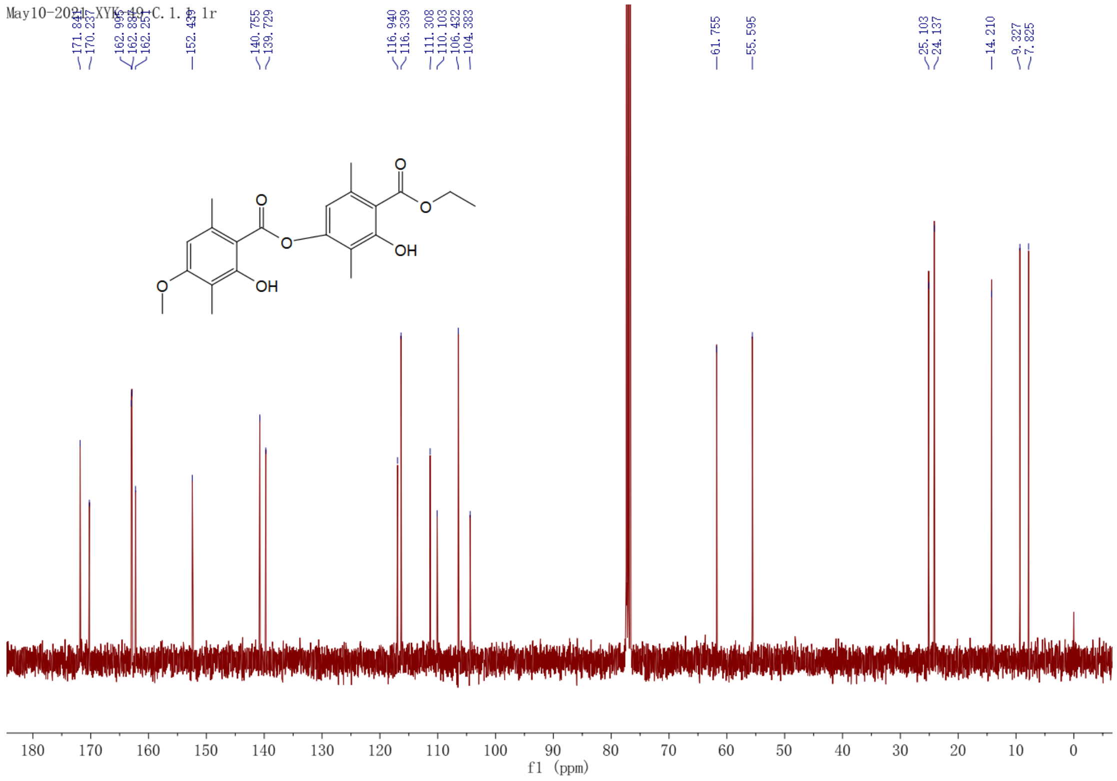

Figure S9. ^{13}C NMR spectrum of compound **6c**

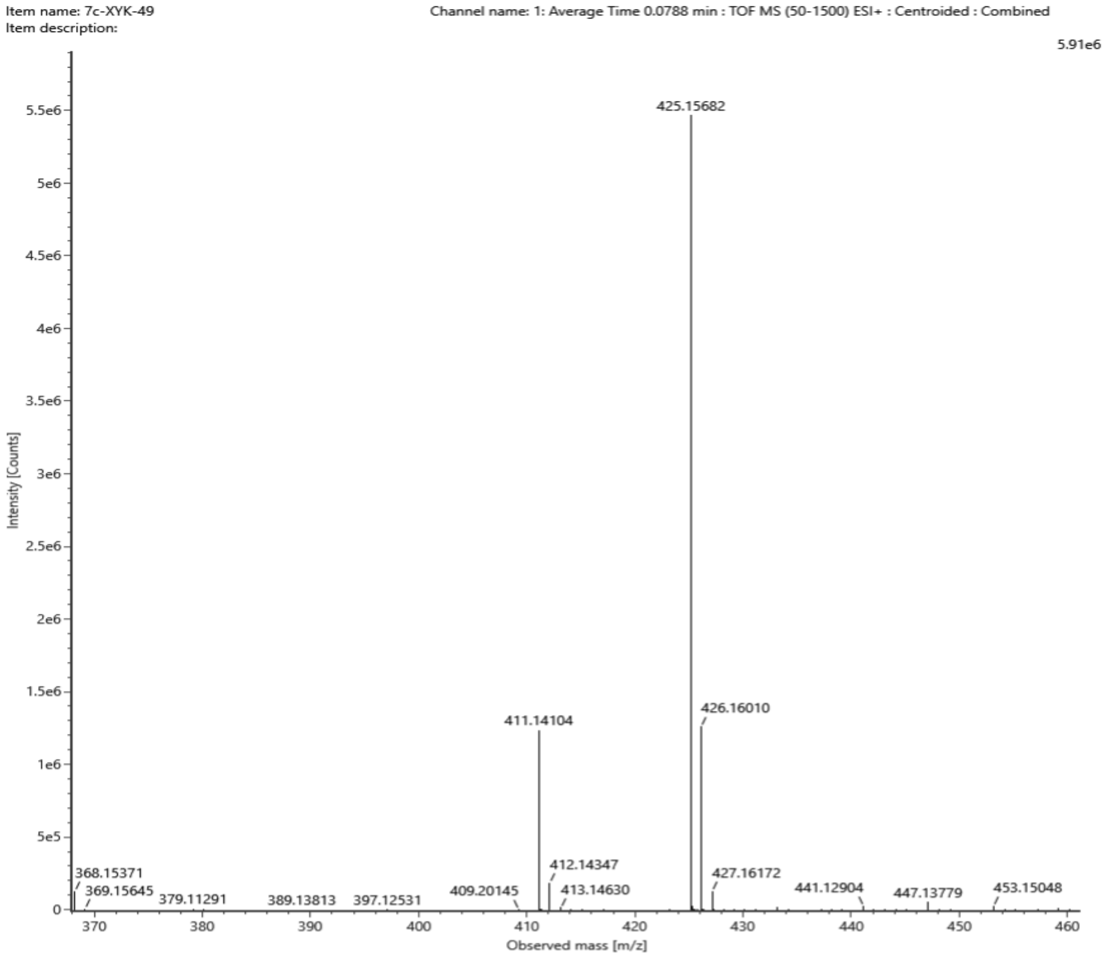

Figure S10. HRMS spectrum of compound **6c**

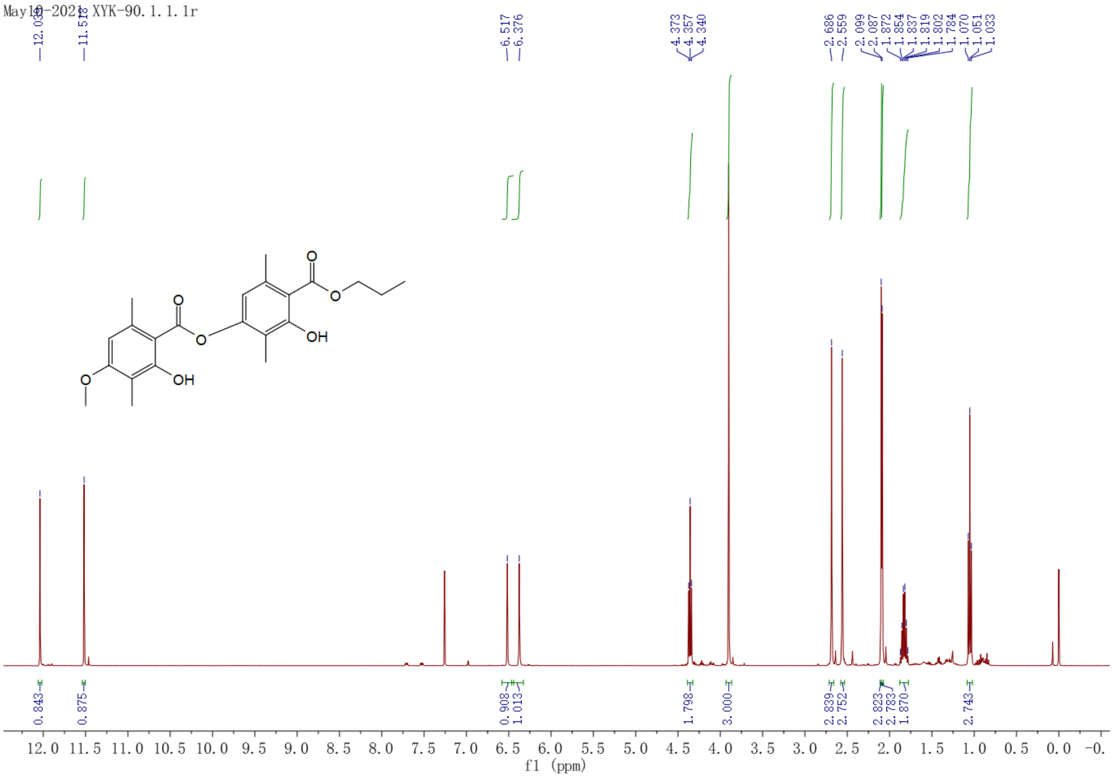

Figure S11. ^{1}H NMR spectrum of compound **6d**

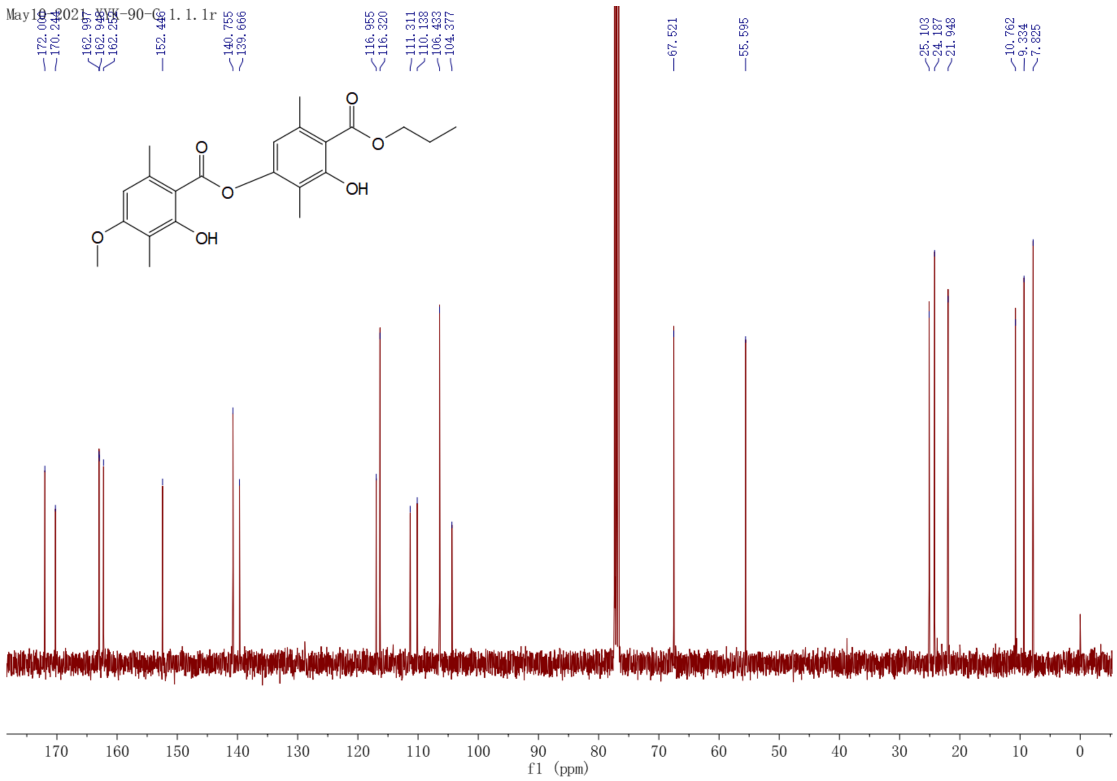

Figure S12. ^{13}C NMR spectrum of compound **6d**

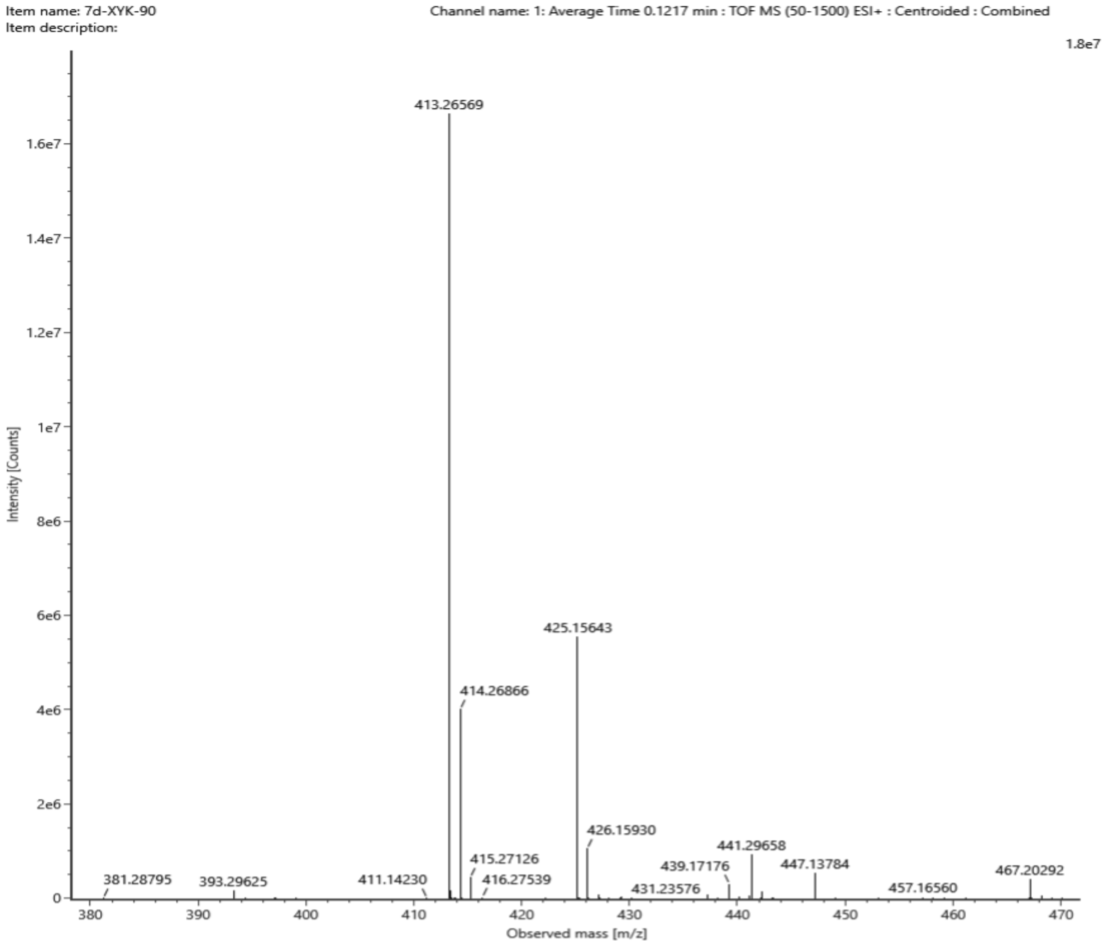

Figure S13. HRMS spectrum of compound **6d**

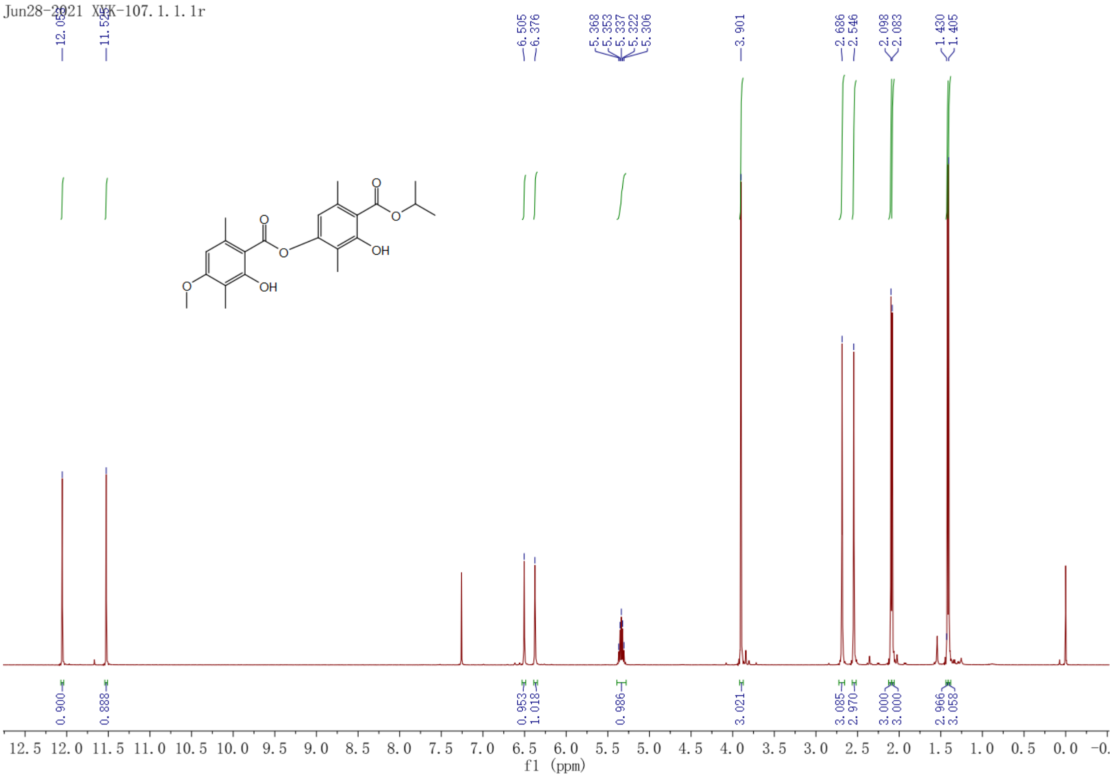

Figure S14. ^{1}H NMR spectrum of compound **6e**

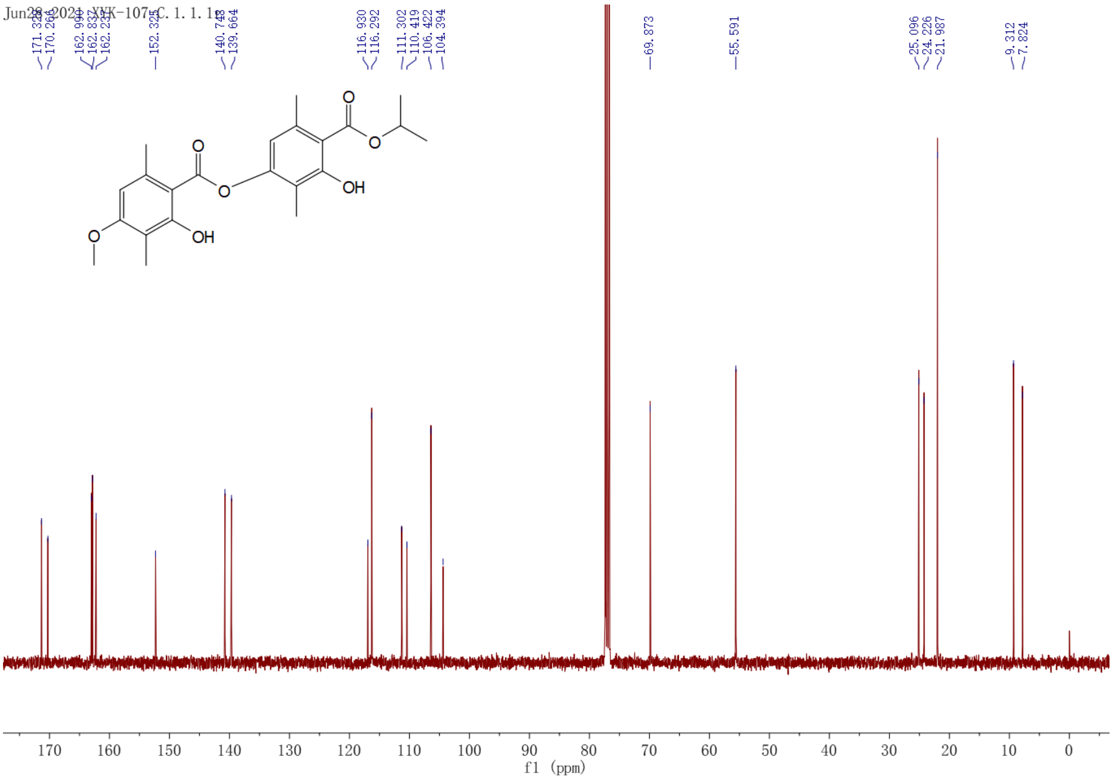

Figure S15. ^{13}C NMR spectrum of compound **6e**

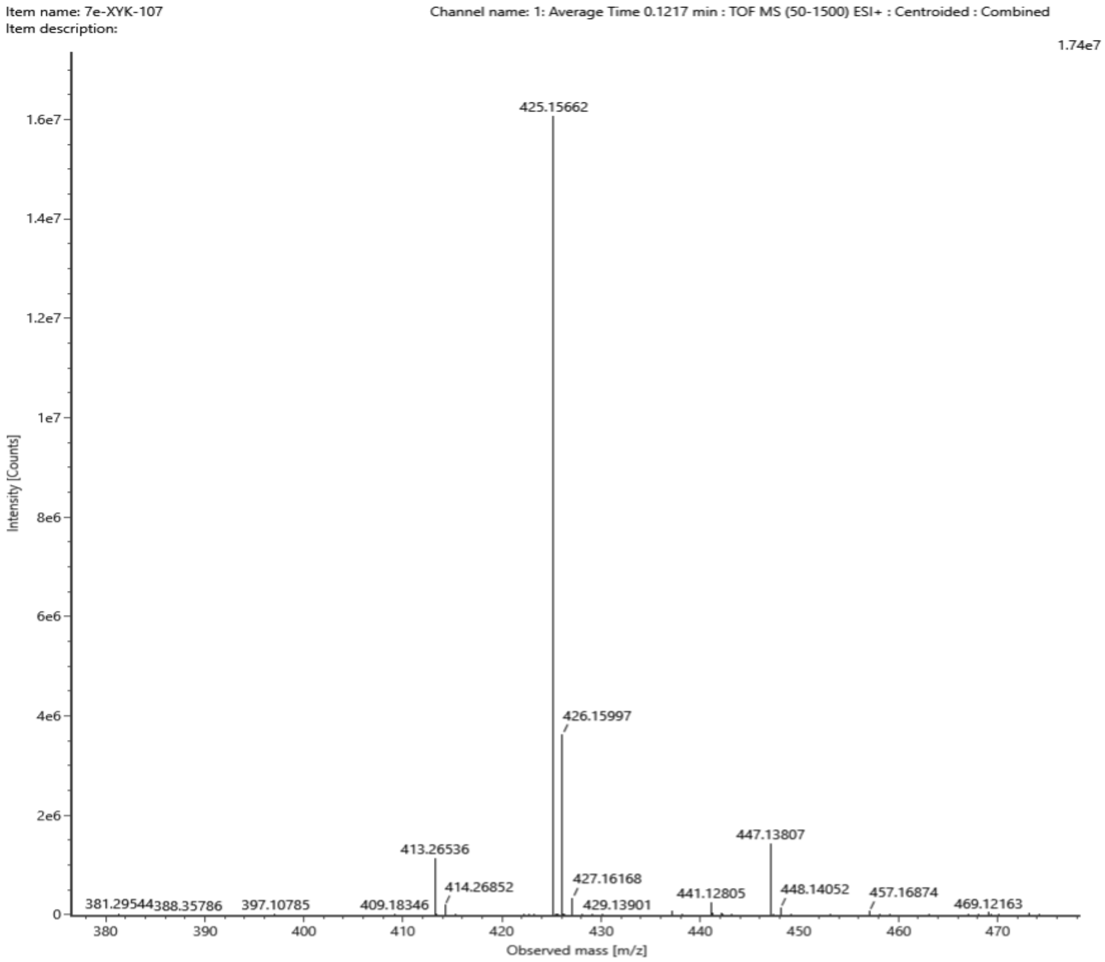

Figure S16. HRMS spectrum of compound **6e**

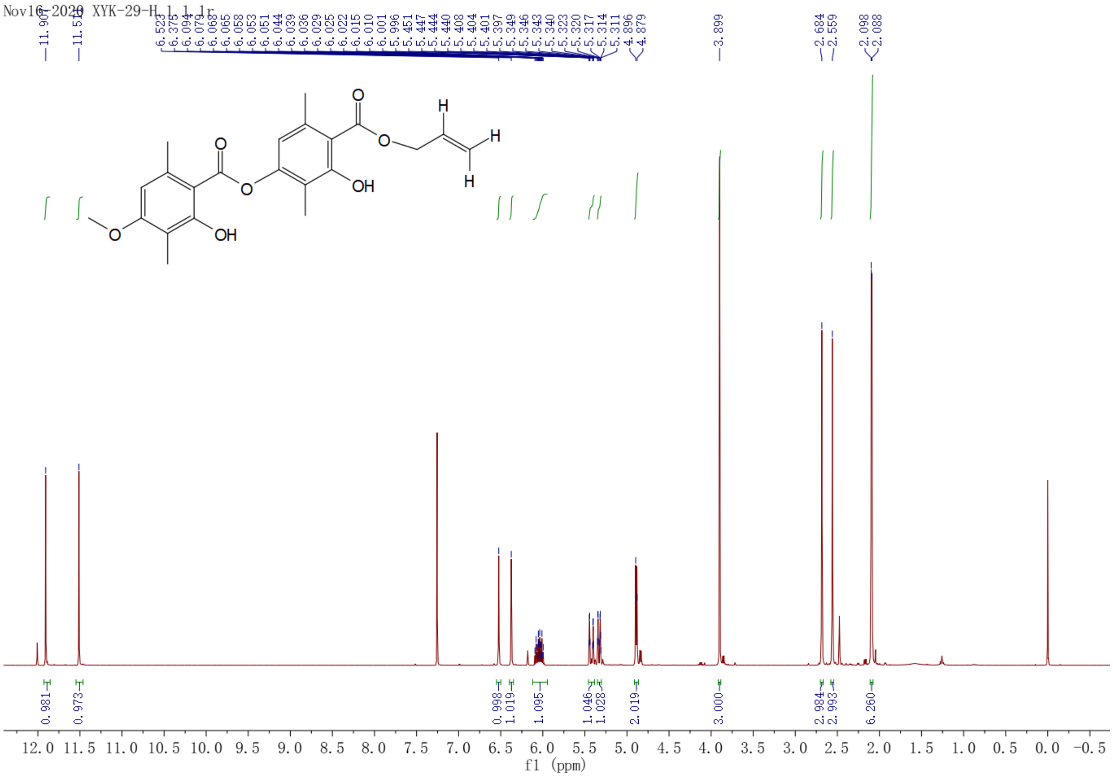

Figure S17. ^{1}H NMR spectrum of compound **6f**

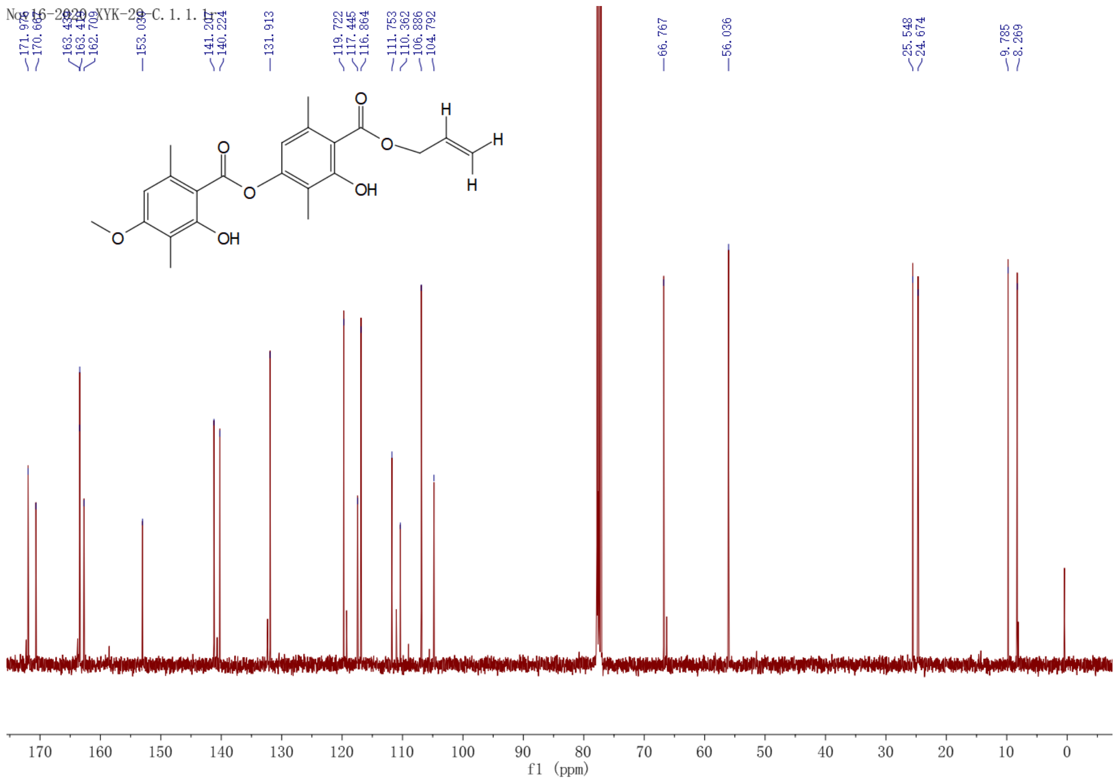

Figure S18. ^{13}C NMR spectrum of compound **6f**

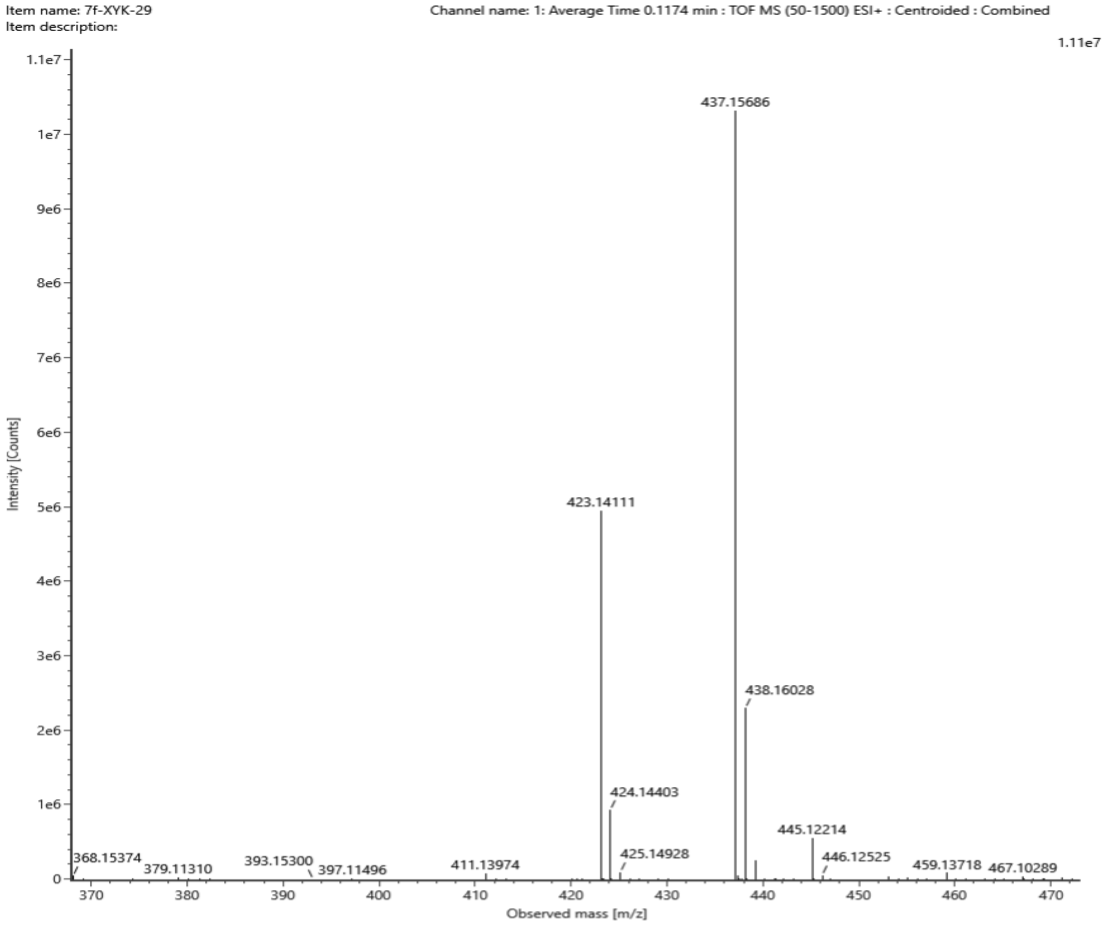

Figure S19. HRMS spectrum of compound **6f**

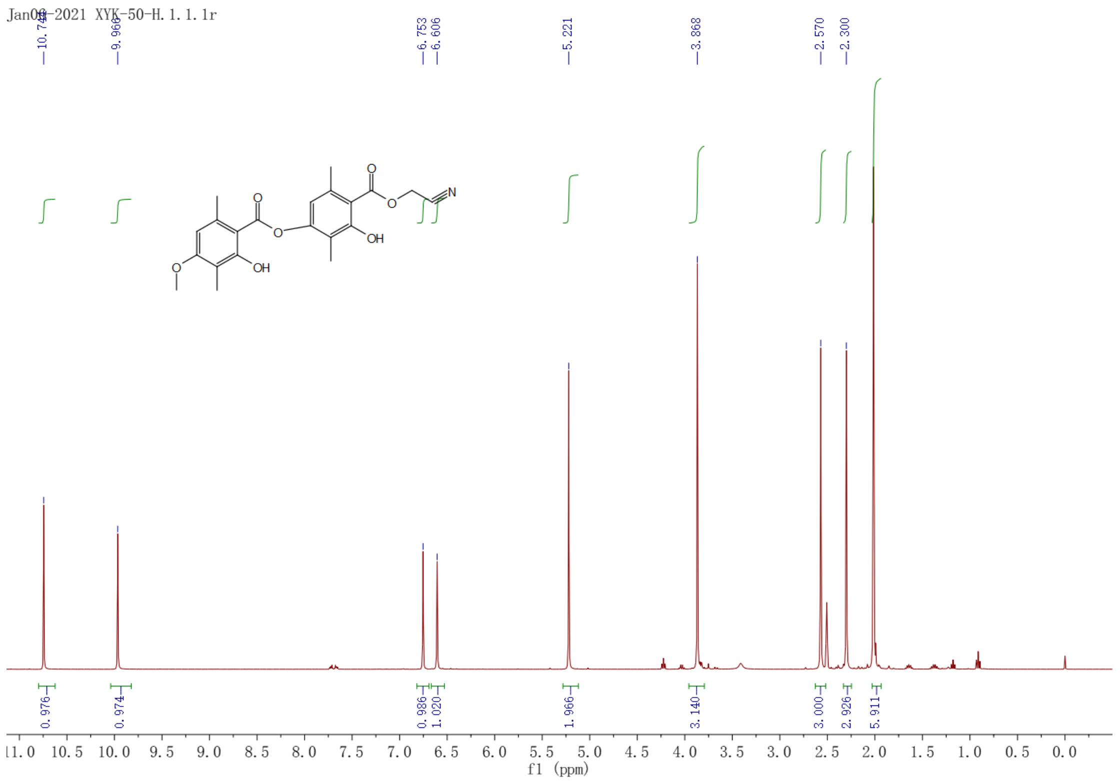

Figure S20. ^{1}H NMR spectrum of compound **6g**

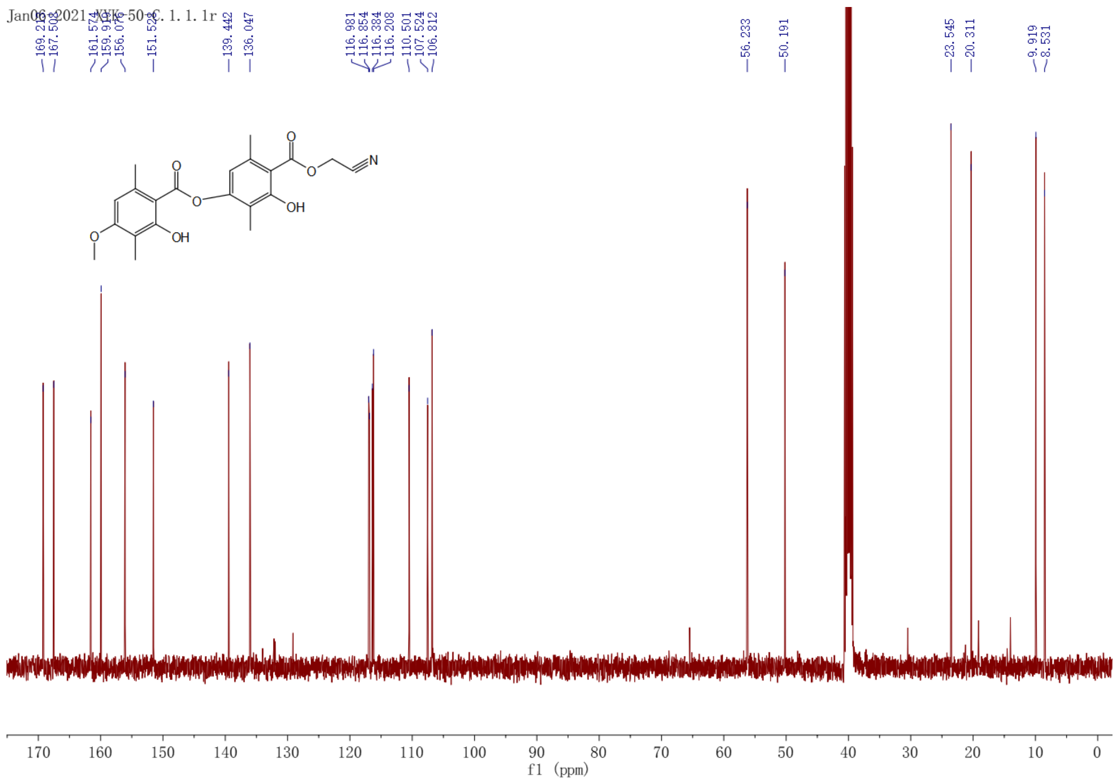

Figure S21. ^{13}C NMR spectrum of compound **6g**

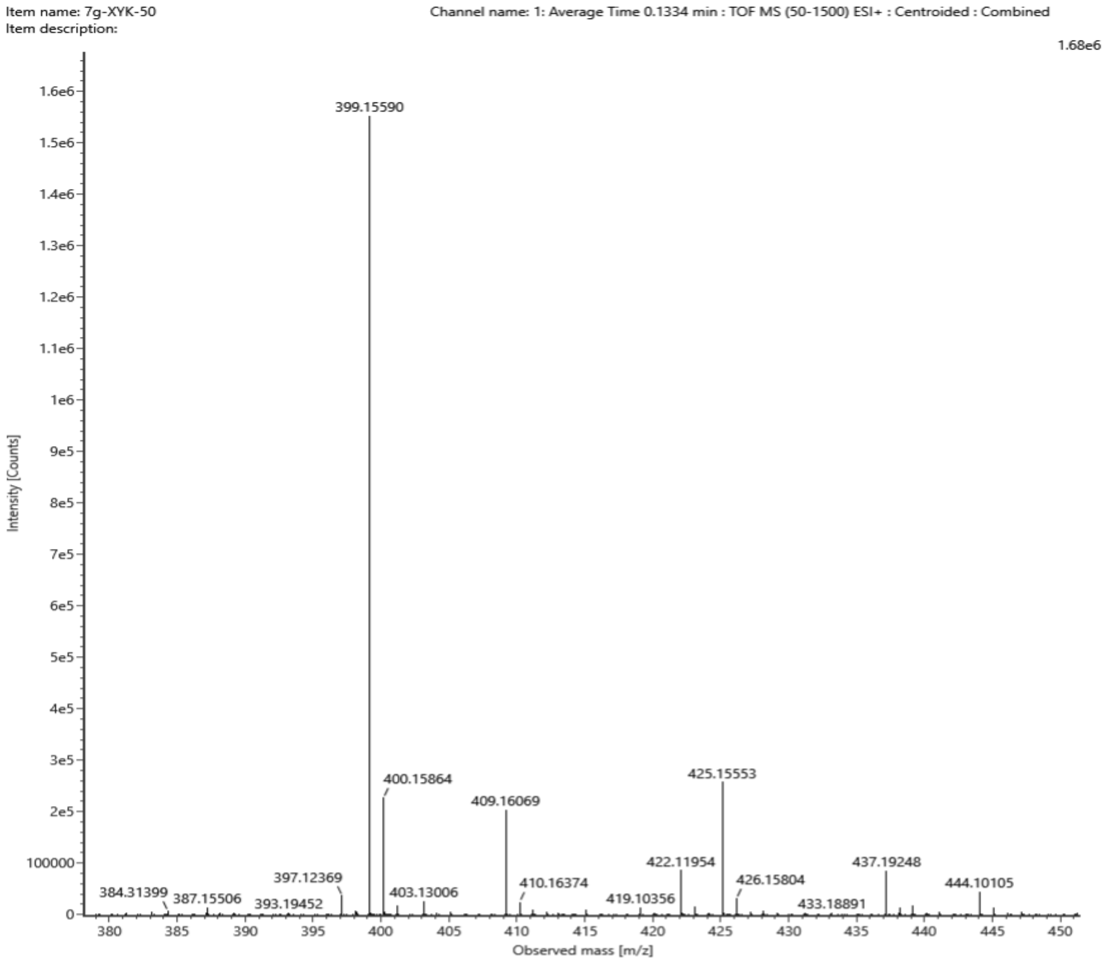

Figure S22. HRMS spectrum of compound **6g**

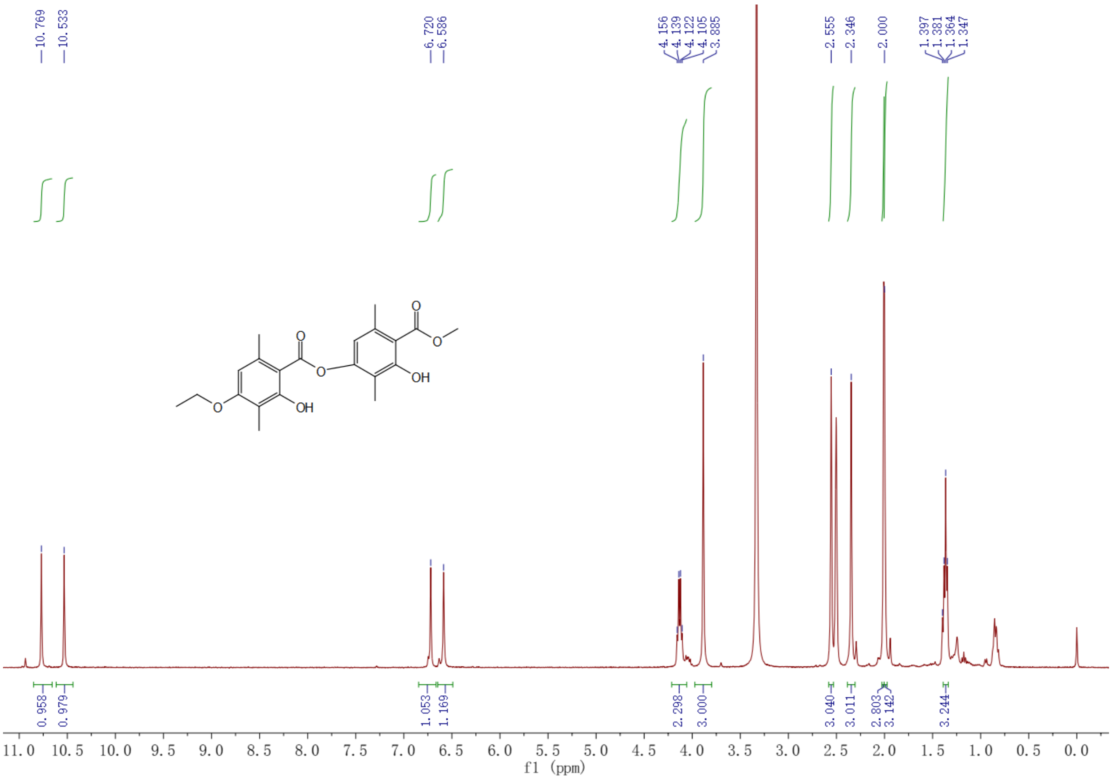

Figure S23. ^{1}H NMR spectrum of compound **6h**

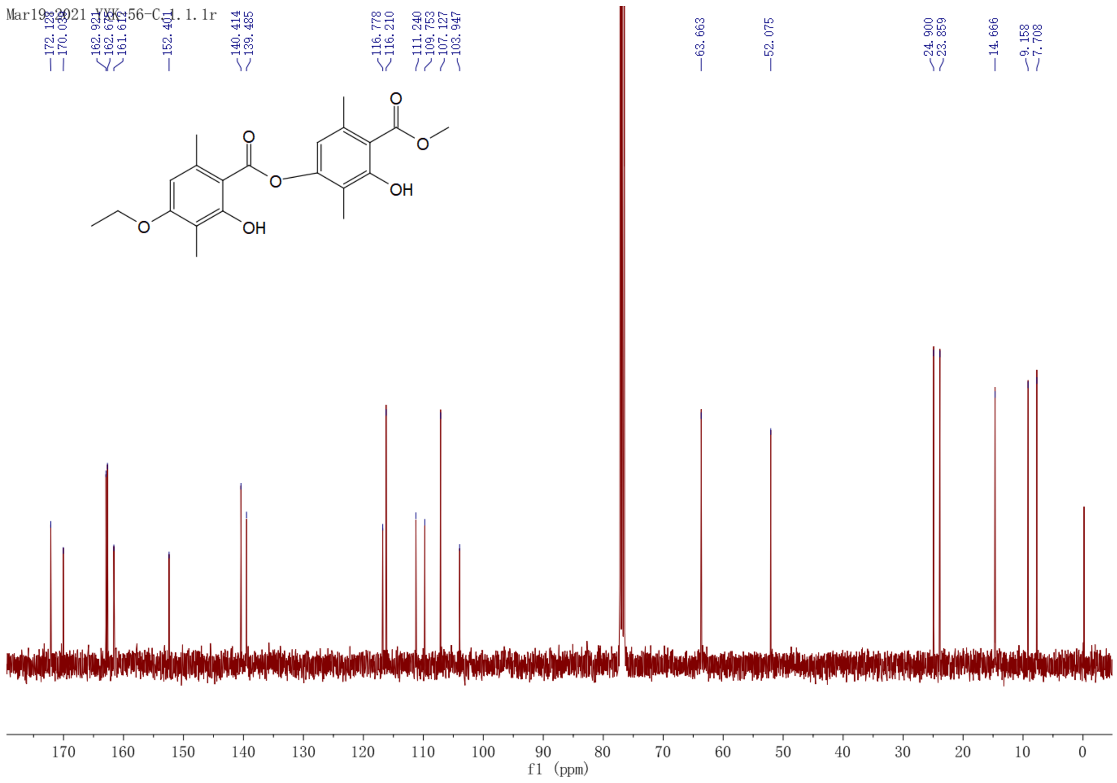

Figure S24. ^{13}C NMR spectrum of compound **6h**

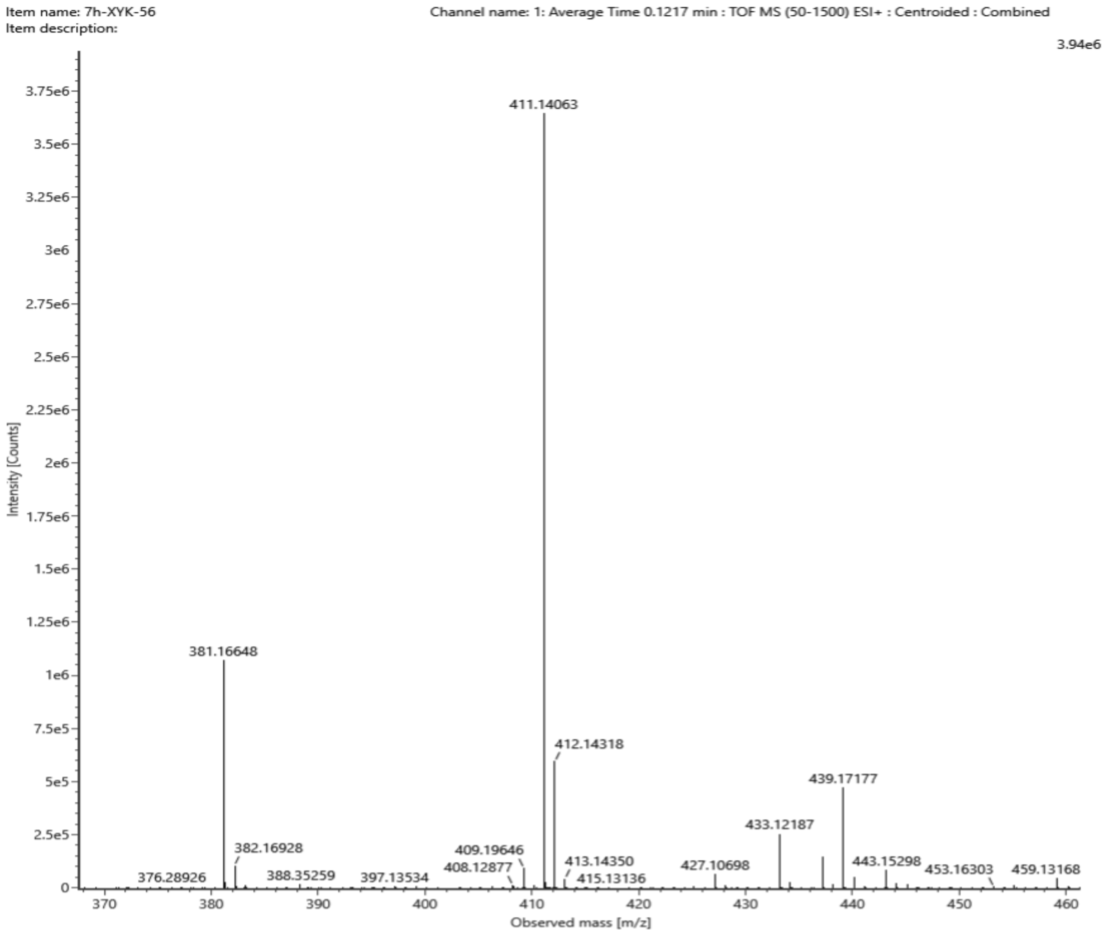

Figure S25. HRMS spectrum of compound **6h**

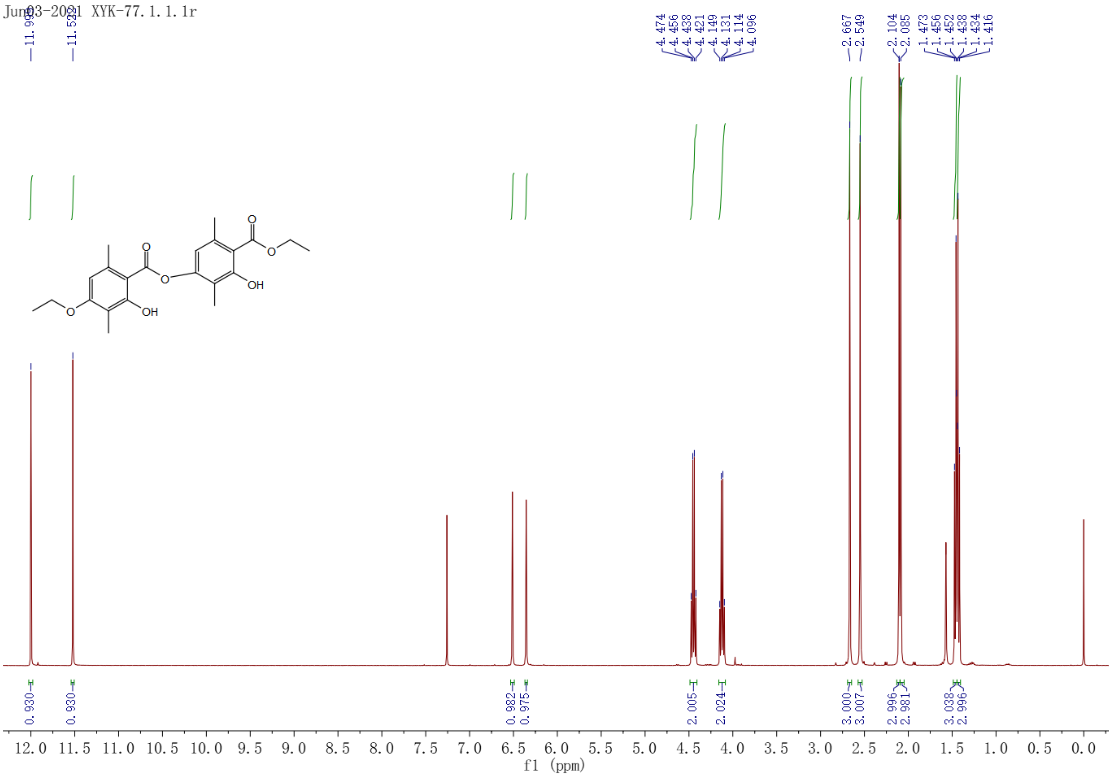

Figure S26. ^{1}H NMR spectrum of compound **6i**

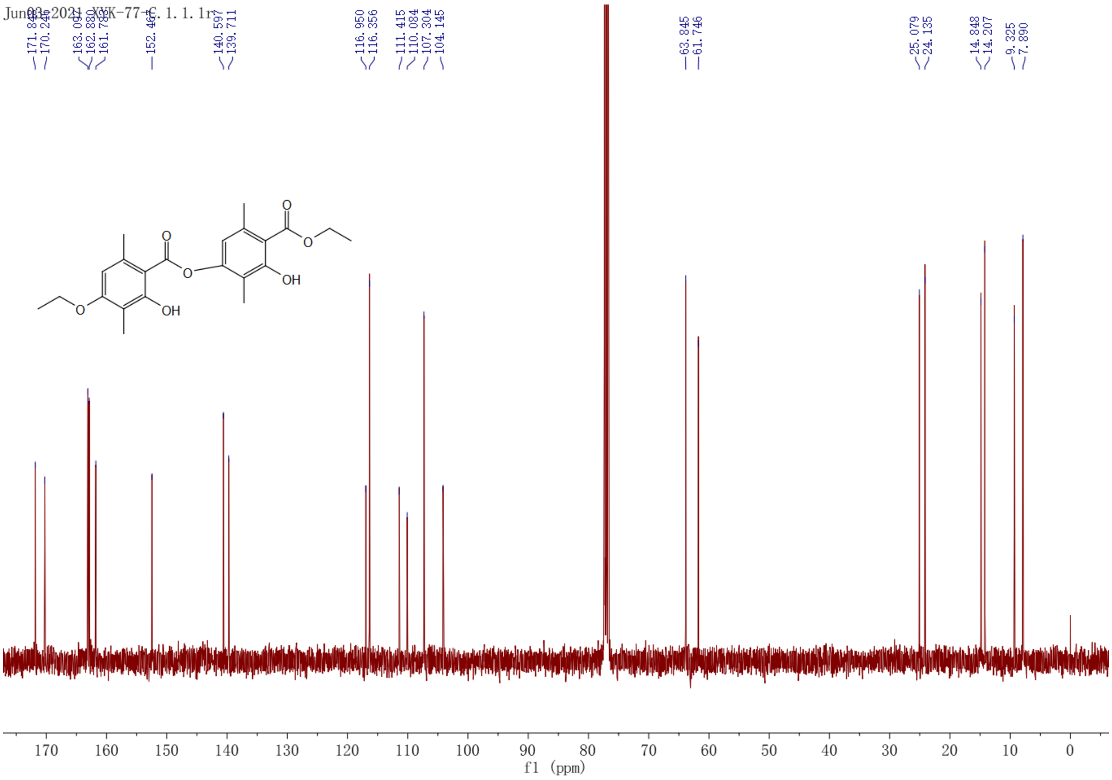

Figure S27. ^{13}C NMR spectrum of compound **6i**

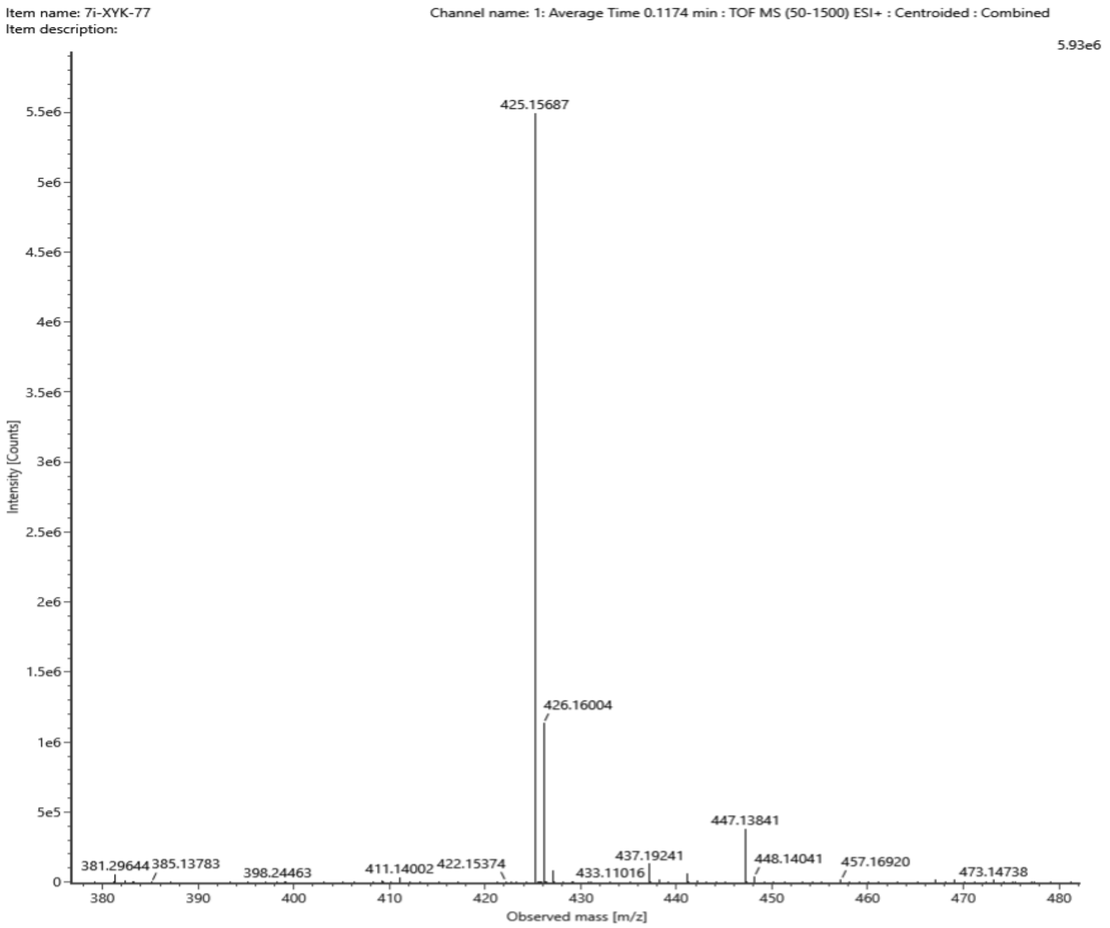

Figure S28. HRMS spectrum of compound **6i**

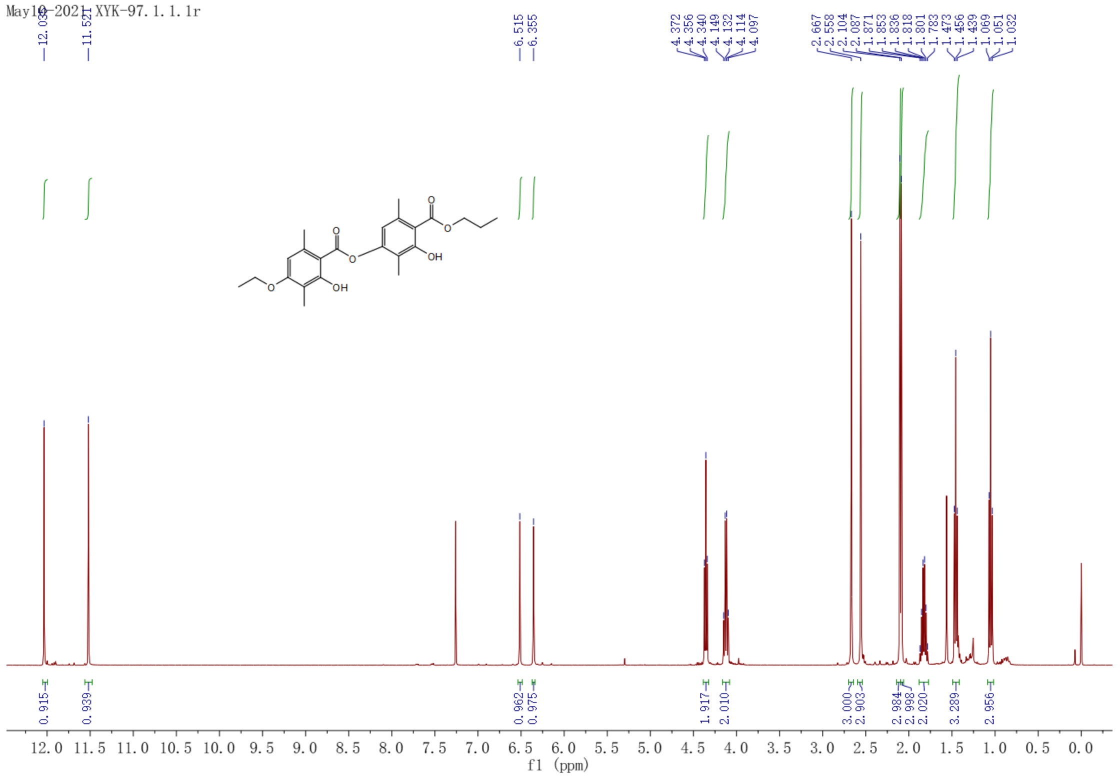

Figure S29. ^{1}H NMR spectrum of compound **6j**

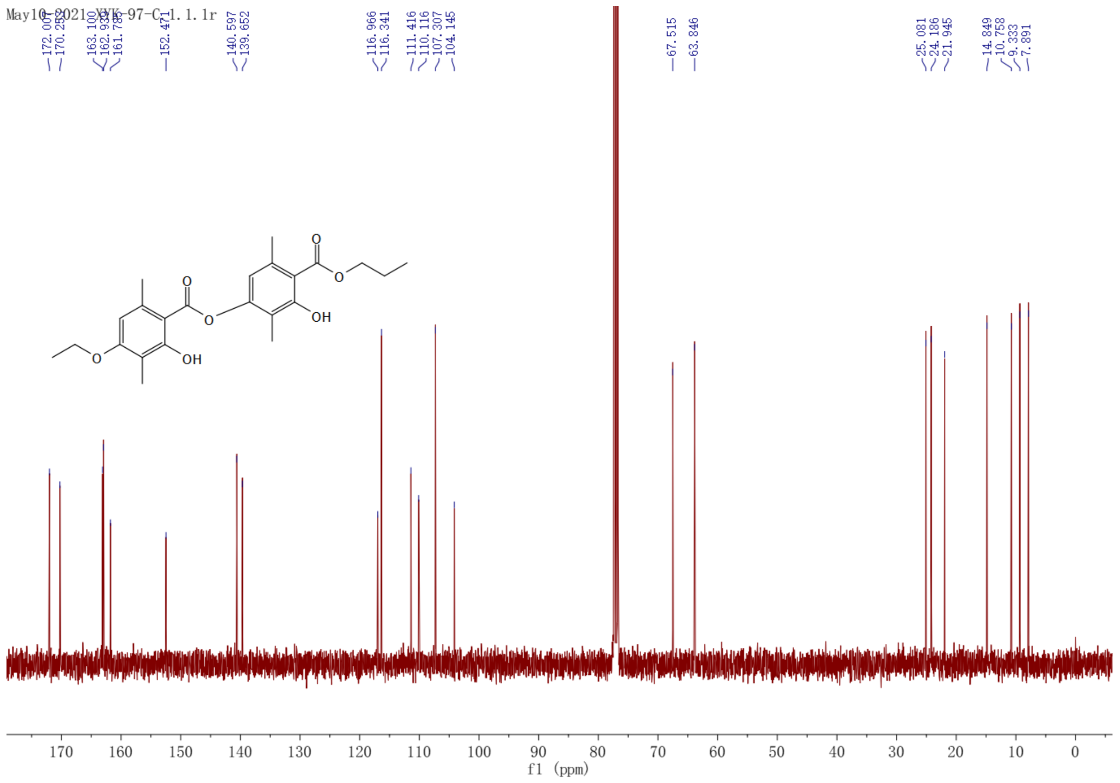

Figure S30. ^{13}C NMR spectrum of compound **6j**

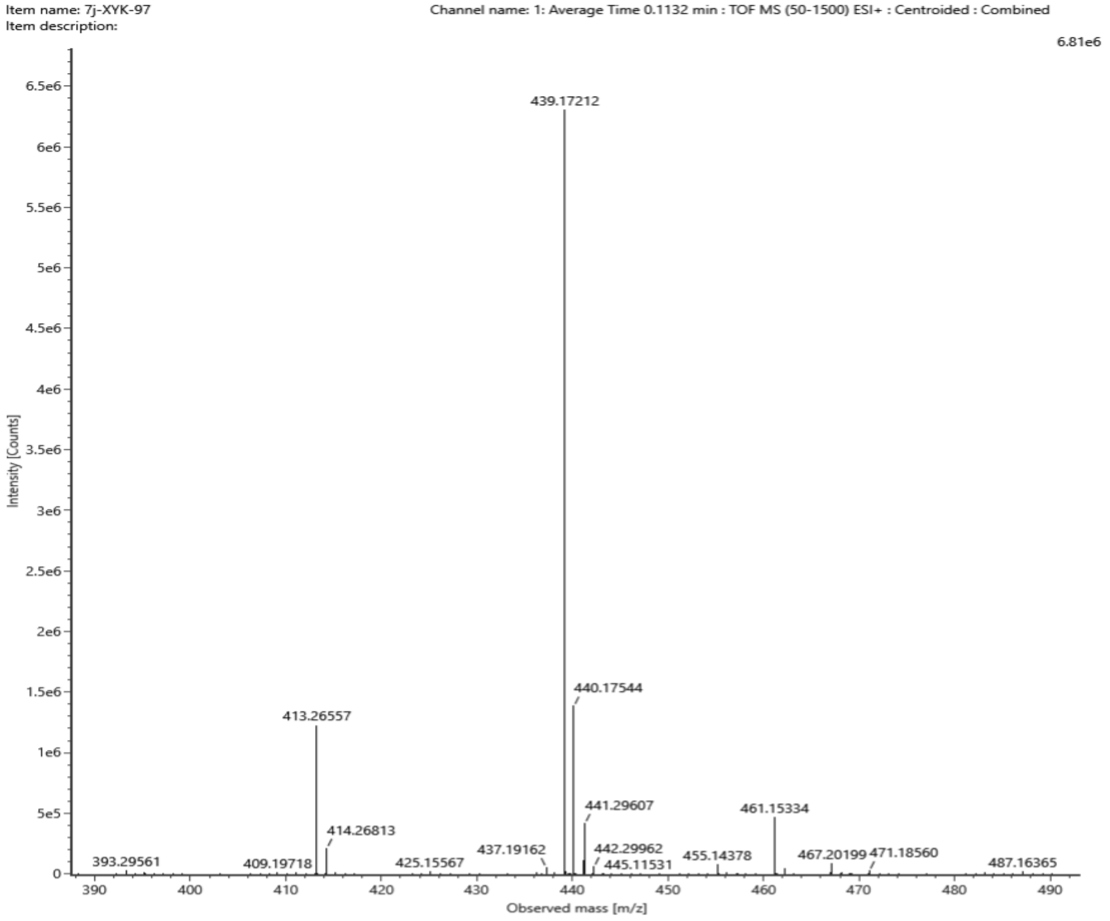

Figure S31. HRMS spectrum of compound **6j**

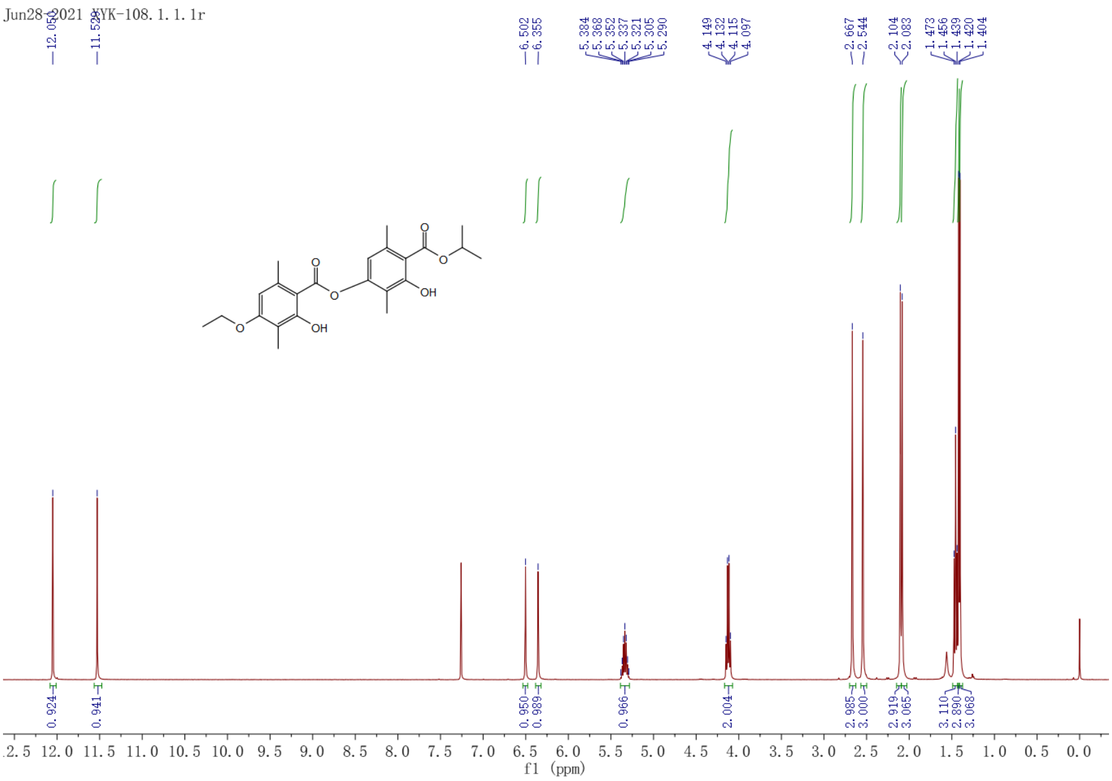

Figure S32. ^{1}H NMR spectrum of compound **6k**

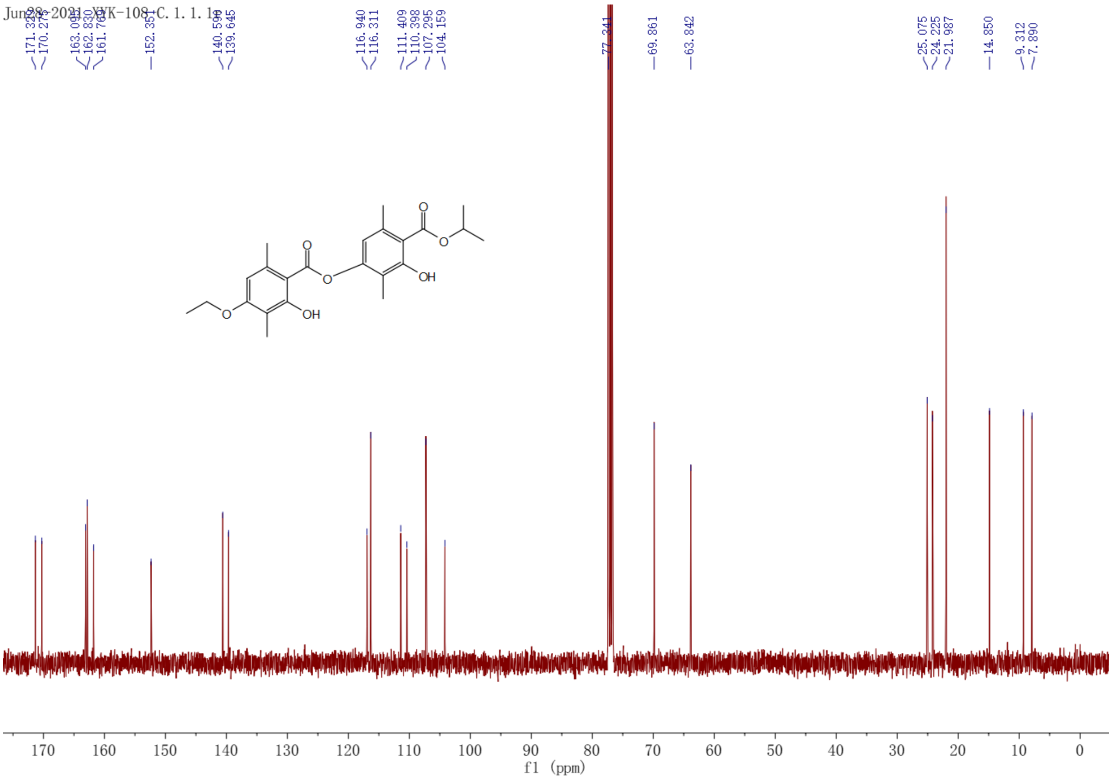

Figure S33. ^{13}C NMR spectrum of compound **6k**

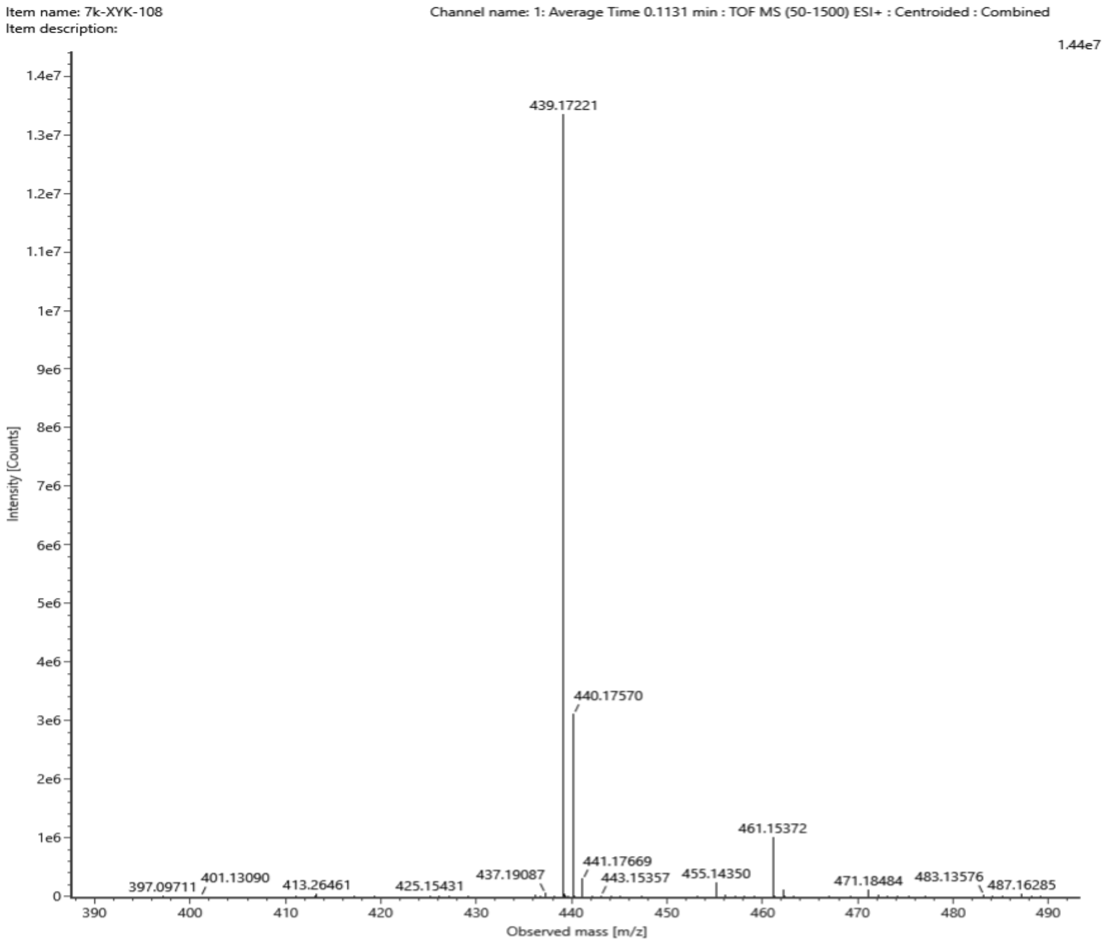

Figure S34. HRMS spectrum of compound **6k**

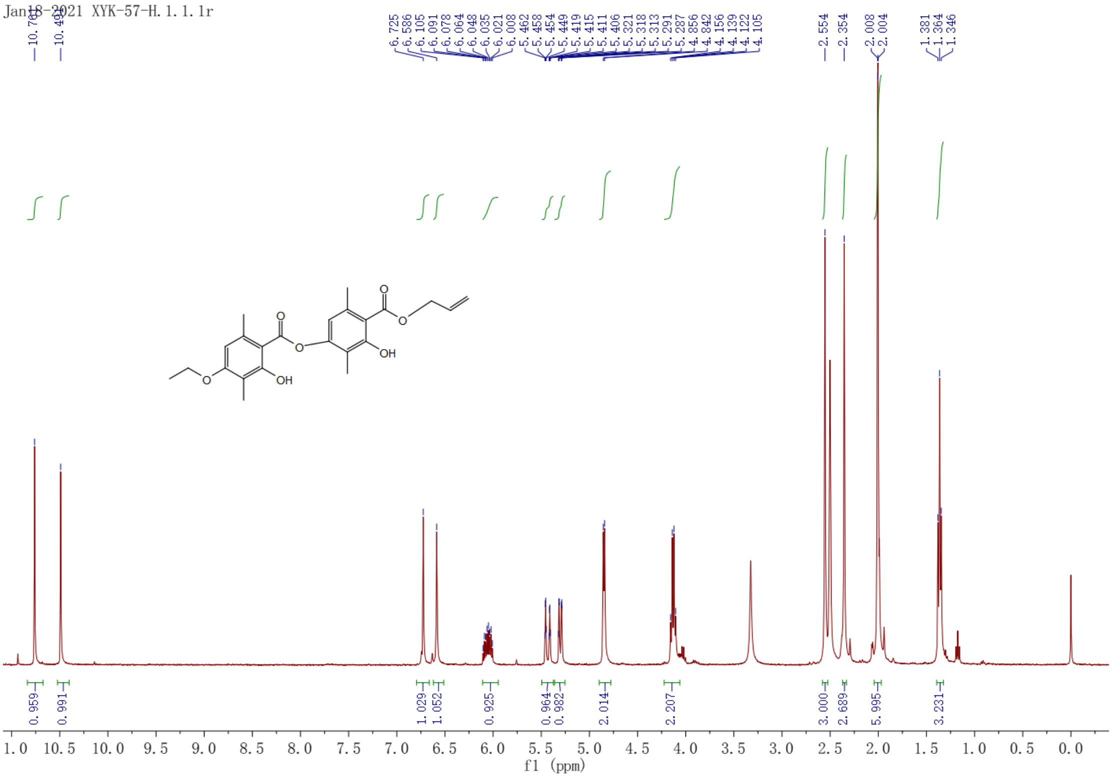

Figure S35. ^{1}H NMR spectrum of compound **6l**

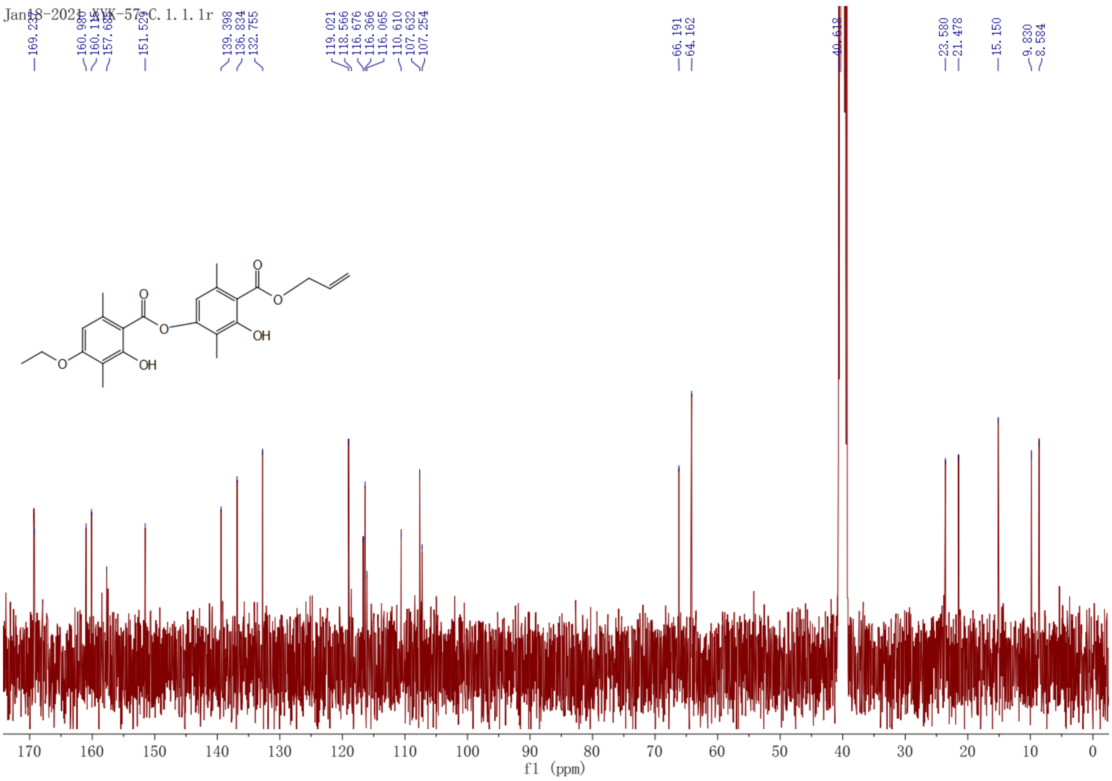

Figure S36. ^{13}C NMR spectrum of compound **6l**

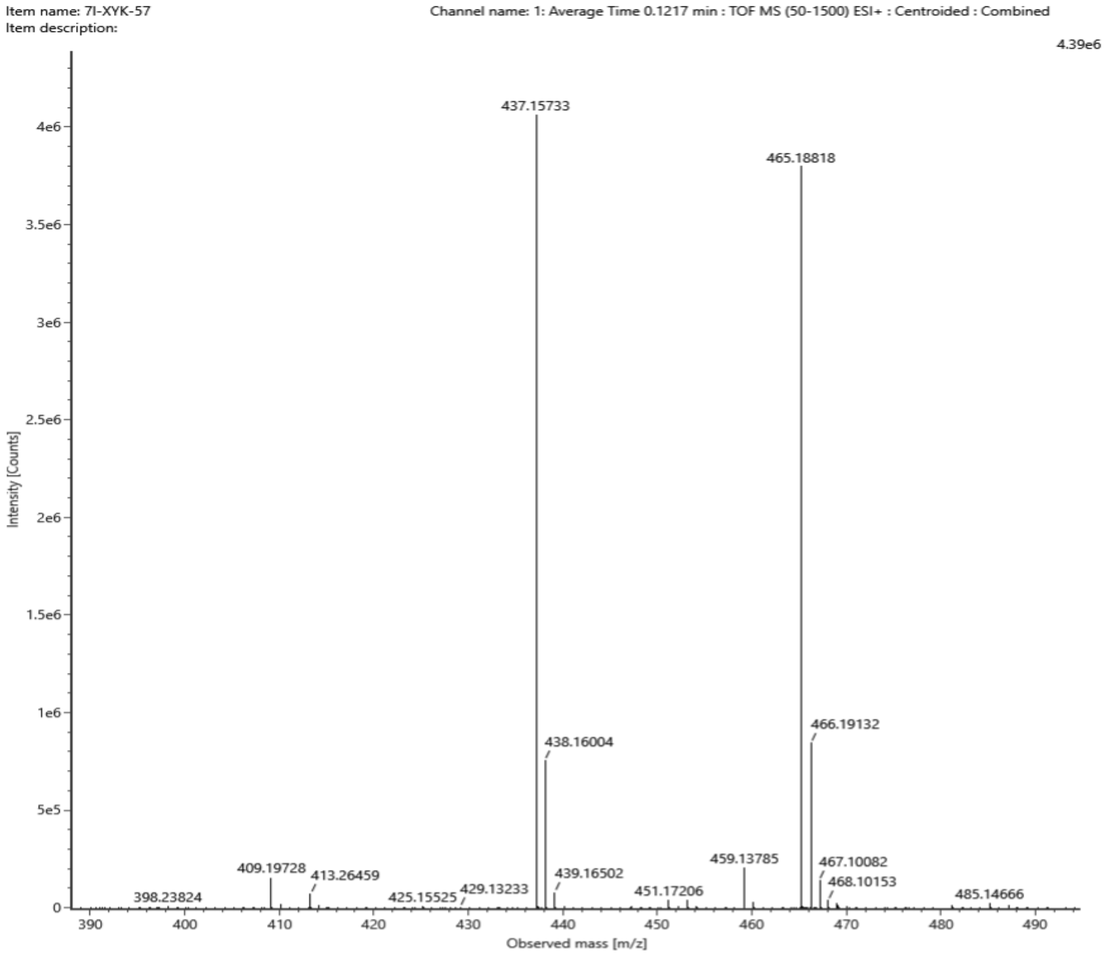

Figure S37. HRMS spectrum of compound **6l**

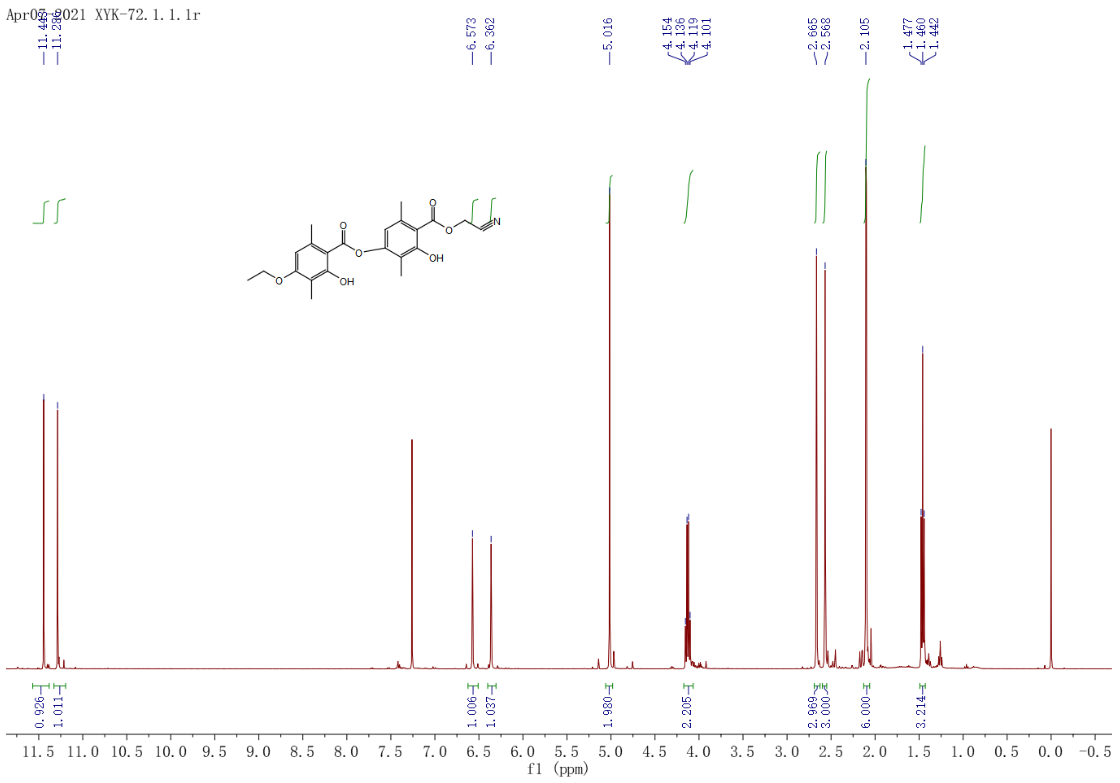

Figure S38. ^{1}H NMR spectrum of compound **6m**

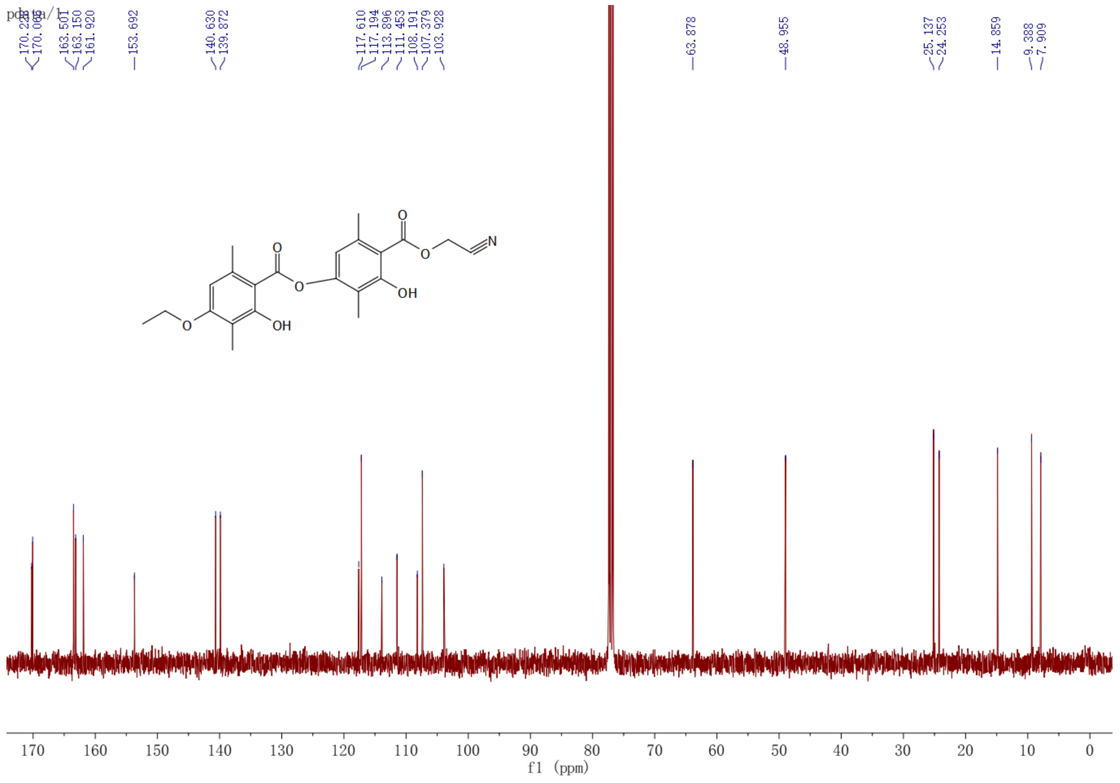

Figure S39. ^{13}C NMR spectrum of compound **6m**

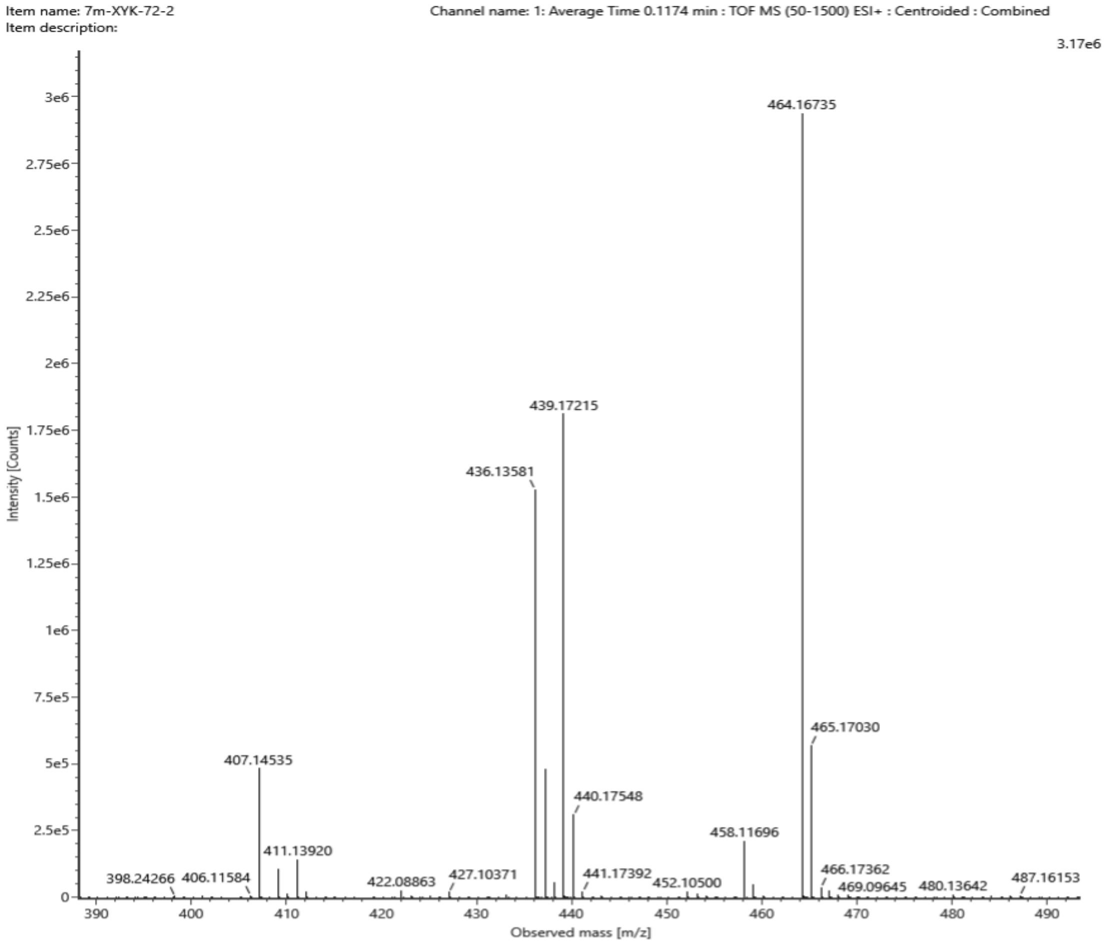

Figure S40. HRMS spectrum of compound **6m**

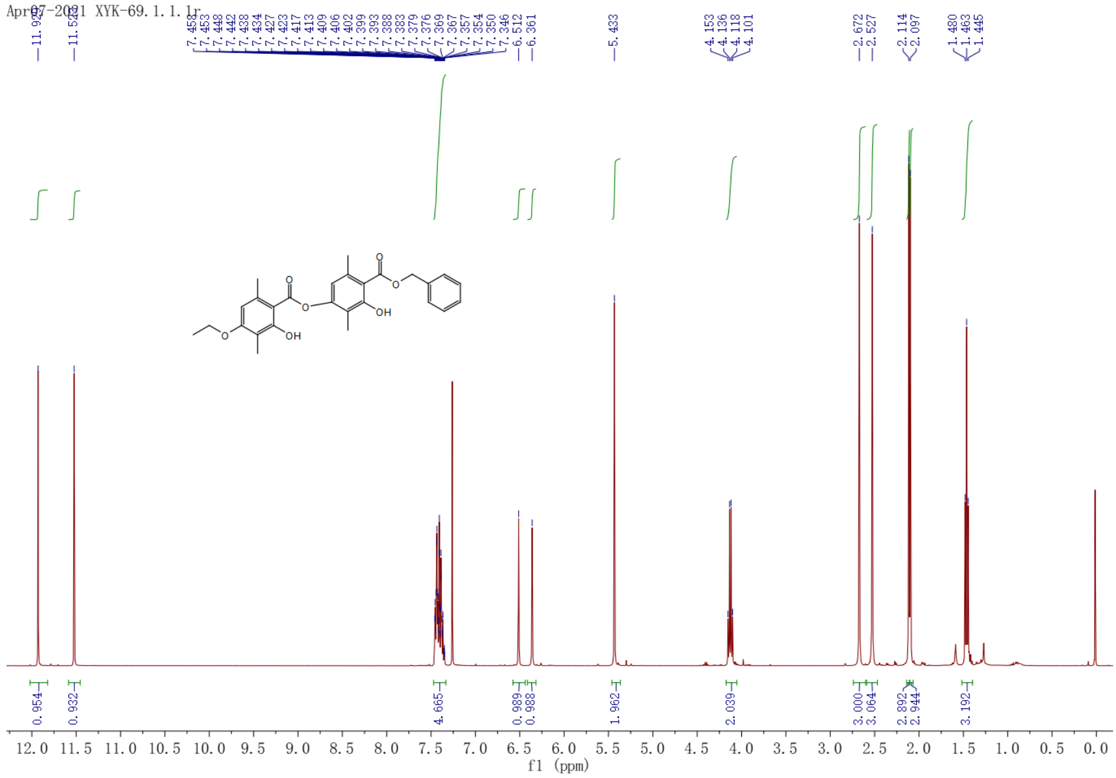

Figure S41. ^{1}H NMR spectrum of compound **6n**

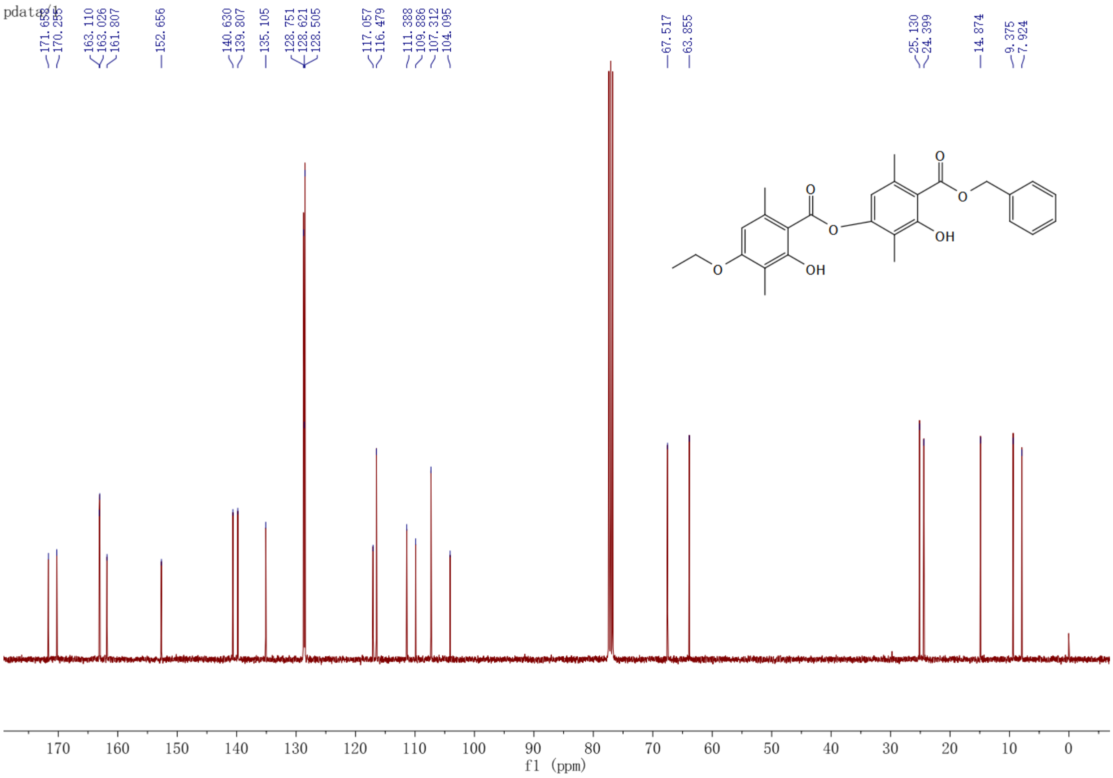

Figure S42. ^{13}C NMR spectrum of compound **6n**

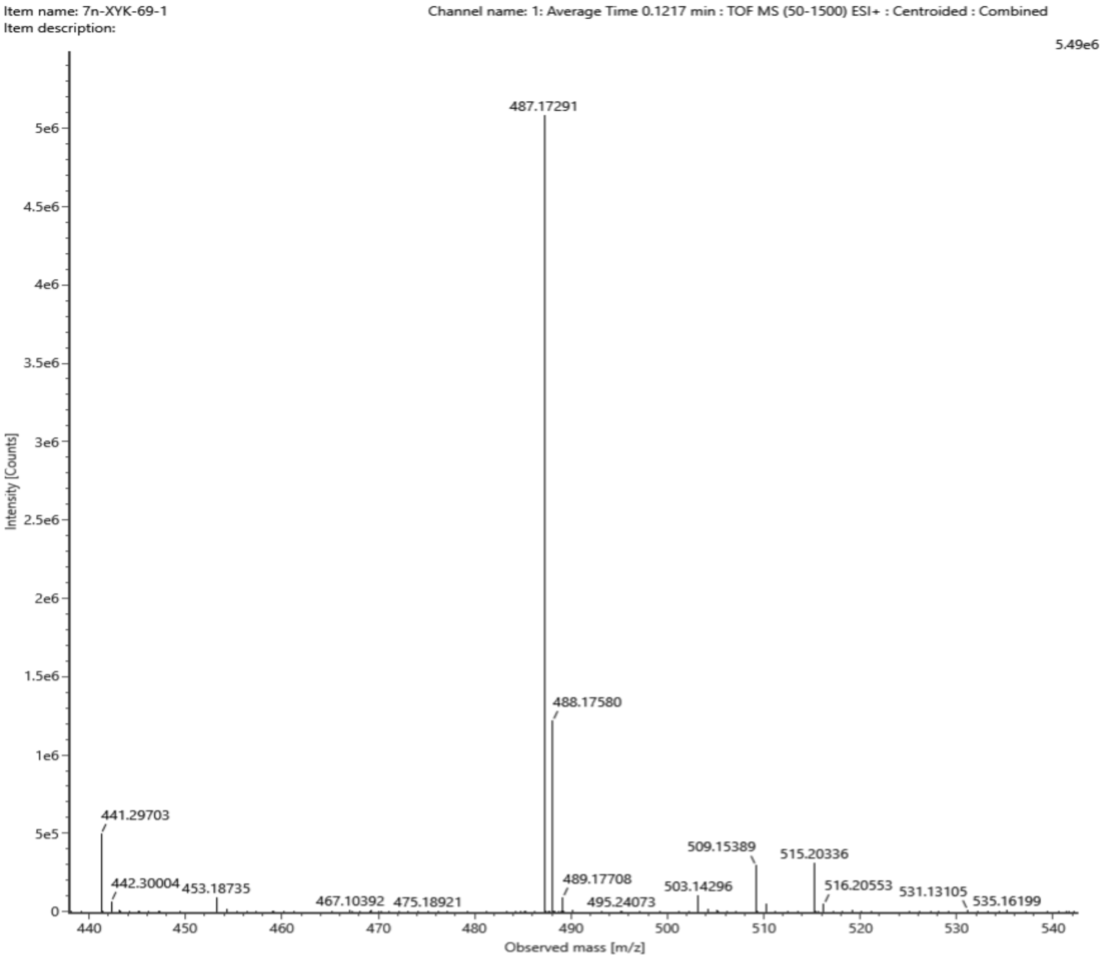

Figure S43. HRMS spectrum of compound **6n**

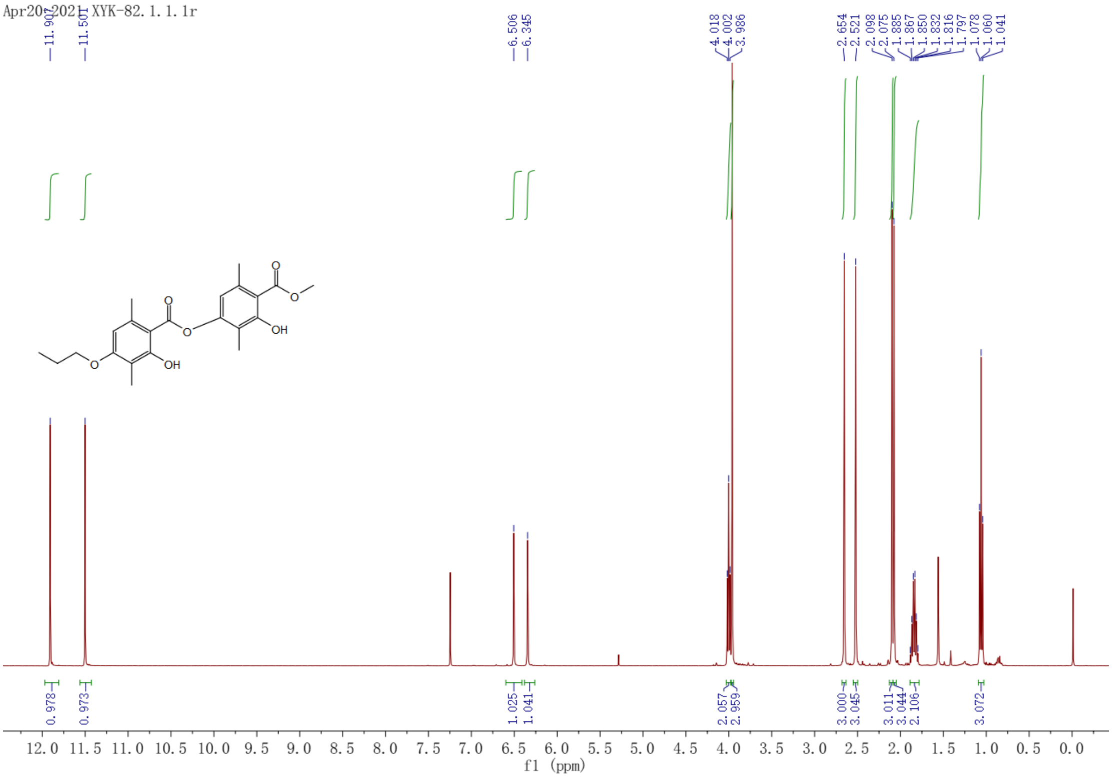

Figure S44. ^{1}H NMR spectrum of compound **6o**

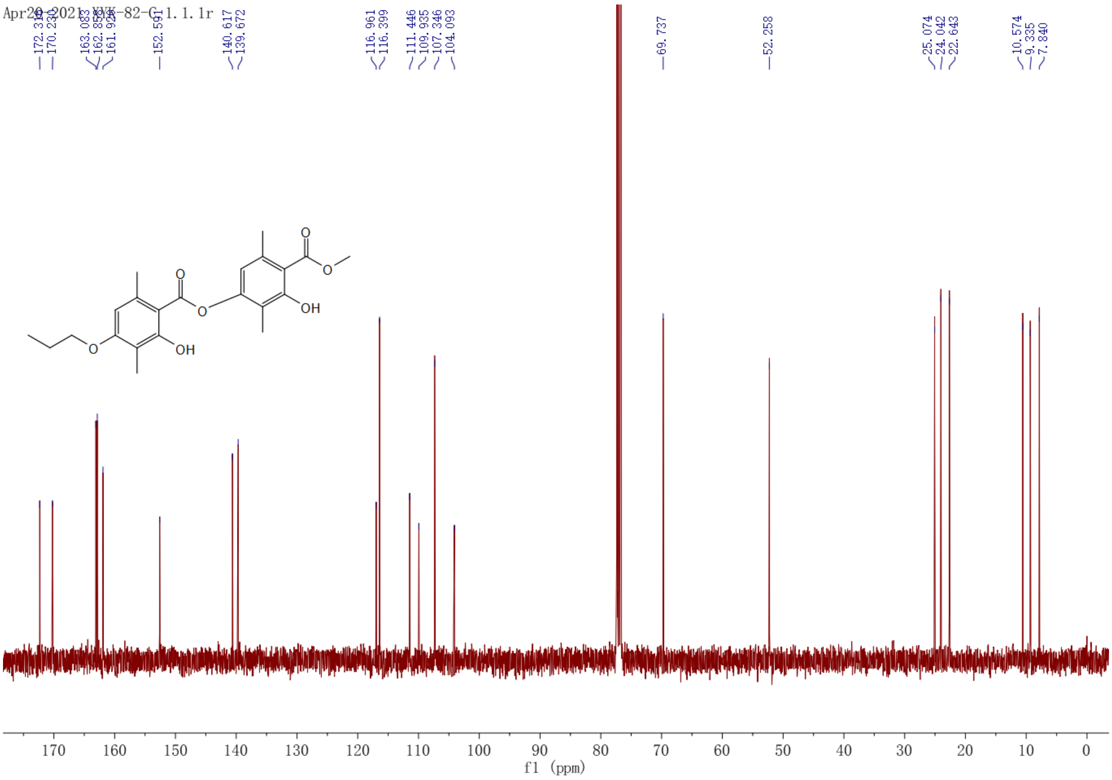

Figure S45. ^{13}C NMR spectrum of compound **6o**

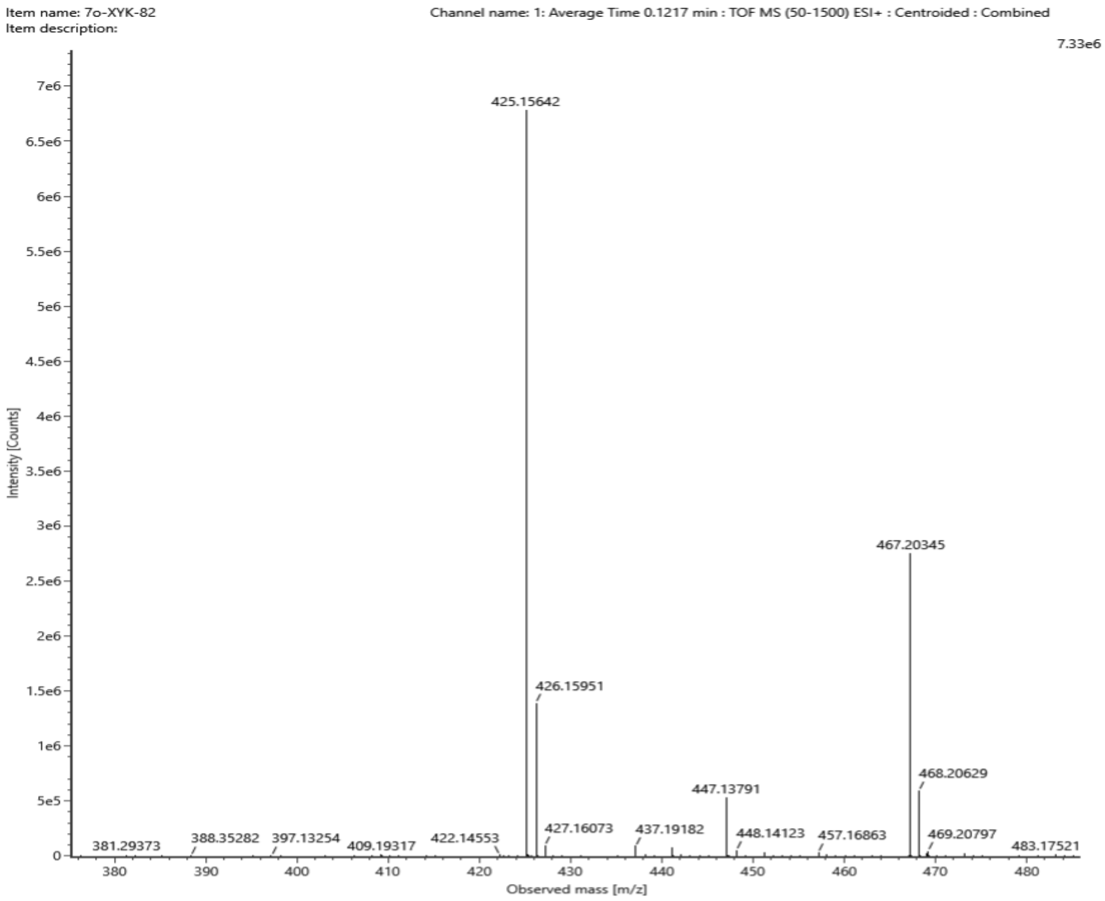

Figure S46. HRMS spectrum of compound **6o**

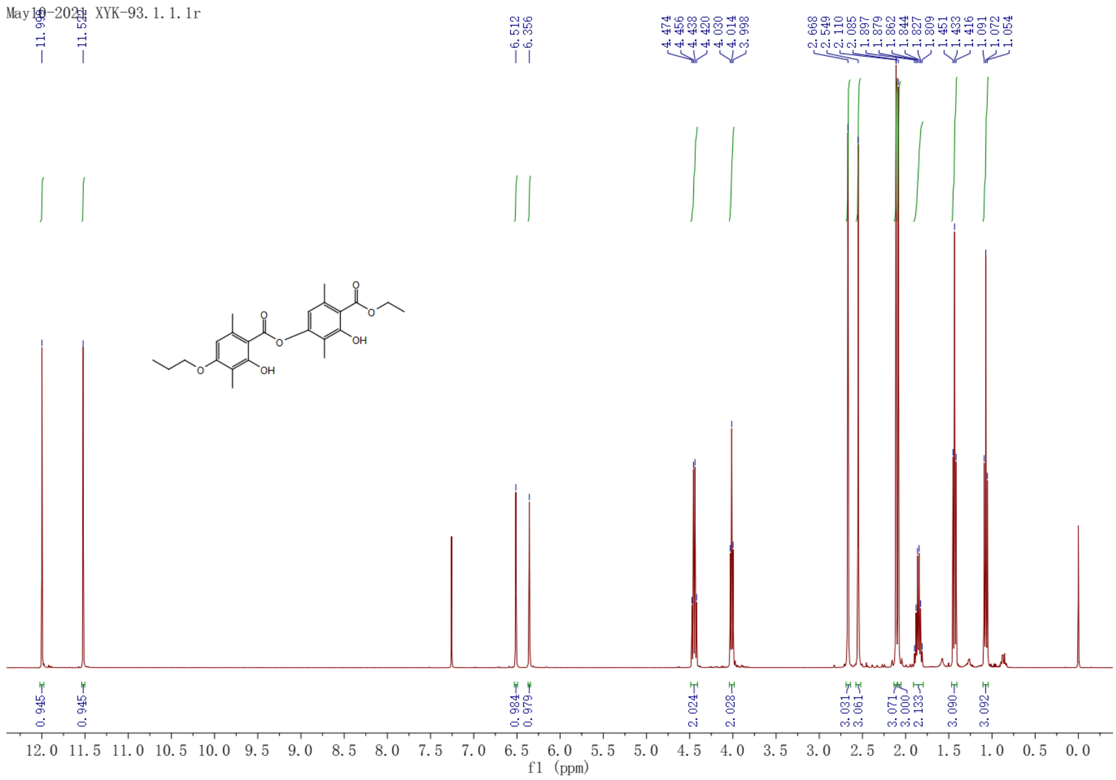

Figure S47. ^{1}H NMR spectrum of compound **6p**

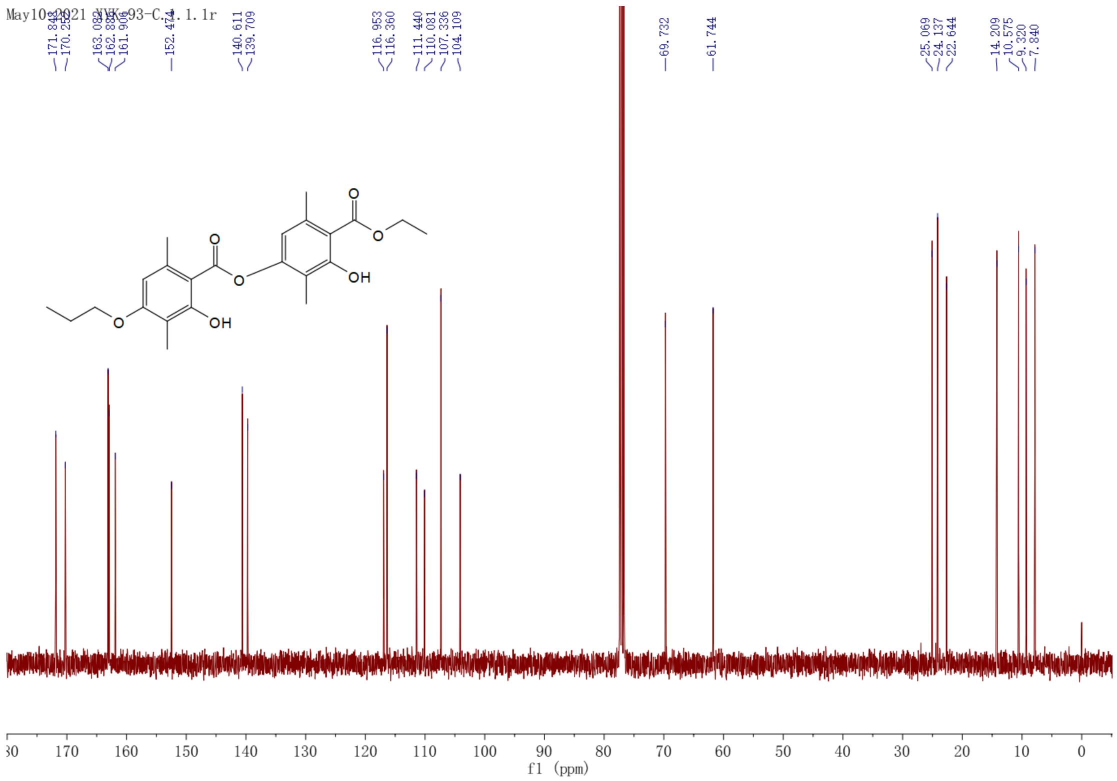

Figure S48. ^{13}C NMR spectrum of compound **6p**

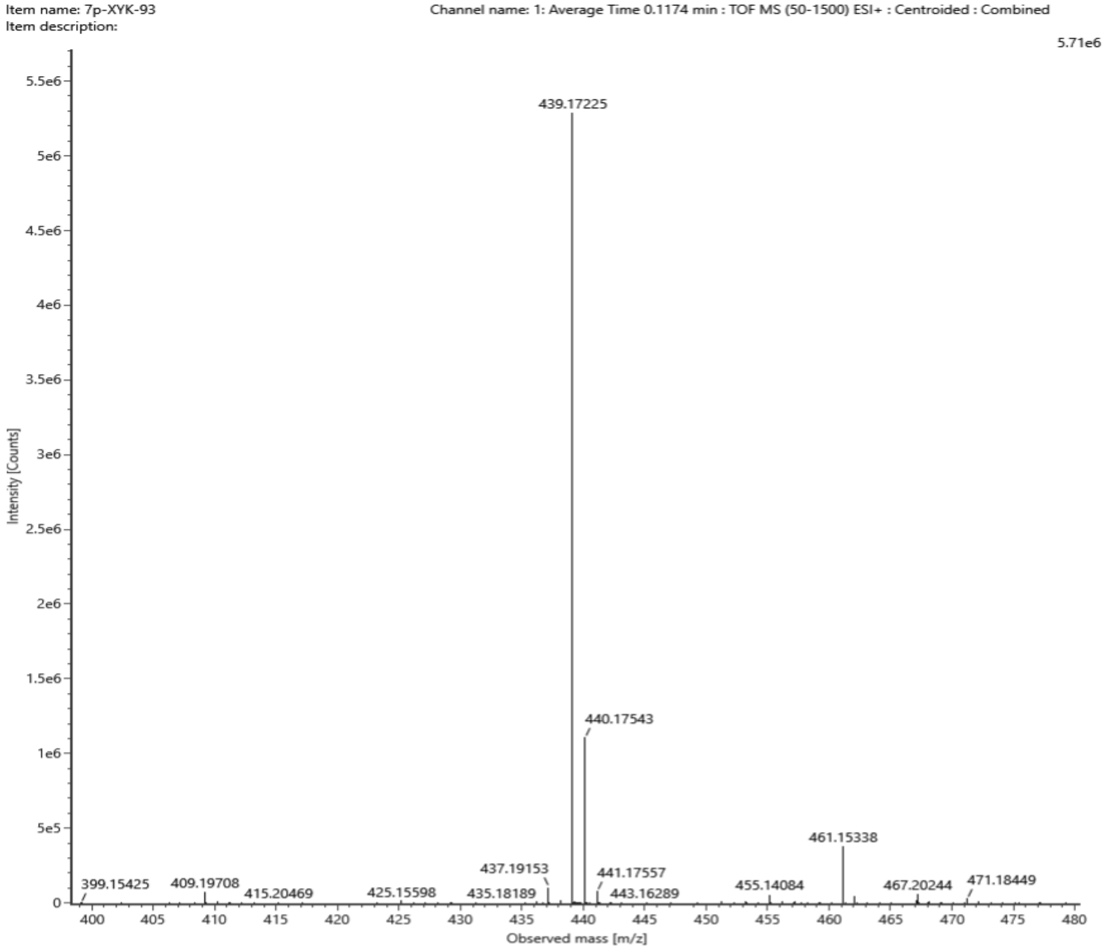

Figure S49. HRMS spectrum of compound **6p**

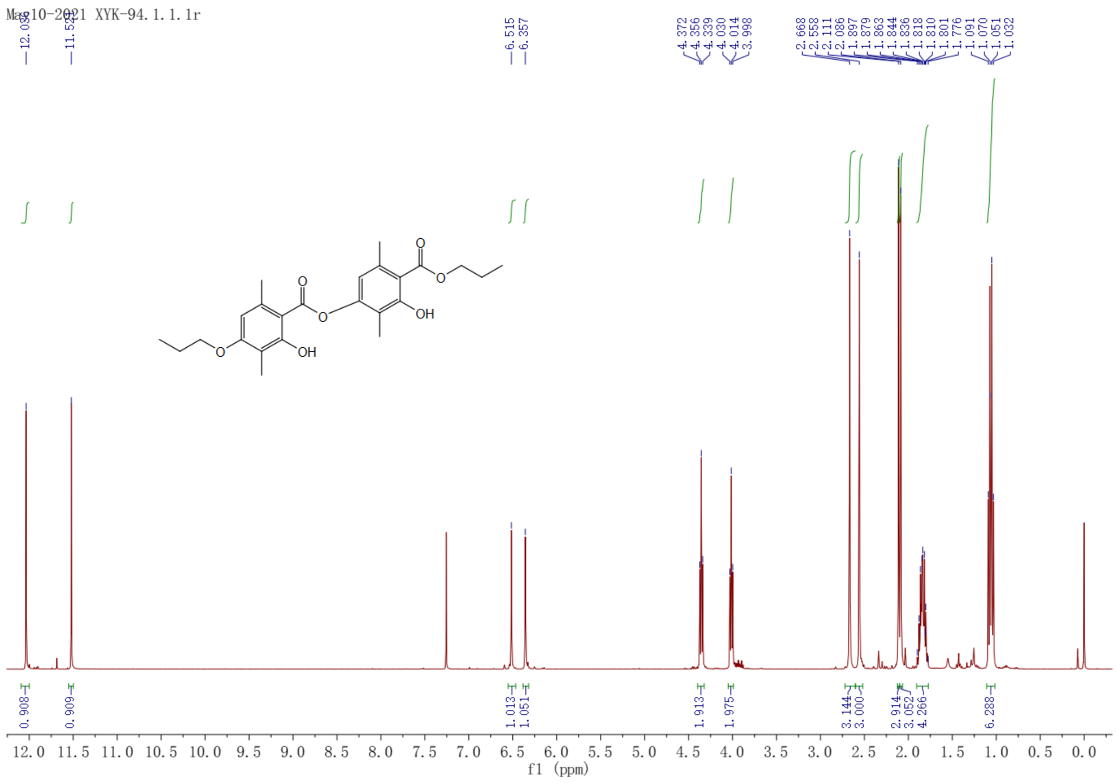

Figure S50. ^{1}H NMR spectrum of compound **6q**

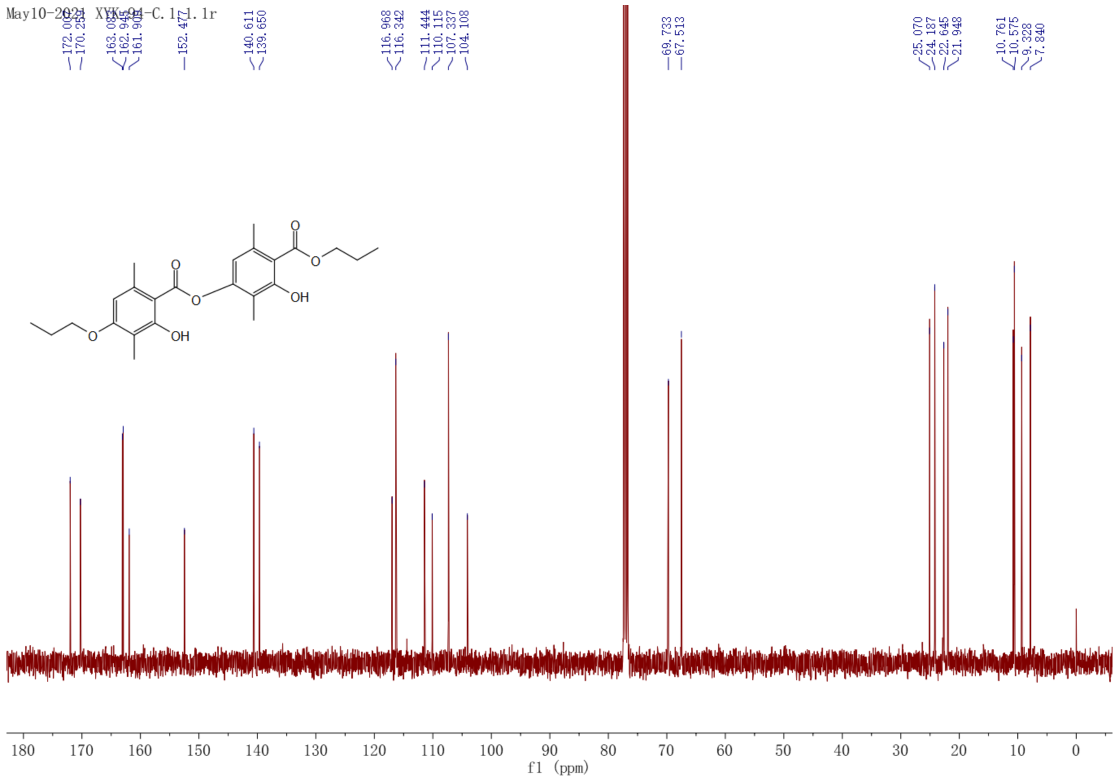

Figure S51. ^{13}C NMR spectrum of compound **6q**

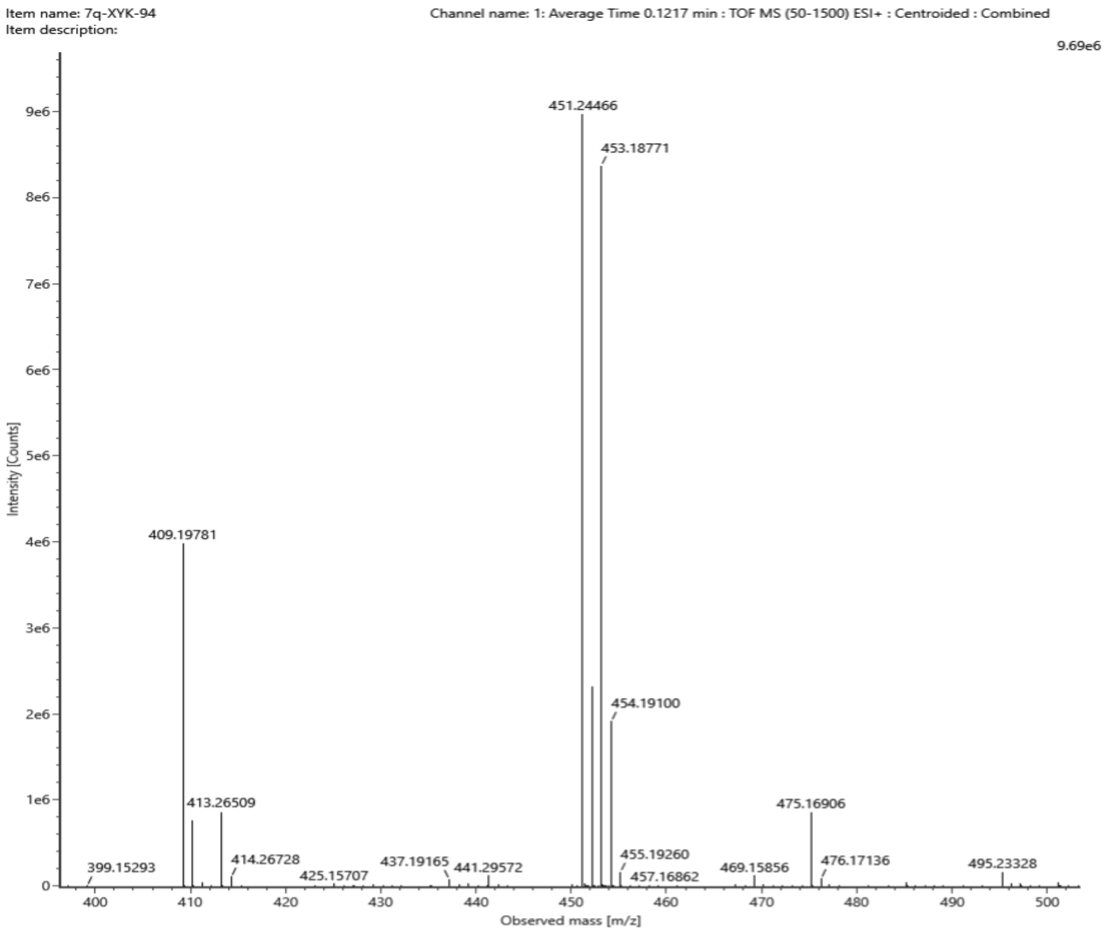

Figure S52. HRMS spectrum of compound **6q**

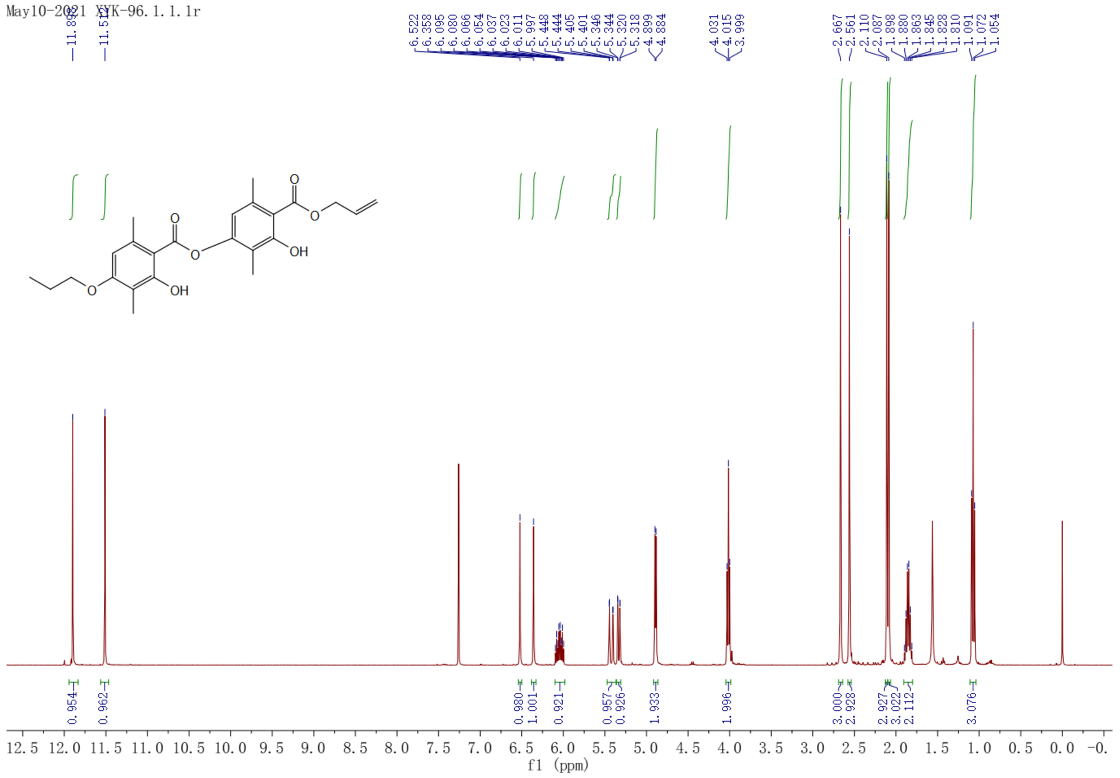

Figure S53. ^{1}H NMR spectrum of compound **6r**

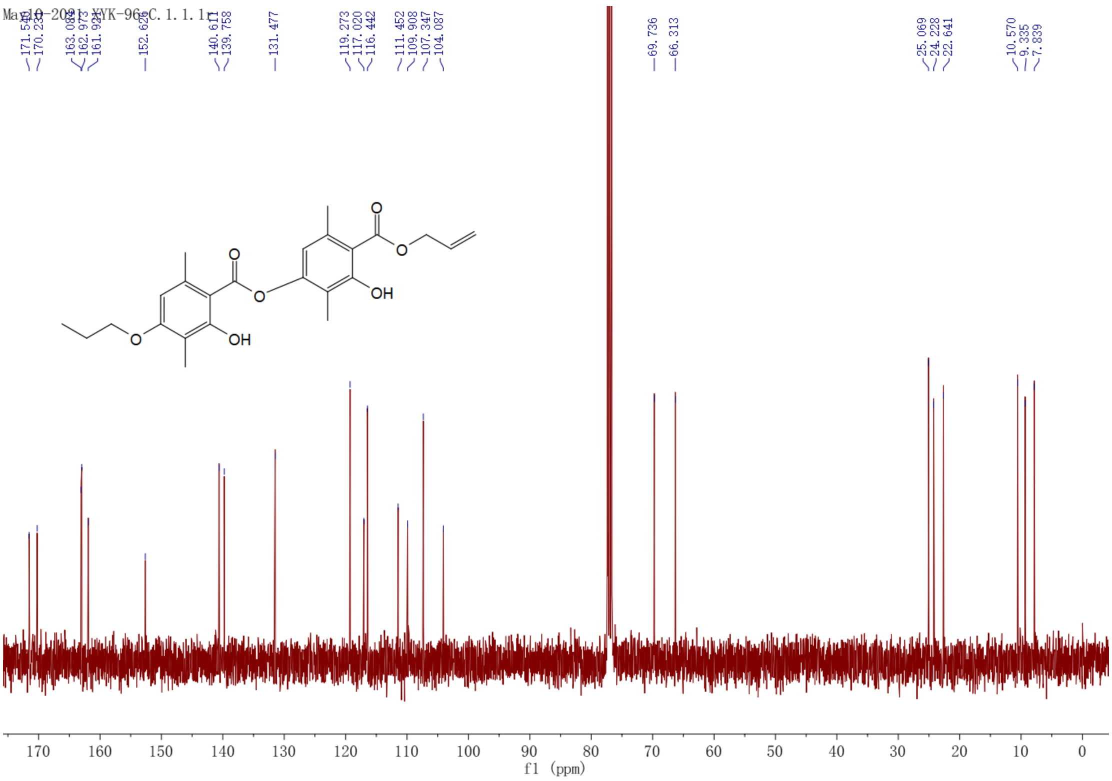

Figure S54. ^{13}C NMR spectrum of compound **6r**

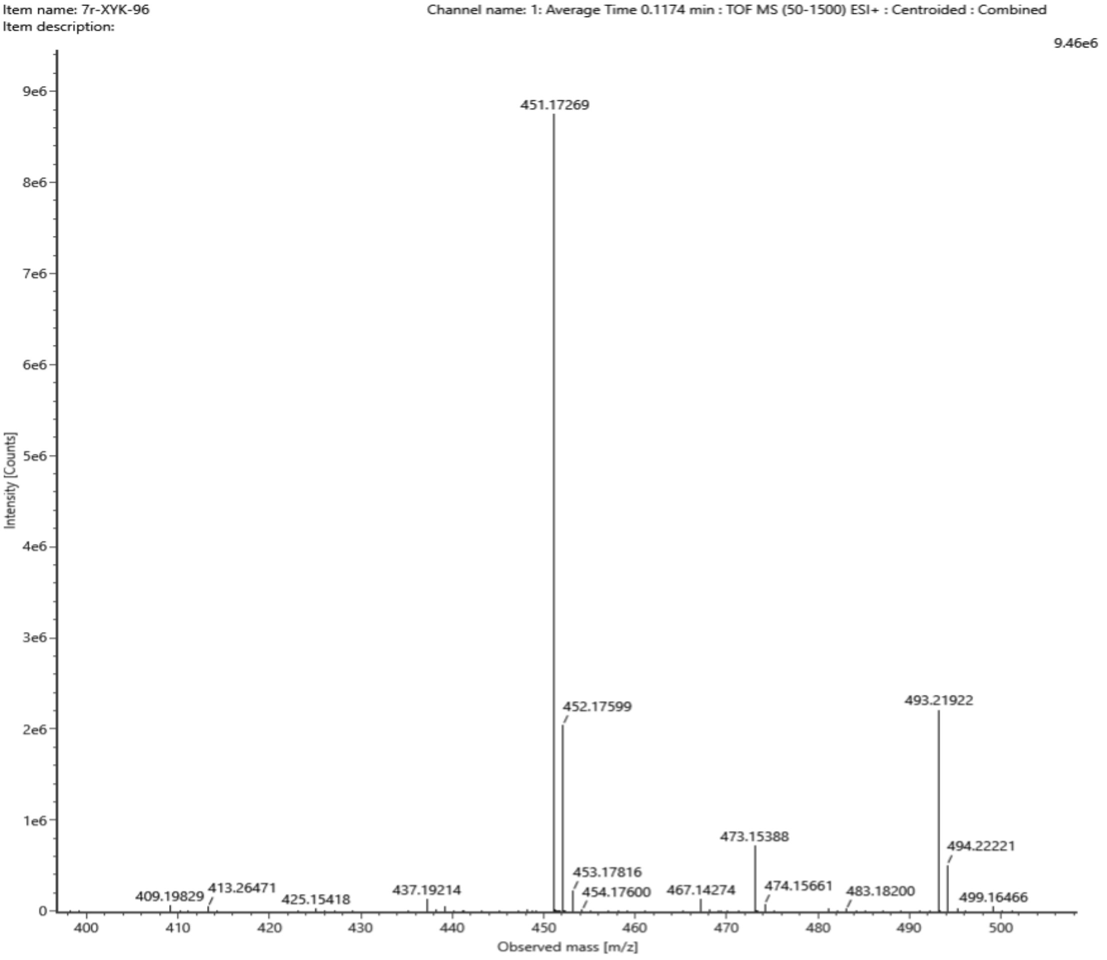

Figure S55. HRMS spectrum of compound **6r**

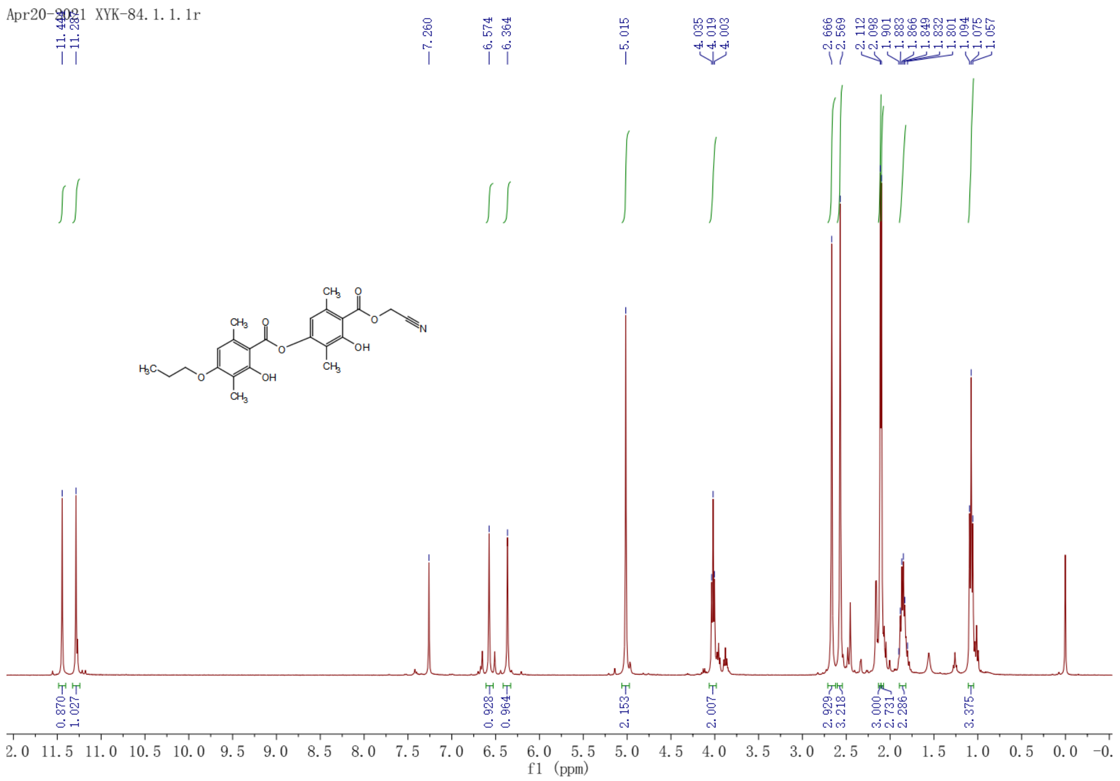

Figure S56. ^{1}H NMR spectrum of compound **6s**

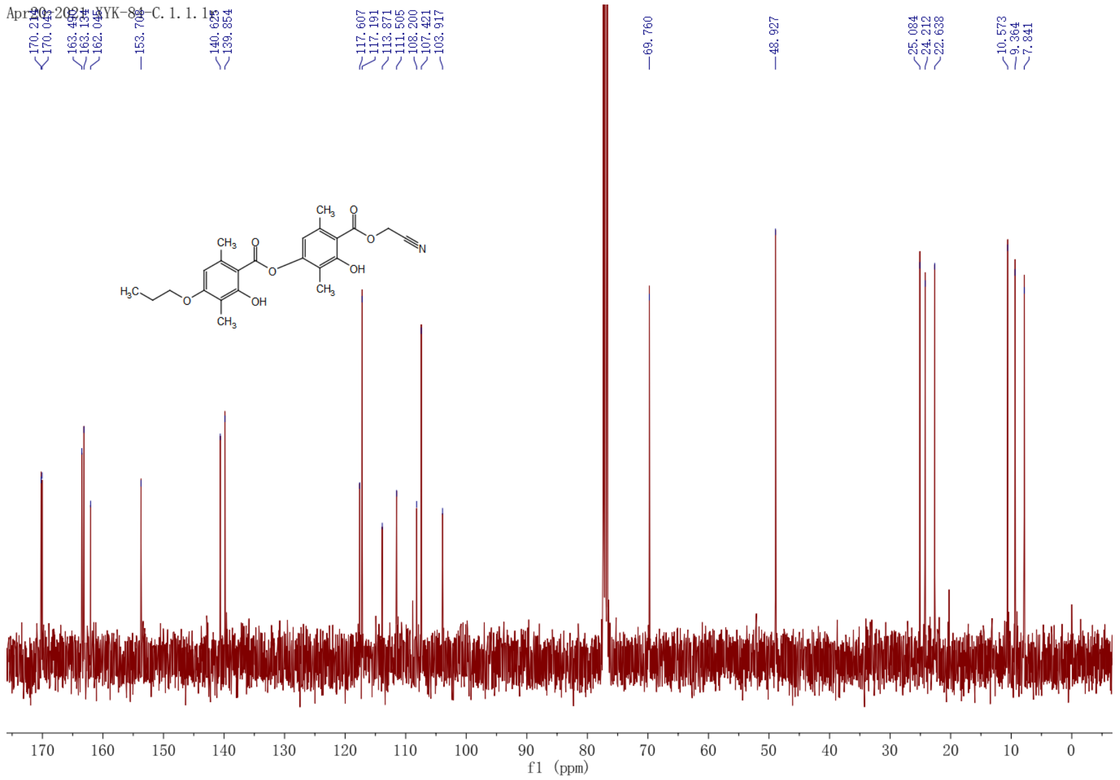

Figure S57. ^{13}C NMR spectrum of compound **6s**

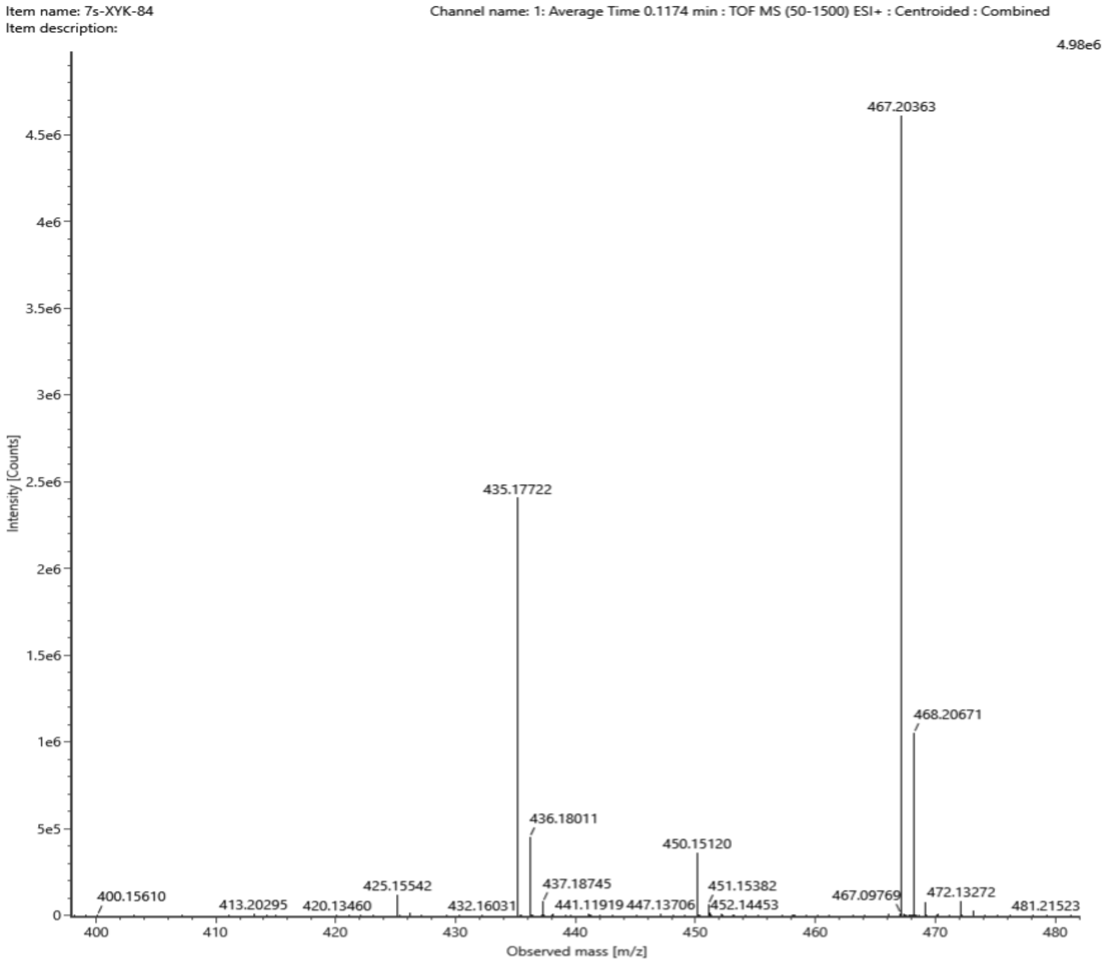

Figure S58. HRMS spectrum of compound **6s**

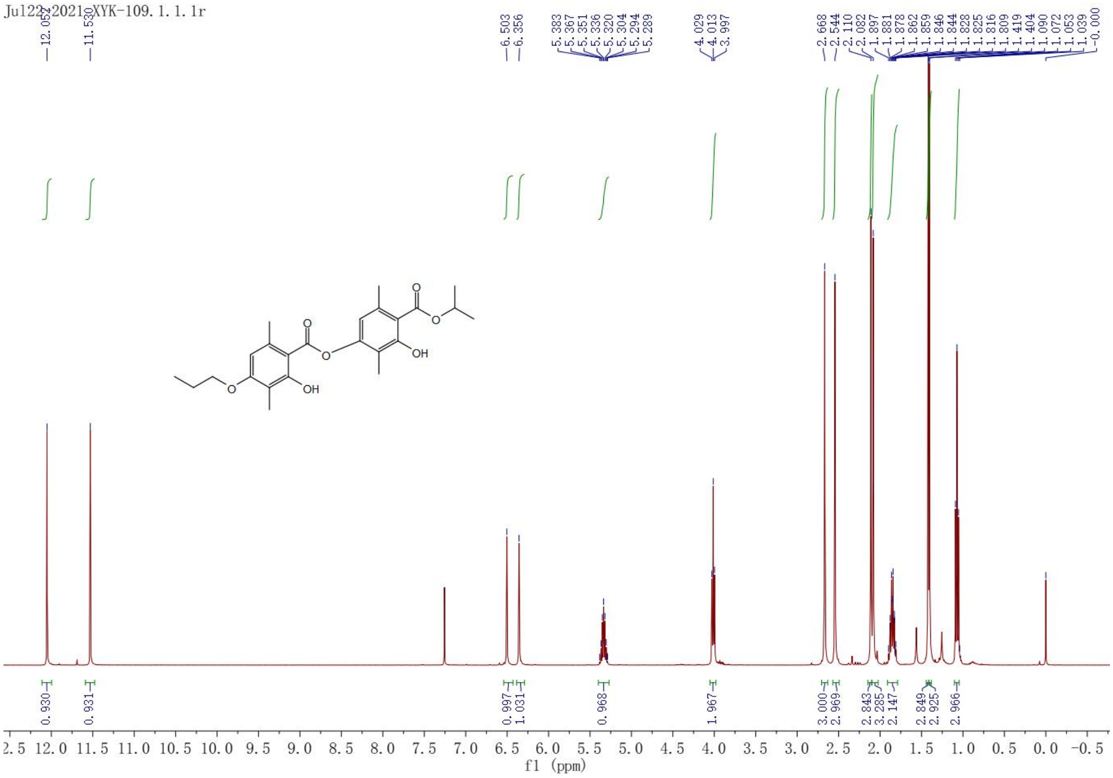

Figure S59. ^{1}H NMR spectrum of compound **6t**

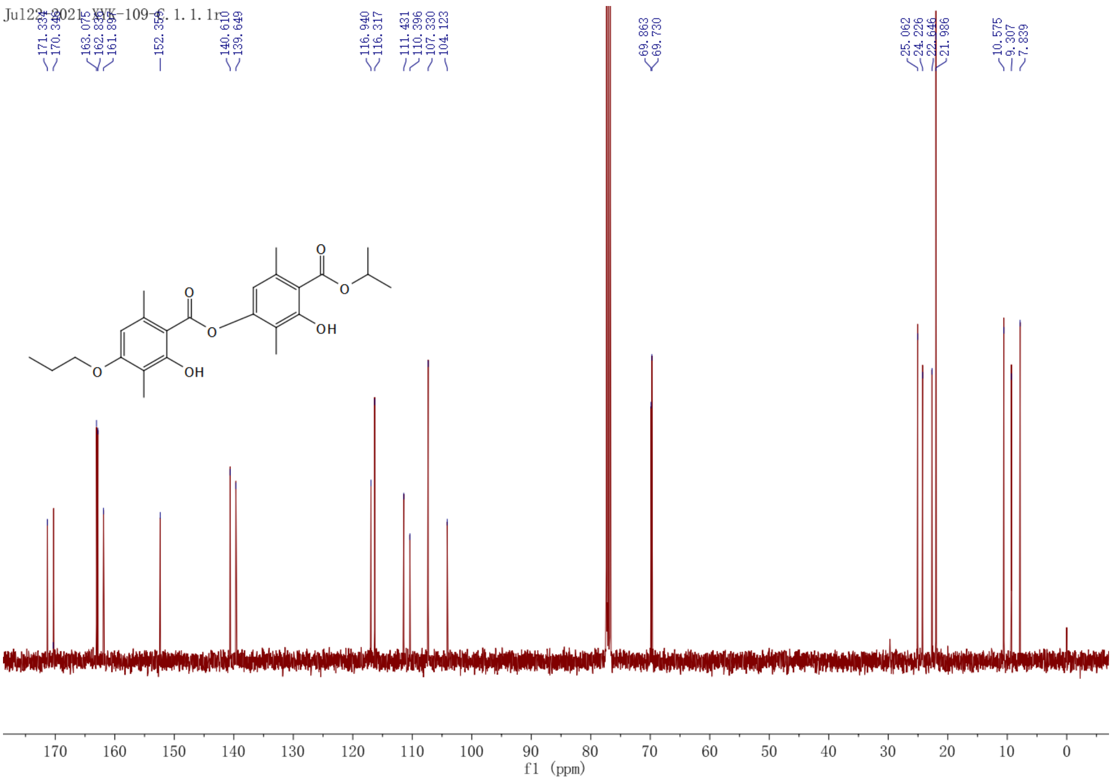

Figure S60. ^{13}C NMR spectrum of compound **6t**

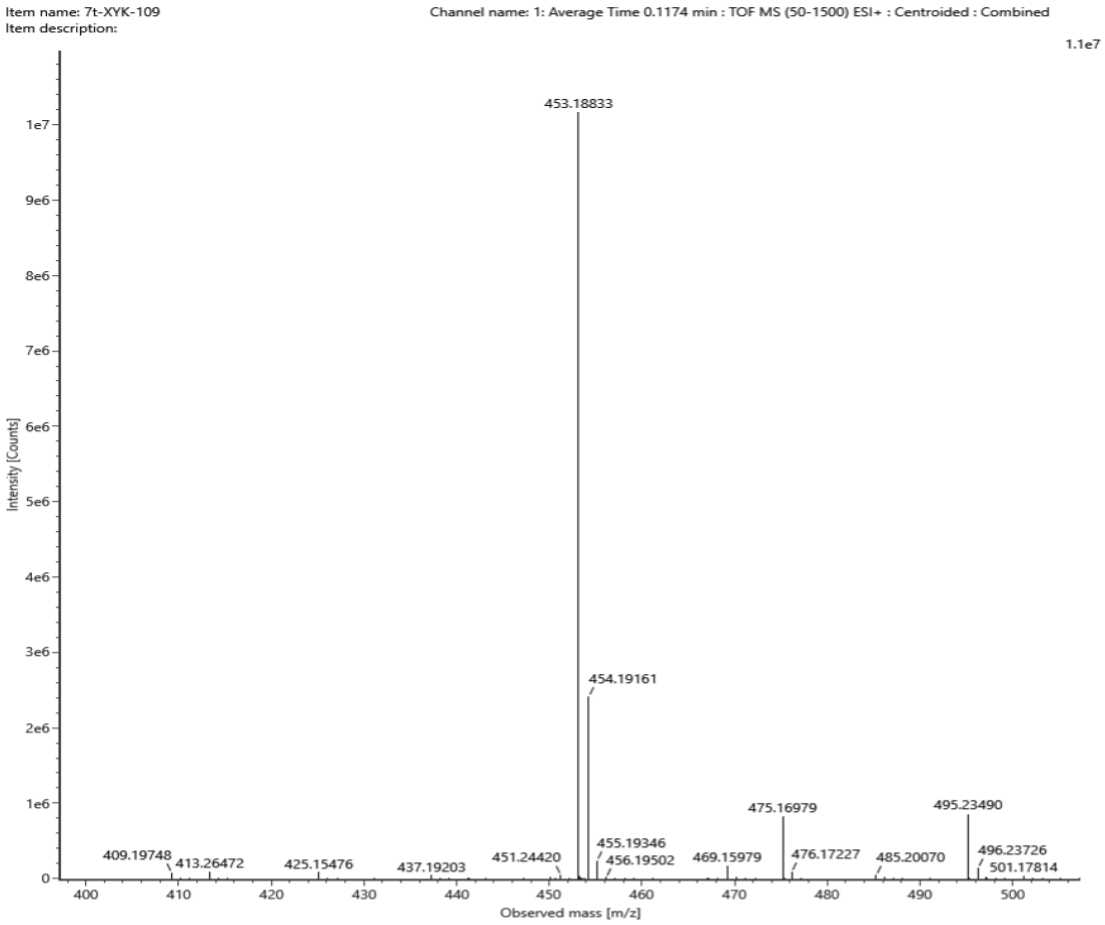

Figure S61. HRMS spectrum of compound **6t**

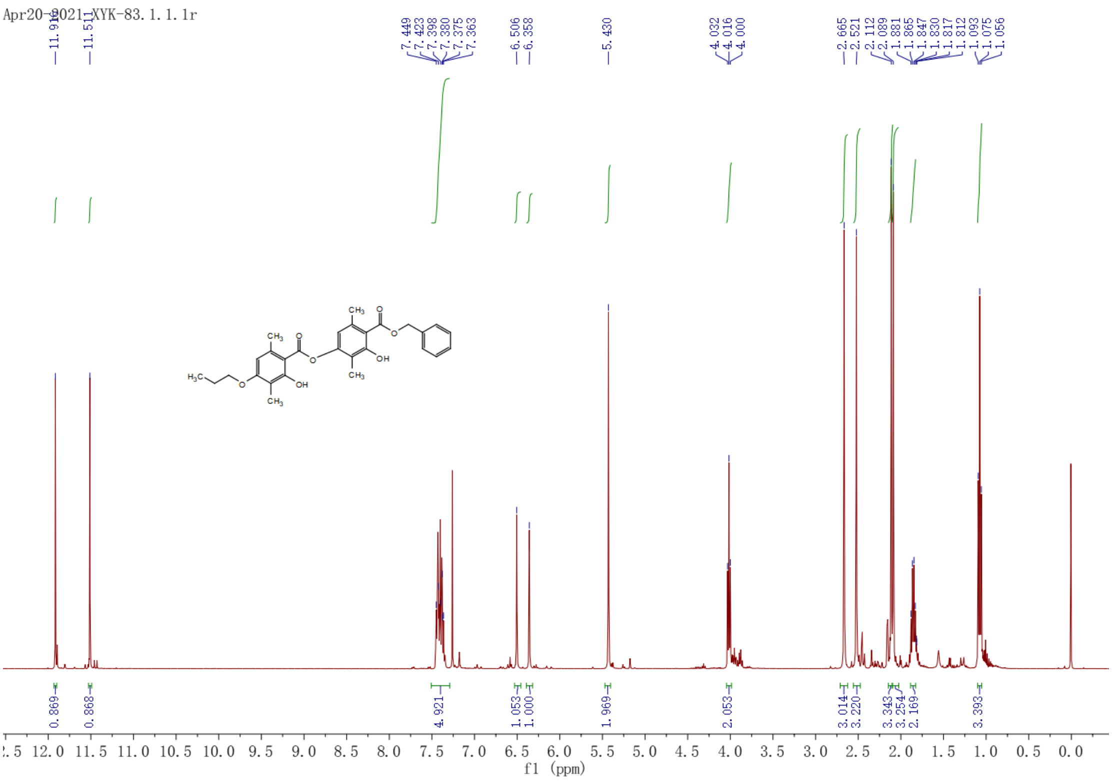

Figure S62. ^{1}H NMR spectrum of compound **6u**

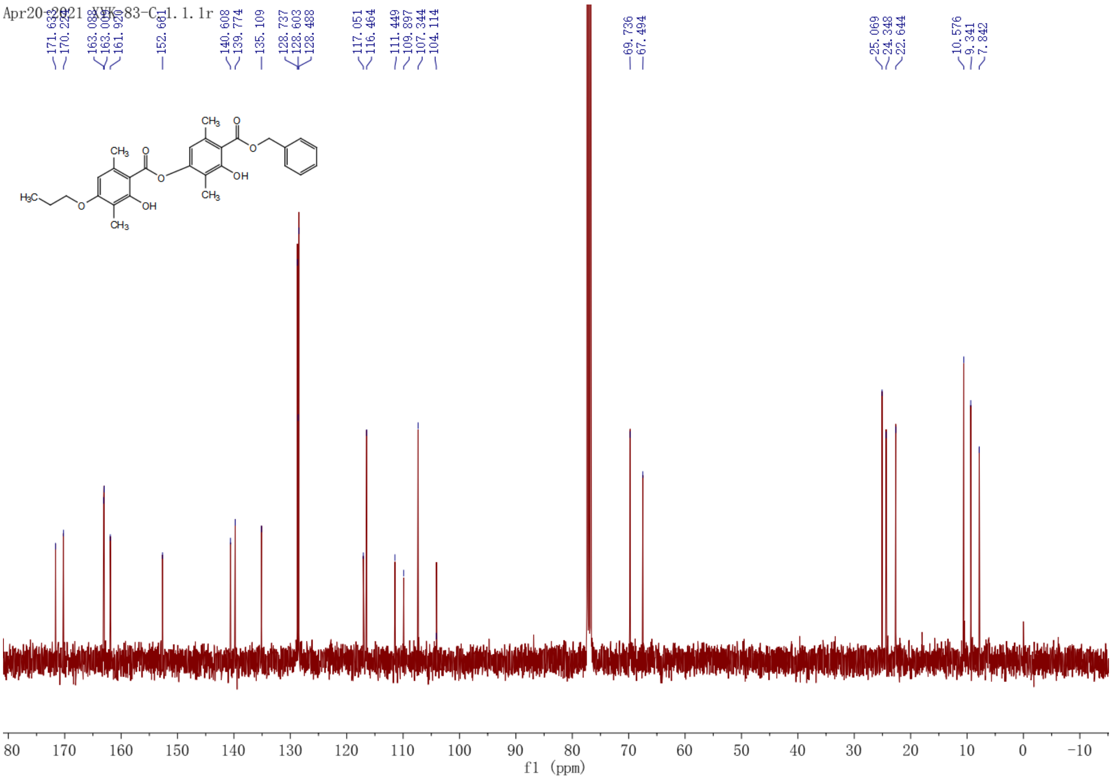

Figure S63. ^{13}C NMR spectrum of compound **6u**

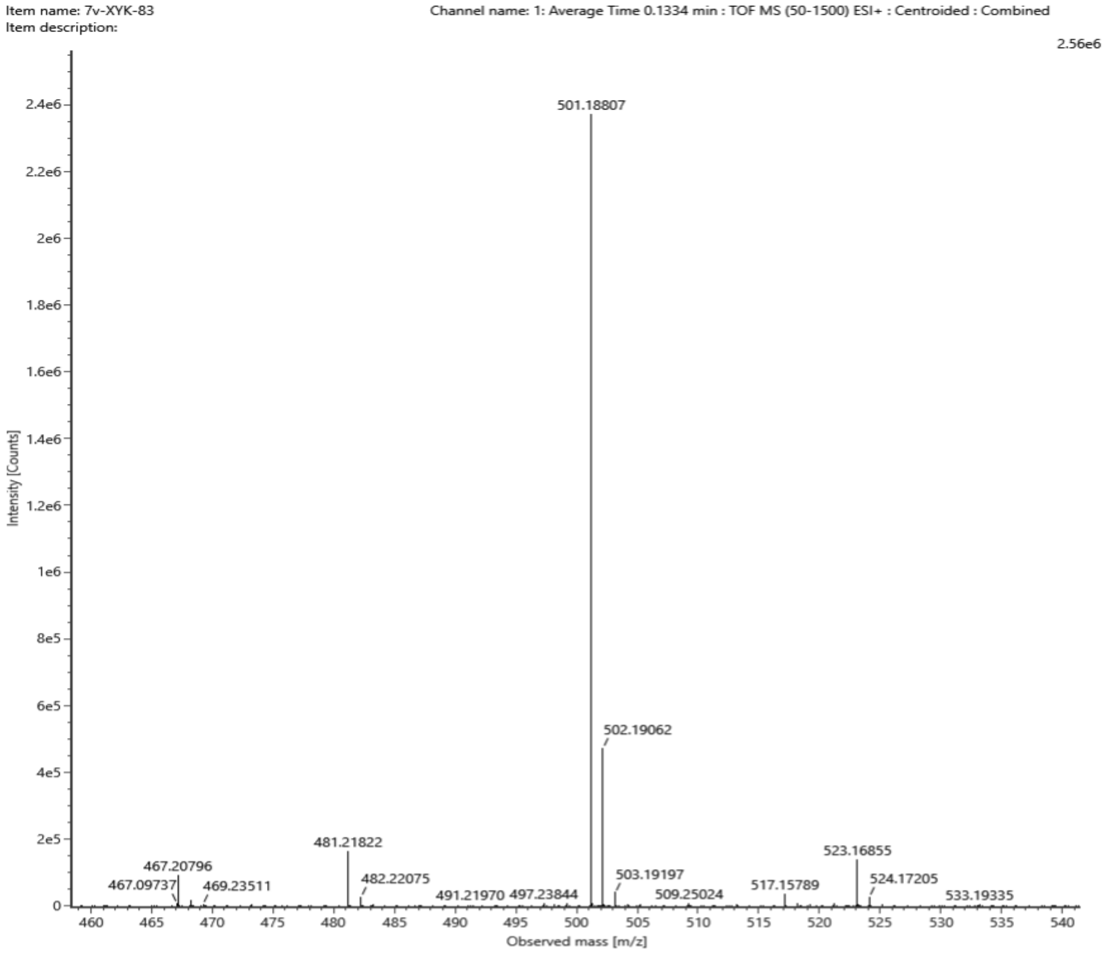

Figure S64. HRMS spectrum of compound **6u**

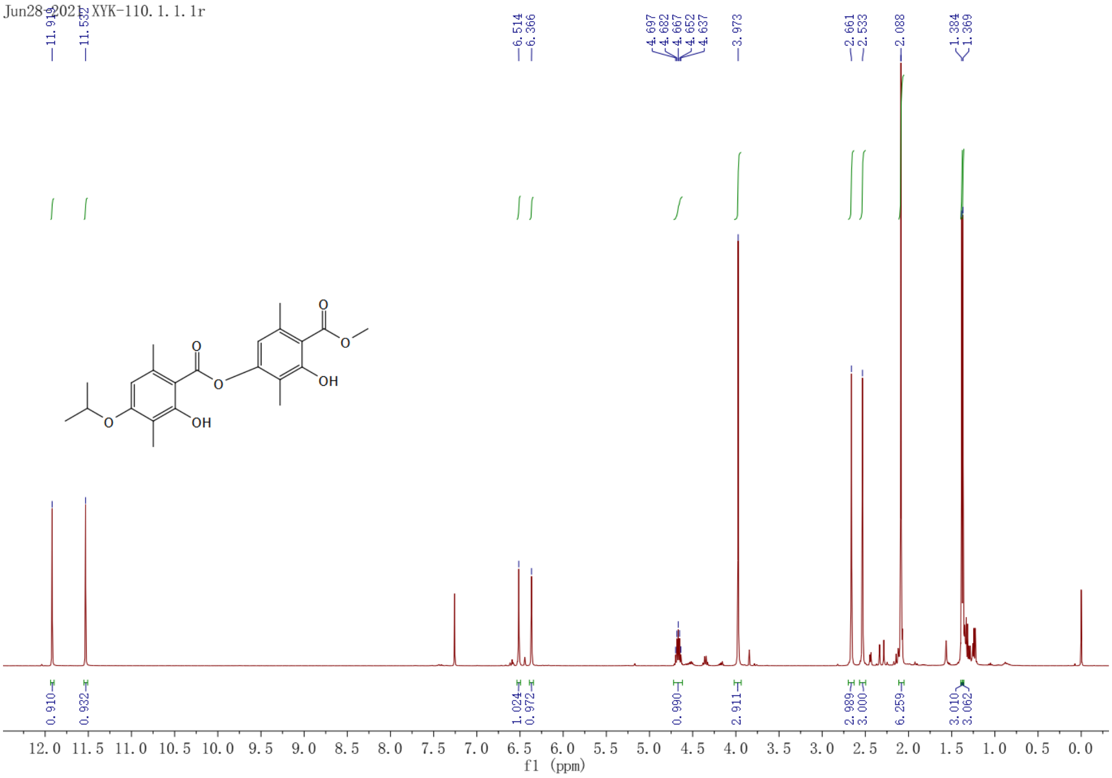

Figure S65. ^{1}H NMR spectrum of compound **6v**

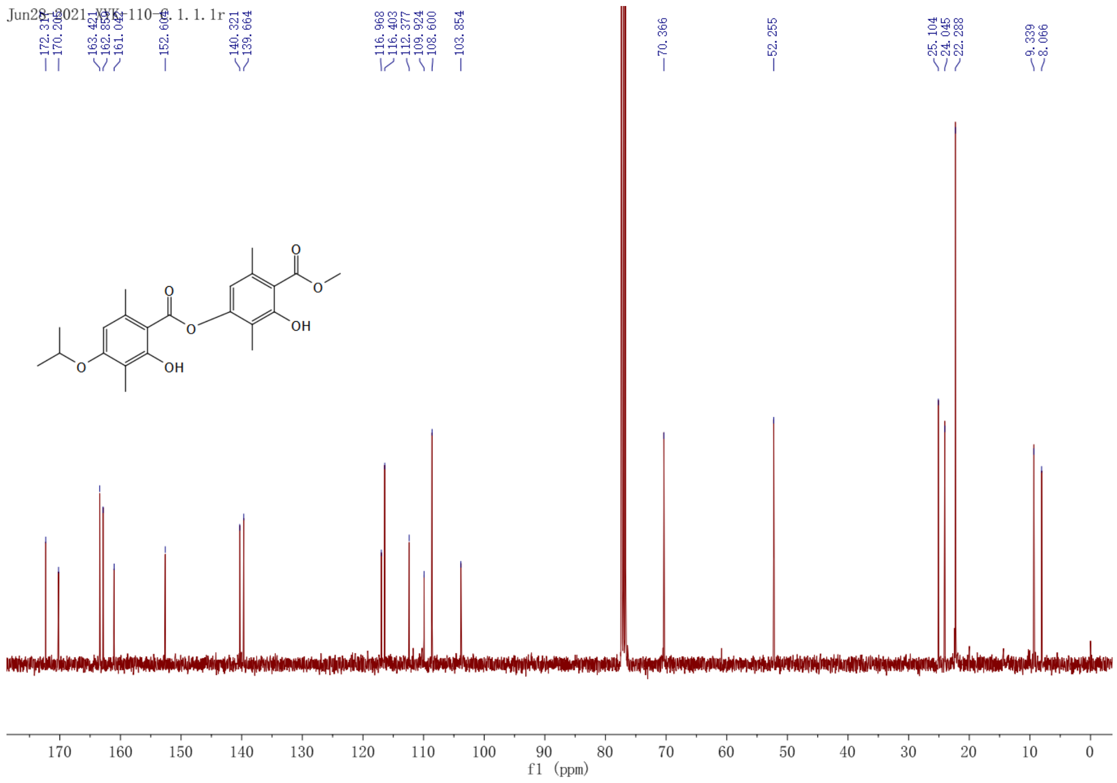

Figure S66. ^{13}C NMR spectrum of compound **6v**

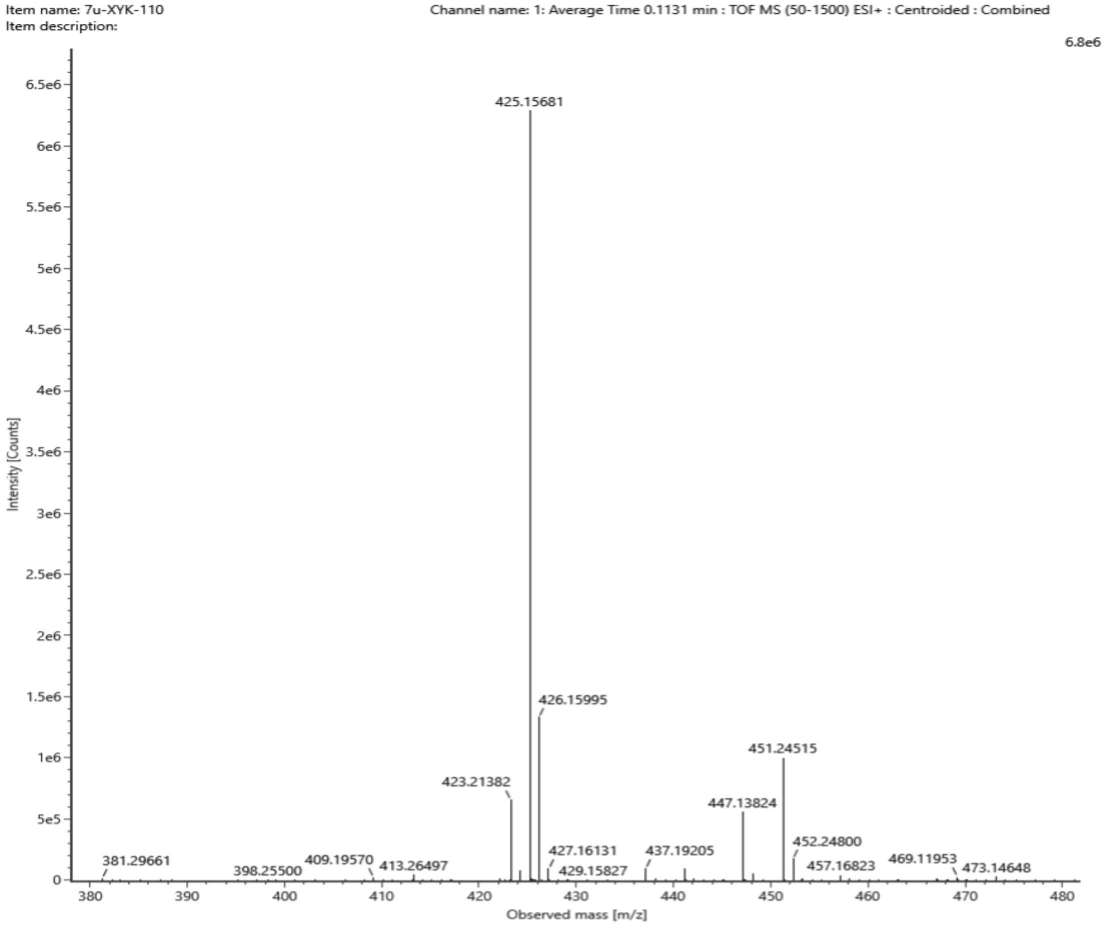

Figure S67. HRMS spectrum of compound **6v**

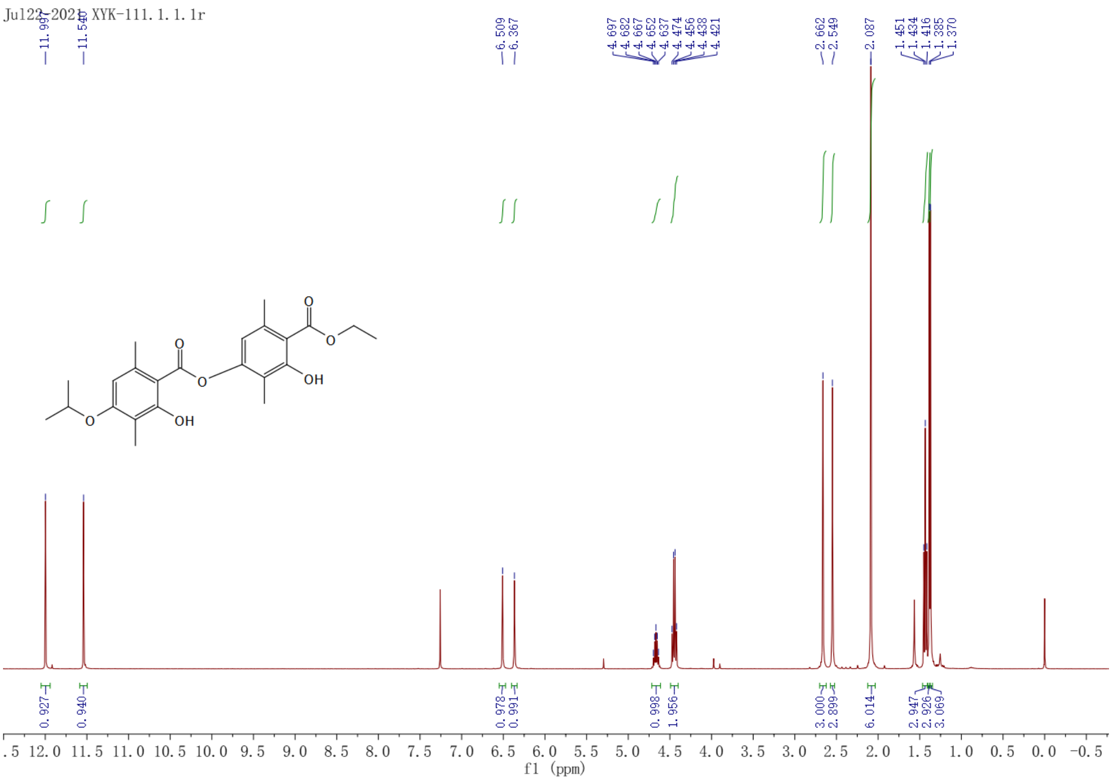

Figure S68. ^{1}H NMR spectrum of compound **6w**

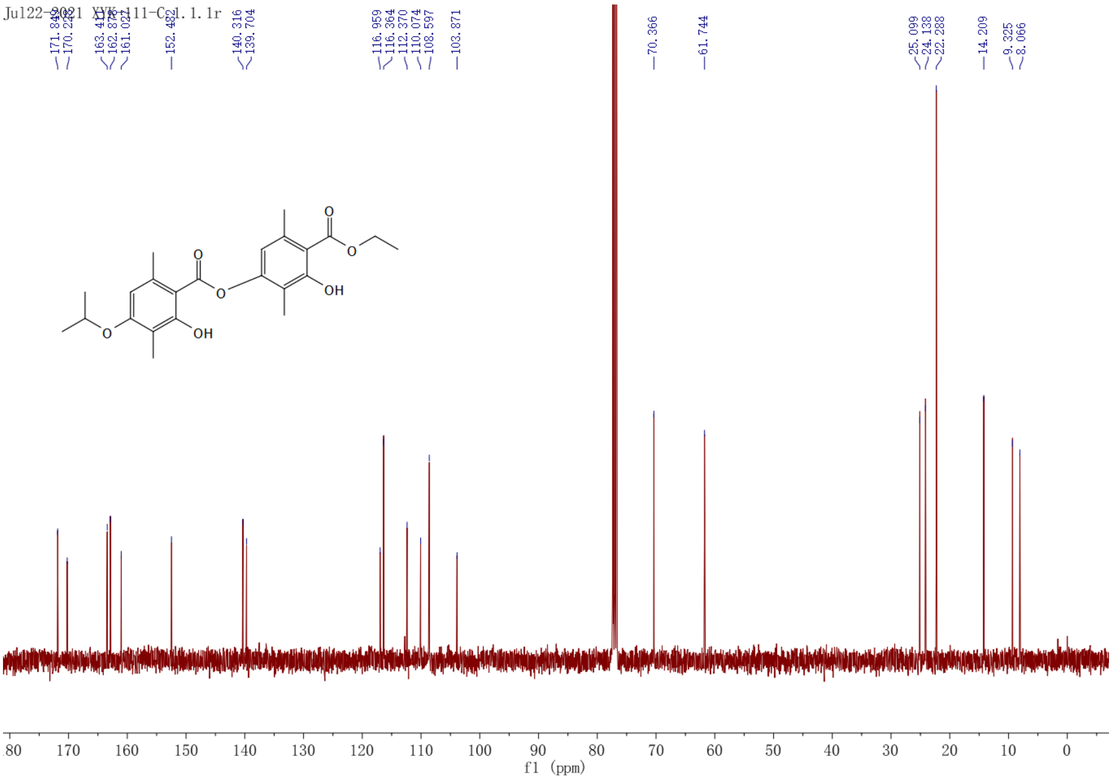

Figure S69. ^{13}C NMR spectrum of compound **6w**

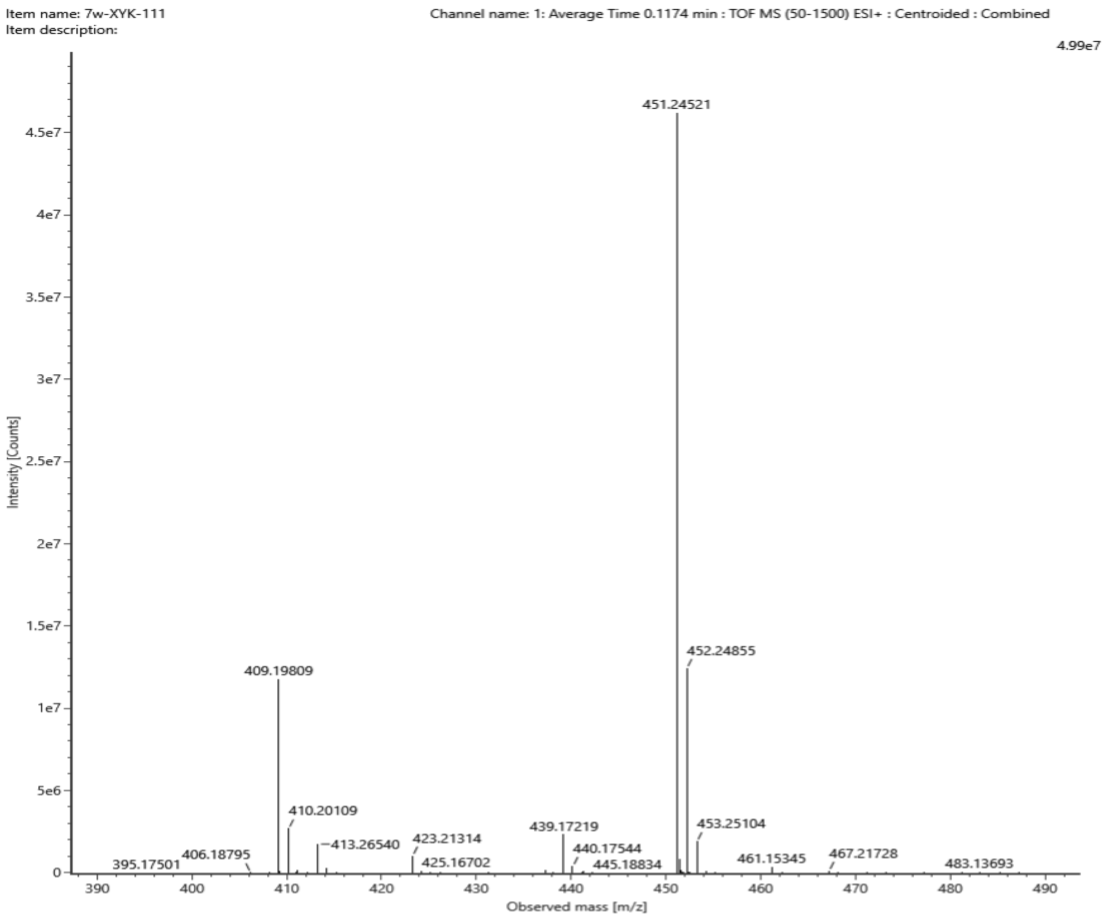

Figure S70. HRMS spectrum of compound **6w**

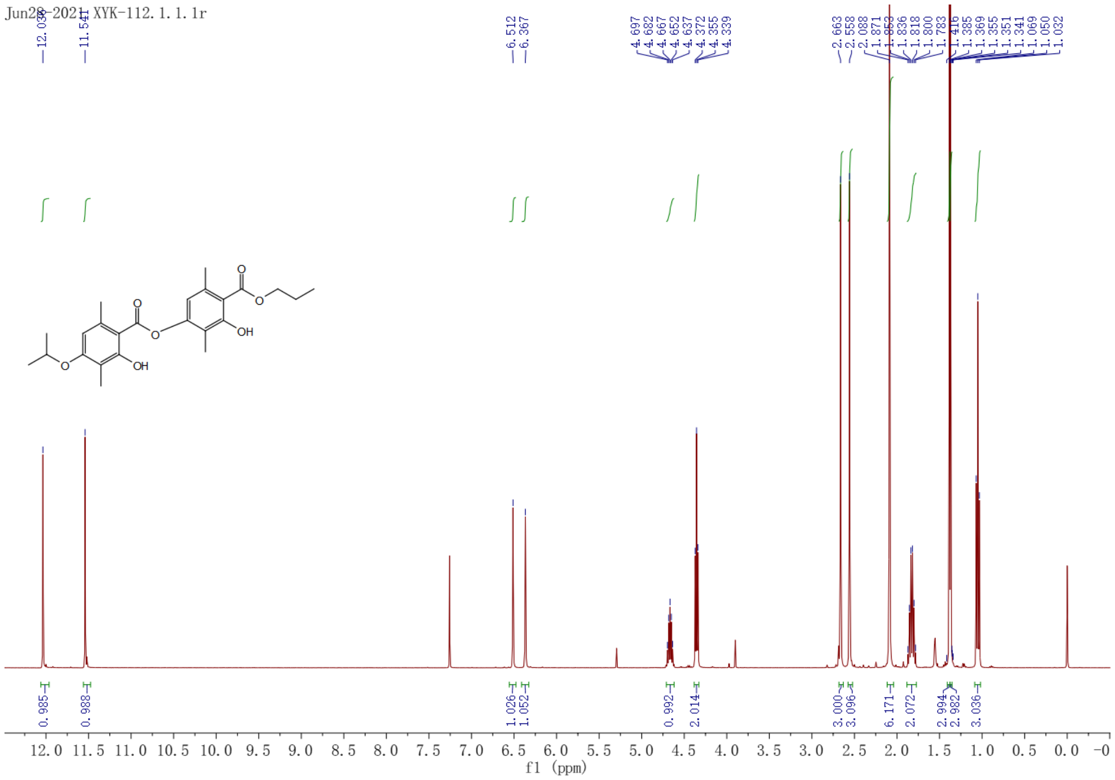

Figure S71. ^{1}H NMR spectrum of compound **6x**

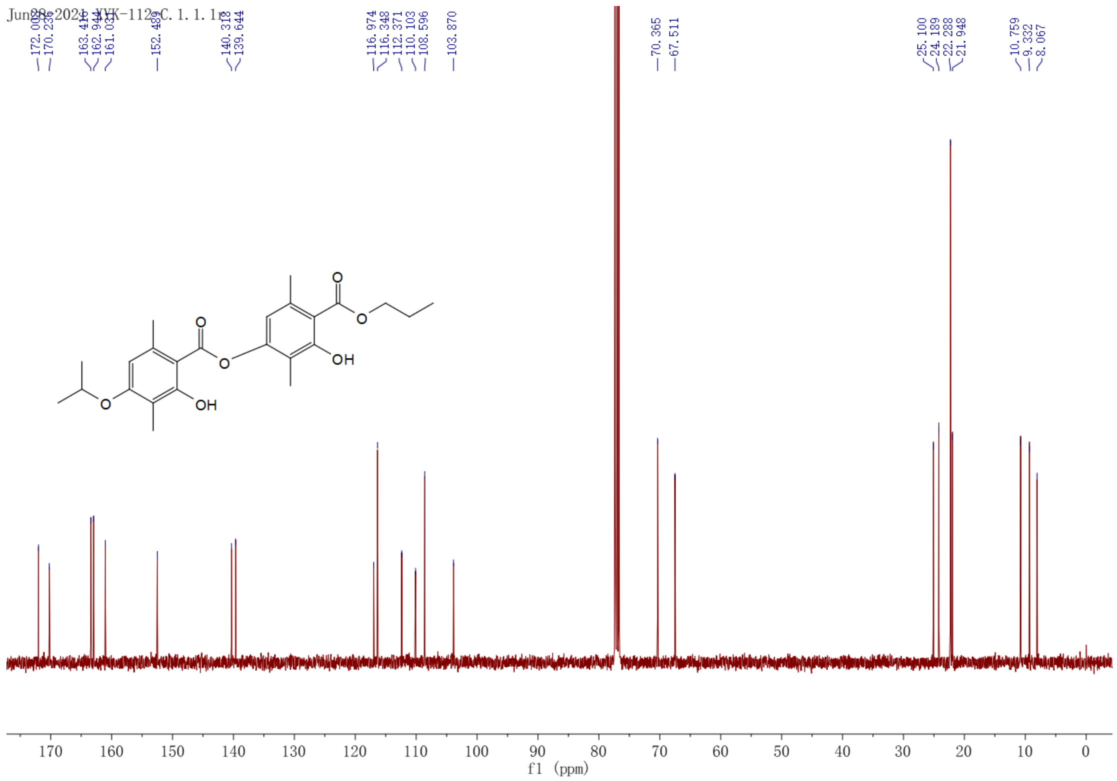

Figure S72. ^{13}C NMR spectrum of compound **6x**

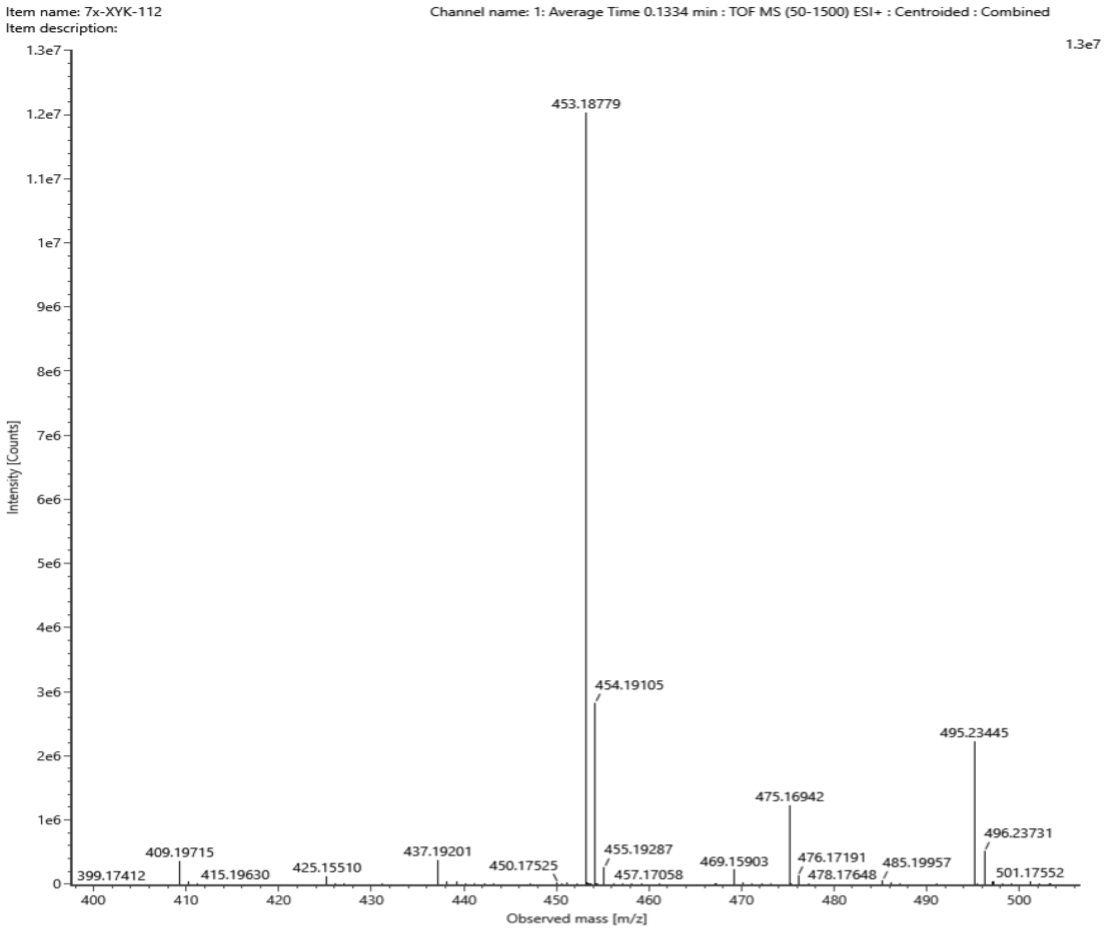

Figure S73. HRMS spectrum of compound **6x**

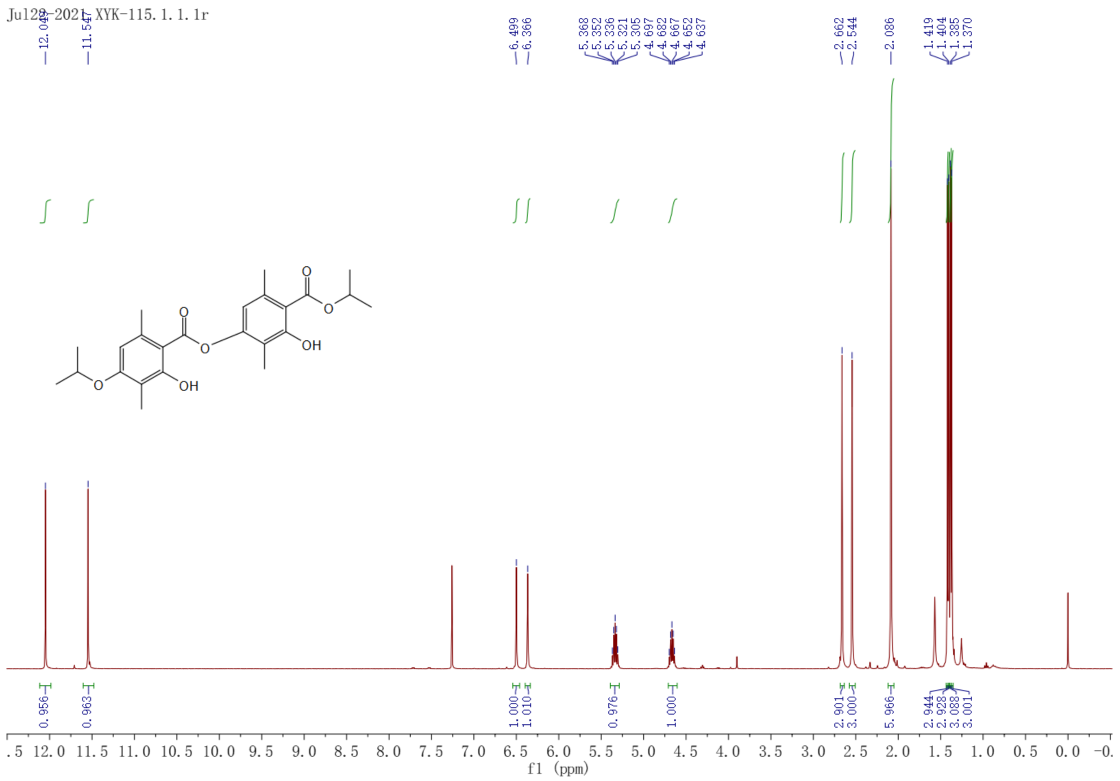

Figure S74. ^{1}H NMR spectrum of compound **6y**

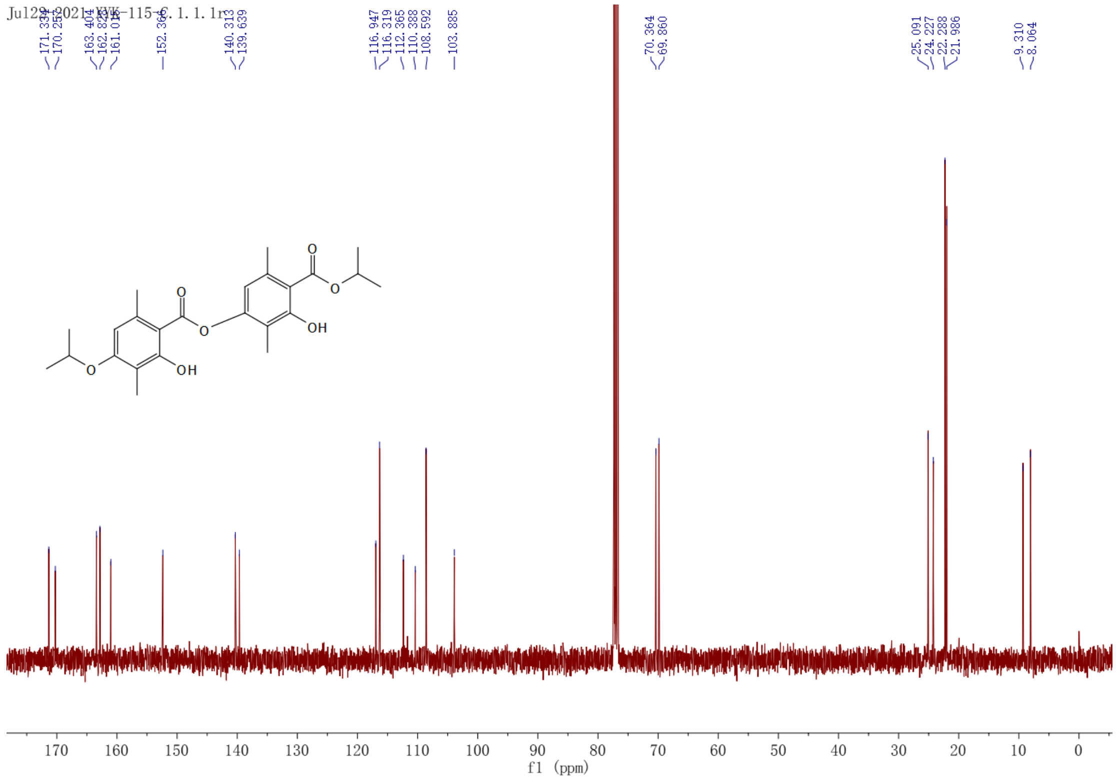

Figure S75. ^{13}C NMR spectrum of compound **6y**

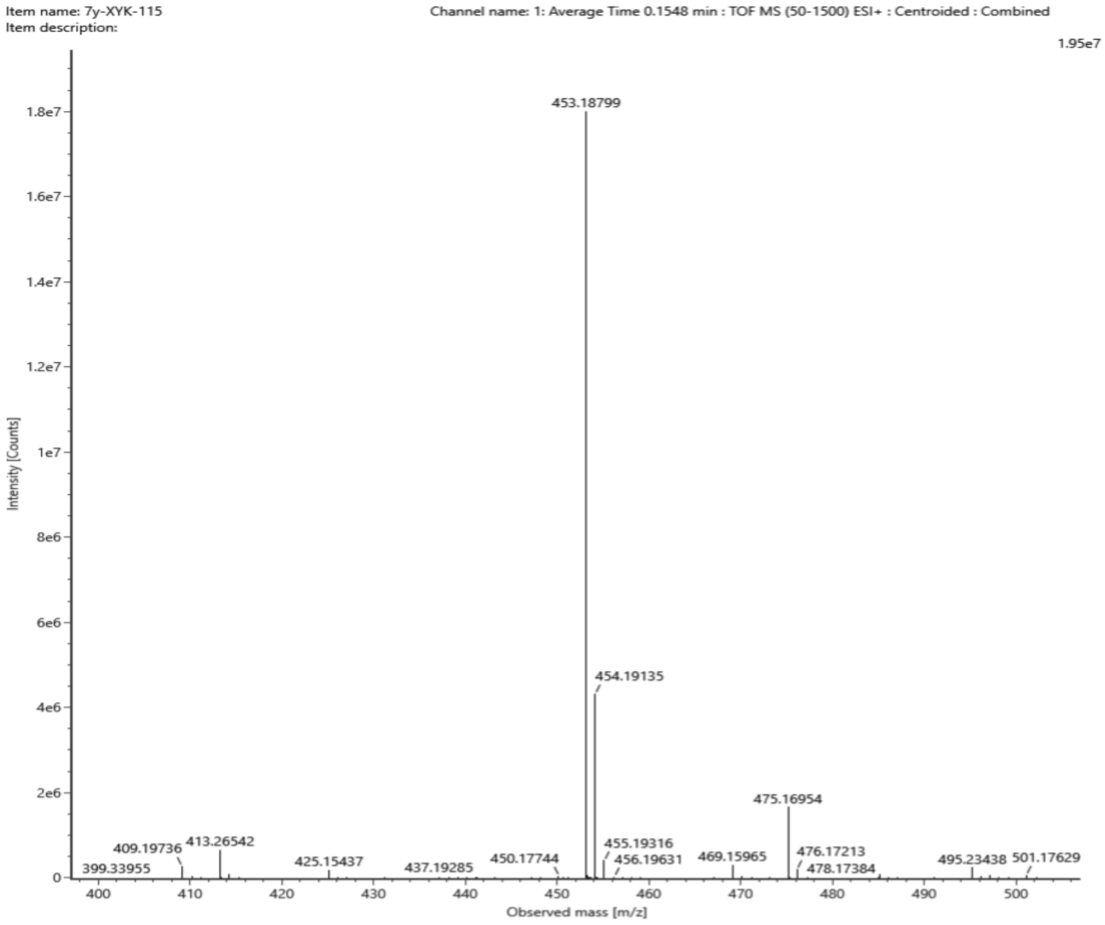

Figure S76. HRMS spectrum of compound **6y**

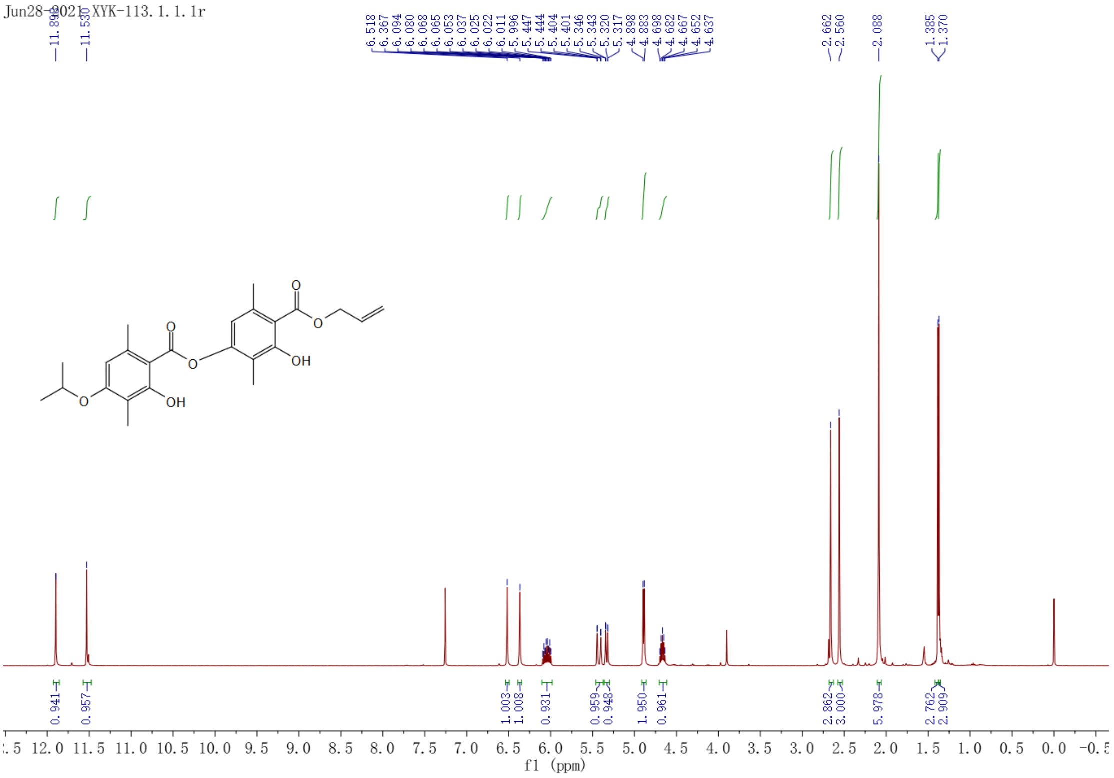

Figure S77. ^{1}H NMR spectrum of compound **6z**

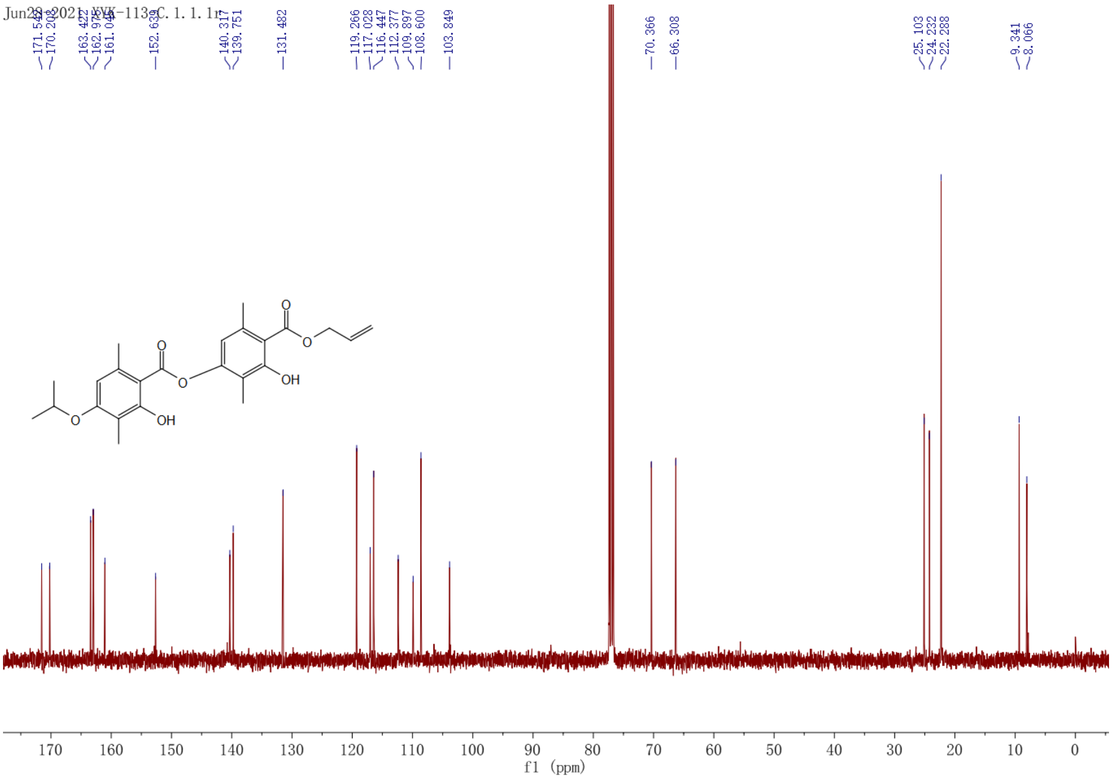

Figure S78. ^{13}C NMR spectrum of compound **6z**

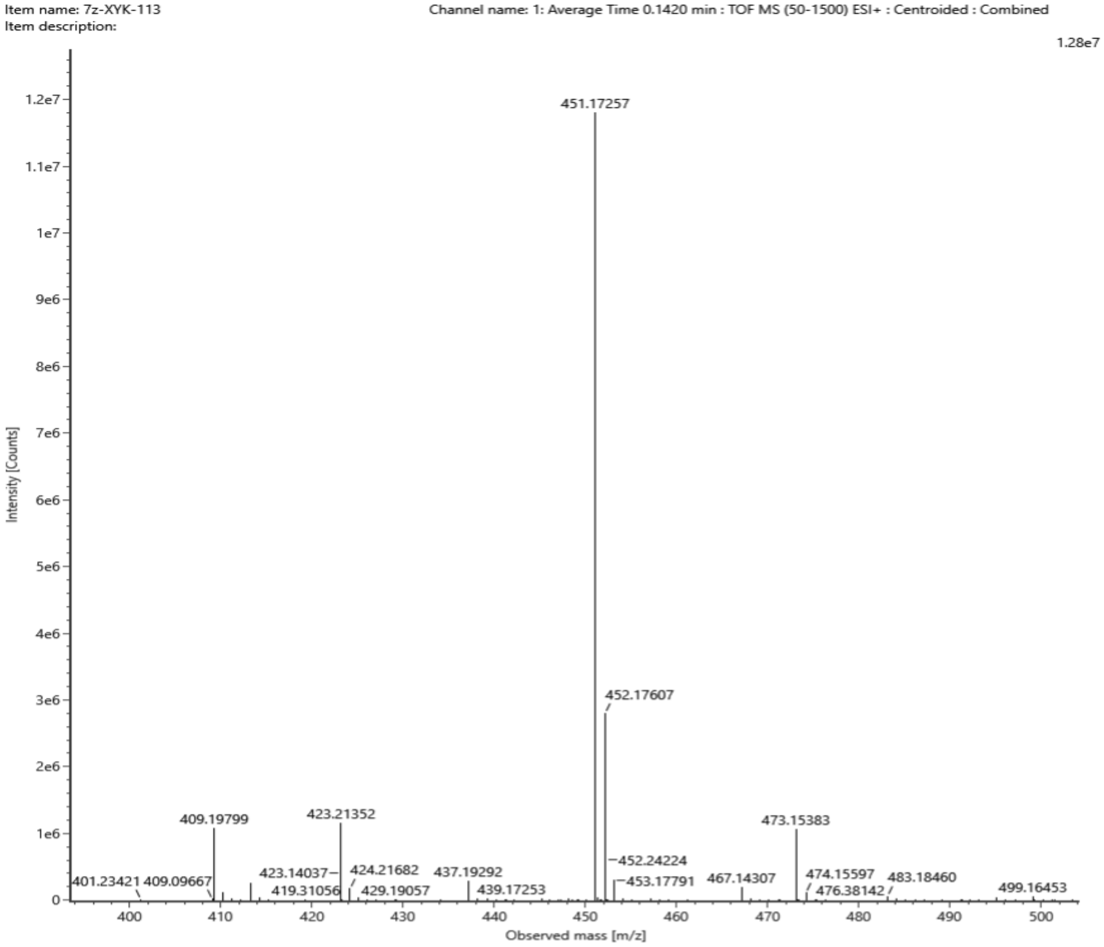

Figure S79. HRMS spectrum of compound **6z**

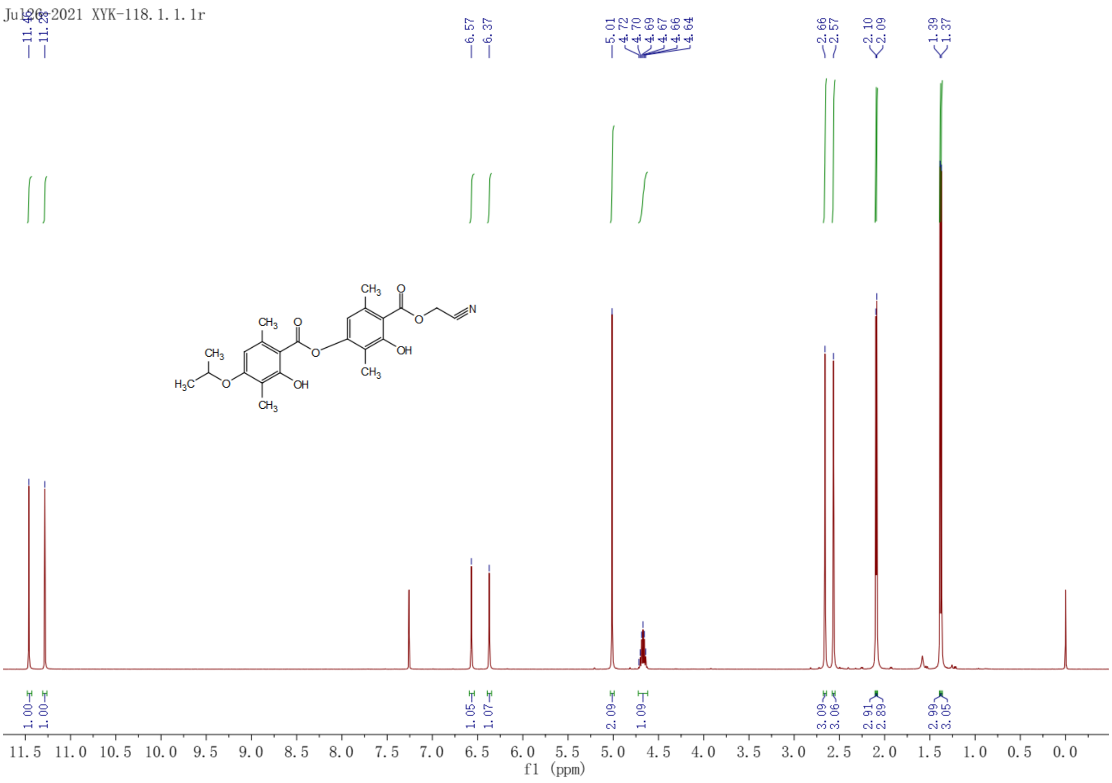

Figure S80. ^{1}H NMR spectrum of compound **6a’**

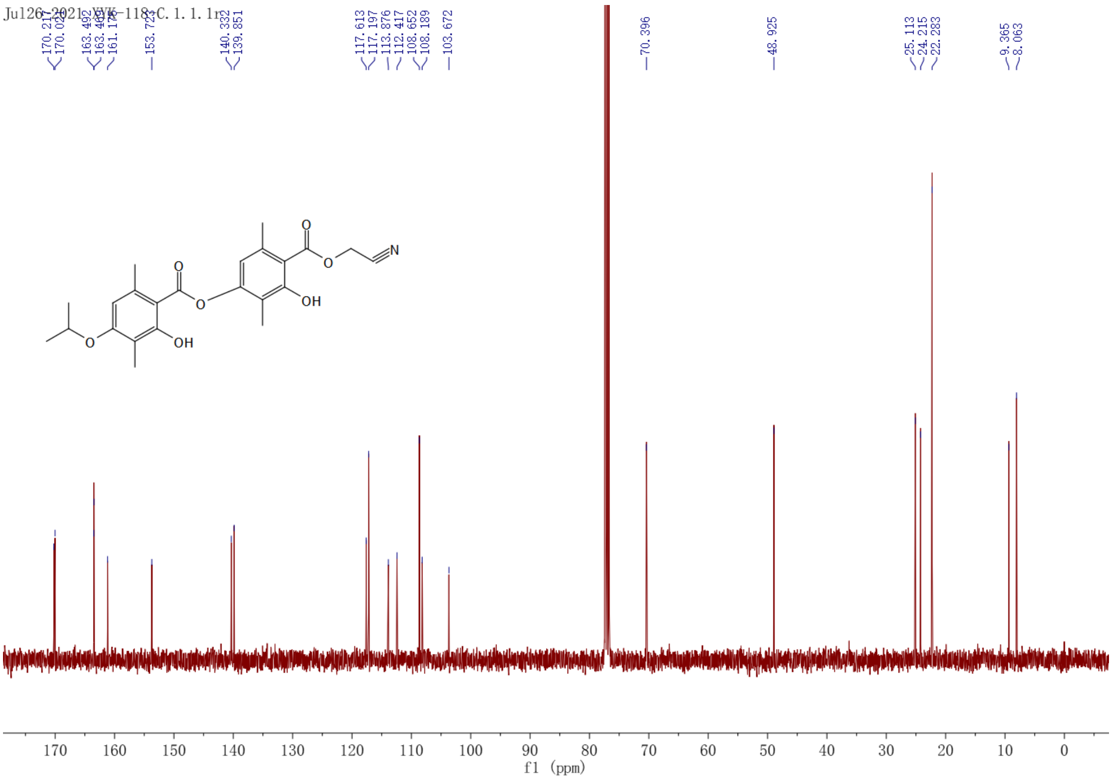

Figure S81. ^{13}C NMR spectrum of compound **6a’**

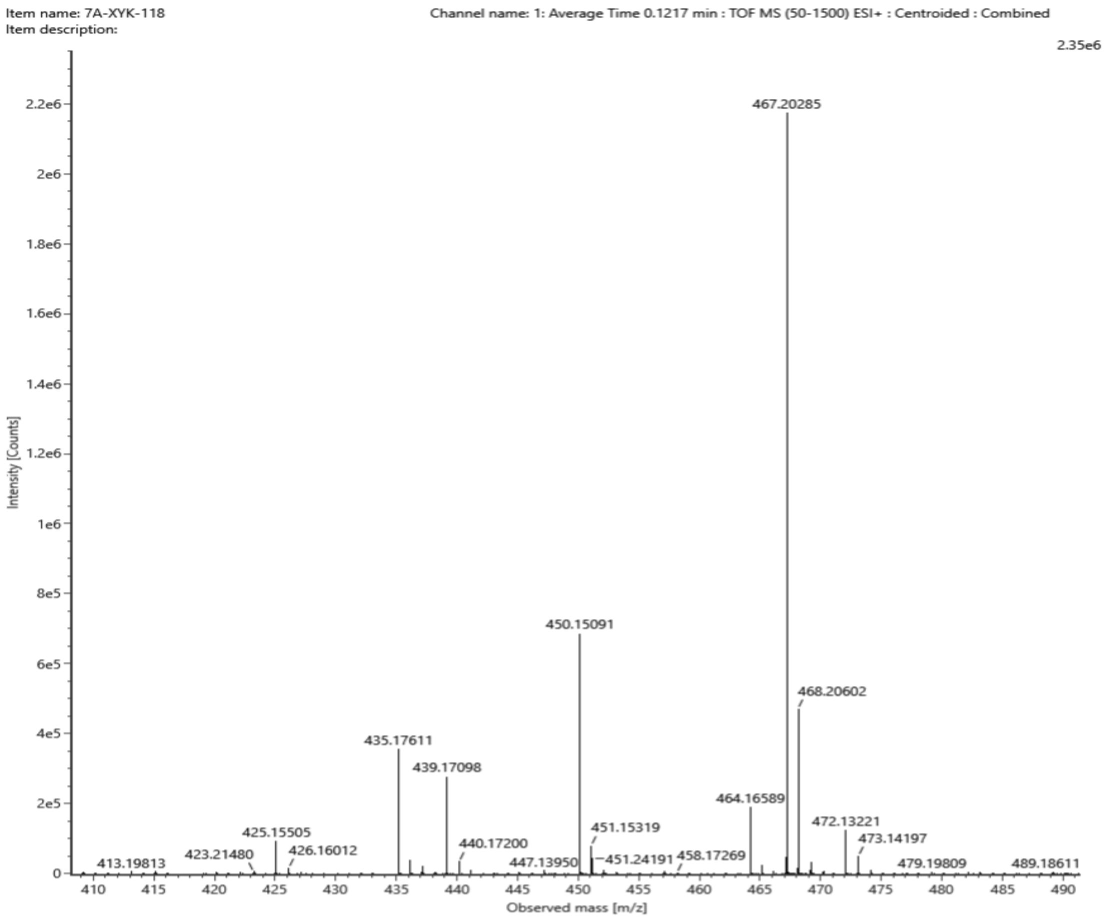

Figure S82. HRMS spectrum of compound **6a'**

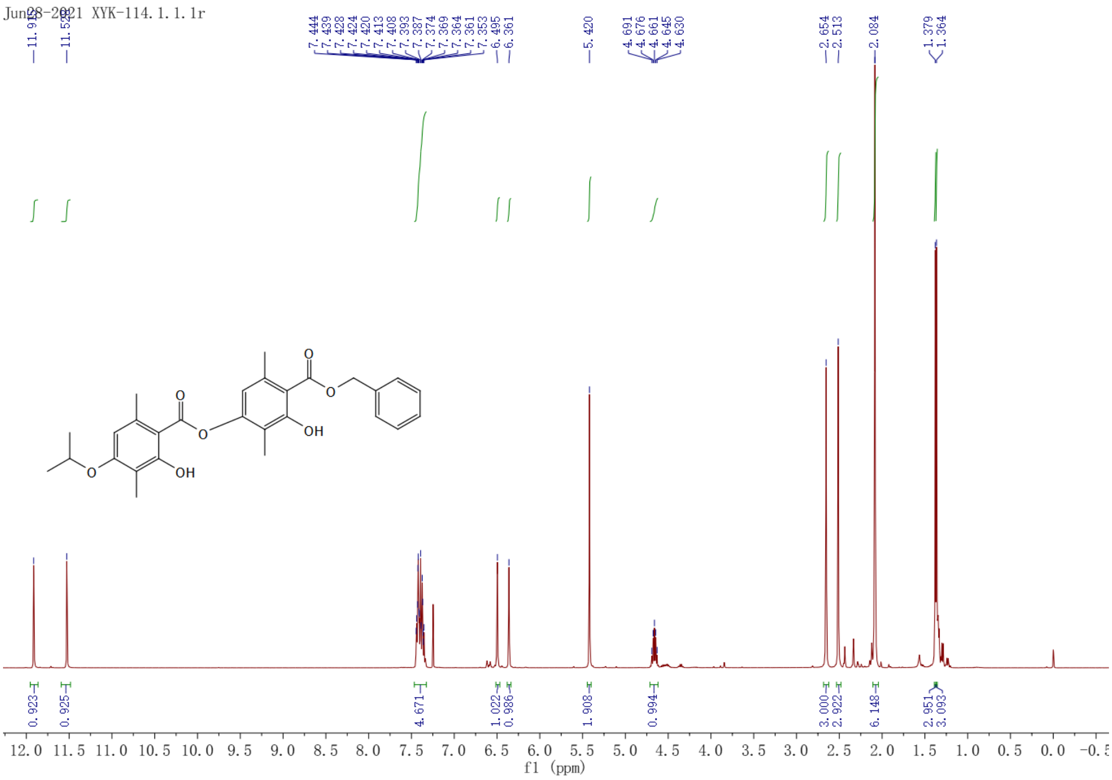

Figure S83. ¹H NMR spectrum of compound **6b'**

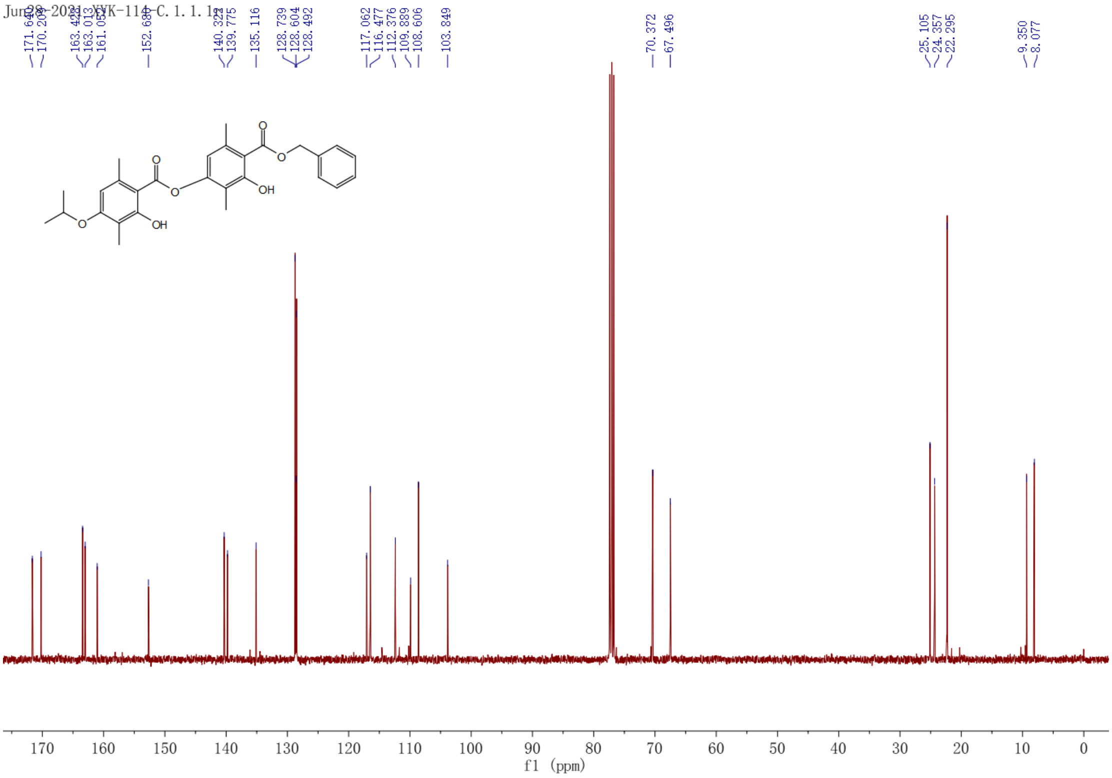

Figure S84. ^{13}C NMR spectrum of compound **6b’**

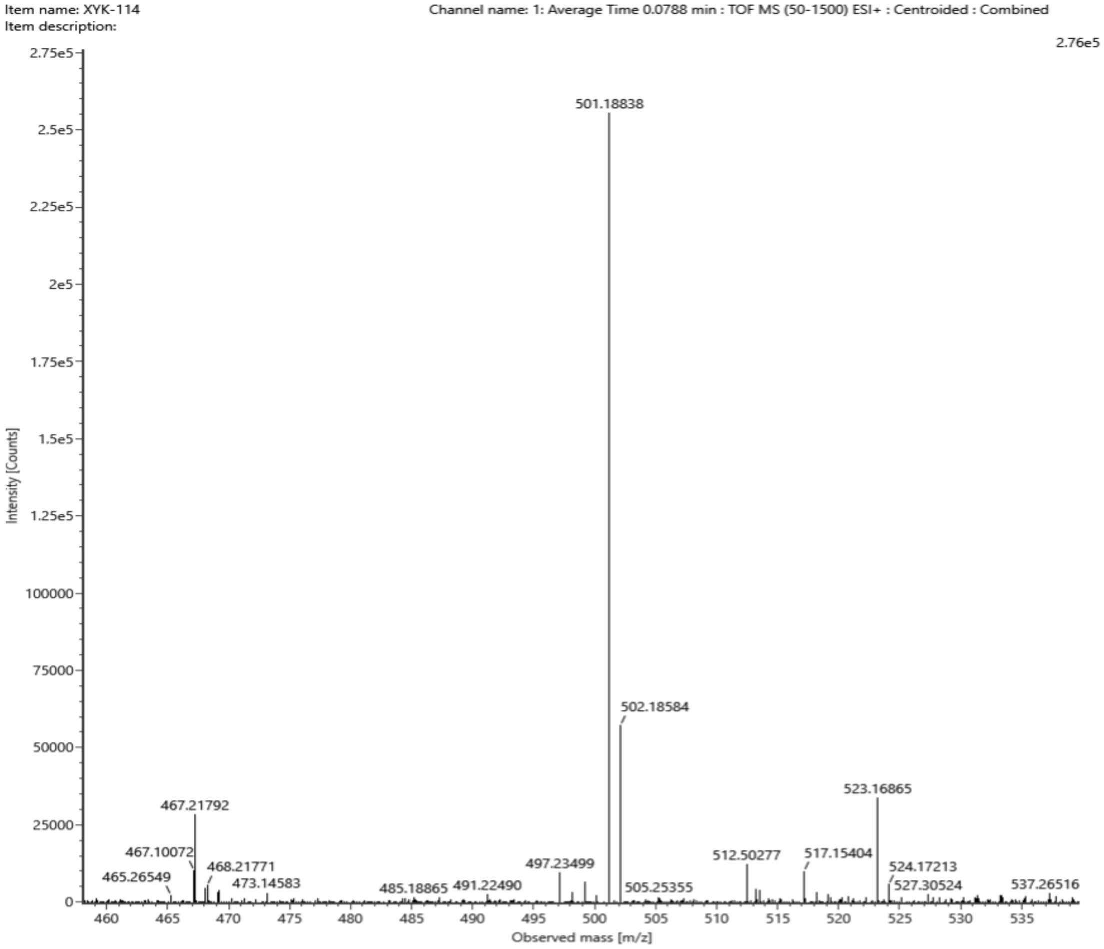

Figure S85. HRMS spectrum of compound **6b’**

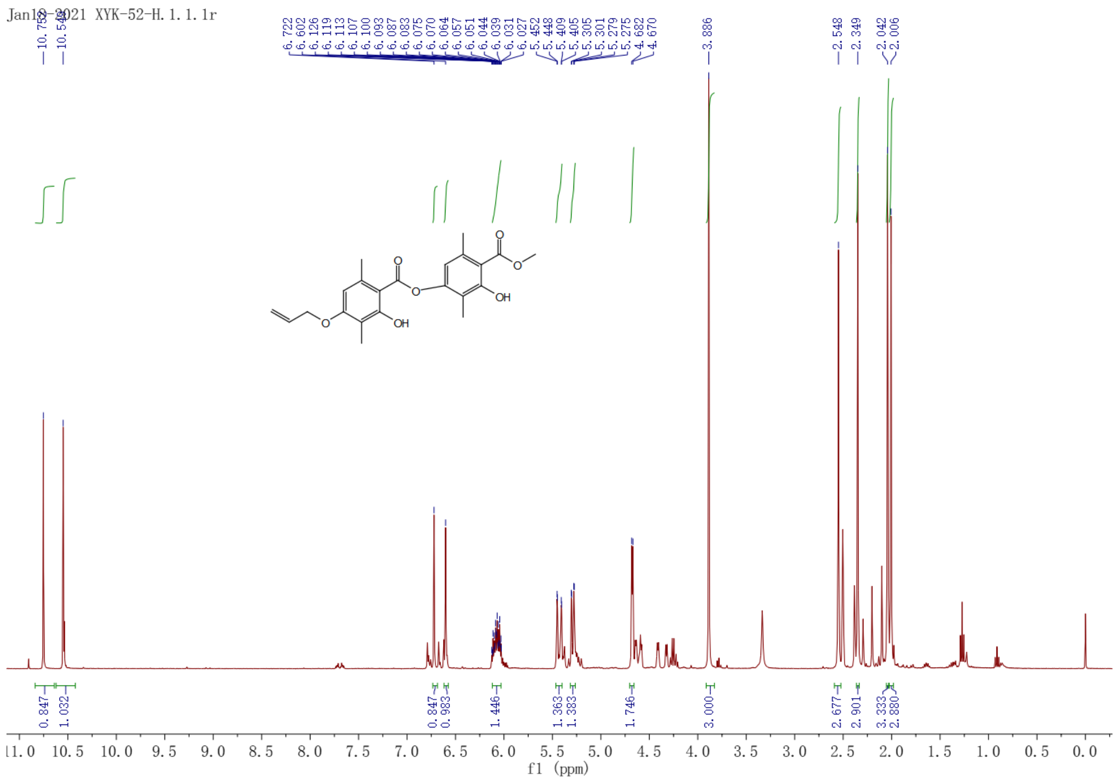

Figure S86. ^{1}H NMR spectrum of compound **6c’**

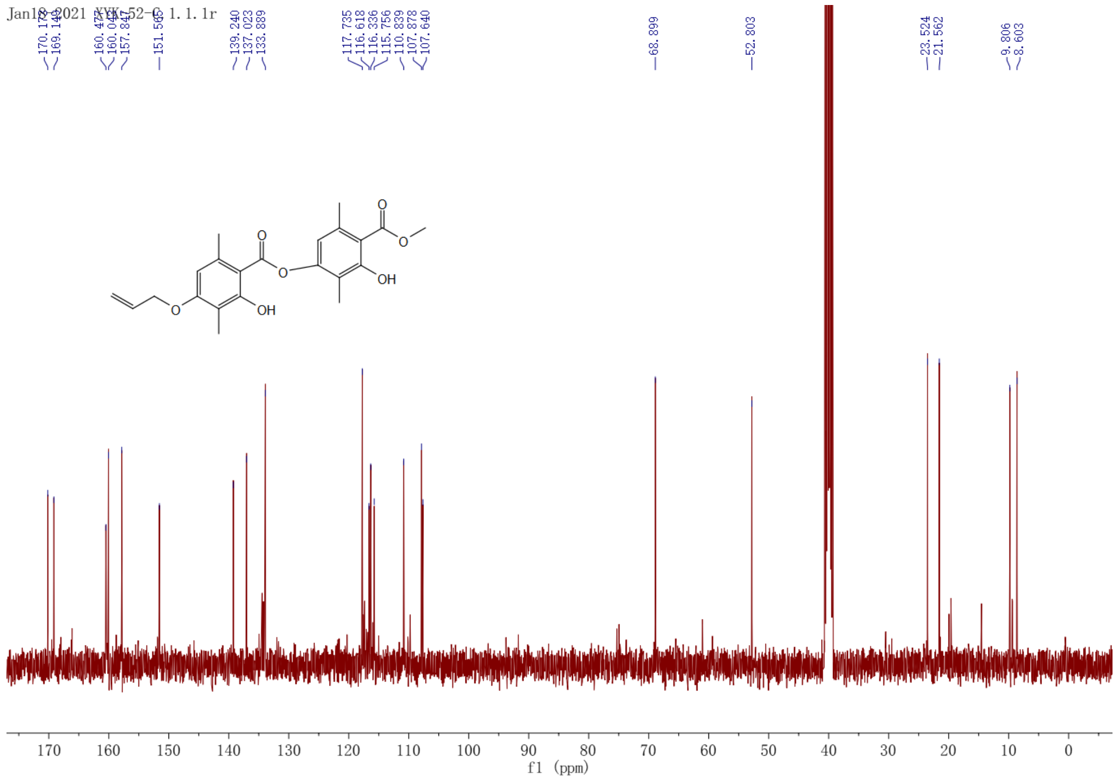

Figure S87. ^{13}C NMR spectrum of compound **6c’**

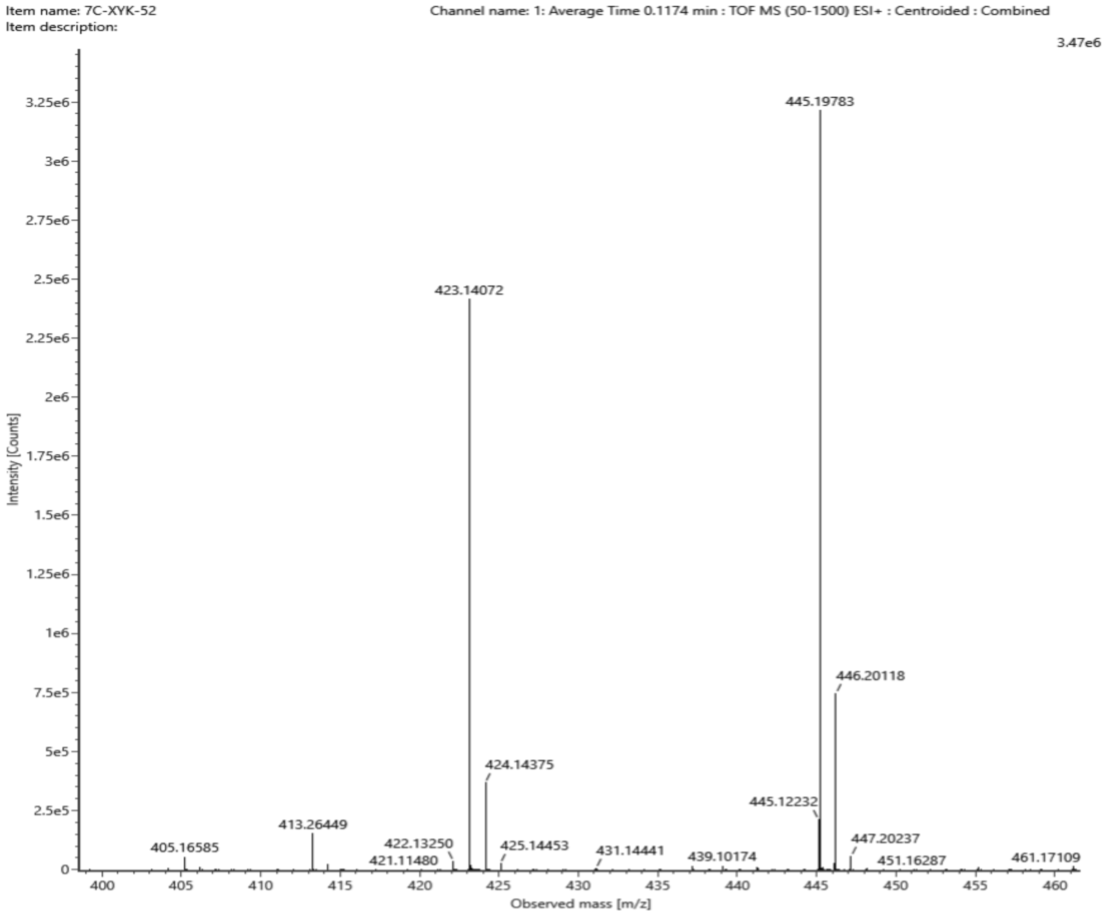

Figure S88. HRMS spectrum of compound **6c’**

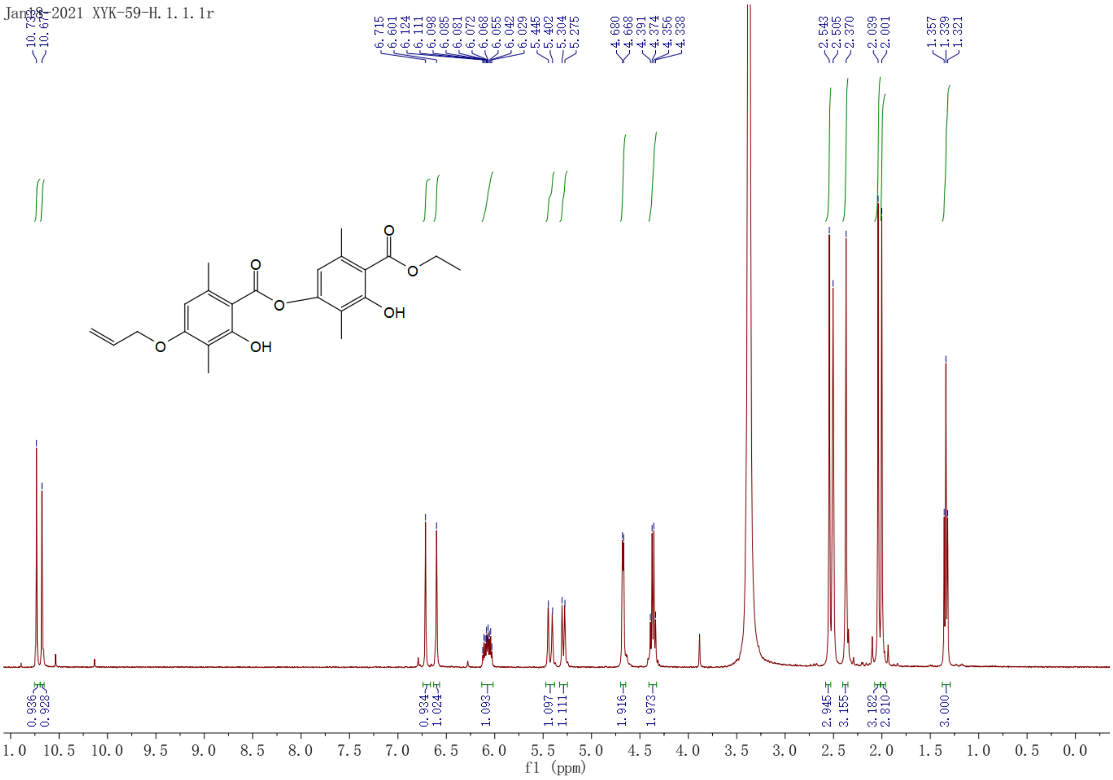

Figure S89. ^{1}H NMR spectrum of compound **6d’**

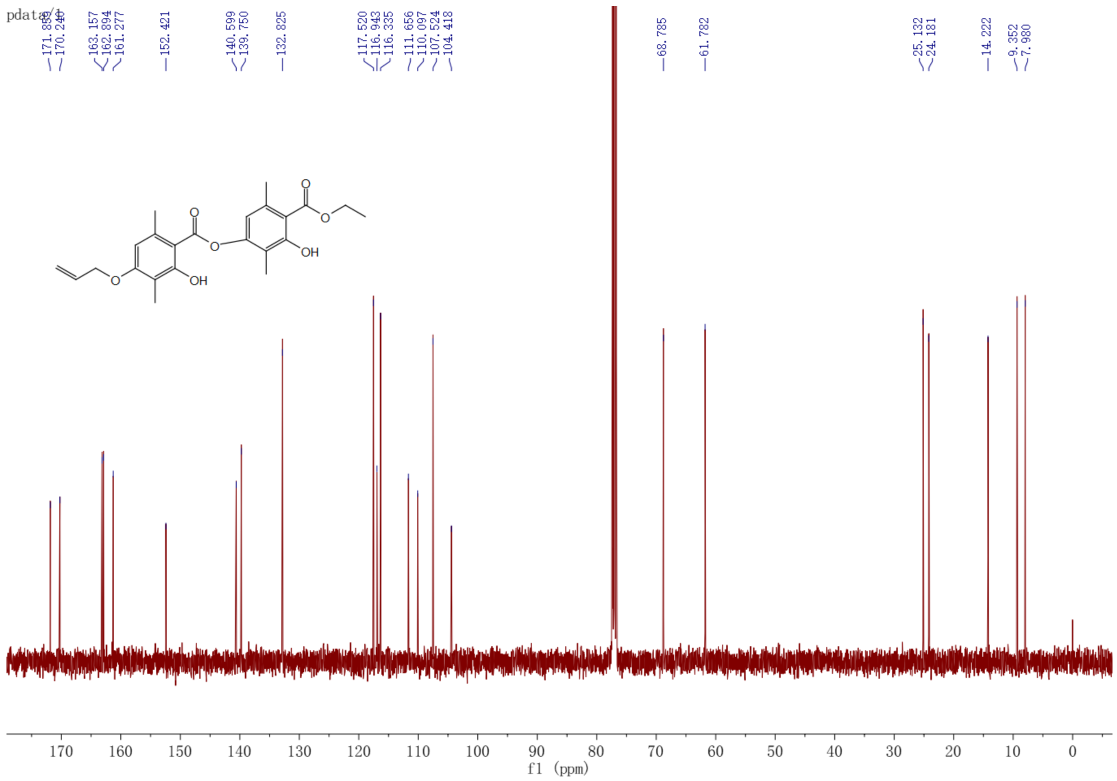

Figure S90. ^{13}C NMR spectrum of compound **6d'**

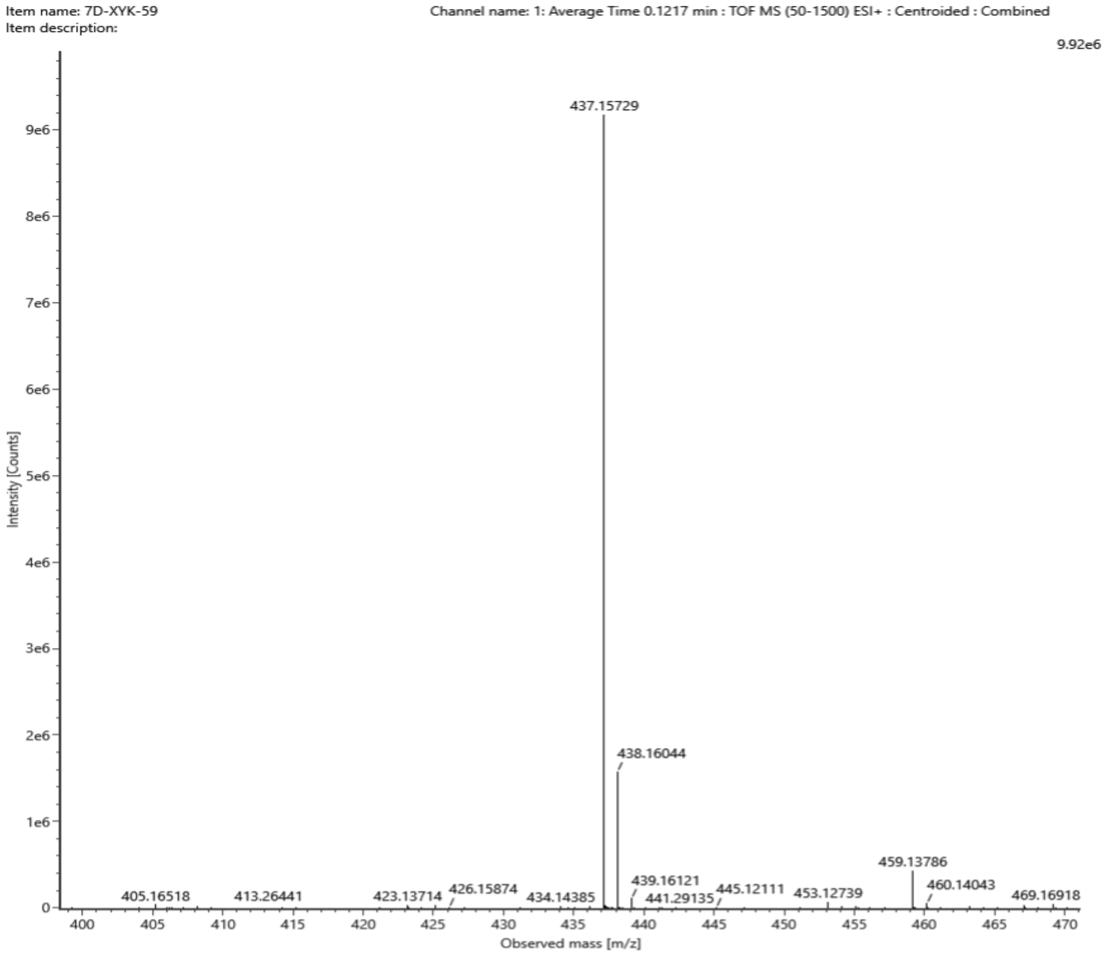

Figure S91. HRMS spectrum of compound **6d'**

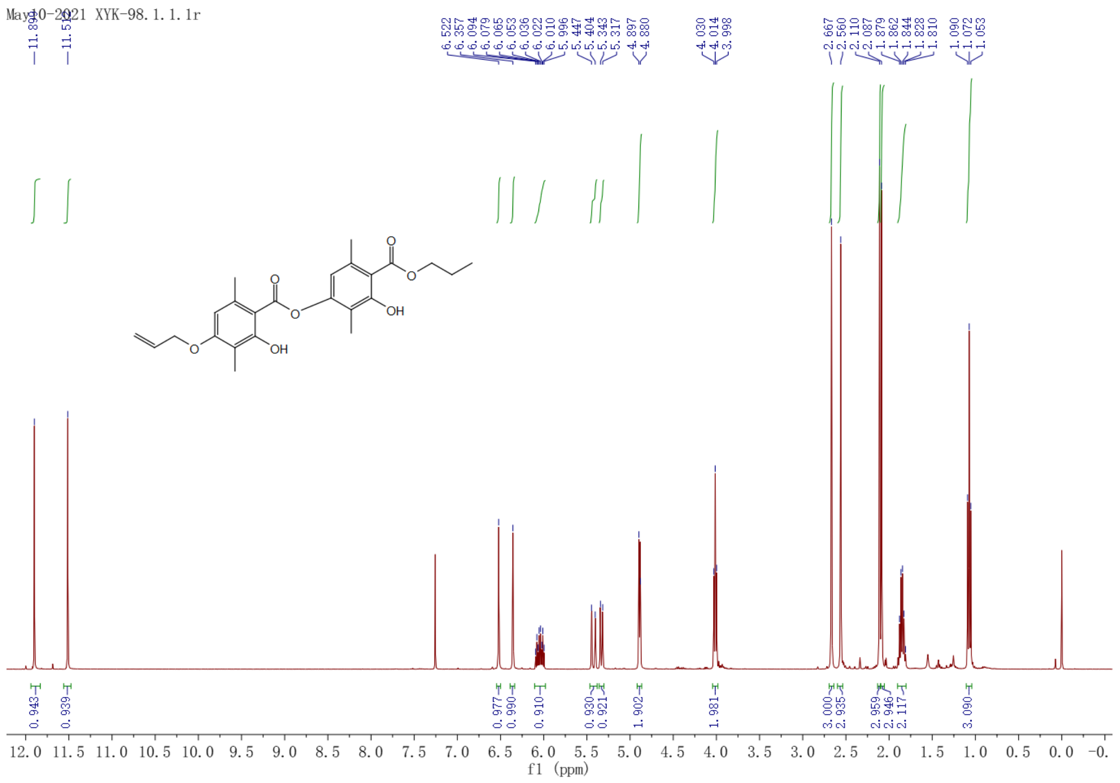

Figure S92. ^{1}H NMR spectrum of compound **6e'**

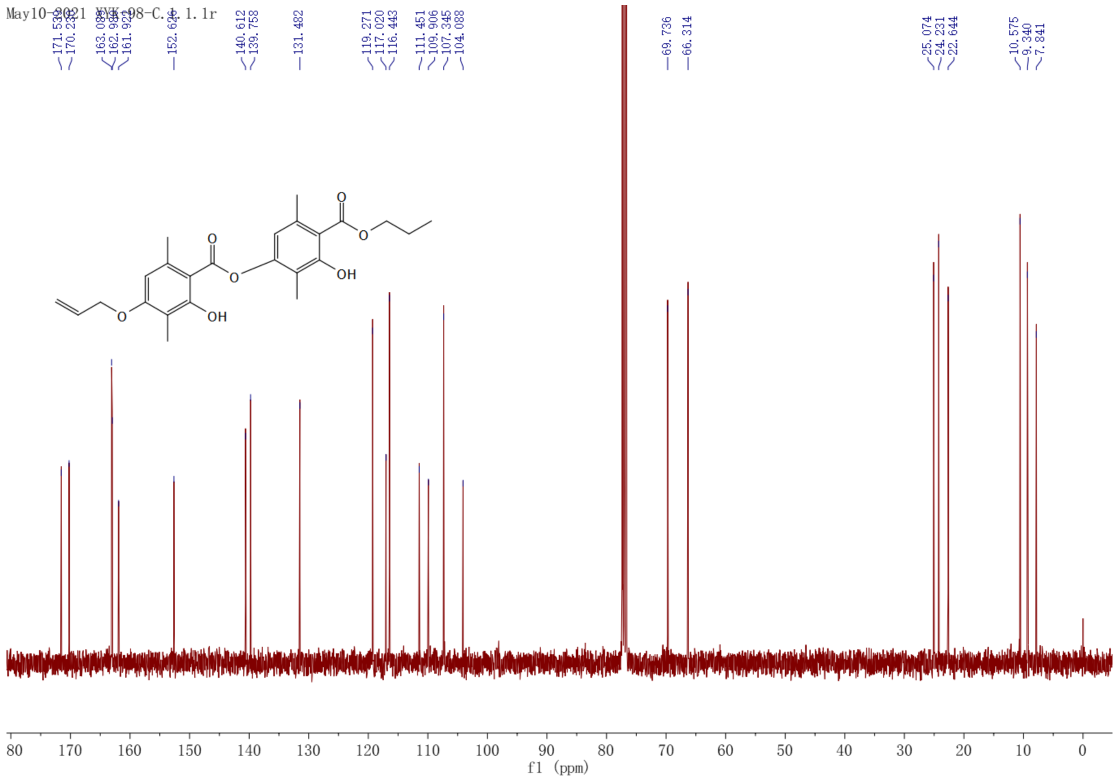

Figure S93. ^{13}C NMR spectrum of compound **6e'**

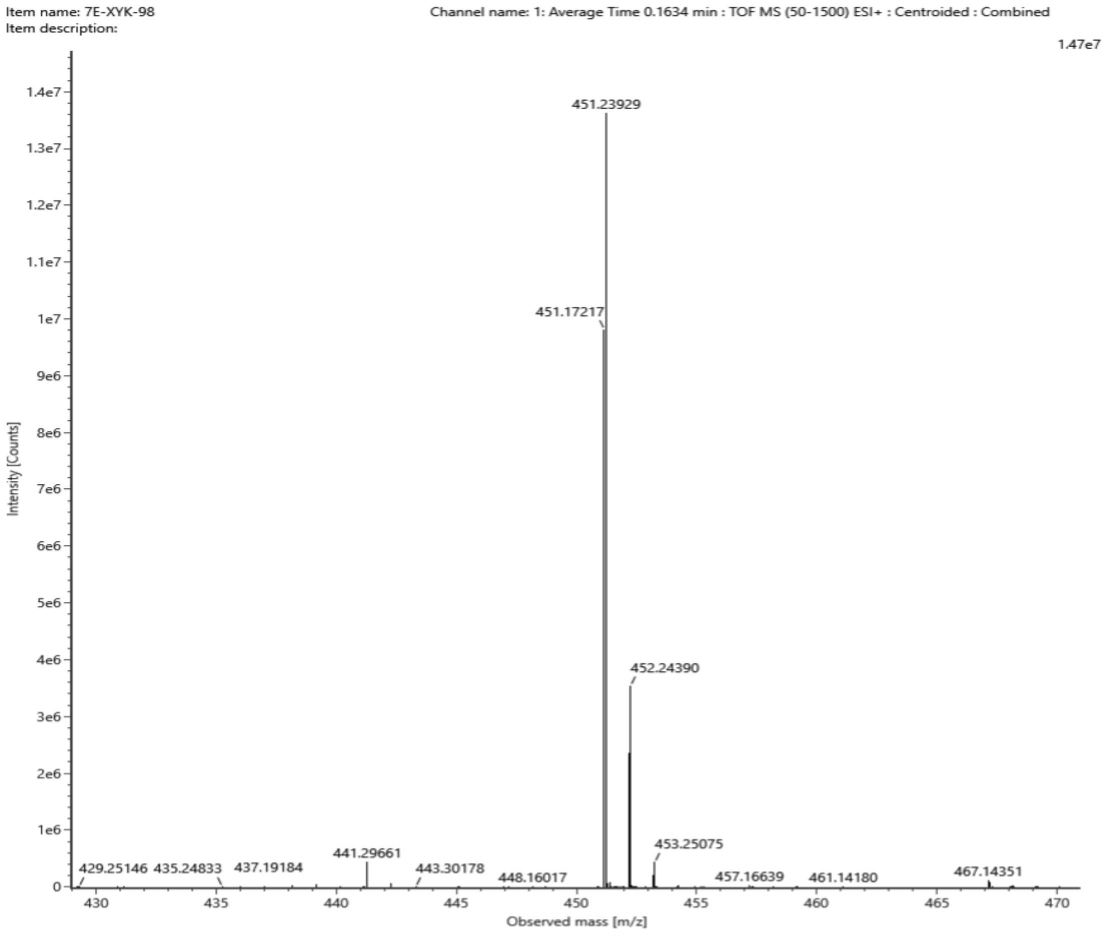

Figure S94. HRMS spectrum of compound **6e'**

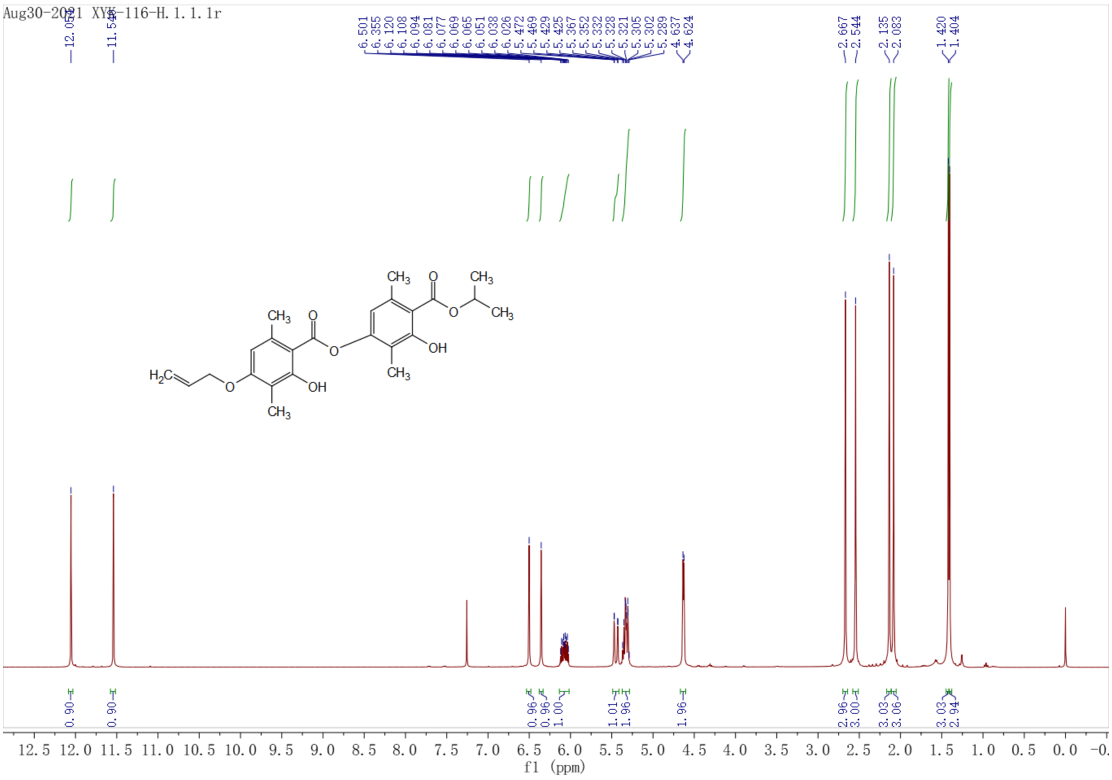

Figure S95. ^{1}H NMR spectrum of compound **6f'**

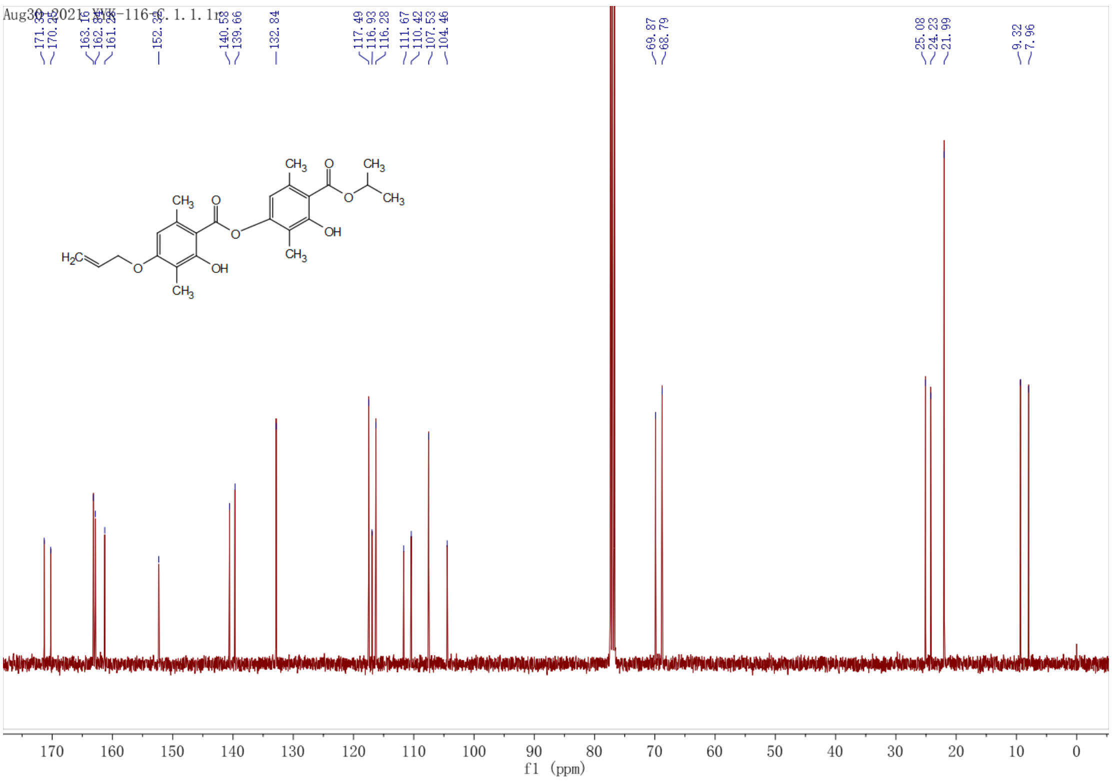

Figure S96. ^{13}C NMR spectrum of compound **6f'**

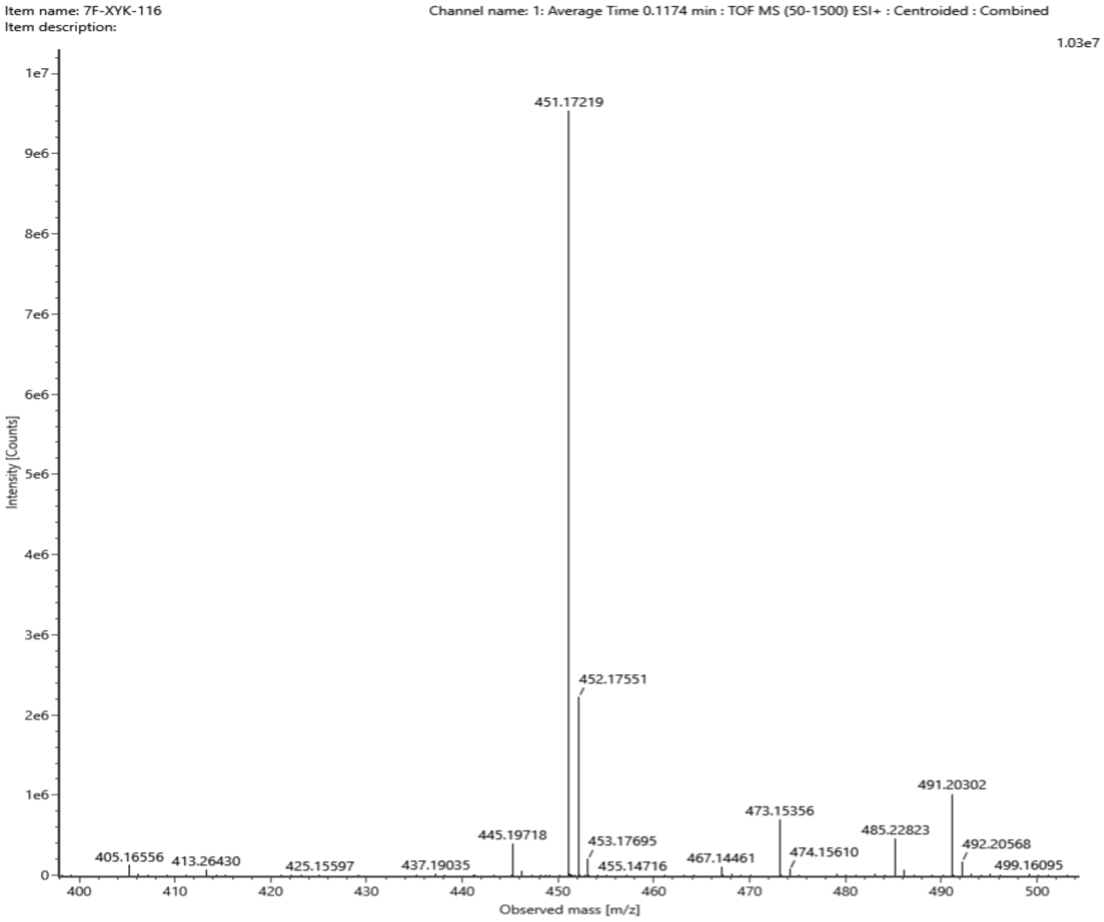

Figure S97. HRMS spectrum of compound **6f'**

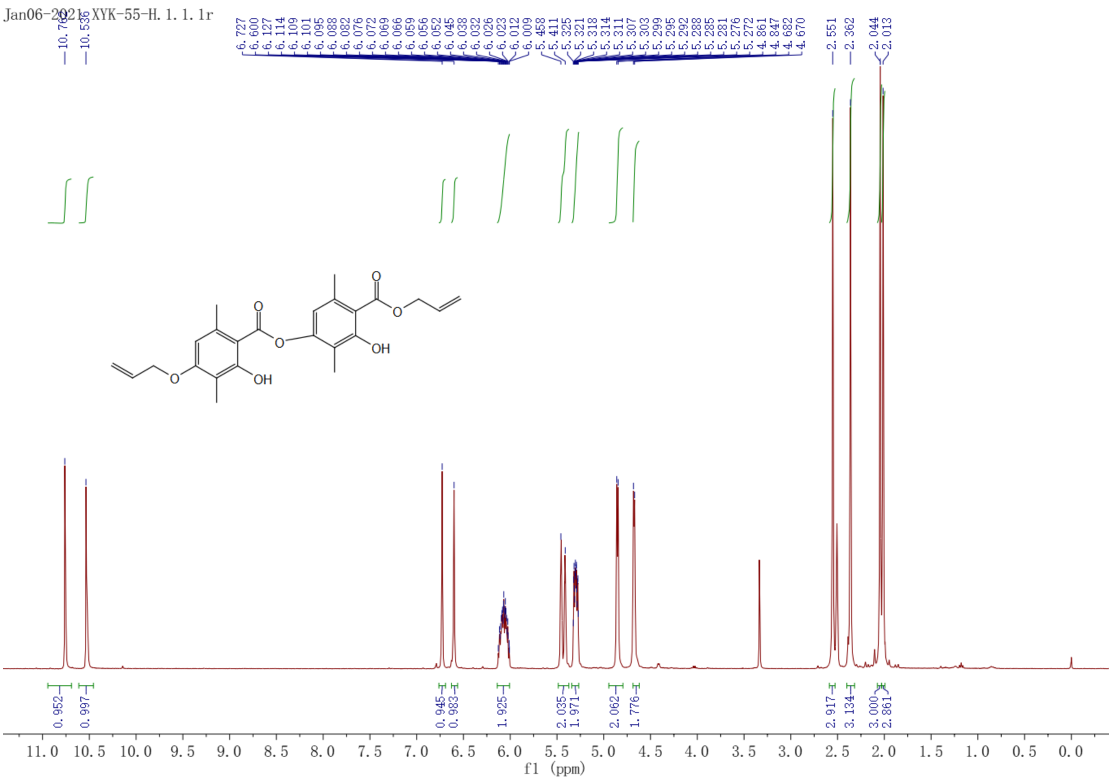

Figure S98. ^{1}H NMR spectrum of compound **6g’**

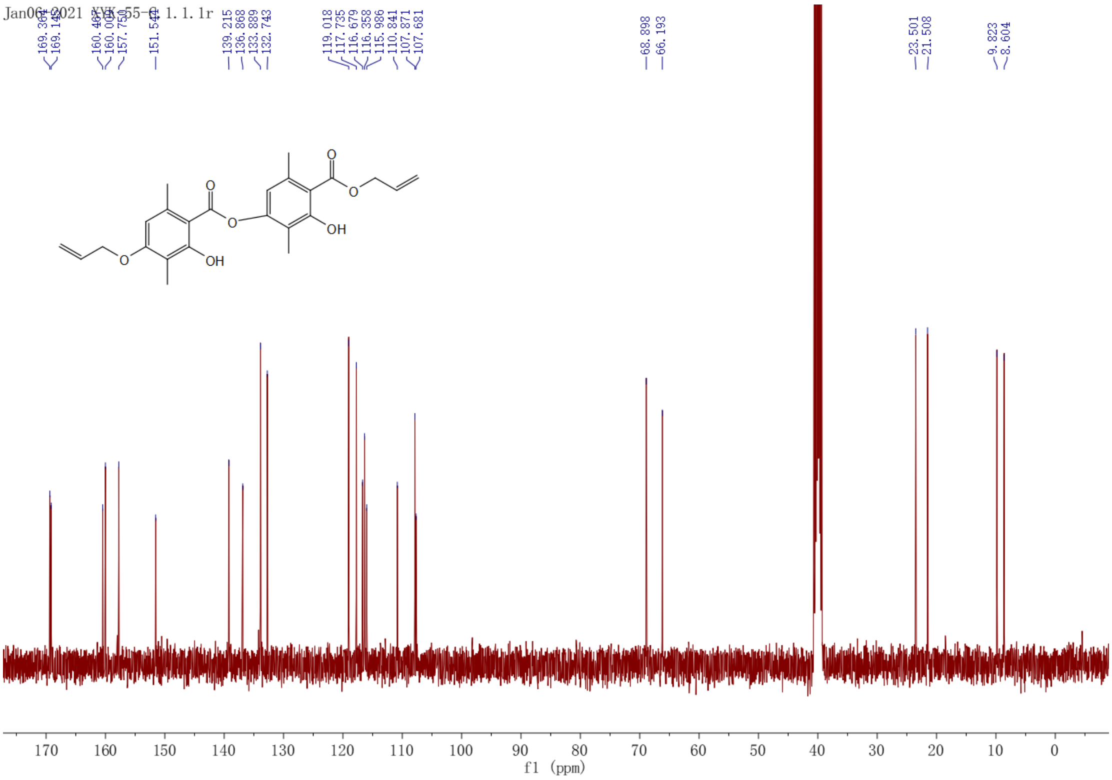

Figure S99. ^{13}C NMR spectrum of compound **6g’**

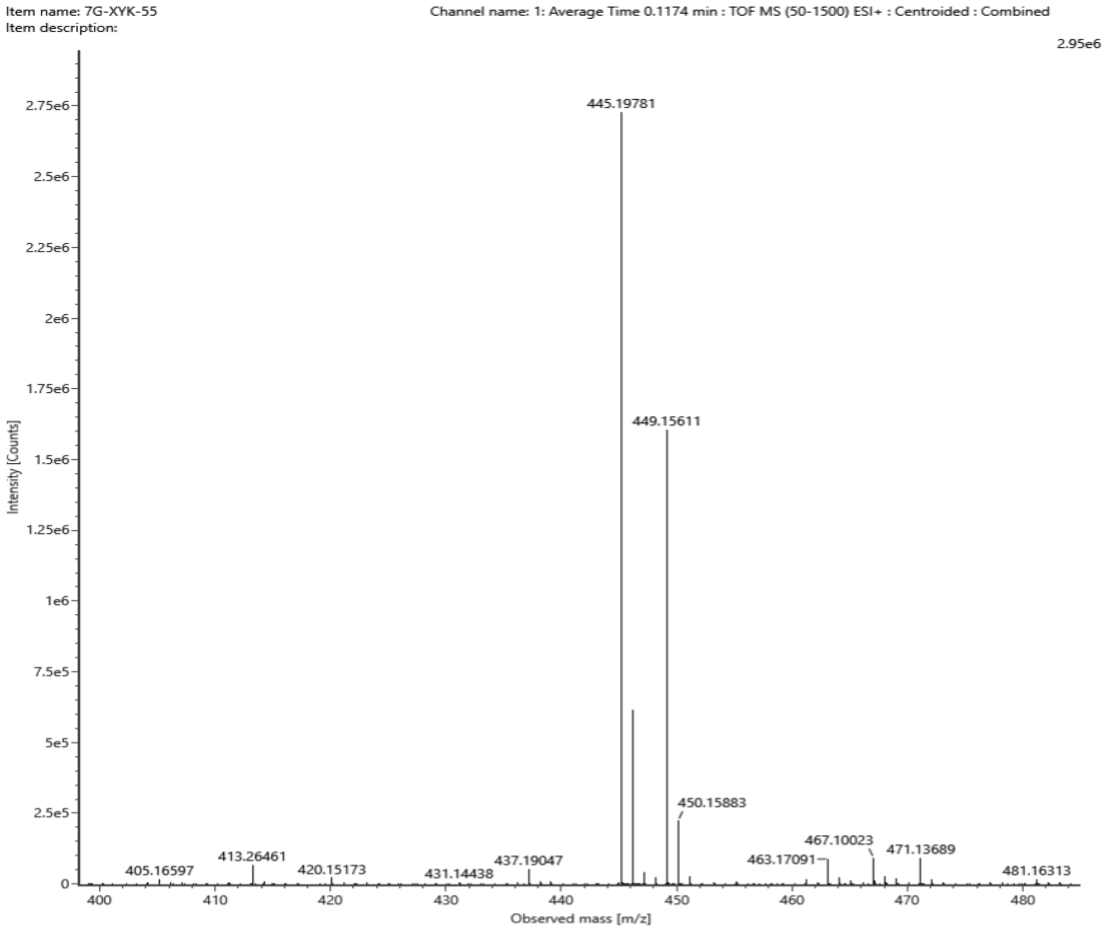

Figure S100. HRMS spectrum of compound **6g’**

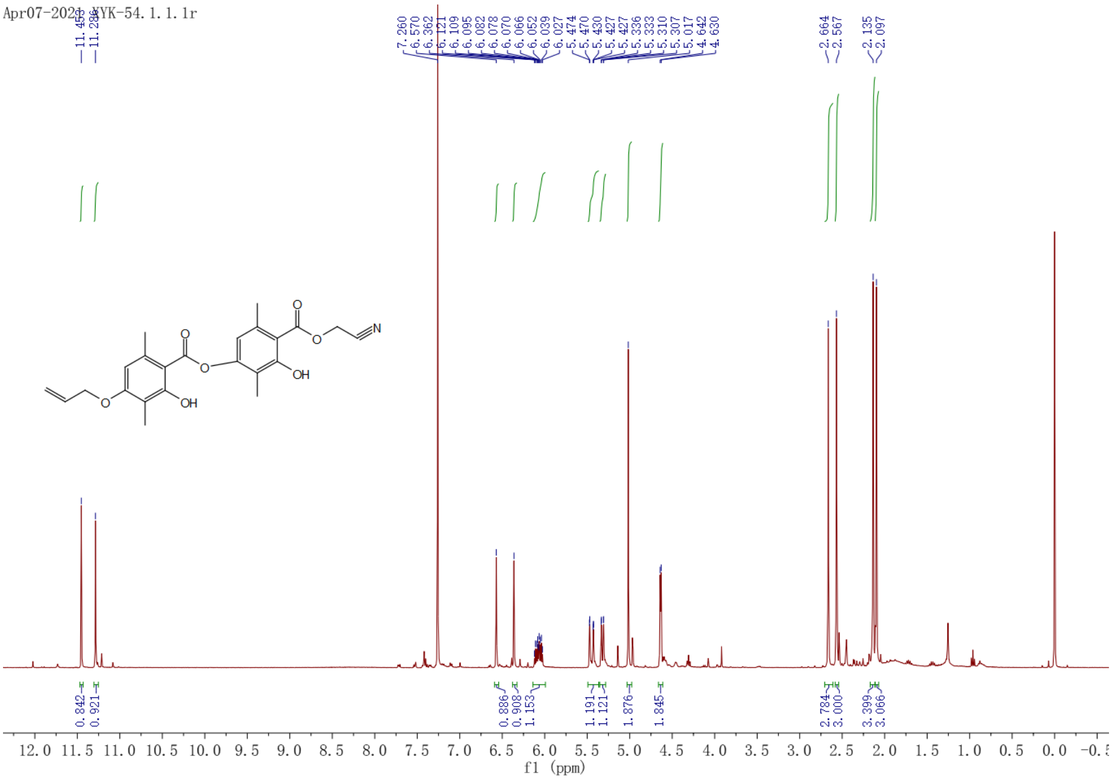

Figure S101. ^{1}H NMR spectrum of compound **6h’**

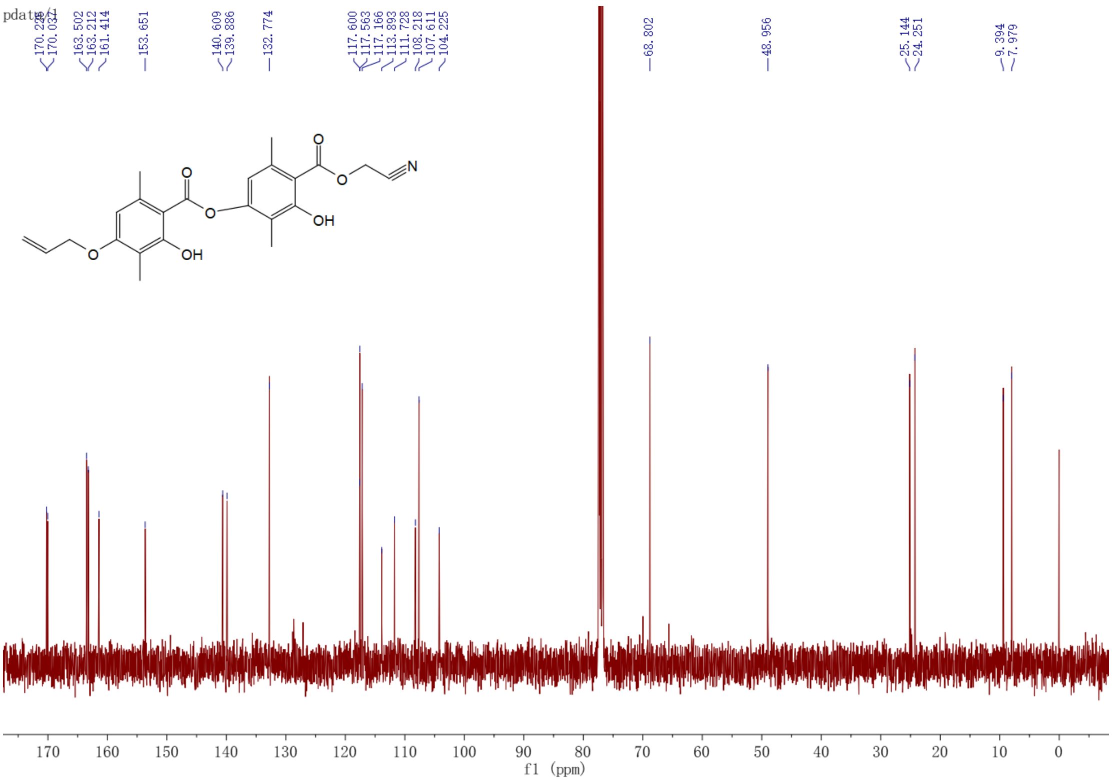

Figure S102. ^{13}C NMR spectrum of compound **6h'**

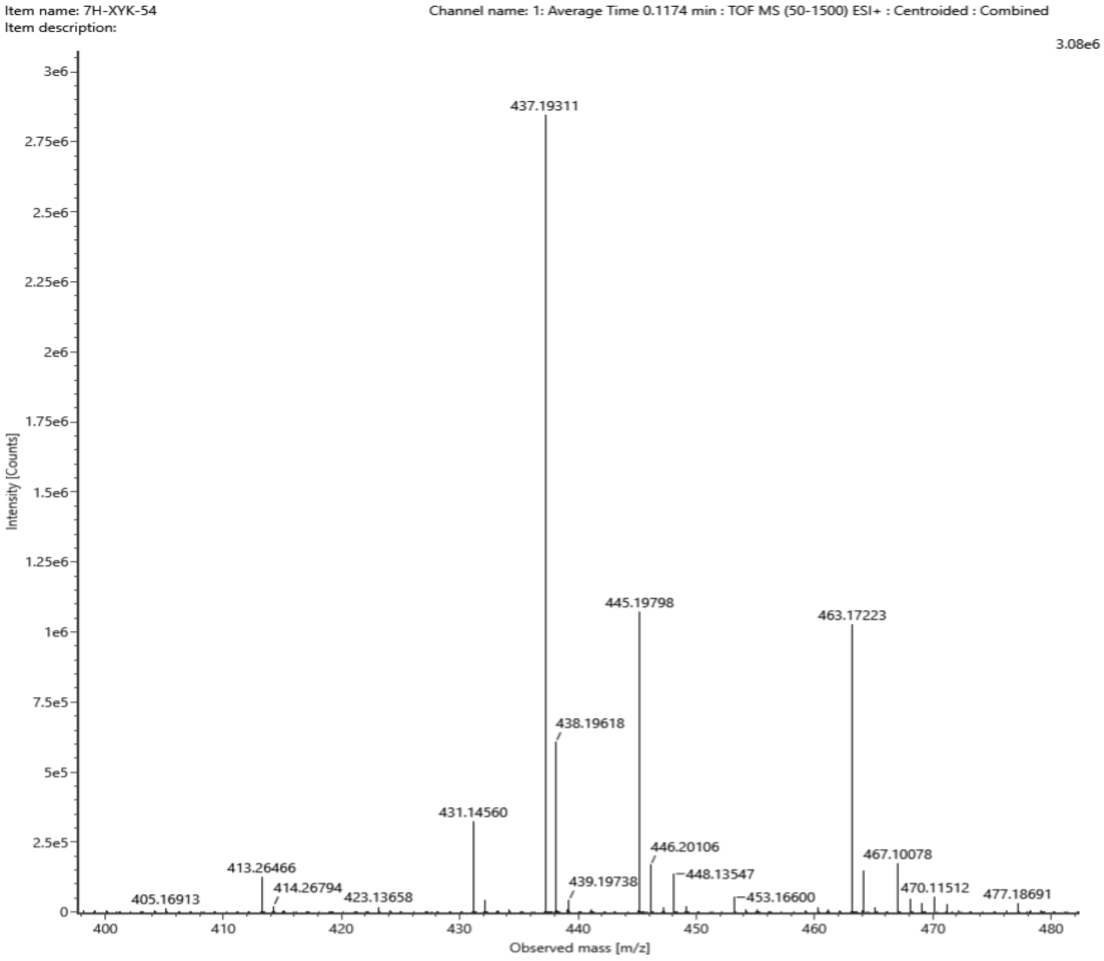

Figure S103. HRMS spectrum of compound **6h'**

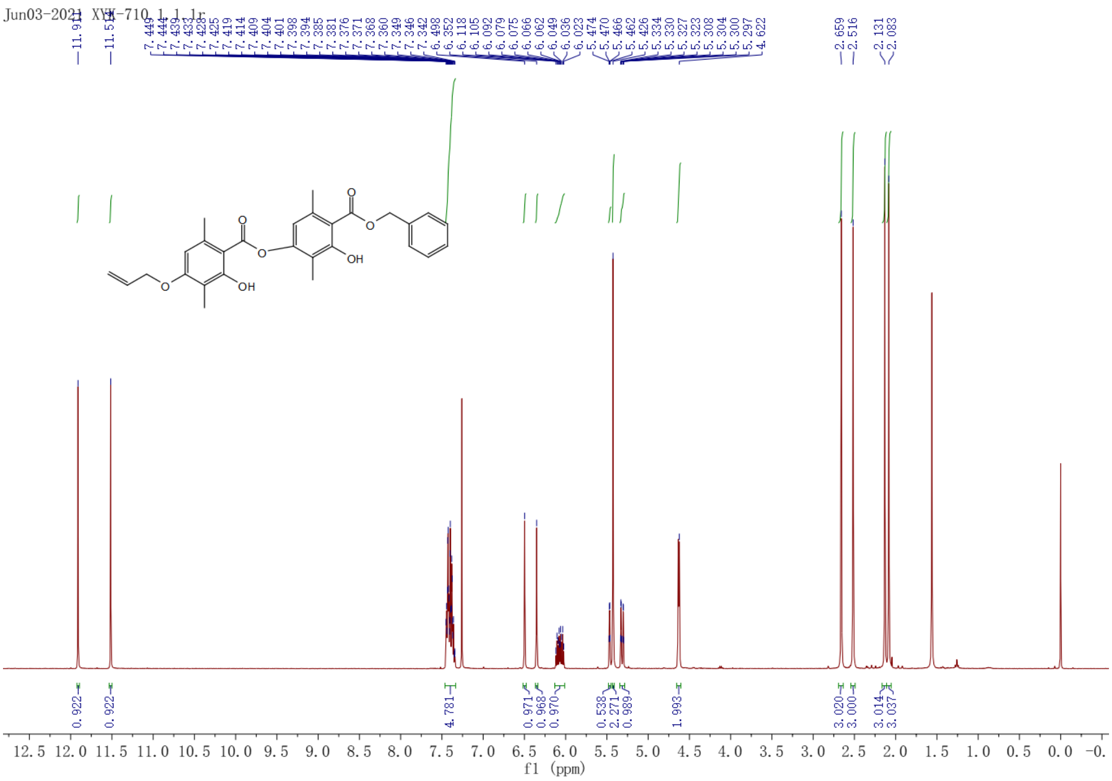

Figure S104. ^{1}H NMR spectrum of compound **6i'**

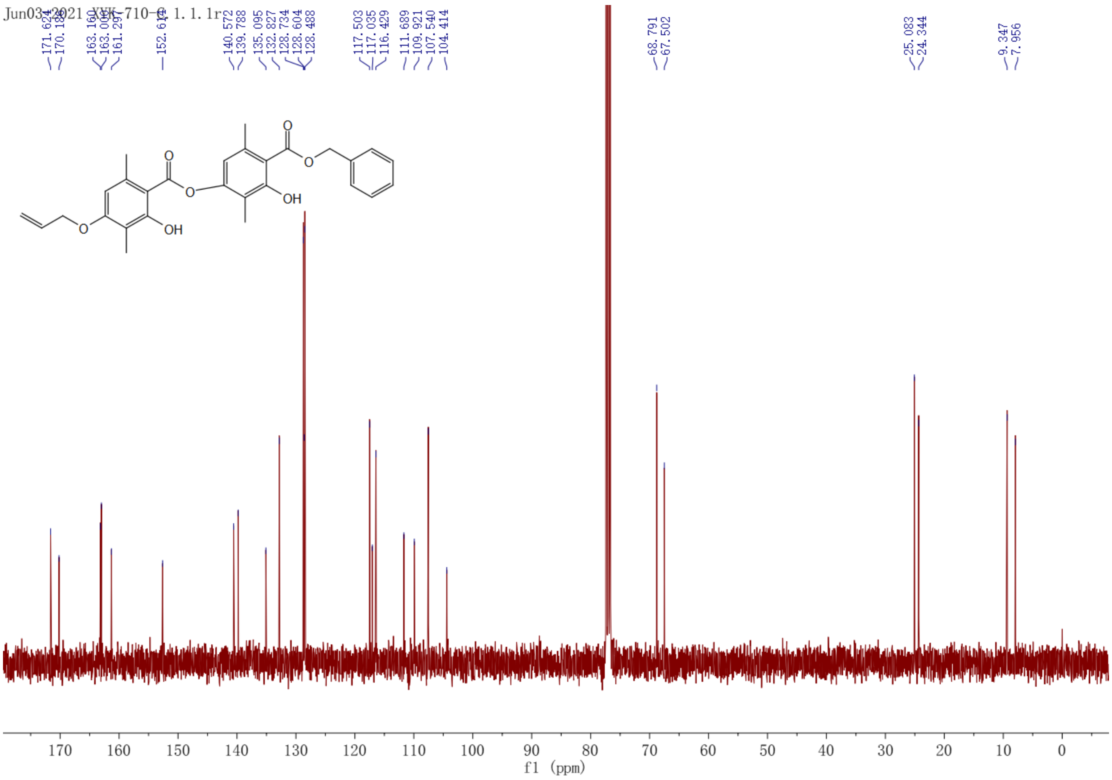

Figure S105. ^{13}C NMR spectrum of compound **6i'**

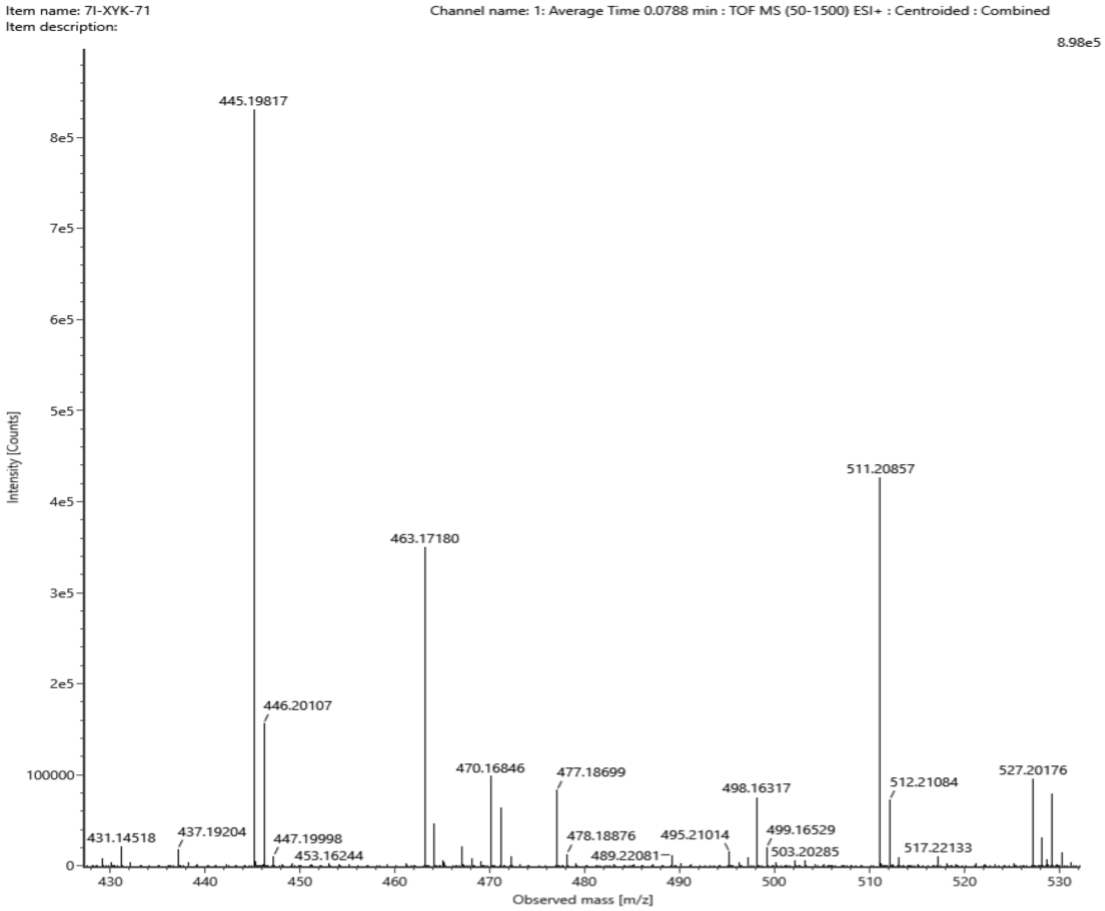

Figure S106. HRMS spectrum of compound **6i’**

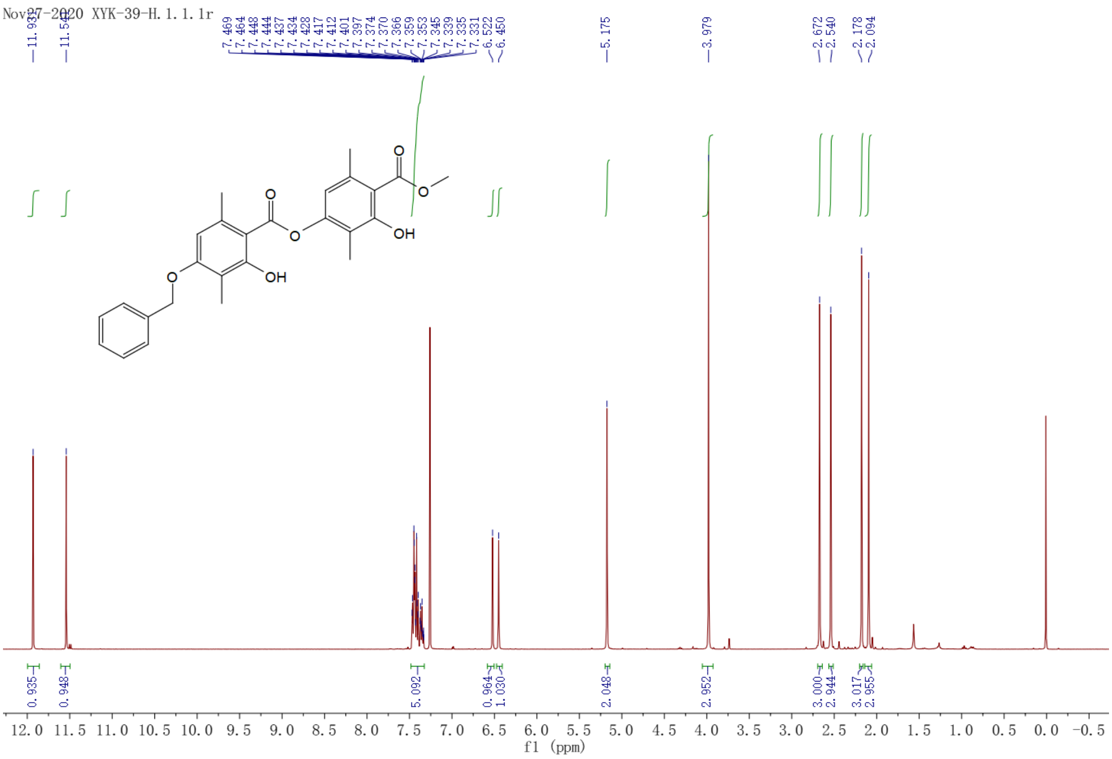

Figure S107. ^{1}H NMR spectrum of compound **6j’**

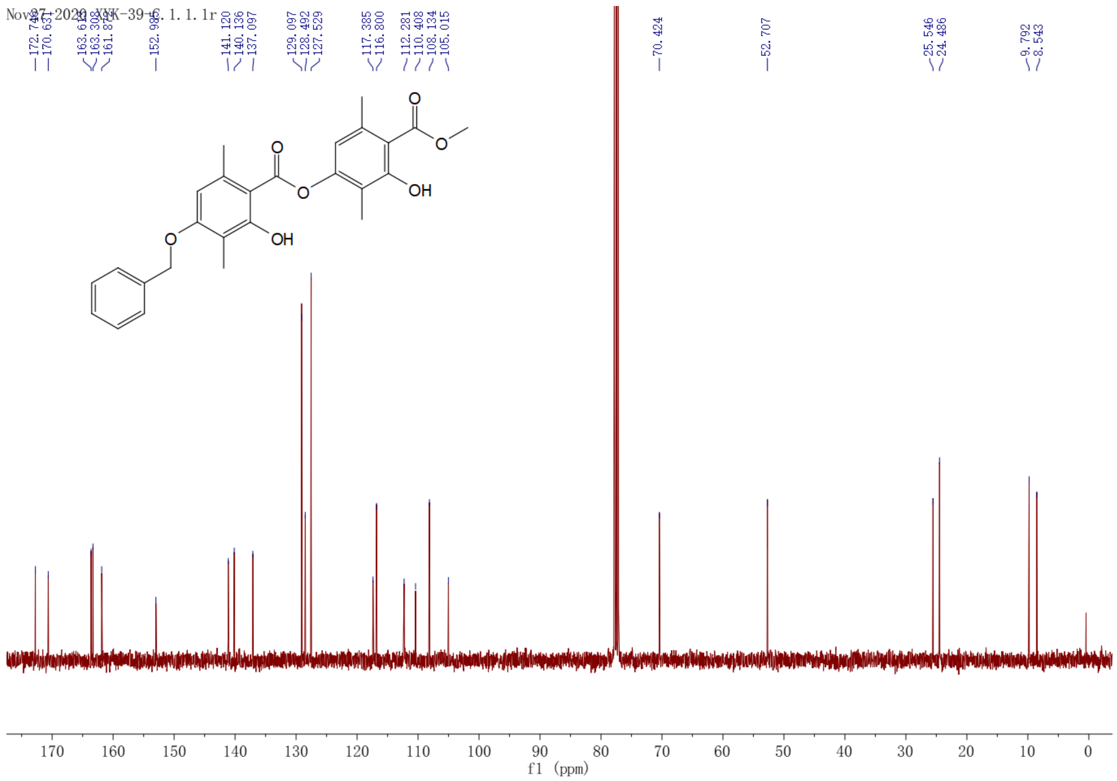

Figure S108. ^{13}C NMR spectrum of compound **6j’**

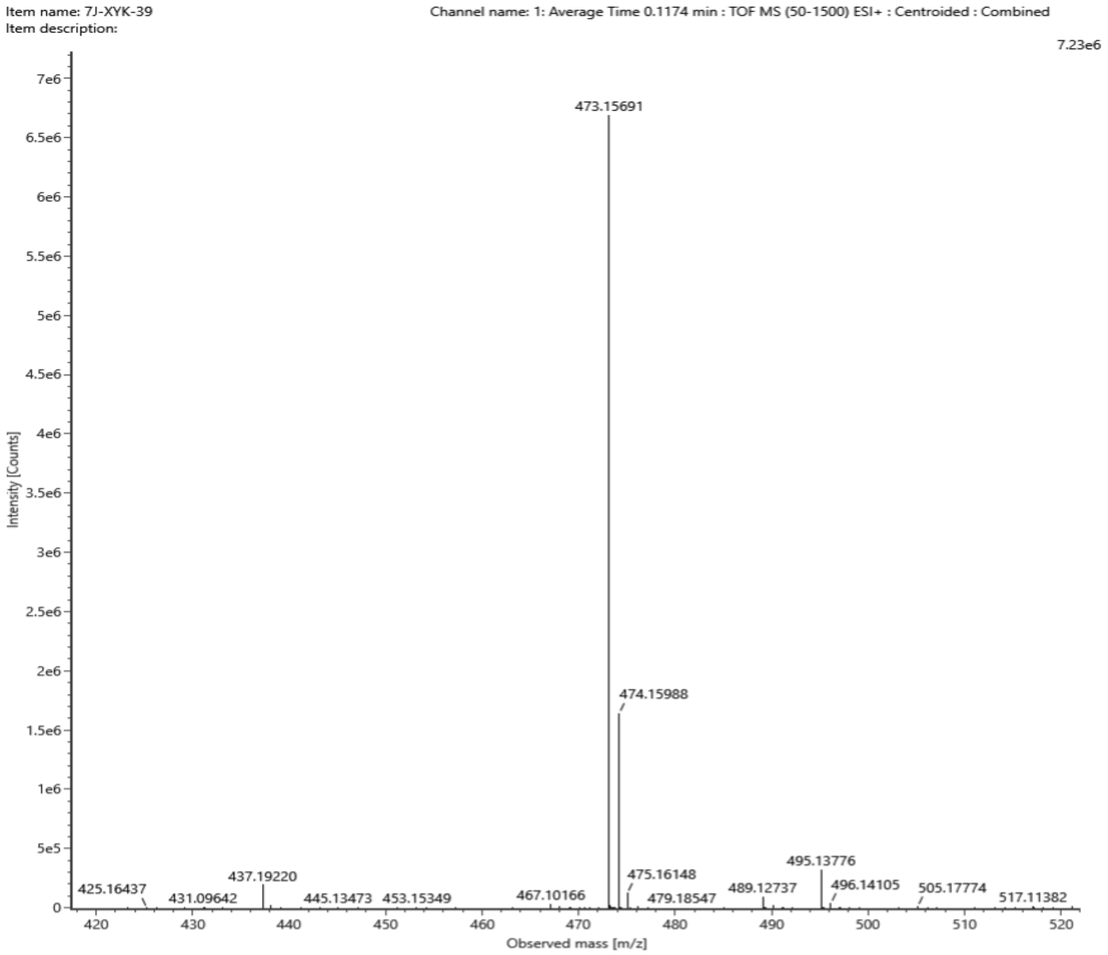

Figure S109. HRMS spectrum of compound **6j’**

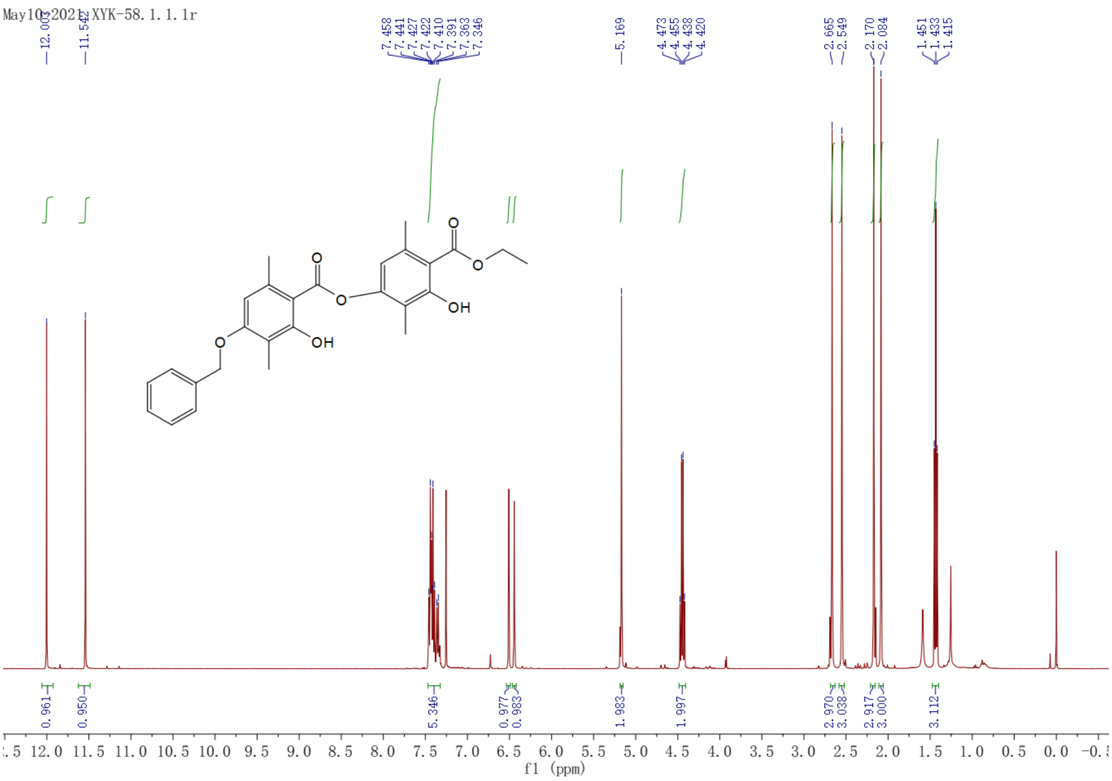

Figure S110. ^{1}H NMR spectrum of compound **6k’**

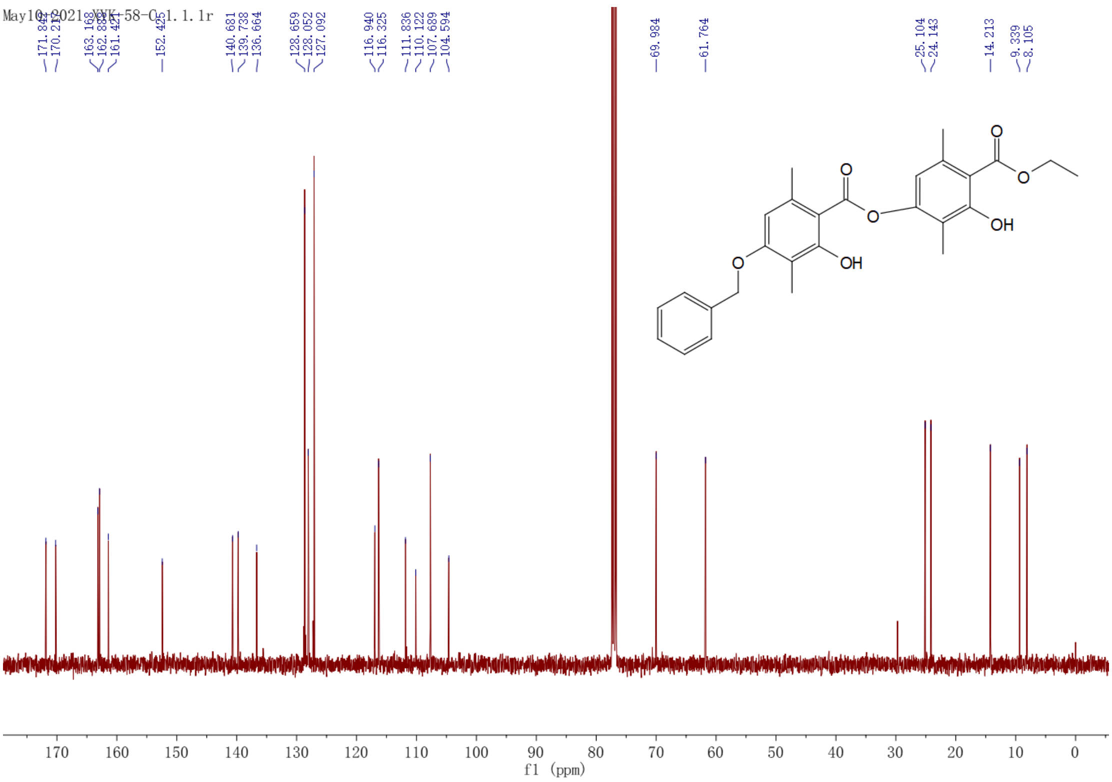

Figure S111. ^{13}C NMR spectrum of compound **6k’**

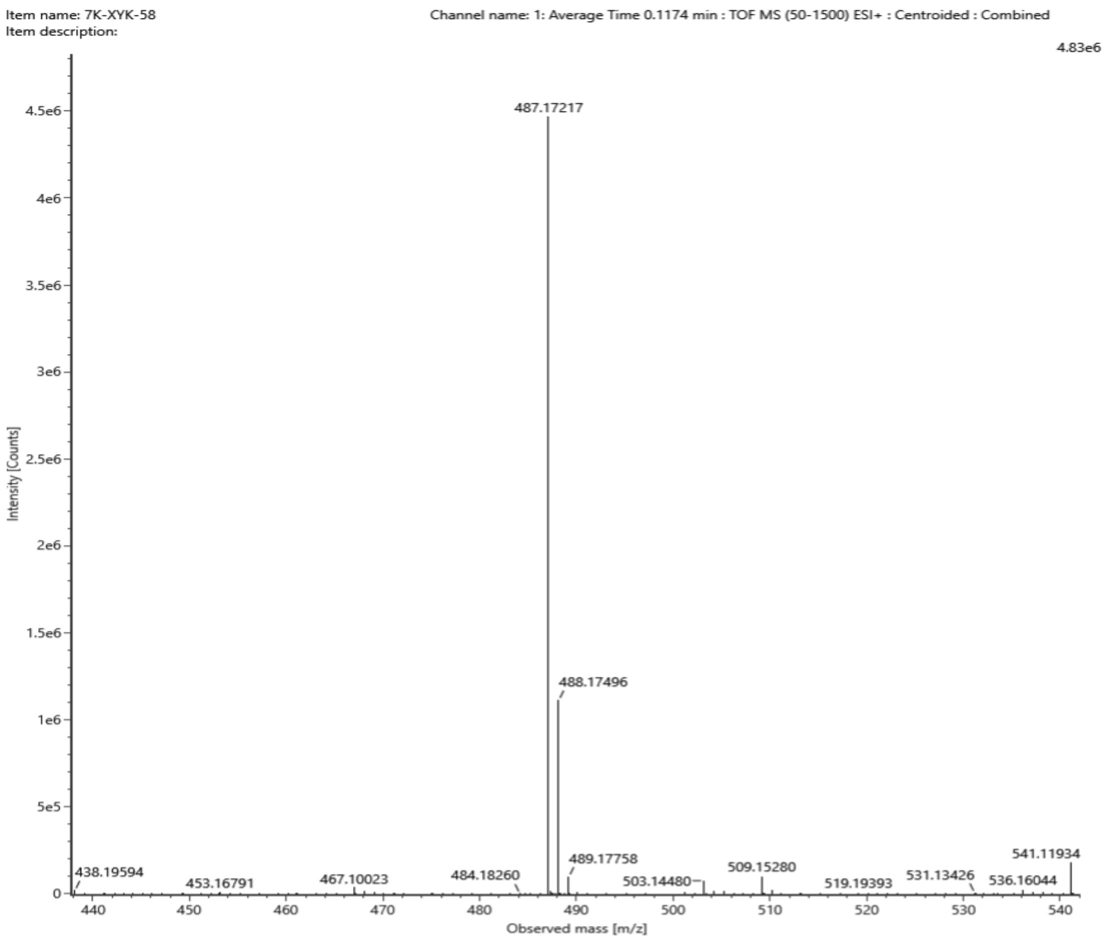

Figure S112. HRMS spectrum of compound **6k'**

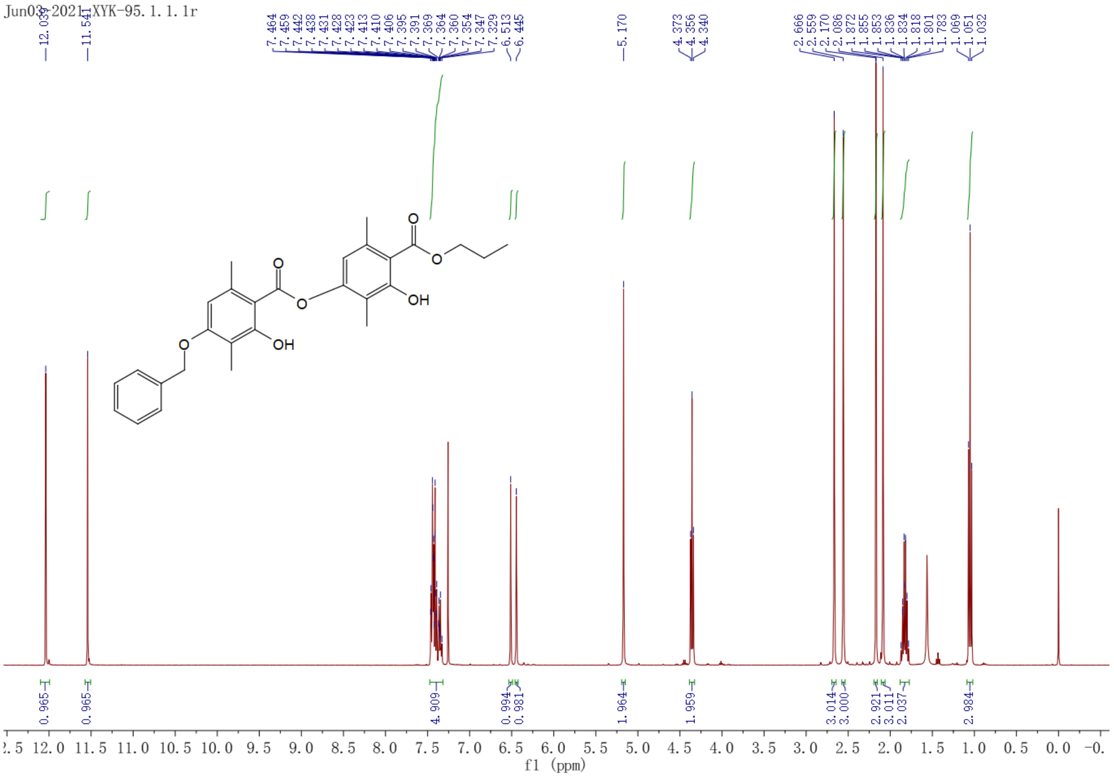

Figure S113. ^{1}H NMR spectrum of compound **6l'**

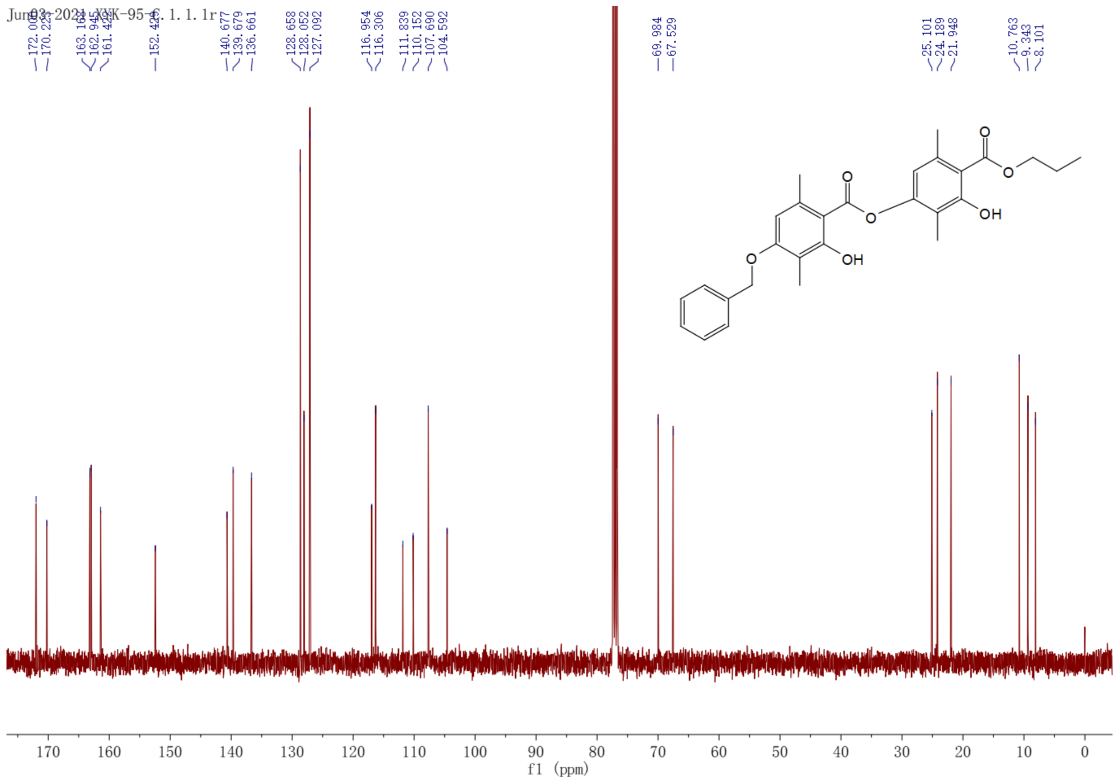

Figure S114. ^{13}C NMR spectrum of compound **6l’**

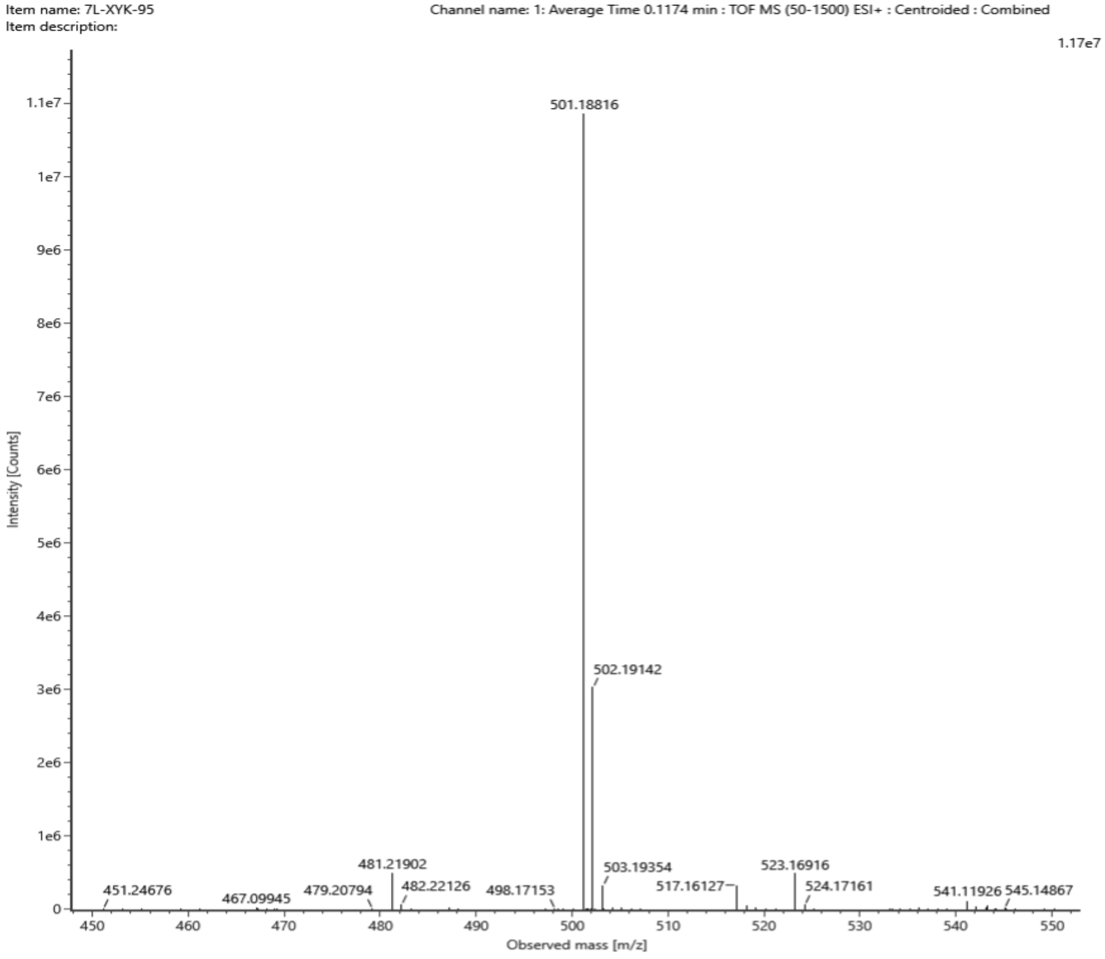

Figure S115. HRMS spectrum of compound **6l’**

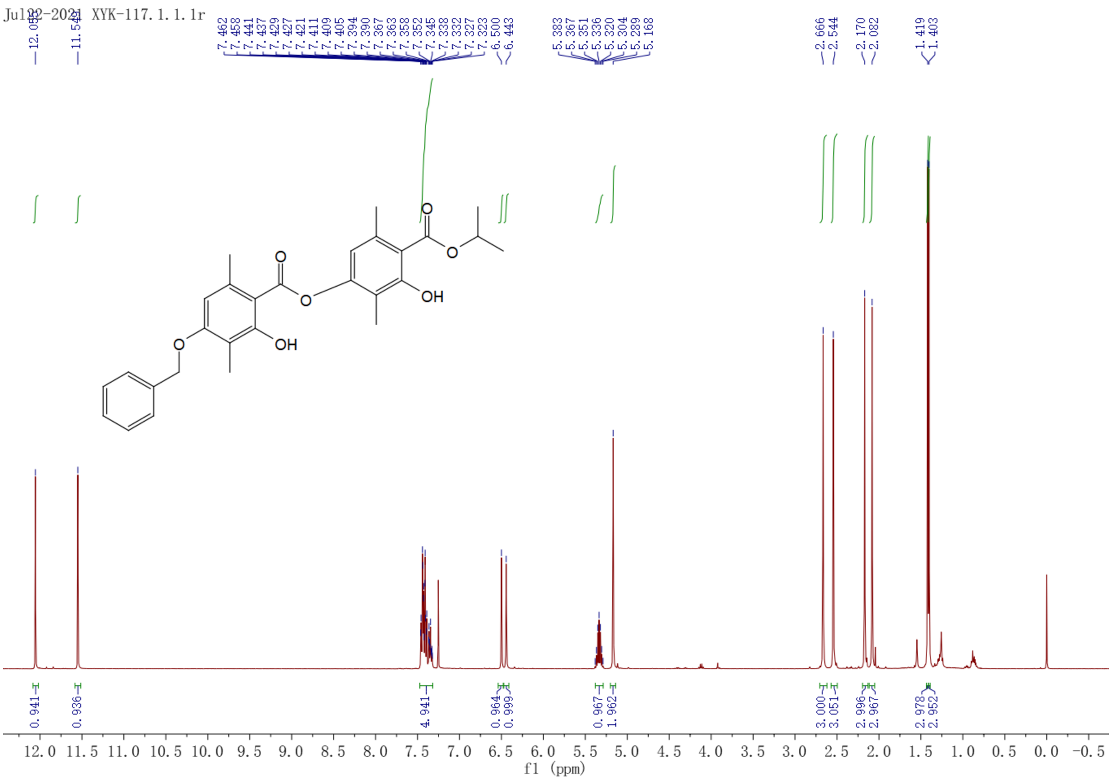

Figure S116. ^{1}H NMR spectrum of compound **6m’**

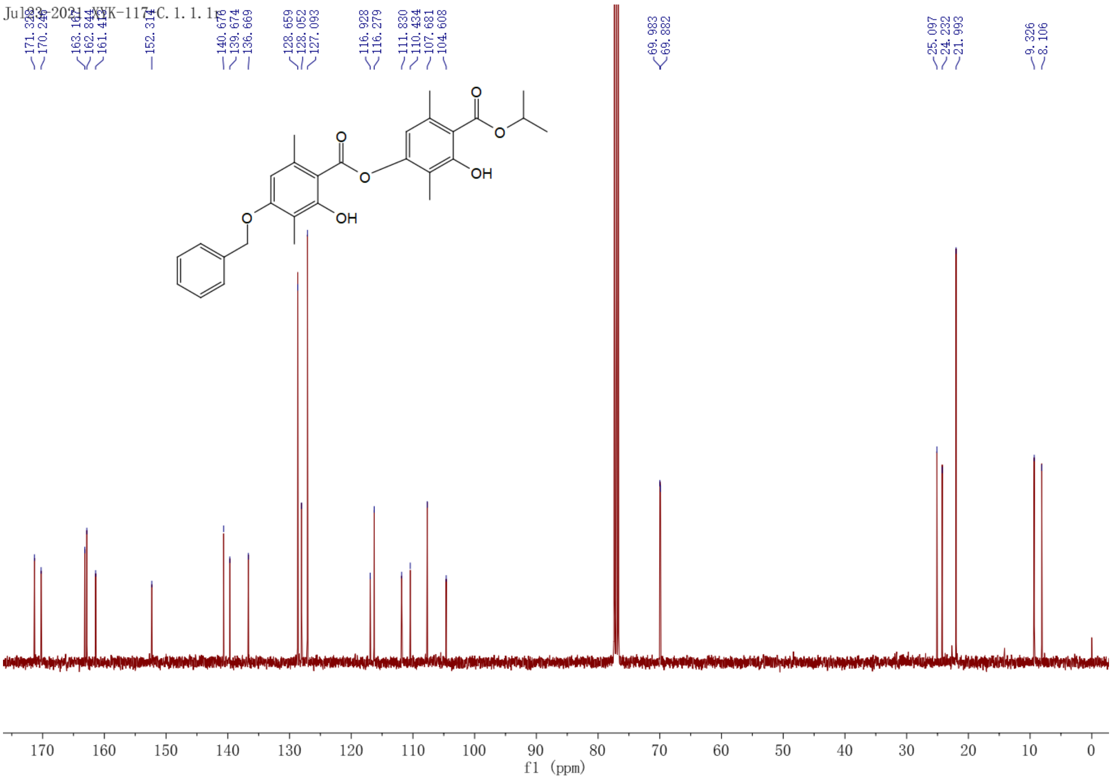

Figure S117. ^{13}C NMR spectrum of compound **6m’**

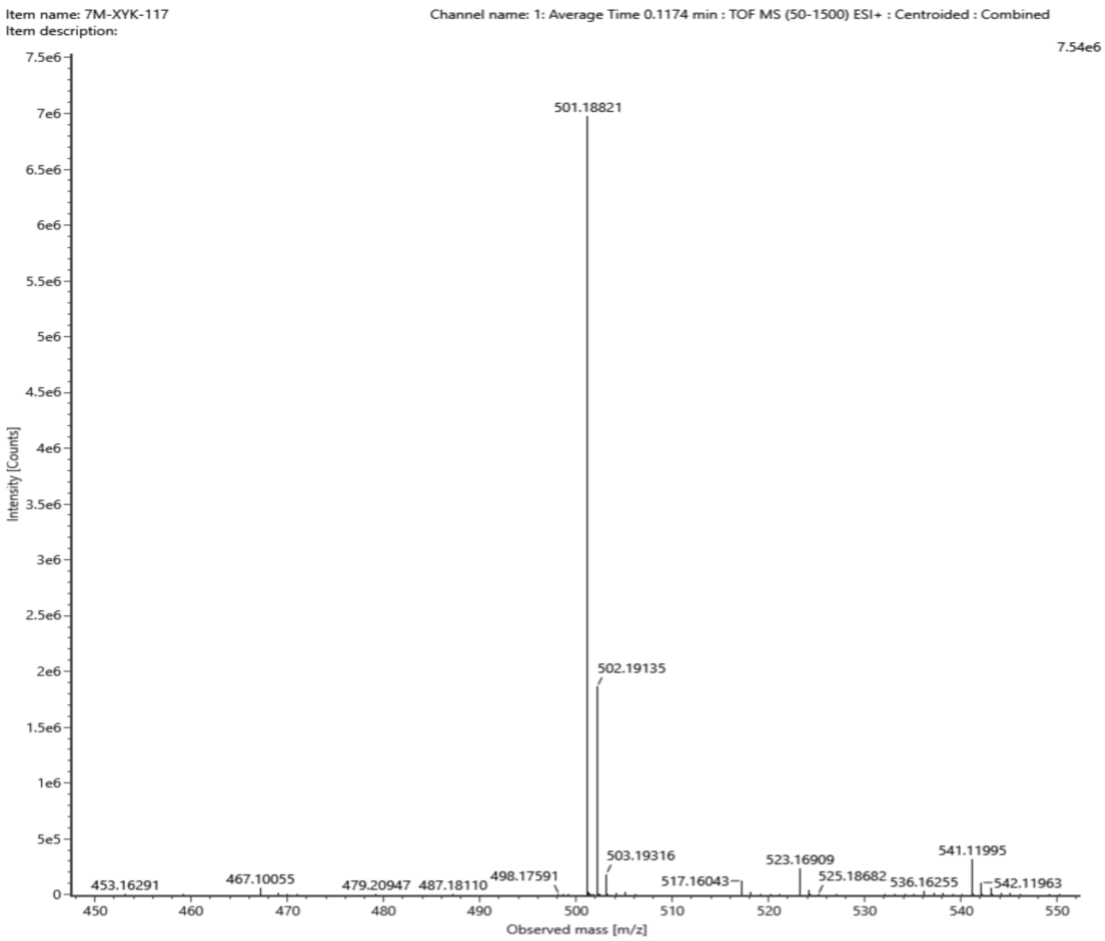

Figure S118. HRMS spectrum of compound **6m’**

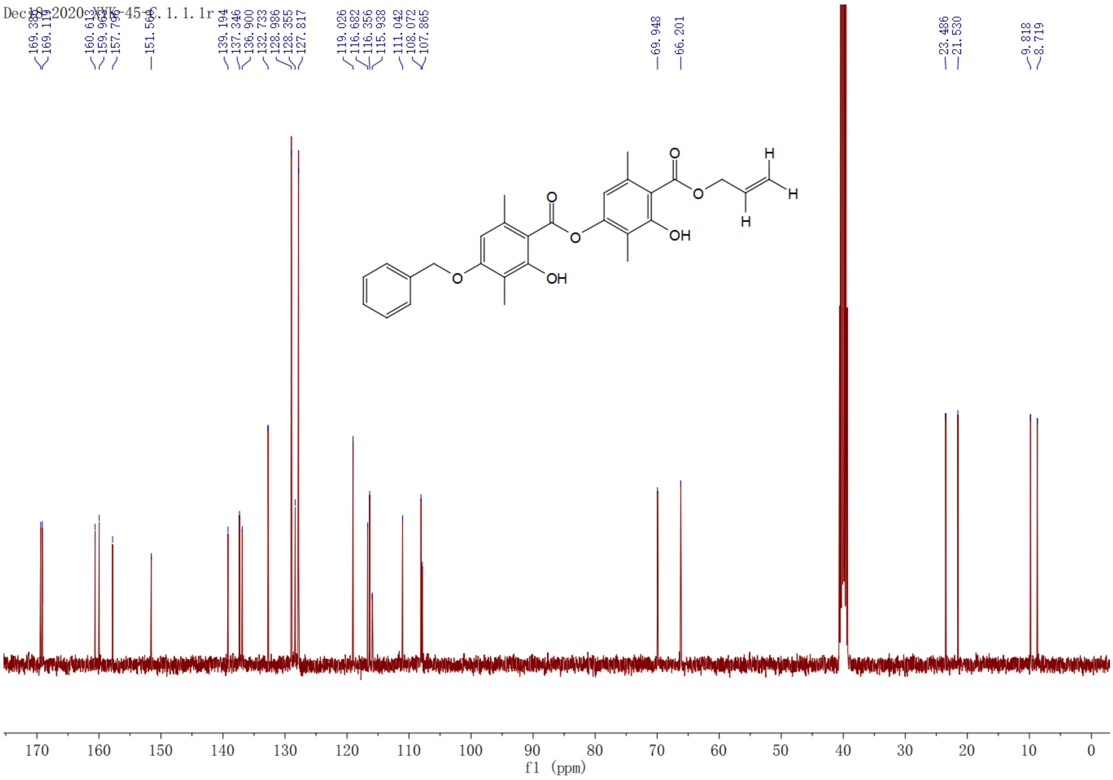

Figure S119. ^{1}H NMR spectrum of compound **6n’**

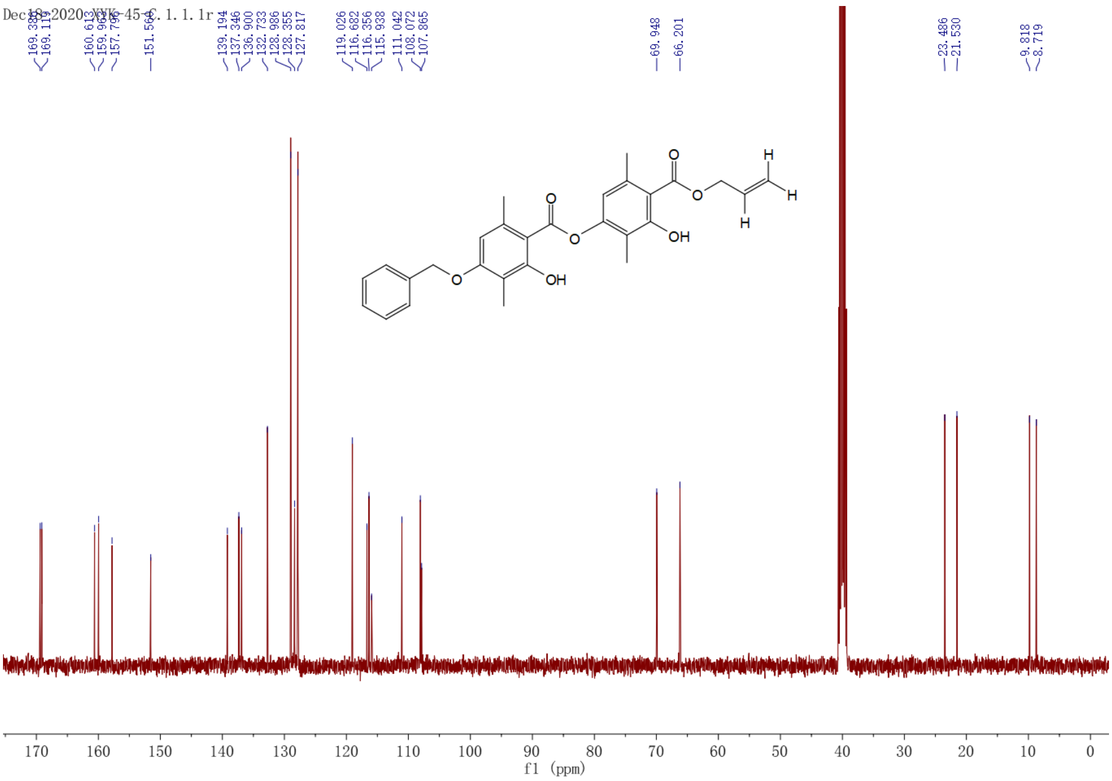

Figure S120. ^{13}C NMR spectrum of compound **6n'**

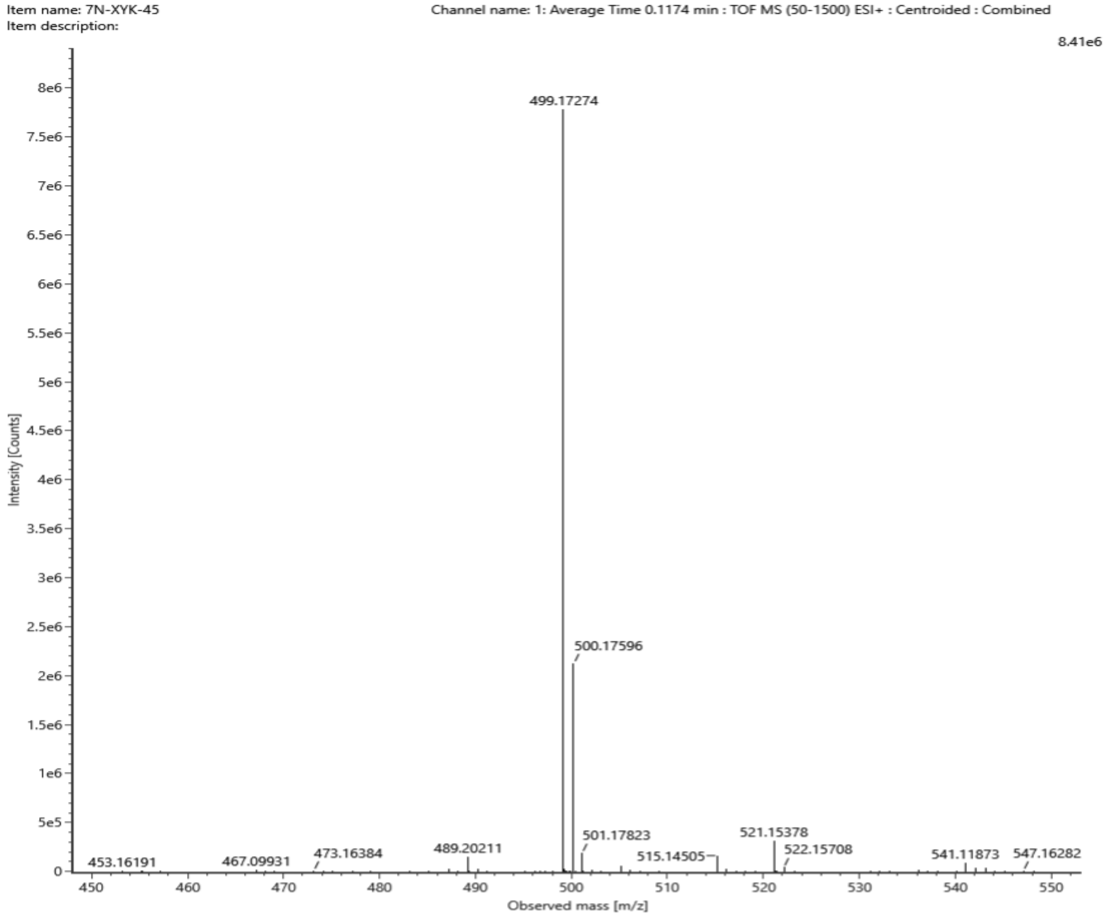

Figure S121. HRMS spectrum of compound **6n'**

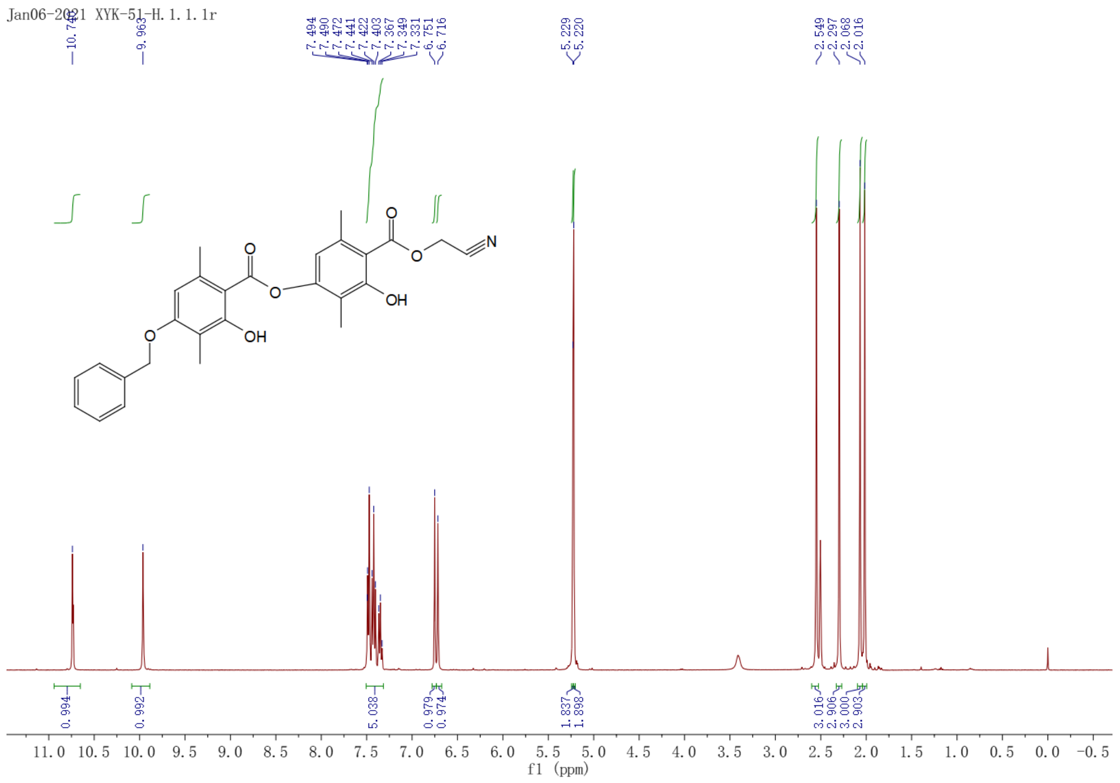

Figure S122. ^{1}H NMR spectrum of compound **6o’**

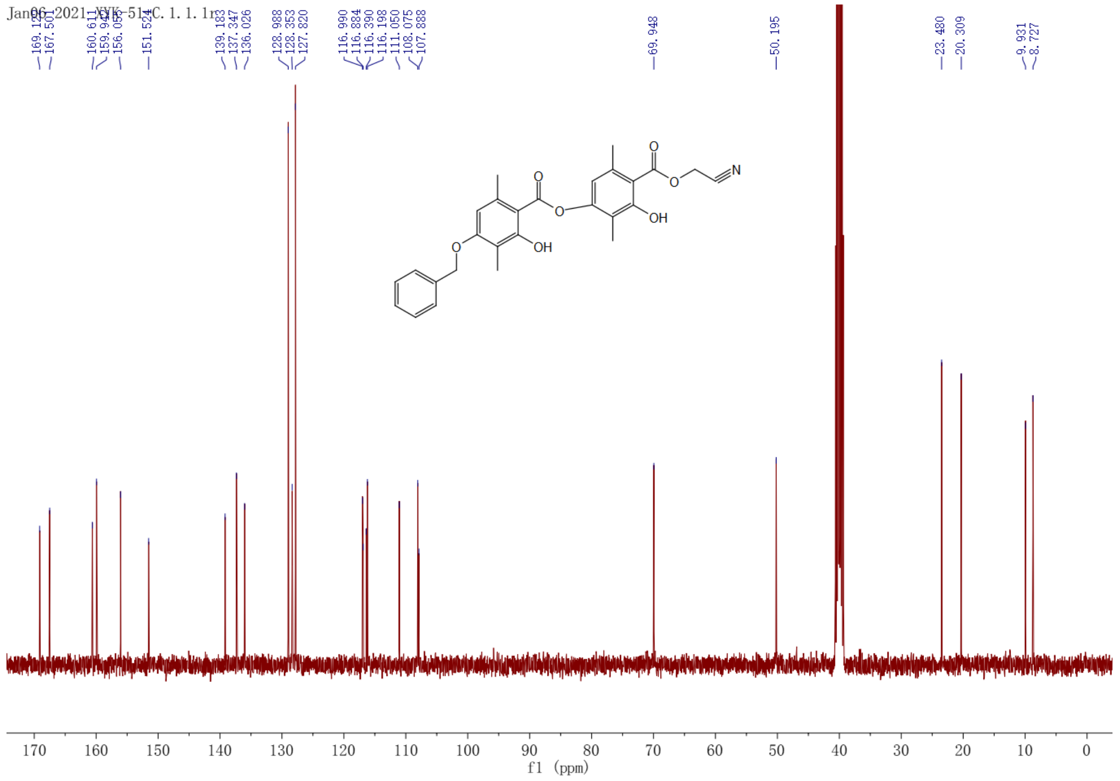

Figure S123. ^{13}C NMR spectrum of compound **6o’**

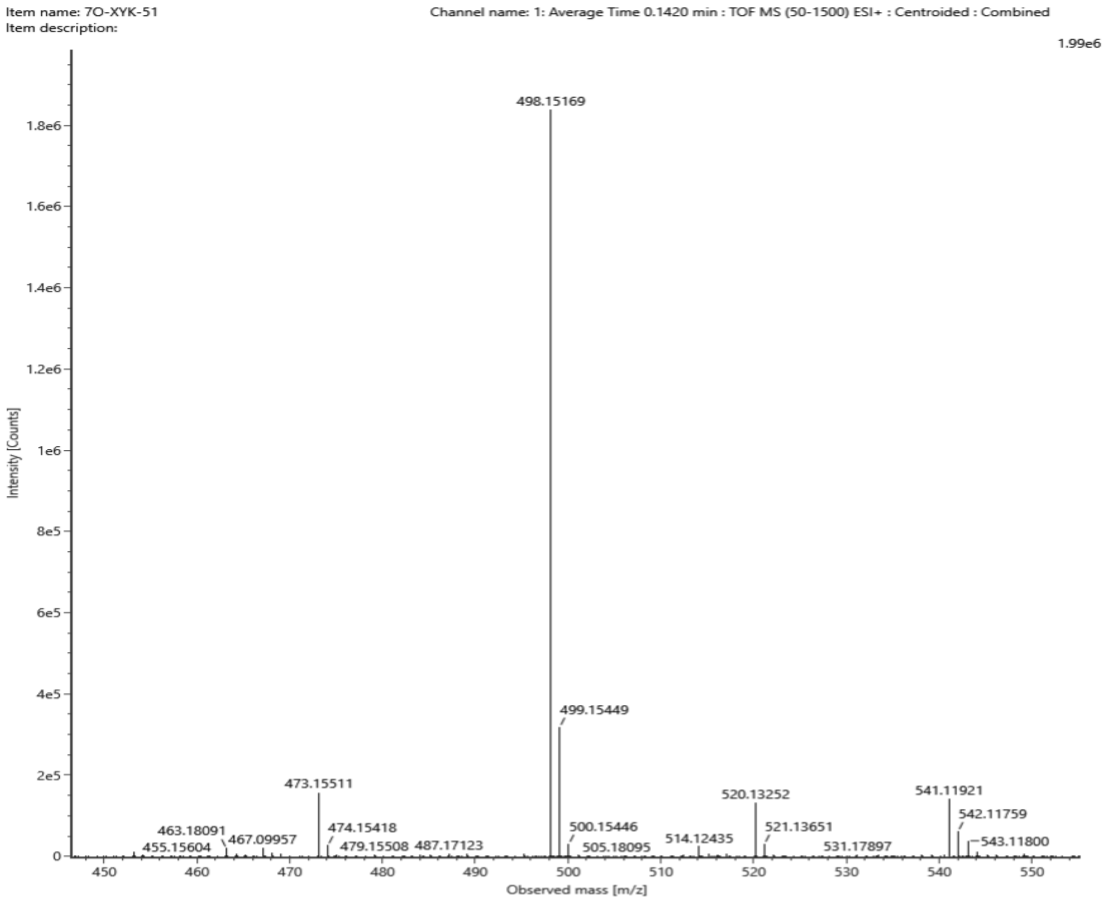

Figure S124. HRMS spectrum of compound **6o’**

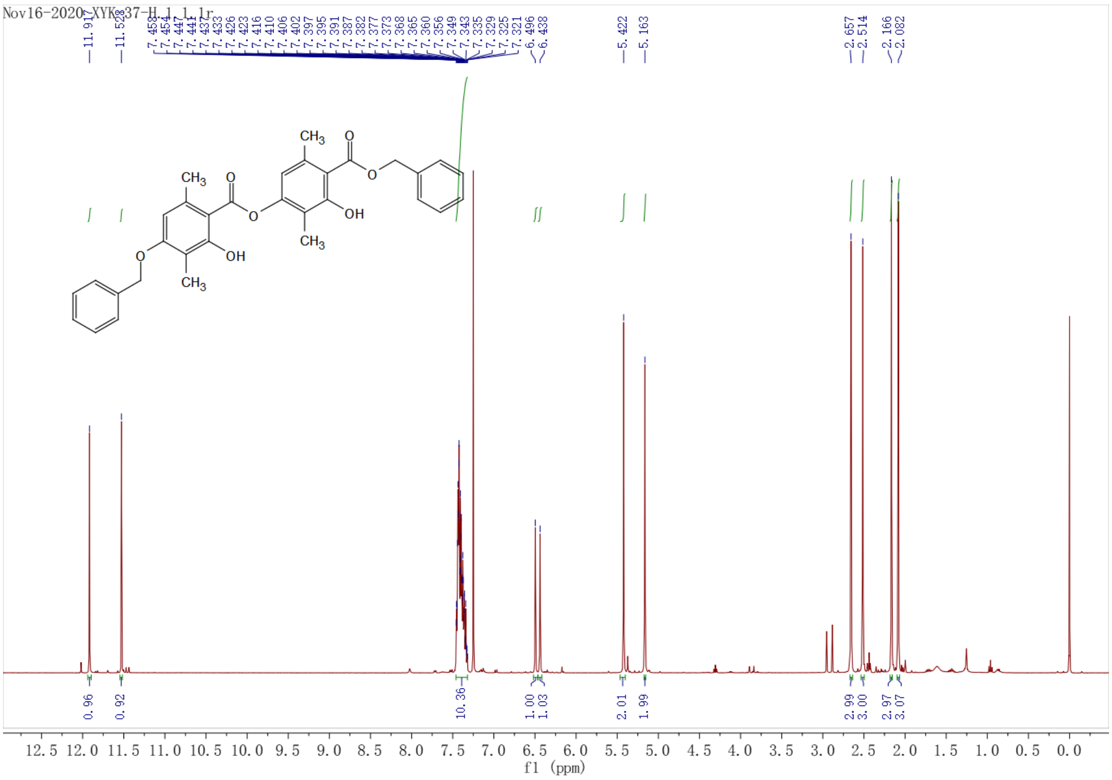

Figure S125. ^{1}H NMR spectrum of compound **6p’**

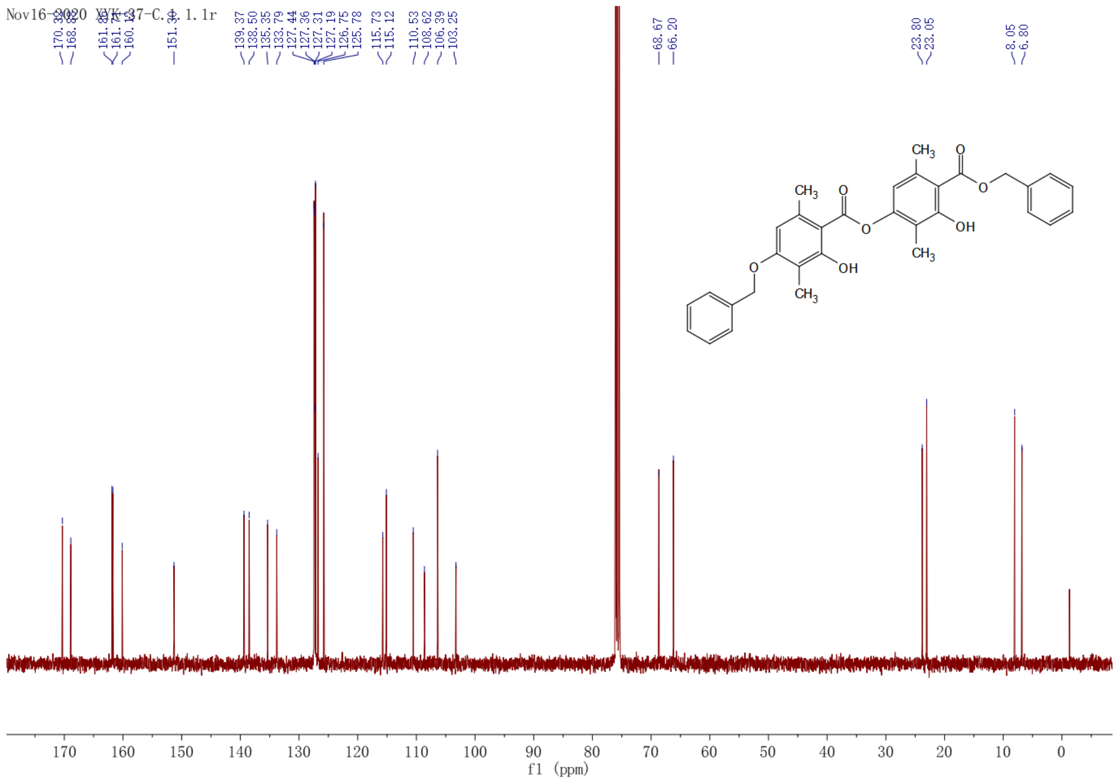

Figure S126. ^{13}C NMR spectrum of compound **6p'**

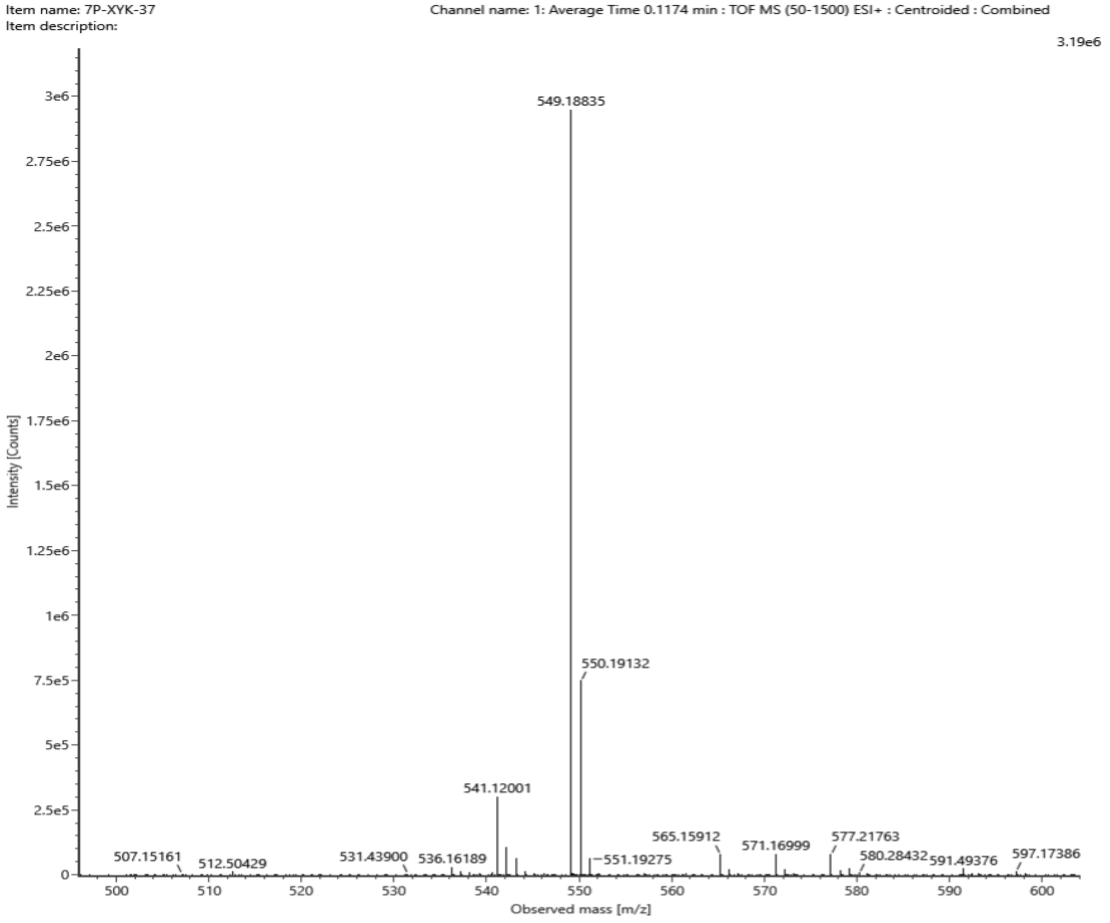

Figure S127. HRMS spectrum of compound **6p'**
